# Safety and Vision Outcomes of Subretinal Gene Therapy Targeting Cone Photoreceptors in Achromatopsia: A Nonrandomized Controlled Trial

**DOI:** 10.1001/jamaophthalmol.2020.1032

**Published:** 2020-04-30

**Authors:** M. Dominik Fischer, Stylianos Michalakis, Barbara Wilhelm, Ditta Zobor, Regine Muehlfriedel, Susanne Kohl, Nicole Weisschuh, G. Alex Ochakovski, Reinhild Klein, Christian Schoen, Vithiyanjali Sothilingam, Marina Garcia-Garrido, Laura Kuehlewein, Nadine Kahle, Annette Werner, Daniyar Dauletbekov, François Paquet-Durand, Stephen Tsang, Peter Martus, Tobias Peters, Mathias Seeliger, Karl Ulrich Bartz-Schmidt, Marius Ueffing, Eberhart Zrenner, Martin Biel, Bernd Wissinger

**Affiliations:** 1 University Eye Hospital, Centre for Ophthalmology, University Hospital Tübingen, Tübingen, Germany; 2 Institute for Ophthalmic Research, Centre for Ophthalmology, University Hospital Tübingen, Tübingen, Germany; 3 Centre for Ophthalmology, University Hospital Tübingen, Tübingen, Germany; 4 Center for Integrated Protein Science Munich, Department of Pharmacy, Center for Drug Research, Ludwig-Maximilians-University of Munich, Munich, Germany; 5 Department of Ophthalmology, Ludwig-Maximilians-Universität München, Munich, Germany; 6 Department of Internal Medicine II, University Hospital Tübingen, Germany; 7 Department of Ophthalmology, College of Physicians and Surgeons, Columbia University, New York, New York; 8 Institute for Clinical Epidemiology and Applied Biostatistics, University Hospital Tübingen, Tübingen, Germany

## Abstract

**Importance:**

Achromatopsia linked to variations in the *CNGA3* gene is associated with day blindness, poor visual acuity, photophobia, and involuntary eye movements owing to lack of cone photoreceptor function. No treatment is currently available.

**Objective:**

To assess safety and vision outcomes of supplemental gene therapy with adeno-associated virus (AAV) encoding *CNGA3* (AAV8.CNGA3) in patients with *CNGA3*-linked achromatopsia.

**Design, Setting, and Participants:**

This open-label, exploratory nonrandomized controlled trial tested safety and vision outcomes of gene therapy vector AAV8.CNGA3 administered by subretinal injection at a single center. Nine patients (3 per dose group) with a clinical diagnosis of achromatopsia and confirmed biallelic disease-linked variants in *CNGA3* were enrolled between November 5, 2015, and September 22, 2016. Data analysis was performed from June 6, 2017, to March 12, 2018.

**Intervention:**

Patients received a single unilateral injection of 1.0 × 10^10^, 5.0 × 10^10^, or 1.0 × 10^11^ total vector genomes of AAV8.CNGA3 and were followed up for a period of 12 months (November 11, 2015, to October 10, 2017).

**Main Outcomes and Measures:**

Safety as the primary end point was assessed by clinical examination of ocular inflammation. Systemic safety was assessed by vital signs, routine clinical chemistry testing, and full and differential blood cell counts. Secondary outcomes were change in visual function from baseline in terms of spatial and temporal resolution and chromatic, luminance, and contrast sensitivity throughout a period of 12 months after treatment.

**Results:**

Nine patients (mean [SD] age, 39.6 [11.9] years; age range, 24-59 years; 8 [89%] male) were included in the study. Baseline visual acuity letter score (approximate Snellen equivalent) ranged from 34 (20/200) to 49 (20/100), whereas baseline contrast sensitivity log scores ranged from 0.1 to 0.9. All 9 patients underwent surgery and subretinal injection of AAV8.CNGA3 without complications. No substantial safety problems were observed during the 12-month follow-up period. Despite the congenital deprivation of cone photoreceptor–mediated vision in achromatopsia, all 9 treated eyes demonstrated some level of improvement in secondary end points regarding cone function, including mean change in visual acuity of 2.9 letters (95% CI, 1.65-4.13; *P* = .006, 2-sided *t* test paired samples). Contrast sensitivity improved by a mean of 0.33 log (95% CI, 0.14-0.51 log; *P* = .003, 2-sided *t* test paired samples).

**Conclusions and Relevance:**

Subretinal gene therapy with AAV8.CNGA3 was not associated with substantial safety problems and was associated with cone photoreceptor activation in adult patients, as reflected by visual acuity and contrast sensitivity gains.

**Trial Registration:**

ClinicalTrials.gov Identifier: NCT02610582

## Key Points

**Question:**

What are the safety and vision outcomes associated with gene therapy for achromatopsia?

**Findings:**

In this nonrandomized controlled trial of 9 patients with confirmed *CNGA3*-linked achromatopsia, gene therapy applying an adeno-associated viral vector encoding CNGA3, was not associated with substantial safety concerns and was associated with improvements of vision in patients.

**Meaning:**

This study provides clinical proof of concept for viral vector–mediated gene supplementation therapy of inherited day blindness caused by pathogenic variants in the cone photoreceptor-specific gene *CNGA3*.

## Teaser

This nonrandomized controlled trial assesses safety and vision outcomes of gene therapy for patients with achromatopsia.

## Introduction

Achromatopsia is an inherited disease that affects cone photoreceptors in the retina. Individuals with achromatopsia demonstrate a total lack of function of all 3 types of cones in the retina.^1^ Achromatopsia is clinically characterized by day blindness (hemeralopia), glare, poor visual acuity, involuntary oscillatory movement of the eyes (nystagmus), and failure to discriminate chromatic contrasts (achromatopsia). In contrast to common forms of color blindness, in which alterations in the opsin genes affect spectral sensitivity only, patients with achromatopsia lack any cone response from birth. Consequently, patients do not report progression of symptoms, and the disease was initially thought to be nonprogressive. Previous studies,^2,3,4^ however, established structural alterations and foveal lesions that emerge with age and are consistent with a slowly progressive degeneration and loss of cone photoreceptor cells in patients with achromatopsia. Variants in 6 genes are implicated in achromatopsia, which together likely explain more than 90% of cases.^5,6^ Most prevalent are pathogenic variants in the 2 genes that encode the α and β subunits of the cone cyclic nucleotide-gated (CNG) channel, *CNGA3* (found in approximately 25%-28% of European and US cases) and *CNGB3* (50% of cases).^7^ CNG channels are essential components of the phototransduction process in cone photoreceptors, which enable daylight vision, high spatial and temporal resolution, color discrimination, and stable fixation. No treatment is currently available for achromatopsia, and any procedure that results in improvement of day blindness and visual function is therefore desired.

Supplemental gene therapy is successfully applied in other inherited retinal diseases due to, for example, mutations in *RPE65*.^8^ The US Food and Drug Administration and the European Medicines Agency recently approved the first gene therapy drug for inherited retinal diseases caused by variants in *RPE65*.^9^ The primary targets for *RPE65* gene therapy are cells of the retinal pigment epithelium. In contrast, our gene therapy approach for *CNGA3*-linked achromatopsia was designed to specifically treat cone photoreceptors. Such an approach was proven successful in a *Cnga3*-deficient mouse model on retinal and behavioral levels.^10^ Preclinical toxicologic studies^11,12^ in nonhuman primates demonstrated no adverse effect and limited biodistribution after subretinal application. We therefore initiated a trial in November 2015 to assess the safety and efficacy of a gene therapy approach that targeted the remaining cone photoreceptors in patients with achromatopsia. We used adeno-associated virus 8 (AAV8) as a vector with proven ability to transduce photoreceptors,^13^ opted for a cone-specific promoter (derived from the human cone arrestin 3 gene *ARR3*), and delivered the vector in a subretinal bleb under the cone-rich macula of patients with achromatopsia due to variants in *CNGA3*. This first, to our knowledge, trial in human patients was designed to evaluate the safety and tolerability of such an approach, to give insights into the association of the treatment with changes in visual function, and to assess the potential utility of functional end points for future studies.

## Methods

### Study Design and Oversight

This open-label, exploratory nonrandomized controlled trial included patients with *CNGA3*-linked achromatopsia enrolled in 3 dose groups from November 5, 2015, to September 22, 2016. Thirty-six patients were initially assessed, and 21 were deemed unsuitable in the investigator‘s opinion (eg, residual cone response in electroretinography or very poor fixation). Of these 15 patients, 6 did not consent to the study. All patients provided written informed consent, and patients were deidentified for the data analysis. Study protocol and documentation were approved by the Paul Ehrlich Institute and the local ethics board of the University Hospital Tübingen. An independent data monitoring committee evaluated the safety data and gave a go or no-go decision for each dose escalation step. International Conference on Harmonization Good Clinical Practice monitoring was performed independently and on behalf of the sponsor (Universitätsklinikum Tübingen). This study followed the Transparent Reporting of Evaluations With Nonrandomized Designs (TREND) reporting guideline. The trial protocol can be found in Supplement 1.

### Patients

**Figure 1.**
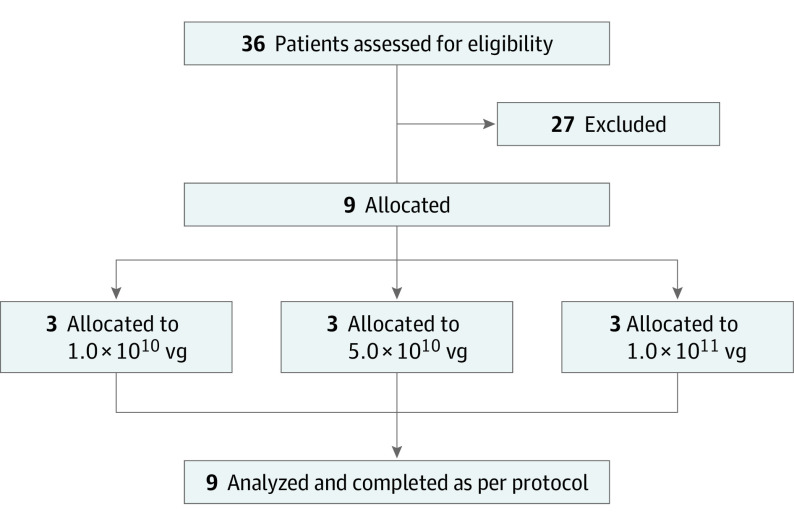
CONSORT Flow Diagram vg indicates vector genomes.

Nine patients (8 men and 1 woman) with a clinical phenotype of complete achromatopsia and confirmed biallelic variants in the *CNGA3* gene were enrolled into this trial (Figure 1); eligibility criteria are given in the eAppendix in Supplement 2. Three patients each were assigned to 1 of 3 groups with escalating doses (1 × 10^10^ total vector genomes [vg], 5 × 10^10^ vg, and 1 × 10^11^ vg) with the aim of investigating first the safety and tolerability and second the therapeutic efficacy of AAV8.CNGA3. The patients received a single unilateral injection of AAV8.CNGA3 and were followed up for a period of 12 months (November 11, 2015, to October 10, 2017). For ethical reasons, the worse eye based on clinical examination and subjective judgment by the patient was used as the study eye in this trial. Patients were not prescreened for immunity to AAV8.

### Vector Production

The vector was produced according to Good Manufacturing Practice requirements at Atlantic BioGMP. Details are described in the eAppendix in Supplement 2. In brief, the expression cassette is based on the AAV2 genome and combines a human cone arrestin (*ARR3*) promoter with the human *CNGA3* complementary DNA.^14,15,16^

### Preclinical Testing

Transgene expression (immune labeling of CNGA3 after subretinal gene therapy of *Cnga3^−/−^* mice) and biological activity (electroretinography after subretinal gene therapy of *Cnga3^−/−^* mice) were assessed with different engineering lots and the final clinical lot (eAppendix in Supplement 2). Short-term (n = 12, 28 days) toxicologic and long-term (n = 22, 13 weeks) toxicologic and biodistribution studies in nonhuman primates demonstrated that AAV8.CNGA3 can safely be applied for subretinal injections.^11,12,17^

### Surgery and Concomitant Medication

AAV8.CNGA3 was delivered by subretinal injection in all 9 patients after standard 23-gauge vitrectomy with the patient under general anesthesia using a 2-step approach described previously.^18^ First, a sterile irrigating solution (BSS, Alcon) was injected using an extendable 41-gauge cannula (1270.EXT, DORC) to create a localized retinal detachment (prebleb). Second, a fixed volume (0.2 mL) of AAV8.CNGA3 was injected into the prebleb formation through the same retinotomy using 7- to 14-kPa positive pressure on the infusion program of the PentraSys system.^18^ All operations were performed without complications. Patients were treated with a 21-day oral course of prednisolone (starting 1 day before surgery) at 1 mg/kg body weight, which was then tapered out. Topical glucocorticoid and antibiotic medication was administered as prophylaxis against inflammation and infection in accordance with the drug regimen used routinely in our hospital after vitreoretinal surgical procedures (eAppendix in Supplement 2).

### Clinical Investigation

Best-corrected visual acuity (to rule out loss of ≥15 letters in visual acuity at 1 m) and clinical examinations of the eye (to rule out severe, vector-induced intraocular inflammation unresponsive to treatment) were performed to examine the primary end point (safety).^19^ Systemic safety parameters included vital signs, clinical chemical analyses (eg, C-reactive protein level, IgG titer, IgM titer, and differential blood cell counts), quantitative polymerase chain reaction–based analysis of vector shedding and biodistribution, an enzyme-linked immunosorbent assay quantifying antibody titers against AAV8 capsid proteins, and lymphocyte transformation tests and supernatant cytokine assays to monitor cellular immune reactivity against AAV8.^11,20^ Details are given in eTables 1 through 6 in Supplement 2.

To explore the efficacy of AAV8.CNGA3 gene therapy, a number of functional tests centered around cone photoreceptor function were performed by an assessor masked to the treatment.^19^ These tests included assessments of visual acuity and contrast sensitivity, color vision, flicker fusion frequency, full-field stimulus threshold, pupillography, microperimetry, and questionnaire-based measures of patient-reported outcome measures at baseline and during follow-up for 1 year. Details are given in eTables 9 through 11 in Supplement 2.

### Statistical Analysis

We present the absolute values for 9 patients alongside the calculated mean (±2 SDs) for the 3 dose subgroups (n = 3) and all treated eyes (n = 9) over time. The exploratory outcomes were assessed as the change from the baseline measurement to 12 months using parametric (2-sided *t* test) and nonparametric (Wilcoxon signed rank test) analyses. A 2-sided *P* > .05 was interpreted as not statistically significant. The trial was not designed to formally test efficacy, and no formal sample size estimation was performed a priori. Therefore, the number of statistical tests for different clinical parameters with *P* < .05 was compared with the expected number of false-positive calls owing to multiple testing (eAppendix in Supplement 2). To combine findings from different clinical efficacy end points, standardized scores (*z* scores) were calculated by using the individual baseline value and the SD of all 9 measurements at baseline to achieve a dimensionless quantity. Data analysis was performed from June 6, 2017, to March 12, 2018.

## Results

### Patient Characteristics and Intervention

**Figure 2.**
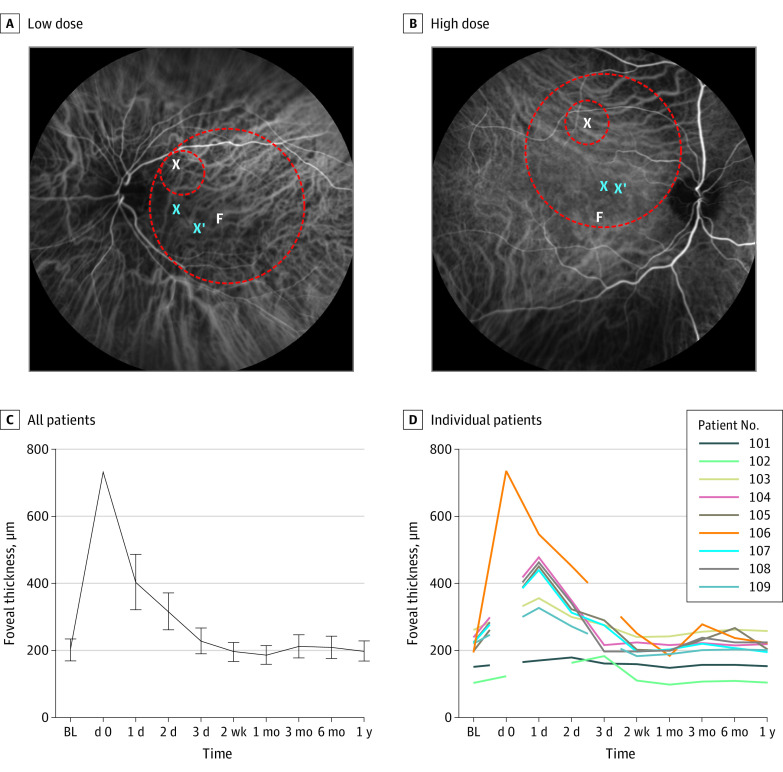
Treatment Area and Association With Foveal Anatomic Features Representative indocyanine green angiographic images from 2 patients at 1 year after treatment with low (1 × 10^10^ vector genomes) (A) and high (1 × 10^11^ vector genomes) (B) doses of adeno-associated virus encoding *CNGA3* gene therapy showing no change in retinal and choroidal perfusion. The target area included the cone photoreceptor–rich macula in all cases (small dashed circles indicate prebleb area; larger circles, extent of full bleb; white X, retinotomy; blue X, preferred retinal locus at baseline; blue X with apostrophe, preferred retinal locus at 1 year after treatment). Foveal thickness as the mean (±2 SDs [error bars]) of all patients (C) and of individual patients (D) over time. Foveal thickness measurement at day 0 (immediately after surgery) was only available for 1 patient. BL indicates baseline.

Nine patients (mean [SD] age, 39.6 [11.9] years; age range, 24-59 years; 8 [89%] male) were included in the study. The patients’ retinas represented different morphologic stages of the disease ranging from near-normal foveal structure to complete foveal atrophy (Table 1 and eFigure 1 in Supplement 2). Baseline visual acuity letter score (approximate Snellen equivalent) ranged from 34 (20/200) to 50 (20/100). Baseline contrast sensitivity log scores ranged from 0.1 to 0.9. All patients received the full designated vector dose without complications. The cone-rich center of the retina was included in the treatment area in all cases. The treatment was not associated with clinically relevant adverse changes in the neuroretinal tissue and/or vasculature (Figure 2). Moreover, all patients maintained their preferred retinal locus inside the treatment area, and the foveal thickness remained unchanged after reattachment (Figure 2 and eFigure 2 in Supplement 2).

**Table 1.** Baseline Demographic and Clinical Characteristics Before and After Gene Therapy

Patient No.	Eye, dose, vector genomes	Disease causing *CNGA3* variants^a^	Before gene therapy^b^			1 Year after gene therapy^b^		
			BCVA		Contrast sensitivity, log*s	BCVA		Contrast sensitivity, log*s (change)
			ETDRS letter score	Snellen score		ETDRS letter score (change)^a^	Snellen score	
101	Right, 1 × 10^10^	c.[1641C>A];[1682G>A]; p.[Phe547Leu(;)Gly561Glu]	41	20/160	0.45	43 (+2)	20/160	0.90 (+0.45)
102	Left, 1 × 10^10^	c.[1641C>A];[(1641C>A)]; p.[Phe547Leu];[(Phe547Leu)]	34	20/200	0.20	38 (+4)	20/200	1.05 (+0.85)
103	Left, 1 × 10^10^	c.[800G>A];[1963C>T]; p.[Gly267Asp];[Gln655*]	35	20/200	0.10	39 (+4)	20/160	0.45 (+0.35)
104	Right, 5 × 10^10^	c.[800G>A];[1963C>T]; p.[Gly267Asp];[Gln655*]	38	20/200	0.60	44 (+6)	20/125	0.65 (+0.05)
105	Left, 5 × 10^10^	c.[847C>T];[847C>T]; p.[Arg283Trp];[Arg283Trp]	49	20/100	0.90	52 (+3)	20/100	1.15 (+0.25)
106	Left, 5 × 10^10^	c.[847C>T];[847C>T]; p.[Arg283Trp];[Arg283Trp]	45	20/125	0.50	46 (+1)	20/125	0.90 (+0.40)
107	Left, 1 × 10^11^	c.[848G>A];[848G>A]; p.[Arg283Gln];[Arg283Gln]	43	20/160	0.60	45 (+2)	20/125	0.85 (+0.45)
108	Left, 1 × 10^11^	c.[940_942delATC];[940_942delATC];p.[Ile314del];[Ile314del]	39	20/160	0.55	40 (+1)	20/160	0.85 (+0.30)
109	Right, 1 × 10^11^	c.[872C>G];[1641C>A]; p.[Thr291Arg];[Phe547Leu]	50	20/100	0.80	53 (+3)	20/100	0.85 (+0.05)

Abbreviations: BCVA, best-corrected visual acuity; ETDRS, Early Treatment Diabetic Retinopathy Study.

^a^ GenBank reference sequence: NM_001298; variant nomenclature according to recommendations of the Human Genome Variation Society.

^b^ Higher values in visual acuity and contrast sensitivity indicate improvement. Patients 103 and 104 are siblings.

### Primary Outcome

The following limits were defined in the trial protocol for ocular safety: (1) loss of 15 or more letters in visual acuity at 1 m and (2) severe, vector-induced intraocular inflammation unresponsive to treatment. Neither of those limits occurred in any of the patients, and no serious adverse event was documented throughout the study (eAppendix in Supplement 2). Two adverse events were deemed possibly related to the study drug: one patient (patient 105) had hyperreflective spots on optical cross-sections (optical coherence tomography) in the treated retina 1 month after treatment; another patient (patient 106) reported symptoms of iridocyclitis 1 month after treatment. Both adverse events quickly resolved with corticosteroid treatment without sequelae.

Analysis of systemic safety end points (vital signs, clinical chemical analysis, and vector biodistribution and shedding) confirmed an excellent safety profile (eAppendix in Supplement 2). Specifically, there was no measurable shedding of AAV8.CNGA3 in lacrimal or nasal fluids, blood, or urine as early as 3 days after surgery (earliest time point measured). Consequently, no changes in antibody titers directed against AAV8 epitopes were observed. Adaptive cellular immune reactions were not observed clinically (apart from the above-mentioned hyperreflective spots potentially signifying recruitment of immune cells and the iridocyclitis). Proliferation and activation assays of peripheral blood mononuclear cell subfractions (CD4^+^ or CD8^+^ T cells, CD19^+^ B cells, and CD56^+^ natural killer cells) (eAppendix in Supplement 2) demonstrated that AAV8.CNGA3 was associated with increased reactivity of CD4^+^ TH1 cells in the medium- and high-dose group after day 90 and with activated B cells. For the mean activation rate of CD4^+^ TH1 cells at days 90 and 180 (eTable 6 in Supplement 2), only 0.6% of cells from the low-dose cohort samples reacted to AAV8.CNGA3 antigen, whereas 1.25% were reactive in the intermediate-dose cohort and 6.6% in the high-dose cohort. Similarly, 6.9% of CD19^+^ B cells were reactive to AAV8.CNGA3 antigen in samples from the low-dose cohort at days 90 and 180 (eTable 6 in the Supplement 2), whereas samples from the intermediate-dose cohort had 11.5% reactive B cells, and 54.7% of B cells from the high-dose cohort were reactive to the AAV8.CNGA3 antigen. The general immune response toward recall antigens was only marginally associated with the stimulation with AAV8 epitopes.

### Secondary Outcomes

**Figure 3.**
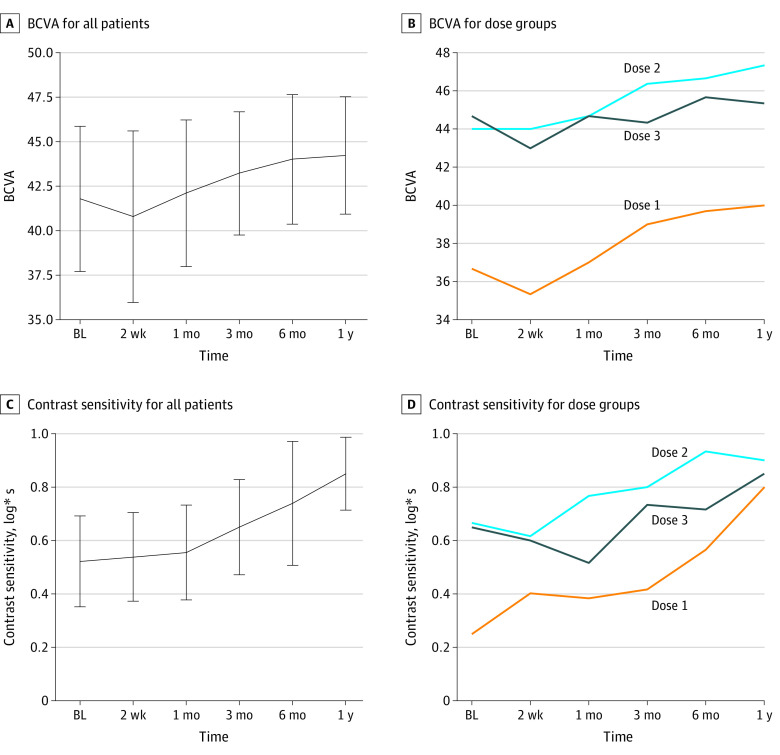
Visual Acuity and Contrast Sensitivity A and B, Improvement of best-corrected visual acuity (BCVA) as the sum score of identified letters on a standardized chart over time. C and D, Increase in contrast sensitivity in patients from baseline over time. All data are presented as mean (±2 SDs [error bars]) of all 9 patients (C) and mean of 3 by dose group (D). Dose 1 was 1 × 10^10^ vector genomes, dose 2 was 5 × 10^10^ vector genomes, and dose 3 was 1 × 10^11^ vector genomes. BL indicates baseline.

Best-corrected visual acuity and contrast sensitivity are well-established end points associated with cone-mediated vision.^21,22^ Both end points showed an improvement (mean best-corrected visual acuity, 2.9 letters (95% CI, 1.65-4.13 letters; *P* = .006, 2-sided *t* test paired samples); mean log contrast sensitivity, 0.33 log (95% CI, 0.14-0.51 log); *P* = .003, 2-sided *t* test paired samples) when comparing the treated eye at baseline vs 1 year after treatment (Figure 3, Table 1, and eFigure 3 in Supplement 2). A potential learning effect cannot be fully ruled out for contrast sensitivity testing, which at 1 year after treatment also improved in the untreated contralateral eye (log*s [±SD] difference from 1 year to baseline, 0.189 [0.236]). However, the measured improvement was more pronounced in the treated eye (log*s [±SD] difference from 1 year to baseline, 0.328 [0.240]). Color vision showed a significant improvement expressed in reduced chromatic discrimination thresholds by 5.3 ± 6.5 1/1000th^2^ L*u*v (*P* = .04, 2-sided *t* test).^23^ Analysis of flicker fusion frequency showed that in 4 of 9 patients, the critical fusion frequency increased by 5 Hz or more, suggesting that the temporal resolution of the retina reached levels suggestive of newly gained cone function (Table 2 and eFigure 3 in Supplement 2). A chromatic pupillography protocol previously shown to discriminate between patients with achromatopsia and healthy control individuals was applied as an objective outcome measure independent of any placebo effect in this open-label study.^24^ Five of 9 patients had a normalization of pupil constrictions after chromatic stimuli 1 year after gene therapy (Table 2).

**Table 2.** Descriptive Analysis and Tests of Significance vs Baseline in Functional Outcomes of the Treated Eye

Outcome	Baseline, mean (SD)	6 mo, mean (SD)	Parametric/nonparametric *P* value	1 y, mean (SD)	Parametric/nonparametric *P* value
BCVA	41.38 (6.39)	43.75 (5.80)	.005/.02	44.00 (5.24)	.01/.02
Contrast sensitivity, 3 m	0.52 (0.26)	0.74 (0.35)	.02/.03	0.85 (0.20)	.003/.008
Flicker fusion frequency	17.44 (5.59)	20.50 (5.89)	.12/.17	18.89 (4.94)	.53/.67
Mean retinal sensitivity	21.14 (2.51)	20.84 (2.29)	.21/.18	21.41 (2.46)	.53/.53
Fixation stability, 4°	58.29 (16.93)	53.22 (15.14)	.33/.40	56.11 (18.13)	.50/.31
CCT ellipse area	0.0249 (0.0053)	0.0202 (0.0060)	.04/.046	0.0213 (0.0063)	.11/.12
Full stimulus threshold	−38.78 (7.03)	−37.00 (4.95)	.58/.77	−36.25 (3.92)	.37/.62
Pupillography					
Relative constriction with red light stimuli	29.3o (6.05)	22.38 (9.46)	.11/.14	15.48 (3.65)	<.001/.008
Baseline diameter with long blue light stimuli	5.37 (1.50)	4.65 (1.20)	.01/.051	4.81 (0.86)	.14/.11

Abbreviations: BCVA, best-corrected visual acuity; CCT, Cambridge Color Test.

Individual values, descriptive statistics, and significance analyses of all tested efficacy measures are summarized in eTable 8 in Supplement 2. With the exception of contrast sensitivity, none of the other efficacy measures reached statistical significance in the untreated control eye (eFigure 4, eFigure 5, and eTable 7 in Supplement 2).

In the assessment of patient-reported outcome measures, a study-specific symptom scale (A3-PRO)^25^ showed 3 improvements (expected false-positive rate of 1). With the use of the validated Visual Function Questionnaire 25,^26^ 6 improvements were observed (expected by chance: 1 or 2). Thus, results from the patient-reported outcome measures (eTable 9 and eTable 10 in Supplement 2) support a functional gain associated with AAV8.CNGA3 gene therapy. Specifically, patients reported improvements in the ability to identify letters and numbers and in color vision.

To combine findings from different clinical end points, standard *z* scores were calculated from 11 key end points that reflected potential cone function (visual acuity, contrast sensitivity, flicker fusion frequency, full-field stimulus threshold [red stimulus], pupil diameter [red stimulus], chromatic contrast sensitivity [ellipse area], mean retinal sensitivity [microperimetry], Visual Function Questionnaire 25 color vision score, and A3-PRO scores for identification of colors, letters, and glare).^27,28^ These individual *z* scores were normalized for baseline and, if necessary, changed in sign to more intuitively show functional gain (*z* score >0) or loss (*z* score <0) by up to 4 SDs over time (eFigure 6 in Supplement 2). All 9 patients demonstrated improvement in cone function at month 12 (mean *z* score >0). Six of 9 patients (67%) had improvement of more than 0.5 SD.

## Discussion

During the past years, academic and pharmaceutical research activities in the field of ocular gene therapy have been intensified and led to marketing authorization of voretigene neparvovec, the first-in-class US Food and Drug Administration–approved gene therapy for inherited retinal diseases caused by variants in the retinal pigment epithelium–expressed gene *RPE65*. Multiple other forms of inherited blindness still remain without treatment options. We report the results of the first, to our knowledge, human clinical study that evaluated gene therapy for type 2 achromatopsia, an inherited retinal disorder caused by variants in *CNGA3* and leading to cone photoreceptor dysfunction. The aim of the study was to test the safety and efficacy of AAV8.CNGA3 in patients with complete achromatopsia due to biallelic variants in *CNGA3*. All primary outcomes substantiate that AAV8.CNGA3 treatment is not associated with substantial safety problems. The adverse event profile was limited and in line with preclinical findings.^11^ These findings suggest that AAV vectors in the eye are recognized by the adaptive immune system, with the immune response peaking 1 month after treatment. This finding has potential implications for patient management. With respect to treatment of the second eye, the question of whether AAV8.CNGA3 should be administered before or after that peak remains. Bennett et al^29^ ruled out immunogenic adverse events when treating the second eye 2 to 3 years after treatment of the first eye with AAV2. In the pivotal phase 3 trial for Leber congenital amaurosis 2, Russell et al^8^ injected the second eye 6 to 18 days after the first with concomitant corticosteroid treatment, with good results. AAV8 as used in our study is a naturally occurring AAV serotype, which has evolved owing to its ability to multiply as a wild-type virus without eliciting a major immune response. Whereas the recombinant version of the vector is replication deficient, the capsid is identical to the naturally occurring serotype in contrast to other engineered vector systems with potentially lower tolerability.^30^

We enrolled adult patients with advanced disease and treated each patient’s worse eye in which a substantial amount of cone photoreceptors had already undergone degeneration. However, achromatopsia has to be considered as a pediatric ophthalmic disorder because the complete lack of cone photoreceptor function is already present at birth and the patient’s visual cortex never experienced and processed cone-derived visual input. Therefore, deprivation amblyopia and altered organization of the visual cortex should be considered as potential limiting factors for efficacy in nonpediatric patients.^31^ The concept that the human visual cortex matures in an interplay between functional input and information processing during a critical period in childhood stems from the investigations of Wiesel and Hubel^32^ in the mammalian visual cortex. This concept suggests that lack of appropriate input from cones will negatively affect the hard wiring of the visual cortex, leading to amblyopia. Amblyopia is best treated before the end of the critical period but is considered essentially irreversible in adults. Although specific exercises can lead to some improvements in amblyopic eyes even in adults,^33^ it was questionable whether plasticity of the visual cortex is preserved throughout life. As such, efficacy of any therapy aiming at restoration of cone photoreceptor function may depend, at least to some extent, on treating before the critical period. Our results in adult patients with achromatopsia indicated some degree of improved visual function in the treated eye, which was consistent throughout different ophthalmologic and patient-reported outcome measures. The clinical effect of this treatment may be most pronounced before the end of the critical period. In the current study, one cannot exclude that the vision outcomes were limited because of (1) advanced morphologic changes (ie, lack of cone photoreceptor outer segments) or (2) persistent amblyopia and a limited plasticity of the visual cortex in the adult patient population.

Taken together, this gene therapy study for achromatopsia found an excellent safety profile associated with subretinally delivered AAV8.CNGA3 and functional improvement in patients 1 year after treatment, although the absence of randomized concurrent control individuals precludes determining a cause-and-effect relationship. Improvement in functional variables, such as visual acuity, contrast sensitivity, and color vision, were noted despite the limited cohort size. In addition, patient-reported outcome measures provided additional support for a possible beneficial treatment effect. For example, the ability to differentiate colors improved after treatment with AAV8.CNGA3, as did measures for vision and identification of letters and numbers—key tasks in everyday life. As such, we found that targeting cone photoreceptors for gene supplementation with recombinant AAV8 can be applied safely and successfully. Of more importance, these data provide evidence that cone photoreceptor function can be gained in adult patients with complete achromatopsia.

### Limitations

The results of this study should be considered within the context of its limitations attributable to its design (open-label, nonrandomized controlled trial), the small patient numbers (9 patients in total and 3 patients per dose group), and the relatively short follow-up period.

## Conclusions

The findings suggest that gene therapy with AAV8.CNGA3 in adults with *CNGA3*-linked achromatopsia is not associated with substantial safety problems and is associated with improvement in vision outcome. Future studies are needed to investigate whether treatment at an earlier age (ie, before closure of the critical period of visual cortex development at the age of approximately 7-8 years and before structural loss of cone photoreceptors) will lead to greater functional benefit because of higher cortical plasticity.

## Notes

Supplement 1. Trial Protocol

Supplement 2. eAppendix. Supplementary Methods and Results eTable 1. Flowchart of Visits and Procedures eTable 2. Classification of Reported Adverse Events eTable 3. Findings in Blood Tests eTable 4. Reporting of Vector Genome Copies Detected Over Time eTable 5. Titers of anti-AAV8 Antibodies Over Time (ELISA) eTable 6. Reaction Profile of Immune Cells Against AAV8 Epitopes and Recall Antigens eTable 7. Significance Analyses in Functional Outcomes of the Control Eye eTable 8. Ophthalmological Outcomes - Tests of Significance eTable 9. Patient Reported Outcomes: Study Specific Scale A3 (PRO) Results eTable 10. Patient Reported Outcomes: VFQ-25 Results eTable 11. Patient Reported Outcomes: BSI Results eFigure 1. Color Photographs, Infrared Images and Virtual Cross Sections of Treated Eyes eFigure 2. Treatment Area and Effect on Foveal Anatomy of Treated Eyes eFigure 3. Visual Acuity, Contrast Sensitivity, Color Contrast Sensitivity, and Temporal Resolution of the Treated Eyes. eFigure 4. Visual Acuity, Contrast Sensitivity, Color Contrast Sensitivity, and Temporal Resolution of the Untreated Control Eyes. eFigure 5. Visual Acuity and Contrast Sensitivity Comparison Between the Treated Eyes and Untreated Control Eyes eFigure 6. Z Score Normalization of Efficacy Endpoints
