# Supplementary material for: Safety and Vision Outcomes of Subretinal Gene Therapy Targeting Cone Photoreceptors in Achromatopsia: A Nonrandomized Controlled Trial
Source: JAMA Ophthalmol. 2020 Apr 30;138(6):1–9. doi: 10.1001/jamaophthalmol.2020.1032 (PMC7193523; doi:10.1001/jamaophthalmol.2020.1032)
Supplement: Supplement 1. — Trial Protocol [file jamaophthalmol-138-643-s001.pdf]

SUPPLEMENT – Clinical Trial Protocol

Table of Contents **... 1**

1. Clinical trial protocol
a. Original protocol .... 2 - 69
b. Final protocol .... 70 - 139
c. Summary of changes 140
2. Statistical analysis plan
a. Original statistical analysis plan (chapter 10 of original protocol) 52 - 54
b. Final statistical analysis plan (chapter 10 of final protocol) ..... 123 - 124
c. Summary of changes 140

Safety and efficacy of a single subretinal injection of rAAV.hCNGA3 in patients with CNGA3-linked achromatopsia investigated in an exploratory, dose-escalation trial

Version 1.0

Status: Final

Protocol No: RDC-CNGA3-01
EudraCT No: 2014-001874-32
Reference No EC Tübingen:

Date: 09 February 2015

Table of Contents

I Amendment history....4
II Abbreviations 5
III Roles in the study.... 8
IV Synopsis in English....10
V Investigator agreement....12
VI Synopsis in German....13
VII Flowchart of visits and procedures....15

1. Introduction.... 17
1.1.1. Clinical characteristics and genetics of achromatopsia 17
1.2. Rationale of the Trial 19
1.3. The rAAV.hCNGA3 Vector for the use in the human trial 21
1.3.1. Vector Production.... 22
1.3.2. Preclinical testing in rodents.... 23
1.3.3. Preclinical testing in non-human primates 23
2. Aim of the Trial 24
2.1. Primary Aim.... 25
2.2. Secondary Aims 25
3. Study Plan 25
3.1. Study Design 25
3.2. Study Duration.... 27
3.3. Participating Sites.... 27
3.4. Number of enrolled patients.... 27
4. Study Population 27
4.1. Characterization of Patient Population.... 27
4.2. Inclusion Criteria (Study Eye) 28
4.3. Exclusion Criteria.... 28
5. Patient Inclusion 29
5.1. Time plan for inclusion 29
5.2. Mode of assignment of patients to treatment.... 29
6. Intervention.... 29
6.1. Treatment 29
6.2. Concomitant medication 30
7. Endpoints for Safety and Efficacy of a single subretinal injection of rAAV.hCNGA3 genome.... 31
7.1. Efficacy 31
7.2. Safety 31
7.2.1. Ocular and systemic safety 31
7.2.2. Data Monitoring Committee 32
7.2.3. Definitions.... 32
7.3. Reporting Procedures for all Adverse Events.... 33
7.3.1. Reporting Procedures for Serious Adverse Events 34

7.4. Rules for discontinuation of the trial 34
7.5. Risk-Benefit Considerations 35
7.6. Risks for the Environment 37
8. Examinations 38
8.1. Screening Examinations 38
8.2. Trial Examinations 39
8.3. Follow-up Examinations 44
8.4. Non-ophthalmological and ophthalmological Procedures 46
8.4.1. Medical / surgical history 46
8.4.2. Ocular / surgical history 47
8.4.3. Body Mass Index 47
8.4.4. Vital signs 47
8.4.5. Urine pregnancy test 47
8.4.6. Hematology, basic chemistry and urine analysis 47
8.4.7. CRP, Immunoglobulines 48
8.4.8. Immunopathology 48
8.4.9. PCR of rAAV8 genome 48
8.4.10. Best corrected visual acuity 48
8.4.11. Basic ophthalmological examination 48
8.4.12. Contrast sensitivity (Pelli Robson Charts) 48
8.4.13. Flicker Fusion Frequency 48
8.4.14. Colour constancy 49
8.4.15. Anomaloscopy 49
8.4.16. Cambridge Colour Test 49
8.4.17. Infrared-Video-Pupillography 49
8.4.18. Microperimetry 49
8.4.19. Fundoscopy 50
8.4.20. Dark adaptation (DA) 50
8.4.21. Ganzfeld electroretinogram (ERG) 50
8.4.22. Spectral domain optical coherence tomography (SD-OCT) 50
8.4.23. Fundus autofluorescence (FAF) 50
8.4.24. Fundus photography (FP) 50
8.4.25. Angiography 51
8.4.26. Topical steroids and antibiotics 51
8.4.27. Systemic steroids 51
8.4.28. Subretinal injection of rAAV8.hCNGA3 vector 51
8.4.29. VFQ25 51
8.4.30. Psychiatric examination 52
8.4.31. Brief symptom inventory (BSI) 52
8.4.32. Psychological counselling 52
8.4.33. Study specific scale, A3-PRO 52
8.4.34. Adverse event (AE) recording 52
8.4.35. Concomitant medication 52
9. Documentation of Trial Data 52
10. Biometrical Planning and Analysis 53

10.1. Trial Design 53
10.2. Sample Size Issues 53
10.3. Definition for Study Groups 53
10.4. Statistical Analysis 54
10.5. Interim Analyses 54
11. Anticonception Rules and Pregnancy 54
13. Conditions for Amendments 55
14. Ethical and Regulatory Aspects 56
15. References 57
16. Appendices 64

I. Amendment history

Number	Protocol Version	Date	Summary of changes

II. Abbreviations

µm	micrometer
A3-PRO	intervention-specific scale assessing patient reported outcome
AAV	adeno-associated viral
AAV-5	virus capsid
AAV5-S	virus capsid
AAV8	virus capsid serotype for expression of transgenes in photoreceptors
AAV8-hCNGA3	AAV8-pseudotyped hCNGA3 viral particles
ACHM	congenital achromatopsia
AE	adverse event
AIS	electronic patient file of the Centre for Ophthalmology
ANSM	Agence nationale de sécurité du médicament et des produits de santé
AO-SLO	applied optics scanning laser ophthalmoscopy
AR	adverse reaction
ARR3	promoter
BCA	bicinchoninic acid
BCVA	best corrected visual acuity
BGHpA	bovine growth hormone polyadenylation site
bp	base pair
BSI	Brief Symptom Inventory
c19q13.3	gene locus
cGMP	cyclic guanosine monophosphate
cGMP-PDE	guanosine monophosphate-gene
CNG	cyclic nucleotide gated
CNGA3	cyclic nucleotide gated channel alpha 3 gene
$CNGA3^{-/-}$	CNGA3 knockout
CRF	case report form
CRO	clinical research organization
CRP	c-reactive protein
CsCl	cesium chloride
D	day
de-sat.	de-saturated
DMC	data monitoring committee
DMEM	Dulbecco's Modified Eagle's Medium
E. coli	Escherichia coli
e. g.	for example
E1A	protein
EC	Ethics Committee
EFS	Etablissement Français du Sang
ELISA	enzyme-linked immunosorbent assay
EMA	European Medicines Agency
ERG	electroretinography

196	ESR	erythrocyte sedimentation rate
197	ETDRS	Early Treatment Diabetic Retinopathy Study
198	FAF	fundus autofluorescence
199	FDA	Food and Drug Administration
200	FBS	fetal bovine serum
201	FP	fundus fotography
202	ff-ERG	full field electroretinography
203	FST	dark adaptation test
204	G	gauge
205	GCP	good clinical practice
206	GMP	guanosine monophosphate
207	GMP	good manufacturing practice
208	GNAT2	gene
209	hArr3	human cone arrestin promoter
210	hCNGA3	gene involved in rod monochromacy
211	HEK293	human embryonic kidney cell line
212	HRD	inherited retinal dystrophies
213	ICA	infectious center assay
214		indocyanine green
215	IgG	antibody
216	IgM	antibody
217	INL	inner nuclear layer
218	ip/ml	infectious particles/milliliter
219	IR	infrared
220	ITRs	inverted terminal repeats
221	ITT	intent to treat
222	kg	kilogram
223	LCA	Lebers congenital amaurosis
224	m	meter
225	M	month
226	ml	milliliter
227	MP	microperimetry
228	NCT00481546	clinical trial
229	NCT00516477	clinical trial
230	NCT00643747	clinical trial
231	NCT01461213	clinical trial
232	NHP	non-humane primate
233	OCT	optical coherence tomography
234	ONL	outer nuclear layer
235	OPN1MW opsin	promoter
236	OPN1SW opsin	promoter
237	OS	outer segments
238	PCR	polymerase chain reaction
239	PDE6C	gene
240	PDE6H	gene

pDP8	gene
pDP8-KanR	AAV8 *trans* plasmid
PEG	polyethylene glycol
PEI	Paul Ehrlich Institute (Regulatory Authority)
PI	principle investigator
PNA	peanut agglutinin
PP	polypropylene
PP	per protocol
PR charts	Pelli Robson charts
PRO	patient reported outcome
pSub-hArr3-hCNGA3-WPREm-KanR	AAV *cis* plasmid
qPCR	quantitative polymerase chain reaction
rAAV	recombinant adeno-associated viral
rAAV.hCNGA3	vector
REP1	gene
RPE65	gene
SAE	serious adverse event
SAR	serious adverse reaction
Sat.	saturated
sdOCT	spectral domaine optical coherence tomography
SDS-PAGE	sodium dodecylsulphate polyacrylamide gelelectrophoresis
SSSOI	Balanced Sterile Saline Solution for Intraocular Irrigation
SUSAR	suspected unexpected serious adverse reaction
TC	telephone conference
TCID50	tissue culture infection dose
TE	treated eye
TFF	tangential flow filtration
UE	untreated eye
UKT	Universitaetsklinikum Tuebingen
USP	U.S. Pharmacopeial Convention
VA	visual acuity
VFQ	visual function questionnaire
vg	vector genome
vgp	vector genome particles
vol/vol	volume percent concentration
vp/ml	virus particle/milliliter
WPRE	woodchuck hepatitis virus post-transcript. regulatory element
wt	wild type
y	year

III. Roles in the study

Principal Investigator

PD Dr. med. M. Dominik Fischer
Centre for Ophthalmology, University Tuebingen
Schleichstr 12-16
D-72076 Tuebingen
Phone ++49 7071 298 37 21
Fax ++49 7071/29 50 21
Email: dominik.fischer@med.uni-tuebingen.de

Deputy Principal Investigator

Prof. Dr. med. Karl Ulrich Bartz-Schmidt
Schleichstr 12-16
D-72076 Tuebingen
Phone ++49 7071 298 4001
Fax ++49 7071 29 52 15
Email: karl-ulrich.bartz-schmidt@med.uni-tuebingen.de

Responsible Ethics Committee

Ethics Committee of the Medical Faculty of the University of Tuebingen
Gartenstr. 47
D-72074 Tuebingen
Tel.: ++49 70 71/ 29- 7 76 61
Fax: ++49 70 71/ 29-59 65
ethik.kommission@med.uni-tuebingen.de

CRO

STZ *eyetrial* at the Centre for Ophthalmology, University Tuebingen
Schleichstr. 12-16
D-72076 Tübingen
Phone ++49 7071/298 48 - 98 (Prof. Wilhelm); -94 (Dr. Peters)
Fax ++49 7071/29 50 21
Email: barbara.wilhelm@stz-eyetrial.de; tobias.peters@stz-eyetrial.de

IV. Synopsis

Title: Safety and efficacy of a single subretinal injection of rAAV.hCNGA3 in patients with CNGA3-linked achromatopsia investigated in an exploratory, dose-escalation trial

Phase: I/II

Indication: CNGA3-linked achromatopsia

Aim: To proof the safety and efficacy of rAAV.hCNGA3 in patients with achromatopsia

Study design: open, mono-center trial with fellow-eye comparison

Study Population:

Inclusion Criteria (Study Eye)

- clinical diagnosis of achromatopsia
- ≥ 18 years of age
- confirmed mutation in CNGA3
- BCVA ≥ 20/400; first cohort: BCVA ≤ 20/160
- area of residual outer nuclear layer thickness in OCT scans of posterior pole
- ability to understand and willingness to consent to study protocol

Exclusion Criteria

- additional interfering eye conditions (e.g. uveitis, advanced cataract) in the study eye
- systemic conditions (e.g. coronary heart disease, autoimmune disorders) which may affect study participation or outcome measures
- current or recent (30 days) participation in other study and/or administration of biologic agent
- gross asymmetry of ocular condition/lack of control eye
- recent (6 months) ocular surgery, intravitreal or subretinal implantation of a medical device
- known sensitivity to any compound used in the study
- contraindications to systemic immunosuppression
- subject/partner of childbearing potential unwilling to use adequate contraception for four months
- nursing or pregnant women
- any other cause that, in the investigator‘s opinion, renders potential subjects not suitable for the study

Patient Number: Nine patients will be assigned to three cohorts of three patients respectively.

Treatment:
Each cohort will receive a different, increasing dosage of viral vector genome particles. Three cohorts of patients are planned with low, medium and high dose administration levels up to 1x10e11 of rAAV8 incorporating the therapeutic expression cassette.
After standard three-port 23G pars plana vitrectomy, balanced salt solution will be used to induce a localized primary retinal detachment in a controlled fashion. Vector solution will be applied using disposable 41G extendible subretinal injection needles within a standard 23G body to fit the port system. Administration will be unilateral (worse eye) in each patient.

Primary Endpoint: Safety as the primary endpoint will be assessed by clinical examination of ocular inflammation (slit lamp, fundus biomicroscopy, angiography, perimetry or electrophysiology). Systemic safety will be assessed by vital signs, routine clinical chemistry testing (including CRP, ESR) and full/differential blood counts. Immunopathology essays will include specific enzyme-linked immunosorbent assays for humoral antibodies against rAAV8 capsid protein and/or CNGA3 gene product and specific enzyme-linked immunosorbent spot assays to monitor cellular immune reactivity against rAAV8 capsid protein and/or CNGA3 gene product. Biodistribution will be monitored by polymerase chain reaction studies on rAAV8 genome in blood and urine.

Statistical Methods: AEs and SAEs will be documented using line listings and tabulations (MetraCad code will be applied). Descriptive analysis will include line charts for individual patients. Standard errors and means will also be displayed using line charts. This will be done for the raw measurements and for the difference between treated and untreated control eye within each patient.
Descriptive parameters will be given for the full cohort (n=9) but not for the sub-cohorts (n=3). Additionally correlation analysis will be performed in a purely descriptive manner for the comparison of subjective and objective measurements. For each subject and measurement, the delta of the value for the interventional eye minus the mean of control eye values over time will be determined. In scatter plots for pairs of variables (measurements pooled over subjects) the association between these deltas will be displayed.

Time Schedule: Start of trial March 2015, end of recruitment September 2016, end of trial September 2017, duration of trial per patient: one year with four years of follow-up.

V.

V. Investigator Agreement

I have read the clinical study protocol entitled: "Safety and efficacy of a single subretinal injection of rAAV.hCNGA3 in patients with CNGA3-linked achromatopsia investigated in an exploratory, dose-escalation trial" Version 1.0 and I have verified that it contains all necessary information for conducting the study.

I hereby confirm that:

- I have carefully read and understood this clinical study protocol
- My staff and I will conduct the study according to the study protocol and will comply with its requirements, including ethical and safety considerations.

I understand that, should the Sponsor decide to prematurely terminate or suspend the study for whatever reason, such decision will be communicated to me in writing. Conversely, if I decide to withdraw from execution of the study, I will immediately communicate such a decision to the Sponsor.

I agree not to publish any part of the results of the study carried out under this clinical study protocol without consulting the sponsor.

Principal Investigator (PI) M.D. FISCHER

Date 10/2/2015

Signature

Deputy Principal Investigator BARTZ-SCHMIDT

Date 9.2.2015

Signature

Synopsis in German / Deutsche Prüfplanzusammenfassung

Titel: Sicherheit und Wirksamkeit einer einzelnen subretinalen Injektion von rAAV.hCNGA3 bei Patienten mit CNGA3-chromosomaler Achromatopsie, untersucht in einer explorativen Studie mit ansteigender Dosierung.

Phase: I/II

Indikation: CNGA3-chromosomale Achromatopsie

Ziel: Nachweis der Sicherheit und Wirksamkeit von rAAV.hCNGA3 in Patienten mit Achromatopsie

Studiendesign: Offene monozentrische Studie mit Vergleich zum Partnerauge

Studienpopulation:
Einschlusskriterien (Studienauge)

- Klinisch diagnostizierte Achromatopsie
- Alter ≥18 Jahre
- Bestätigte Mutation in CNGA3
- bestkorrigierte Sehschärfe ≥ 20/400; Erste Kohorte: ≤ 20/160
- Bereich der verbliebenen äußeren nuklearen Schichtdicke im OCT-Scan des hinteren Pols
- Fähigkeit das Studienprotokoll zu verstehen und Bereitschaft einzuwilligen

Ausschlusskriterien

- Zusätzliche Augenkrankheiten (z.B. Uveitis, fortgeschrittener Katarakt) im Studienauge
- Störende systemische Krankheiten (z.B. Koronare Herzkrankheiten, Autoimmun-erkrankungen), die für die Studienteilnahme als wesentlich erachtet werden
- Aktuelle oder kürzliche (30 Tage) Teilnahme an anderen Studien und/oder Einnahme biologischer Agenzien
- Grobe Asymmetrie der Achromatopsie / Ausfall des Partnerauges
- Kürzliche (6 Monate) Augenoperation, intravitreale oder subretinale Implantation eines Medizinproduktes
- Bekannte Unverträglichkeit gegenüber einer in der Studie eingesetzten Substanz
- Kontraindikationen zur systemischen Immunsuppression
- Gebärfähige Patienten oder Partner, die nicht zur Empfängnisverhütung für vier Monate bereit sind
- Stillende oder schwangere Frauen
- Andere Gründe, die nach Meinung des Prüfers gegen die Eignung eines Probanden sprechen

Anzahl der Patienten: Neun Patienten werden 3 Kohorten mit je 3 Patienten zugeordnet.

Behandlung:
Jede Kohorte wird eine andere Dosierung des viralen Vektor-genom-Partikels erhalten. Drei Patientenkohorten sind geplant mit niedriger, mittlerer und hoher Dosierung bis zu 1x10e11vgp des rAAV8.
Nach Standard 3-Port 23G pars-plana-Vitrektomie wird Salzlösung eingesetzt, um eine kontrollierte, lokale, primäre Netzhautablösung zu induzieren. Die Vektorlösung wird mittels 41G erweiterbarer subretinaler Einwegs-Injektionsnadel mit einem Standard 23G Korpus, passend zum Port-System, eingebracht. Die Gabe wird bei jedem Patient unilateral vorgenommen (schlechteres Auge).

Primärer Endpunkt:
Die Sicherheit als primärer Endpunkt wird durch klinische Untersuchung der okulären Entzündung (Spaltlampe, Funduskopie, Angiographie, Perimetrie oder Elektrophysiologie) beurteilt. Systemische Sicherheit wird durch Vitalparameter, routinemäßige klinische Chemie (einschließlich CRP, BSG) und Voll-/Differential-blutbild beurteilt. Als immunpathologisches Nachweisverfahren dient das spezifische antikörpergekoppelte Nachweisverfahren ELISA (enzyme-linked immunosorbent assay) für humorale Antikörper gegen das rAAV8 Capsid-Protein, um die zelluläre Immunantwort gegen das rAAV8 Capsid-Protein zu überwachen. Die Biodistribution wird durch Polymerase-Kettenreaktion (PCR)-Untersuchungen über das rAAV8 Genom in Blut und Urin überwacht werden.

Statistische Methoden:
Deskriptive Statistik ist geplant.

Zeitplan: Studienstart März 2015, Ende der Rekrutierung September 2016, Studienende September 2017, Dauer der Studie pro Patient: ein Jahr mit anschließend vier Jahren Nachbeobachtungsphase.

VI. Flowchart of Visits and Procedures

See next page

Flowchart of Visits and Procedures CNGA3 Trial

	**Visit (nr)**	**1**	**2**	**3**	**4**	**5**	**6**	**7**	**8**	**9**	**10**	**Fup 1**	**Fup2**	**Fup3**	**Fup 4**
	Date	Screening	D 0	D1	D2	D3	D14	D30	D90	D180	D365, Close out / ET	M24	M36	M48	M60
	Time window	day -30 to -2				± 1 day	± 3 days	± 5 days	± 7 days	± 7 days	± 14 days	± 1 month	± 1 month	± 1 month	± 1 month
	Procedure														
1	Medical /surgical/ history	x													
2	Ocular / surgical/ history	x													
3	BMI	x									x				
4	Vital signs	x	x	o	o	x	o	o	o	o	x	o	o	o	o
5	Urine pregnancy test	x	x				x	x	x	x	x				
6	Hematology/basic chemistry/urine analysis	x	o	o	o	x	x	o	o	x	x	x	x	x	x
7	CRP, IgG, IgM	x			x	x	x	x	o	o	o	o	o	o	o
8	Immunopathology [1]	x						x	x	o	o	o	o	o	o
9	PCR of rAAV8 genome	x				x	x	o	o	o	o	o	o	o	o
10	Best corrected visual acuity	x					x	x	x	x	x	x	x	x	x
11	Basic ophthalmological exam (miosis)	x	x	x	x	x	x	x	x	x	x	x	x	x	x
12	Contrast sensitivity (PR charts)	x					x	x	x	x	x	x	x	x	x
13	Flicker fusion frequency	x						x	x	x	x				
14	Colour constancy (chromatic adaptation)	x						o	o	x	x				
15	Anomaloscopy	x					x	x	x	x	x	o	o	o	o
16	Cambridge Colour Test	x					x	x	x	x	x	o	o	o	o
17	IR-Video-Pupillography	x						x	x	x	x	o	o	o	o
18	MP-1 Microperimetry (20°)	x						x	x	x	x	x	x	x	x
19	Fundoscopy (mydriasis)	x	x	x	x	x	x	x	x	x	x	x	x	x	x
20	Dark adaptation test	x						x	x	x	x	o	o	o	o
21	ff-ERG (scotopic and photopic)	x						x		o	x	o	o	o	o
22	sdOCT	x	o	o	o	o	x	x	o	o	x	x	x	x	x
23	Fundus autofluorescence	x					x	x	o	o	x	x	x	x	x
24	Fundus photography	x					x	x	x	o	x	x	x	x	x
25	Angiography	x					o	o	x	o	x	o	o	o	o
26	Systemic steroids [2]	x	x	x	x	x									
27	Topical steroids and antibiotics [3]	x	x	x	x	x	x	x							
28	Subretinal injection of rAAV8 vector		x												
29	VFQ25	x						x		x	x	o	o	o	o
30	Psychiatric examination	x													
31	Brief Symptom Inventory (BSI)	x					x	x	x	x	x				
32	Psychological counselling [4]		x	x	x	x	x	x	x	x	x				
33	study specific scale, A3-PRO						x	x	x	x	x	x	x	x	x
34	Adverse event recording		x	x	x	x	x	x	x	x	x	x	x	x	x
35	Concomitant medication	x	x	x	x	x	x	x	x	x	x	x	x	x	x

O: optional, if control deemed necessary by investigator

1 including but not limited to: Anti-AAV8 capsid ELISA, ELISPOT (anti-CNGA3)
2 Systemic steroids will be given orally 1.0mg/kg for appr. three weeks starting at day -1 and then tapered off after day 19 as deemed appropriate by the investigator (e.g. 40/30/20/15/10mg).
3 Moxifloxacin eye drops 0.5% 4x/d & 0.1% dexamethasone gel 4x/d for 21d starting at day -1
4 at patient's need and discretion

1. Introduction

1.1.Background

This is an investigator-initiated trial fully funded by the Tistou and Charlotte Kerstan Stiftung, Germany. The trial is part of the RD-Cure project, a joint project of the Universities Tübingen and Munich, Germany, addressing the development of the first ophthalmological gene therapy approaches in CNGA3-linked achromatopsia and – at a later stage – PDE6A-linked retinitis pigmentosa in Germany. The RD-Cure project including its clinical trials is under the continuous supervision of an international scientific Reviewer and Advisory Board (RAB).
Sponsor of the Trial presented here are the University Hospitals Tübingen, Germany.

1.1.1. Clinical characteristics and genetics of achromatopsia

Congenital achromatopsia (ACHM) or rod monochromatism is an autosomal recessively inherited congenital defect. Individuals can have a complete form of the disease, with a total lack of function of all three types of cones in the retina, or an incomplete form, in which one or more cone types may be partially functioning (Blackwell and Blackwell, 1961; Pokorny et al., 1982)
ACHM belongs to the group of inherited retinal dystrophies (HRD), a highly heterogeneous group of rare ocular diseases, with impaired light sensitivity and/or signal transmission within the neuroretina. Retinal dystrophies in general are a major cause of visual disability and legal blindness in the working population: The prevalence of general HRD in Danish children is 1.3 per 10,000 (Bertelsen et al., 2013), in Northern France the prevalence is 6.7 per 10,000 (Puech et al., 1961). Patients with HRD are more likely to be registered as blind at a much younger age, and have a longer duration of blindness and visual impairment, which significantly decreases their quality of life (Prokofyeva et al., 2009). HRD thus have considerable socio-economic impact (Flanagan et al., 2003).
Clinical Symptoms of ACHM are characterized by
Achromatopsia: A lack of the perception of color.
Amblyopia: A neural condition causing reduced visual acuity without any morphological cause.
Hemeralopia: Reduced visual capacity in bright light causing debilitating glare.
Nystagmus: A pathological condition involving an uncontrolled oscillatory movement of the eyes.
Photophobia: The avoidance of bright light by those suffering from hemeralopia.
ACHM is characterized by lack of cone photoreceptor function. Due to the complete unresponsiveness of cones (in contrast to common forms of color blindness, in which changes in expression of opsin genes merely affect spectral sensitivity but not the physiology of photoreceptors), ACHM is considered a severe ocular disease with serious consequences for vision as high-acuity central vision mediated by densely packed cones in the fovea is missing.

The disease was initially thought to be non-progressive because cone function appeared to be absent from birth, but recent morphological in vivo studies established ACHM to be a slowly progressing degenerative retinal disease, and foveolar cone photoreceptor loss can be observed in most adult ACHM patients (Thiadens et al., 2010; Aboshiha et al., 2014; Greenberg et al., 2014; Sundaram et al., 2014; Yang et al., 2014)

The key characteristics of ACHM are therefore very poor visual acuity (± 0.1), severe photophobia at normal daylight conditions and complete color blindness that is accompanied by a pendular nystagmus. In addition there is a significant incidence of hyperopia in ACHM patients. Clinical testing by functional and morphological methods can reveal the complete lack of central cones.

Mutations in five genes are implicated in ACHM (see Table 1), which explains around 93% of cases (Roosing et al., 2014). The most common are mutations in one of the two genes encoding the alpha and beta subunit of the cone cyclic nucleotide-gated (CNG) channel subunits, CNGA3 (found in about 25-28% of cases: (Kohl et al., 1998; Kohl et al., 2000) and CNGB3 (50% of cases, (Sundin et al., 2000; Kohl et al., 2005). CNG channels are involved in transmitting information about vision and smell from sensory cells to the brain. Less common mutations are the GNAT2 gene (1-2% of cases (Aligianis et al., 2002; Kohl et al., 2002), which provides instructions for making the alpha subunit of transducin, that plays an essential role in transmitting visual signals from cones to the brain, and the PDE6C gene (1-2% of cases, (Chang et al., 2009; Thiadens et al., 2009; Thiadens et al., 2010; Sundaram et al., 2014), which encodes the alpha-prime subunit of cyclic guanosine monophosphate (cGMP) phosphodiesterase. Extremely rare (0.5% of cases) is a mutation in the PDE6H gene that participates in processes of transmission and amplification of the visual signal (Chang et al., 2009; Thiadens et al., 2009; Kohl et al., 2012). cGMP-PDEs are the effector molecules in G-protein-mediated phototransduction in vertebrate rods and cones

Table 1. Genetic Variants of Achromatopsia

Gene	Populations	Location	Incidence
CNGB3	Pingelapese and Irish	8q21	50%
CNGA3	Danish, Jews of Iraq, Iran and Morocco	2q11	25%
GNAT2	Europeans of Italy and Denmark	1q13	2%
PDE6C	Not determined	10q24	2%
PDE6H	Europeans of the Netherlands and Belgium	12q13	0.5%

Currently there is no effective and approved treatment available for ACHM.

1.1.2. Current experience with gene therapeutic treatment of hereditary degenerative retinal diseases

Treatments for other hereditary retinal degenerative diseases (Lebers congenital amaurosis, LCA; chorioideremia) are currently administered in clinical trials with positive results. Three landmark clinical trials (NCT00516477, NCT00481546 and NCT00643747) have first shown safety and evidence of efficacy using recombinant adeno-associated viral (rAAV) vectored gene therapy in the eye (Bainbridge et al., 2008; Hauswirth et al., 2008; Maguire et al., 2008; Jacobson et al., 2012). In these three studies, nine patients with a different form of retinal degeneration caused by mutations in the gene encoding RPE65 were treated by subretinal injections of rAAV2/2 vector solutions without a single serious adverse event (SAE). A subset of these patients even demonstrated some degree of improved visual function in the untreated eyes. A phase 1 trial with 12 LCA patients additionally confirms the safety and efficacy of the method, also after re-administration to the second eye (Maguire et al., 2009; Simonelli et al., 2010; Bennett et al., 2012). A follow up in 5 patients after 3 years additionally shows positive results (Testa et al., 2013). However, although improved vision is reported, the underlying retinal degeneration may continue (Cideciyan et al., 2013).

A fourth trial (NCT01461213) is currently on-going aiming to transduce retinal pigment epithelial and photoreceptor cells by subretinal delivery of rAAV2/2 vector solution in patients with choroideremia, caused by mutations in REP1. No SAE has been reported so far and accumulating evidence suggests a dose dependent therapeutic effect in the treated vs. untreated eye (Barnard et al., 2014; Huckfeldt and Bennett, 2014; Maclaren et al., 2014). Further trials are planned.

The knowledge and experience gained in these trials mentioned above have been strongly encouraging for the planned novel intervention in ACHM.

1.2. Rationale of the Trial

The goal of the CNGA3 study is to develop, produce and investigate a recombinant adeno-associated viral (AAV) gene transfer vector for the curative therapy of CNGA3-linked inherited retinal dystrophies in patients, in order to counteract their progression and impairment of visual function leading to disability.

CNGA3 encodes the alpha subunit of the cone photoreceptor cyclic nucleotide-gated cation channel and mutations in this gene have been associated with complete and incomplete achromatopsia (Kohl et al., 1998; Wissinger et al., 2001; Burgueno-Montanes et al., 2014; Yang et al., 2014).

Recently, the curative potential of gene therapy in the CNGA3-deficient animal model has been demonstrated (Michalakis et al., 2010). The primary therapeutic effect was confirmed on the level of retinal morphology and histology as well as on the functional level. Regarding function a variety of methods were applied, including ERG and ganglion cell recordings, and tests of vision-guided behavior. The treatment in the animal model was exceptionally robust regarding the choice of specific promoters (OPN1SW opsin, OPN1MW opsin, ARR3) and virus capsides (AAV-5, AAV-8) and

worked in all combinations. It was also robust despite the technically challenging subretinal injection procedure in the small mouse eye; 9 out of 10 mice showed substantial functional rescue. Finally, it was found that because trafficking to cone outer segments is limited to heterotetrameric CNG channels with correct stoichiometry, the genetically unaffected amount of endogenous CNGB3 subunits determines the amount of channels in the active compartment of the cell, the outer segment, ensuring the correct dosage.

The principal proof-of-concept for restoration of cone-mediated vision by AAV5-mediated retinal gene replacement therapy in CNGA3$^{-/-}$ mice (Figure 1) has been described (Michalakis et al., 2010) and is illustrated in the following.

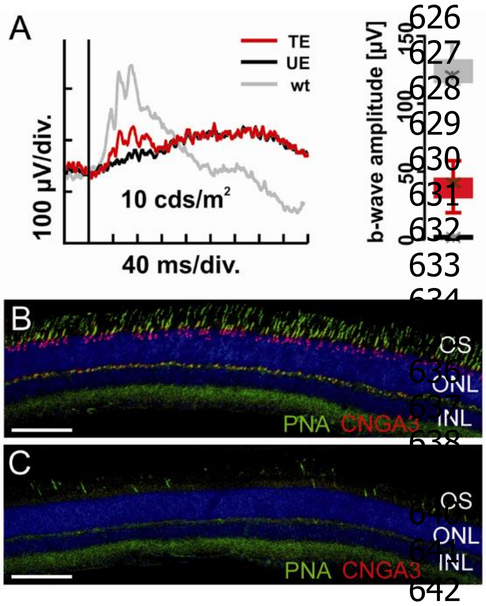

Figure 1. *Restoration of cone-mediated ERG and delay of cone degeneration in treated CNGA3$^{-/-}$ cones. (A) Single flash photopic ERG shows substantial restoration of cone system function (traces left, corresponding box plot bottom right). Treated eye (TE), untreated eye (UE), and wildtype eye (wt). (B and C) Retinal slices of age-matched treated (B) and untreated (C) CNGA3$^{-/-}$ mice, stained with the cone marker peanut agglutinin (PNA, green) and anti-CNGA3 (red) reveals the preservation of a high number of cones after treatment (B). Scale bars mark 100 µm. Nuclei are stained with Hoechst dye (blue). Abbrevations: INL, inner nuclear layer, ONL, outer nuclear layer, OS, (photoreceptor) outer segments.*

Different AAV serotypes with distinct preferential tropism for certain retinal cell types are available, and together with cell-type specific promoters provide exceptionally versatile and effective tools for gene therapy approaches targeting photoreceptors (see e.g. (Liu et al., 2011; Asokan et al., 2012; McClements and MacLaren, 2013)). In recent years AAV5 and especially AAV8 have evolved as the preferred serotypes for photoreceptor-specific gene expression in several animal models (Allocca et al., 2007; Lebherz et al., 2008; Tan et al., 2009; Mussolino et al., 2011; Vandenberghe et al., 2011; Smith et al., 2012). In subsequent studies Michalakis et al. confirmed a high efficacy for AAV8-mediated retinal gene replacement therapy in CNGA3$^{-/-}$ mice (Michelfelder et al., 2011). In addition, it was found that AAV8-based vectors resulted in a faster and slightly more robust restoration of visual function compared to AAV5 (Figure 2). This further argues for AAV8 as the most effective serotype for expression of transgenes in photoreceptors. The ocular delivery of AAV8 is novel in this trial, but the same vector type has been applied systemically. A study that aimed at the correction of Hemophilia B tested the systemic delivery of very high AAV8 titers and no serious adverse effects have been reported (Nathwani et al., 2011)

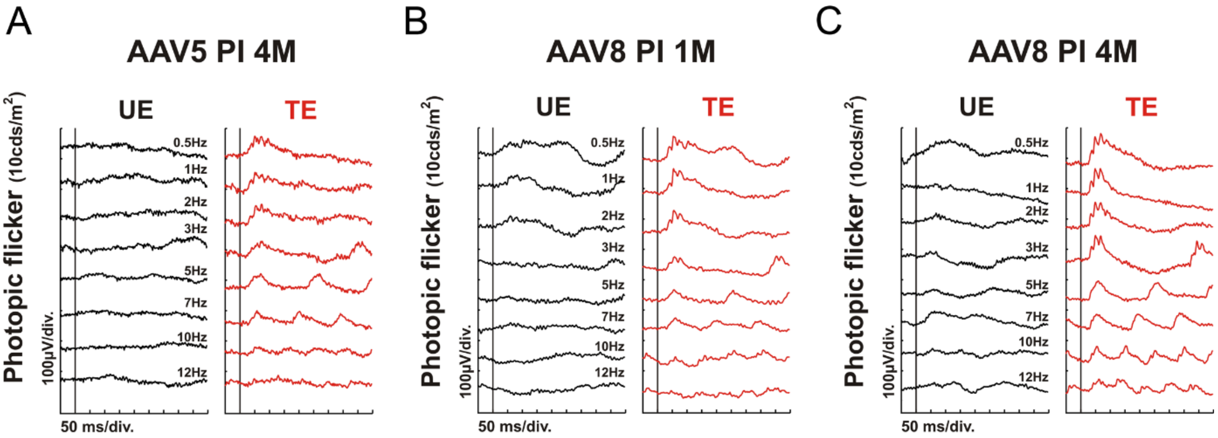

D

	4 weeks PI	6 weeks PI	8 weeks PI	3M PI	4M PI
AAV5-S opsin-CNGA3	X	X	✓	✓	✓
AAV8-S opsin-CNGA3	✓	✓	✓	✓	✓

Figure 2. Effect of AAV serotype on therapy onset. Representative photopic flicker series to compare the treatment efficacy of AAV5- and AAV8- mediated CNGA3 gene therapy in CNGA3$^{-/-}$ mice. Restoration of cone-specific light responses (A) by AAV5-S opsin-CNGA3 at 4 months (M) post injection (PI), (B) by AAV8-S opsin-CNGA3 at PI 1M and (C) by AAV8-S opsin-CNGA3 at PI 4M. UE, untreated eye. TE, treated eye. (D) Summary on the therapy onset data for AAV5 and AAV8 (Michalakis et al., 2011).

Based on these results the RD-Cure consortium decided for the AAV8 serotype for the CNGA3-linked achromatopsia intervention.

1.3. The rAAV.hCNGA3 Vector for the use in the human trial

The AAV gene therapy vector (AAV *cis* plasmid: pSub-hArr3-hCNGA3-WPREm-KanR) is based on the pSub201 *cis* plasmid backbone (Samulski et al., 1987) containing a kanamycin resistance gene. In this plasmid, the entire expression cassette is incorporated between two wild-type AAV2 inverted terminal repeats (ITRs). The expression cassette contains the 405 bp cone photoreceptor-specific human cone arrestin (hArr3) promoter (Li et al., 2002) and the full-length (2085 bp) human CNGA3 cDNA (Wissinger et al., 1997). The vector further contains a 543 bp woodchuck hepatitis virus post-transcriptional regulatory element (WPRE) with mutated WXF-open reading frame (Zanta-Boussif et al., 2009) and a 207 bp bovine growth hormone polyadenylation site (BGHpA). The AAV8 *trans* plasmid (pDP8-KanR) was obtained from the pDP8 plasmid described in Penaud-Budloo et al. (Penaud-Budloo et al., 2008) by exchange of the ampicillin by a kanamycin resistance gene.

1.3.1. Vector Production

The GMP grade AAV *cis* and *trans* plasmid DNA was manufactured by Aldevron (Fargo, ND, United States of America). The production was carried out using an *E.coli* master cell bank procedure according to USP and EMA guidelines. These plasmids were used for the GMP production of AAV8-pseudotyped hCNGA3 viral particles (AAV8-hCNGA3) at Atlantic BioGMP (Nantes, France). Atlantic BioGMP is a fully GMP compliant pharmaceutical site for the production of AAVs authorized by ANSM and operated by the French National Blood Institute (Etablissement Français du Sang – EFS).
The AAV8-hCNGA3 production process involves calcium phosphate transfection of cis (pSub-hArr3-hCNGA3-WPREm-KanR) and trans (pDP8-KanR) plasmids in HEK293 cells. Cells were cultured in Dulbecco's Modified Eagle's Medium (DMEM) with 10 % fetal bovine serum (FBS; conforming to the "TSE-Note for Guidance EMEA 410/01 rev 03" of the European Directorate for the Quality of Medicines and to the monograph „Bovine Serum, European Pharmacopoeia 01/2008:2262, 7th Ed. Ph. Eur.“). The medium did not contain any antibiotics or phenol red. AAV particles were harvested from cells and supernatant and purified by two consecutive CsCl-gradient centrifugation steps followed by tangential flow filtration (TFF) for buffer exchange and concentration, and finally sterile filtration (0.2µm) and filling. The final product is in Balanced Sterile Saline Solution for Intraocular Irrigation (SSSOI) (Industria Farceutica Galenica Senese, Ref: DD0412004, Supplier: Beaver-Visitec Intl Ref: 581732) with Kolliphor P188 Micro (= GMP grade PluronicF-68; BASF Ref: 50259816) (0.001% vol/vol). The product was filled into 1.2ml conical threaded polypropylene (PP) cryovials with threaded PP cap and a silicone washer (all USP Class VI, Corning Life Science Ref: 430658) at 0.13ml/vial and 5x10e12vg/ml (target genomic titer), finally stored at -70 °C ± 10°C. The production process and the final product conform to the "General chapter 5.14. of the 7th Ed. of the European Pharmacopoeia on gene transfer medicinal products for human use". Vector genome (vg) titer assayed by quantitative PCR (qPCR), infectious titer (infectious center assay, ICA) have been determined after each purification step. The harvest cells and supernatant were additionally tested for sterility (EP 2.6.1), mycoplasma (EP 2.6.7) and adventitious agents (EP 2.6.16). For the formulated final product, we will also determine the infectious titer using TCID50 (ip/ml) and vector particles using ELISA test (vp/ml). Finally, we will test for transgene expression (immunohistochemistry after treatment of CNGA3 KO mice), biological activity (electroretinography after treatment of CNGA3 KO mice), genomic identity (sequencing), protein purity (silver staining, Coomassie blue staining on SDS-PAGE), residual cell (qPCR for albumin and E1A) and plasmid (qPCR Kanamycin resistance gene) DNA, proteins (BCA, ELISAs), residual PEG, residual CsCl, residual benzonase (ELISA), endotoxins (EP2.6.14), pH, osmolarity (EP 2.2.35), aggregates and appearance. The final product will also be tested for replication competent AAVs.

1.3.2. Preclinical testing in rodents

ERG amplitude was used as a biomarker for cone function and measure for vector efficacy. The efficacy of the murine vector pAAV2.1-mBP-CNGA3 was proven in CNGA3-/- knock-out mouse model (Michalakis et al., 2012).
The GMP-produced human vector to be used in our planned human trial was applied in the same knock-out mouse model. The *in vivo* biological activity – although less pronounced in comparison to the murine vector – could be demonstrated 8 weeks after treatment (short-term effect) with the engineering lot used ACHM2-ENG05 which was also used for the toxicology and biodistribution in NHPs. Regarding long-term efficacy, there is clear evidence that efficacy of the human vector is stable until at least 12 months post treatment in the mouse model with the process developmental lot ACHM2-DEV01.

1.3.3. Preclinical testing in non-human primates

Short term (n=12, 28 day) toxicological and long term (n=22, 13 week) toxicology and biodistribution studies were performed in male and female cynomolgus monkeys. The surgical subretinal application procedure proved to be feasible and safe. Out of 34 animals, one showed a retinal detachment, otherwise no ophthalmological adverse events occurred. No serological or clinical adverse events were observed. Animal activity and behavior, ECG and weight were normal up to 13 weeks after subretinal vector administration.
Shedding from the NHP BD studies analyzed in lachrymal and nasal swaps and urine showed detectable DNA in single animals up today 5 (1/6 animals, lachrymal swap), day 7 (nasal swap, 1/6 animals) and day 2 (urine, 1/6 animals) in the low dose subretinal group. Vector DNA could also be identified in single animals up to day 7 (lachrymal swaps, 2/6 animals; nasal swap, 3/6 animals) and day 5 (urine, 2/6 animals) in the high dose subretinal cohort, up to day 7 (lachrymal swap, 1/4 animals; urine, 1/4 animals; nasal swap, 2/4 animals) in the via falsa (intravitreal) high dose cohort. For the majority of animals, at these time-points the number of copies per µg was below or close to the level of significance defined by the FDA (i.e. 100 copies/µg).
None of the animals (which received subretinal injections (either low n=6 or high dose n=6) showed detectable DNA levels in the blood 72h post-injection. In the high dose via falsa group, vector DNA could be identified in the blood of all 4 animals up to four weeks post-injection.
The surgery and the perioperative care in NHPs deviates considerably from the situation in the clinical trial. The instruments available for such a surgery are optimized for human use. Due to the anatomical differences of the eye (e.g. size, location in orbit), some of the safety measures routinely performed in humans were not applicable in the NHPs. For example, displacement of conjunctiva over sclerotomies and introduction of trocars at an oblique angle were not possible. Likewise, NHPs could not be instructed to avoid any manipulation of the eye after surgery to allow rapid healing of the self-sealing 23G sclerotomies. The investigators performing the NHP surgeries judged that stitching the sclerotomies would have

introduced more irritation and cause for manipulation, which would have additionally facilitated potential leakage of fluid from the vitreous cavity into the tear film, lachrymal and nasal system. This is in stark contrast to the planned surgery in human patients, which is performed to minimize leakage and patients are instructed not to manipulate the eye after surgery to facilitate rapid closure of the self-sealing sclerotomies.

At the same time, the low dose in the NHP sample corresponds to the maximum dose planned to be applied in the clinical trial. In particular, regarding the shedding the limited replication-competence needs to be considered.

rAAV.hCNGA3 is unable to replicate independently, even in the presence of a helper virus, since it lacks the rep and cap genes required for rescue/packaging. Homologous recombination between rAAV.hCNGA3 and a wild type AAV could occur if both were present in the same cell. However, such recombination could only result in the exchange of the hCNGA3 expression cassette with the rep and cap genes of the wild type virus. It is not possible for the AAV genome to contain both rep/cap genes and the transgene, as this is beyond the packaging limit of the virion. Therefore the only mechanism by which the transgene could be mobilised is through a triple infection of the same cell by rAAV.hCNGA3 (containing the transgene), wild type AAV (providing the rep and cap functions) and a helper virus. This scenario is expected to be an extremely unlikely event, especially since the vector target cells (cone photoreceptors) are not the natural target cells of helper viruses and are shielded from viral infection by the blood-retina barrier. If it did occur, it would only result in the production of more wild type AAV and more rAAV.hCNGA3 vector particles (which would still lack rep and cap genes and consequently could not be self-sustaining).

Nevertheless, safety countermeasures will be taken for the clinical phase I trial with regard to the results mentioned above. Until day 7 post-injection, any materials and tissues coming into contact with the patient’s eyes, lachrymal fluid and nasal secretions will be subject to disinfection or autoclaving respectively. Study patients will be instructed to avoid intimate physical contact to other subjects until day 7 post-injection.

2. Aim of the Trial

The aim of this phase I trial is to proof the safety and efficacy of rAAV.hCNGA3 at three dosages in patients with achromatopsia.

2.1. Primary Aim

The primary aim of the trial is the investigation of the safety of rAAV.hCNGA3 after subretinal injection in patients with CNGA3-linked achromatopsia. Both the ocular and the systemic safety of the intervention will be investigated. Details regarding methods and parameters are described in chapters 7 and 8 of this trial protocol.

2.2. Secondary Aims

The investigation of treatment effects as reflected by patient reported outcomes and the efficacy of the intervention on visual function, as well as the evaluation of retinal imaging (safety) are secondary aims of the trial.

3. Study Plan

3.1. Study Design

This is an open mono-center trial with fellow-eye comparison.
Three patients will be assigned to three consecutive, increasing dosage groups (see figure 3):

1) low dose: ≤ 1x10e10 vgp (n=3)

After collection of data on study visit Day 30 of all three patients of the dose cohort and before the scheduled start of the next injections of the consecutive dosage group the DMC will meet and evaluate safety data. If no safety concerns are reported the next dosage group of patients will be treated as scheduled. DMC advice may also result in a dose reduction for the next dosage cohort.

2) intermediate dose: ≤ 5x10e10 vgp (n=3)

After collection of data on study visit Day 30 and before the scheduled start of the next injections of the consecutive dosage group the DMC will meet and evaluate safety data of groups 1 and 2. If no safety concerns are reported the next dosage group of patients will be treated as scheduled. DMC advice may also result in a dose reduction for the next dosage cohort. Within the high dose cohort, the interval between injections / individual patients will be four weeks.

3) high dose: ≤ 1x10e11 vgp (n=3)

The following figure (Fig. 3) explains the schedule and timelines of dose groups and the points of decisions of the data monitoring committee.

Dosage/Dose Regimen for rAAV.hCNGA3 Phase I Trial

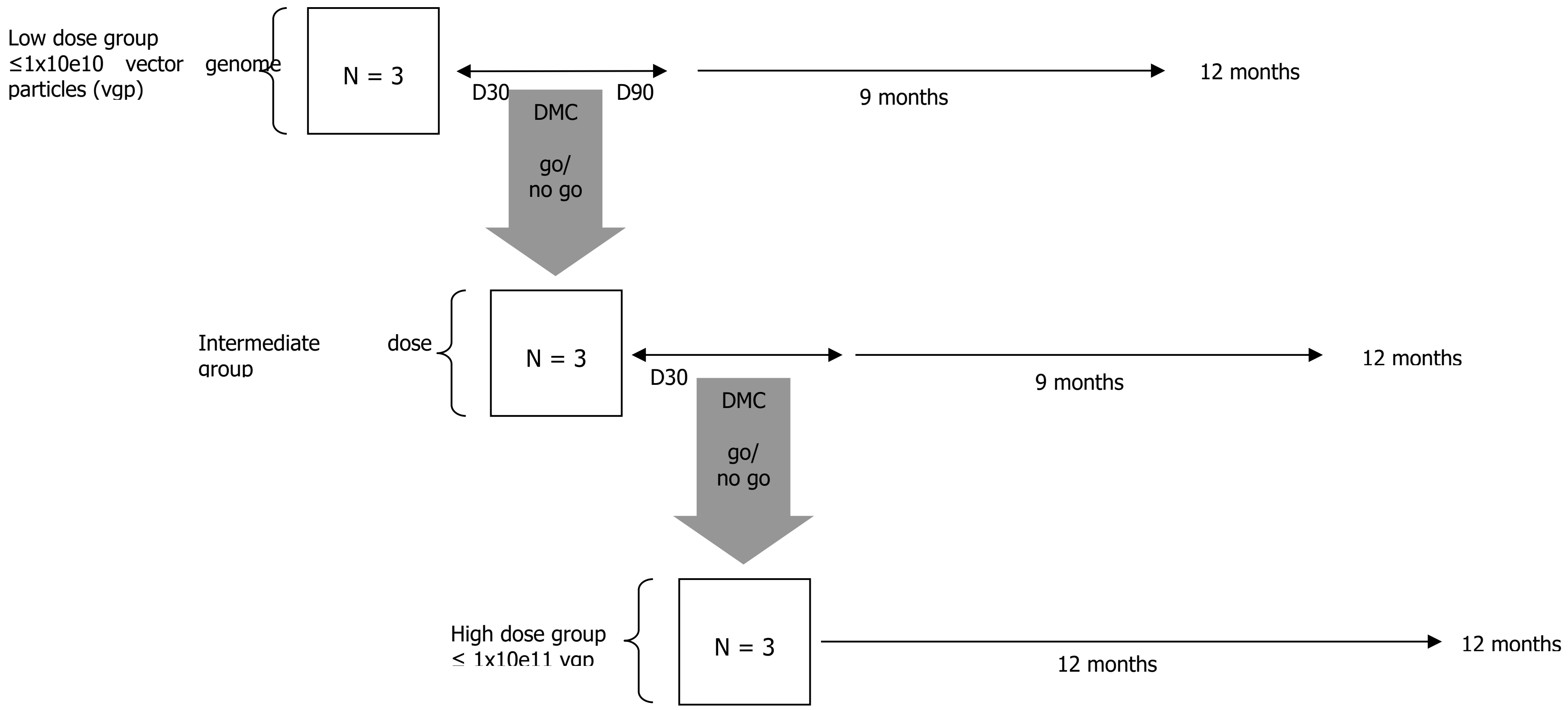

Figure 3: Schedule of dosing groups and intervals

3.2. Study Duration

- First patient first visit: June 2015
- Last patient first visit: December 2015
- Last patient last visit: December 2016
- Duration of trial participation per patient: 1 year plus 4-year follow-up period

3.3. Participating Sites

The trial will be performed at the Centre for Ophthalmology, University of Tübingen. The site has a database of clinically and genetically characterized patients. In addition specialized retinal surgeons of the institution are familiar with subretinal interventions and have been trained in gene-therapeutic injections in an ongoing collaboration of the Centre for Ophthalmology with Oxford Eye Hospital, UK.

3.4. Number of enrolled patients

Nine patients will be enrolled in the trial. Drop-outs will only be replaced in case of non-ocular, non-treatment related reasons for the drop-out up to a number of two patients.

4. Study Population

4.1. Characterization of Patient Population

For this trial, adult patients of both genders with CNGA3-linked achromatopsia will be eligible (natural course and clinical symptoms see section 1.1.1.) with a BCVA ≥ 20/400 and discernable residual outer nuclear layer thickness in OCT scans of posterior pole. The precondition is the confirmed presence of mutation in the CNGA3 gene. A) Patients will be pre-identified via the patient database of the Centre for Ophthalmology, University of Tübingen, they will be informed about the trial and invited for trial screening. B) In addition, new patients to the clinic will be identified, informed about the trial and invited for trial screening. C) Patients from other hospitals, asking for trial participation will also be invited for screening.
Principally an intervention as early as possible in the course of the disease would be preferable, especially as the disease was initially thought to be non-progressive because cone function was thought to be absent from birth. Yet, recent morphological in vivo studies established ACHM to be a slowly progressing degenerative retinal disease, and foveolar cone photoreceptor loss can be observed

in most adult ACHM patients (Thiadens et al., 2010; Aboshiha et al., 2014; Greenberg et al., 2014; Sundaram et al., 2014; Yang et al., 2014). Therefore, it is deemed ethical to treat adult patients in this first interventional trial with the vector.

4.2. Inclusion Criteria (Study Eye)

1. clinical diagnosis of achromatopsia
2. ≥ 18 years of age
3. confirmed mutation in CNGA3
4. BCVA ≥ 20/400; first cohort: BCVA ≤ 20/160
5. area of residual outer nuclear layer thickness in OCT scans of posterior pole
6. ability to understand and consent to study protocol

4.3. Exclusion Criteria

- additional interfering eye conditions (e.g. uveitis, advanced cataract) in the study eye
- systemic conditions (e.g. coronary heart disease, autoimmune disorders) which may affect study participation or outcome measures

1. current or recent (30 days) participation in other study and/or receipt of biologic agent
2. gross asymmetry of ocular condition/lack of control eye
3. recent (≤ 6 months) ocular surgery, intravitreal or subretinal implantation of a medical device
4. known sensitivity to any compound used in the study
5. contraindications to systemic immunosuppression
6. subject/partner with childbearing potential unwilling to use adequate contraception for four months
7. nursing and pregnant women
8. any other cause that, in the investigator‘s opinion, renders potential subjects not suitable for the study

Previous studies have shown no change in humoral immunity against AAV capsids after subretinal application of AAV8 vectors. Therefore, the serostatus of potential study patients will not be relevant for trial inclusion.

5. Patient Inclusion

5.1. Time plan for inclusion

Patients will be screened for their eligibility after informed consent. Screening examinations may be distributed over three working days and should be performed in a time window of 30 to 2 days before treatment. There are no restrictions or recommendations with regard to the time after first diagnosis or the stage of the disease beyond the inclusion and exclusion criteria.

5.2. Mode of assignment of patients to treatment

Suitable patients in the patient data base of the Centre for Ophthalmology have already been identified and invited to an exploratory non-invasive trial, which has been running since December 2012 ("Detaillierte Genotyp-Phänotyp Analyse bei Patienten mit erblichen, genetisch gesicherten Netzhauterkrankungen" "Detailed genotype-phenotype analysis of patients with inherited genetically ensured retinal-diseases"). The patients out of this sample who qualify and are interested in participation in the rAAV.hCNGA3 trial will be invited for informed consent and screening visit.
For the first treatment cohort the inclusion criterion for visual acuity is BCVA ≤20/160 to further limit the risks of the first-dose-in-man cohort.

6. Intervention

6.1. Treatment

The principle of treatment is shown in figure 3. There are three incremental steps of dosages of the vector.

- Low dose: ≤ 1x10e10 vector genome particles (vgp)
- Intermediate dose: ≤ 5x10e10vgp
- High dose: ≤ 1x10e11vgp

See Appendix 1 Rationale for vector and dosing for more details of the procedure.

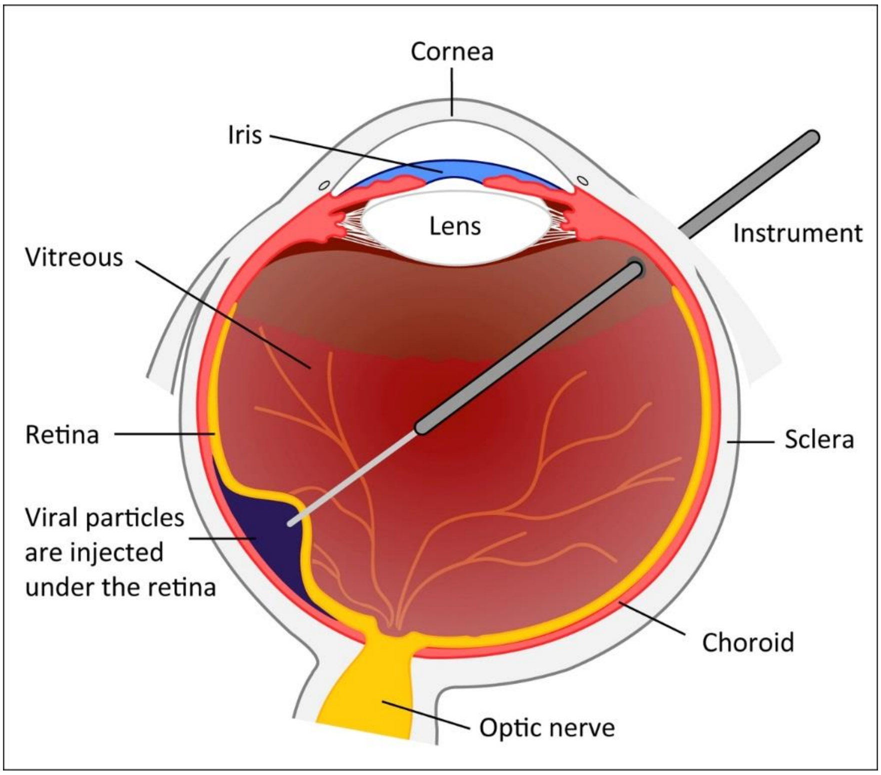

Figure 4: Principle of subretinal vector injection.

All patients will be treated with a single intravitreal injection of one of three dosages of rAAV8 vector. Starting 1 day before treatment, immunosuppression by oral corticosteroids and topical steroids will be performed.

6.2. Concomitant medication

There are no forbidden medications during trial participation beyond the avoidance of anticoagulants around the surgical intervention with time lags depending on the half-life of the relevant drugs. Any concomitant medication will be documented during the trial.

7. Endpoints for Safety and Efficacy of a single subretinal injection of rAAV.hCNGA3 genome

7.1. Efficacy

Efficacy data (improvement in visual function) and patient reported outcomes will be investigated exploratively as well as retinal imaging. They are all secondary endpoints in this trial.

1. BCVA assessed using the ETDRS visual acuity protocol
2. Contrast sensitivity
3. Anomaloscopy
4. Cambridge Colour test
5. Flicker Fusion Test
6. Colour Constancy
7. MP-1 Microperimetry (20°)
8. Dark adaptation test (FST)
9. IR-Video-Pupillography
10. OCT, AO-SLO, IR, FAF and angiography recordings
11. FST (red/blue) and Ganzfeld-ERG
12. VFQ25
13. Brief Symptom Inventory (BSI)
14. A3-PRO (intervention-specific scale assessing patient reported outcome)

7.2. Safety

7.2.1. Ocular and systemic safety

Safety as primary endpoint will be assessed by clinical examination of

Ocular safety

1. loss of ≥ 15 letters visual acuity at 1m
2. severe, vector-induced, intraocular inflammation unresponsive to treatment

Systemic safety

1. vital signs
2. routine clinical chemistry testing (including CRP, IgG, IgM and full/differential blood counts
3. Immunopathology essays will include specific enzyme-linked immunosorbent assays for humoral antibodies against rAAV8 capsid protein and/or CNGA3 gene product and specific enzyme-linked immunosorbent spot assays to monitor cellular immune reactivity against rAAV8 capsid protein and/or CNGA3 gene product.

4. Biodistribution will be monitored by polymerase chain reaction studies on rAAV2/8 genome in blood and urine.

7.2.2. Data Monitoring Committee

A Data Monitoring Committee (DMC) will monitor patients' safety at regular meetings. At crucial steps of the trial (see figure 3) such as after day 30, when safety data of previous patient cohort is available, the committee will provide a go/no go decision for the treatment of the next dose cohort. The DMC consists of Prof. Eberhart Zrenner, Tübingen, Prof. Robert MacLaren, Oxford, and Prof. Christian Hamel, Montpellier. The members of the DMC are independent of the clinical trial.

7.2.3. Definitions

Adverse Event (AE)

An AE or adverse experience is any untoward medical occurrence in a patient or clinical investigation participant administered a medicinal product, which does not necessarily have to have a causal relationship with the study treatment.
An AE can therefore be any unfavourable and unintended sign (including an abnormal laboratory finding), symptom or disease temporarily associated with the use of the study medication, whether or not considered related to the study medication.

Adverse Reaction (AR)

An AR or adverse reaction is an untoward and unintended reaction to a medicinal product related to any dose. The phrase "reaction to a medicinal product" means that a causal relationship between a study medication and an AE is at least a reasonable possibility, i.e., the relationship cannot be ruled out. All cases judged by either the reporting medically qualified professional or the sponsor as having a reasonable suspected causal relationship to the study medication qualify as adverse reactions.

Serious Adverse Event (SAE)

A serious adverse event is any untoward medical occurrence that at any dose:

- Results in death,
- Is life-threatening,
- Requires inpatient hospitalization or prolongation of existing hospitalization,
- Results in persistent or significant disability/incapacity, or
- Is a congenital anomaly/birth defect.

It is important to consider that the term "life-threatening" in the definition of "serious" refers to an event in which the participant was at risk of death at the time of the event; it does not refer to an event, which hypothetically might have caused death if it were more severe.

Serious Adverse Reaction (SAR)
A SAR, a serious adverse reaction, is an adverse event (expected or unexpected) that is both serious and, in the opinion of the reporting investigator, believed with reasonable probability to be due to one of the study treatments, based on the information provided.

Expected Serious Adverse Events/Reactions
Expected serious adverse events/reactions (ESAE/ESAR) are most likely to be related to (1) complications of retinal surgery, (2) immune reactions to the vector capsid or (3) immune reactions and/or toxic effects on retinal function caused by expression of human CNGA3 protein.
(1) Complications of retinal surgery
There are known complications of three port pars plana vitrectomy and subretinal surgery. These include retinal detachment, traumatic cataract, suprachoroidal and/or subretinal hemorrhage and endophthalmitis.
(2) Immune reactions to vector capsid
These are not expected, given the safety history with much larger doses of this vector in non-ophthalmological clinical trials (Nathwani et al., 2011), but cannot be ruled out completely. Therefore, presence of immune reactions will be investigated during the follow-up clinical examinations. Severe immune reactions triggered by the vector are likely to be evident within the first day of administration and might include non-infectious severe vitritis, chorioiditis or endophthalmitis. There may be additional systemic reactions such as headache and fever. Hence the patient will be monitored overnight and undergo a check up on the day after surgery.
(3) Immune reactions and/or toxic effects on retinal function caused by AAV8-CNGA3
Due to the time required for transgene expression following subretinal delivery of AAV8, it is unlikely that any toxic effects of the transgene product would be evident prior to at least two weeks after surgery at the earliest. These might include similar immune reactions as described in (2) above. Also any toxic effects of AAV8-CNGA3 on visual function might be manifest by a worsening of visual acuity after the initial period of post-operative recovery. Potential toxic effects on retinal function are likely to be manifest as a significant drop in visual acuity (defined by 15 letters or more).

Suspected Unexpected Serious Adverse Reaction (SUSAR)
A suspected unexpected serious adverse reaction is a reaction, the nature or severity of which is not consistent with the applicable product information as documented in the Investigator's Brochure and which at the same time is regarded as potentially related to the administration of the investigational product.

7.3. Reporting Procedures for all Adverse Events

All AEs occurring during the study observed by the investigator or reported by the participant, whether or not attributed to study medication, will be recorded on the CRF. The following information will be recorded: description, date of onset and end date, severity, assessment of relatedness to study medication, and action taken.

AEs considered related to the study medication as judged by a medically qualified investigator or the sponsor will be followed until resolution or the event is considered stable and/or until the end of the follow-up period (4 years).
The severity of events will be assessed on the following scale: 1 = mild, 2 = moderate, 3 = severe. The relationship of AEs to the study medication will be assessed by a medically qualified investigator and discussed with the PI.
Any *pregnancy* occurring during the clinical study and the outcome of the pregnancy of female patients or fathered by trial participants, should be recorded and followed up for congenital abnormality or birth defect until delivery. Pregnancy itself is documented as AE, in case of hospitalization as SAE.

7.3.1. Reporting Procedures for Serious Adverse Events

The Data Monitoring Committee (DMC) will review all SAEs for the study at certain time-points of the trial. The DMC is detailed in Appendix B and may hold personal /TC/electronic meetings. The DMC will meet at regular intervals and consider:

- Occurrence and nature of adverse events
- Whether additional information on adverse events is required
- Consider taking appropriate action where necessary to halt the trial (see below)
- Act / advise on incidents occurring between meetings that require rapid assessment (e.g. SUSARs)

Especially before the second and third trial cohort, when the Day 30 results are available, the DMC must meet and make a go/no-go decision for the treatment of the next dose cohort, which needs to be reported to the PI as soon as possible.
All SAEs will be reported to the DMC within one day of discovery or notification of the event. All SAE information will be recorded on an SAE form, which will be sent electronically to members of the DMC. Additional information received for a case (follow-up or corrections to the original case) will be detailed on a new SAE form.
The CRO will – on behalf of the Sponsor - report all AEs, SAEs and SUSARs to the Ethics Committee and the Regulatory Authority (PEI) in an annual safety report. Fatal or life-threatening SUSARs will be reported within 7 days and all other SUSARs within 15 days. The PI will also inform all members of the study group on all adverse events that might affect the safety of participants.

7.4. Rules for discontinuation of the trial

Any of the following events will result in discontinuation of the trial:

1. Severe loss of vision (more than 15 letters ETDRS acuity) occurring deemed to be a result of vector administration.
2. Severe ocular, vector-induced inflammation, unresponsive to treatment (endophthalmitis).
3. Any suspected unexpected serious adverse reaction (SUSAR).

4. Safety concerns other than those mentioned under 1-3 and consecutive request for discontinuation by the Data Monitoring Committee.

If an Adverse Event was the reason for discontinuation of the trial either by the PI or the participant, the participant must undergo an early termination visit (see flowchart). The patient must be given appropriate care under medical supervision until symptoms cease or the condition becomes stable. Maximal follow-up duration will be 1 year after trial termination

7.5. Risk-Benefit Considerations

The **risks** of the subretinal vector injection are related to 1) the surgical procedure three port pars plana vitrectomy and subretinal surgery as well as 2) the ophthalmic or systemic effects of the rAAV.hCNGA3 vector.
Regarding 1) there may be a potential loss of visual function due to complications of the surgical procedure such as bacterial infection, retinal detachment, suprachoroidal and/or subretinal hemorrhage and cataract as a consequence of the surgical trauma. These risks are treatable by standard ophthalmological care but may result in lack of partial or complete restoration of the visual function loss.
Table 2 gives an overview of known risks by pars plana vitrectomy based on the literature and experiences from NHP trials with the vector of this study and estimates the rates to be expected in the current trial.

**Surgical complications during 23g PPV**	Overall rate with mixed indications [1-3]	Incidence in NHP study	Predicted risk in gene therapy trial
retinal detachment / breaks	1-10 %	0%	1%
endophthalmitis	0.01-1 %	0%	0.01-1 %
wound leakage / transient hypotony	5-20 %	100%	5-20 %
choroidal hemorrhage / detachment	< 1 %	0%	< 1 %
suprachoroidal perfusion	< 1 %	0%	< 1 %
vitreal hemorrhage	1-5%	0%	< 1 %
cataract formation	1-5%	9%	10-20%
transient intraocular hypertension	5%	0%	5%
corneal erosion	5-20 %	5-20 %	5-20 %

Table 2: *Risks of complications during 23g PPV referring to literature, previous NHP studies and expected rates for the planned trial. [1] Wilkinson et al., 2013 [2]* Lee et al., 2012 *[3] Wykoff et al., 2010.*

Regarding 2) there is a risk for a loss of vision due to ophthalmic immune reactions to the vector (indirect) or due to direct effects of the vector (however, pre-clinical studies showed no toxic effects with up to 10 fold higher concentrations). If any immune reaction should be triggered by the vector, this might include non-infectious inflammation of the pigment epithelium (epitheliitis), retina (retinitis), vitreous cavity (vitritis) or uveal tissue (uveitis). None of these have been reported in any previous clinical trial involving AAV in ocular gene transfer.

Such effects may result in a significant deterioration of visual function (decrease in VA of ≥ 15 letters) and might also occur after initial improvement. A vulnerable period for direct and/or indirect (immunogenic) effects due to the viral packaging protein of the vector would be the first two weeks (time of concomitant steroid treatment). No clinical trial has as of yet reported immune-reaction to the transgene (even in systemic gene transfer). It therefore seems unlikely that a direct or indirect effect due to transgene expression would occur after ocular gene transfer in the immune-privileged subretinal space.

Previous clinical trials with the same viral vector build have demonstrated good safety profiles (Nathwani et al., 2011). Other trials have shown good safety profiles after subretinal application of similar viral vector constructs (rAAV2) (Bainbridge et al., 2008; Hauswirth et al., 2008; Maguire et al., 2008; Maguire et al., 2009; Bennett et al., 2012; Jacobson et al., 2012). However, systemic risks cannot be completely ruled out and could stem from - previously undetected - **immune reactions**. Both the virus capsid and transgenic protein could potentially activate the immune system. The immune-privilege of the eye severely limits the likelihood of antigen presentation to leukocytes. The compartmentalization of the eye limits the biodistribution of viral vector. Much lower doses are required in the eye compared to previous studies targeting the liver (Nathwani et al., 2011). The transgenic protein is not secreted but expressed intracellularly and only in target cells (cone photoreceptors) due to a cone photoreceptor specific promoter. In light of these arguments and supported by results from previous clinical trials, it seems unlikely that a significant immune reaction is staged after intervention. Nevertheless, as immune reactions cannot be ruled out completely, patients will be screened accordingly during the follow-up clinical examinations. Any systemic reactions may appear during the first days as headache and fever. For safety reasons, patients will be hospitalized for three days including monitoring over the first night.

The potential of **malignancy** due to the AAV vector is also very unlikely. In contrast to e.g. lentiviral vectors, rAAV are considered non-integrating and result in episome

formation in the transduced cells rather than integrating the transgene cassette in the host genome. Wild-type AAVs carry the complete virus genome including re *Rep* genes, which orchestrate the integration of vector DNA (preferentially at the AAVS1 locus (c19q13.3)). Deletion of AAV *Rep* in the recombinant vector reduces these integration events by more than 99.5% (Schnepp et al., 2003). So far, there has been no report on insertional mutagenesis of recombinant AAV (Lipinski et al., 2013). Nevertheless, all patients in this trial will be screened regularly even after study close out.
Dissemination of rAAV.hCNGA3 would most likely only occur between human beings, since it is derived from AAV2/8. However no replication is expected in normal cells of treated individuals exposed to the replication-deficient virus, or from exposure of uninfected people to treated individuals.

Germline transmission is theoretically possible but requires biodistribution far beyond what could be detected today combined with the extremely unlikely event of genomic integration.

Currently, there is no experience with the specific **teratogenity** of the rAAV.hCNGA3 vector, therefore the risk for an unborn child in case of an unforeseen conception, especially during the first half of the study participation, cannot be judged.

Generally, there is the potential of no subjective or objective benefit despite the risks of the procedure.

Patient **benefits** may consist in a deceleration or even halt of cone photoreceptor degeneration and in the initiation of cone photoreceptor function. This might include improvement of visual acuity, decreased glare, increased ability of color discrimination and decreased nystagmus. Especially the debilitating glare is a major impairment in achromatopsia patients' daily life. In addition, many patients are hampered by their very low vision. Beyond glare and visual acuity, novel colour perceptions may be an individual gain for the patients and their performance in daily life. Improvements in visual functions are known to increase the vision-related quality of life and general well-being.

7.6. Risks for the Environment

These are dealt with in detail in the separate document *Information required concerning releases of genetically modified organisms* according to Annex IIIa of the DIRECTIVE 2001/18/EC OF THE EUROPEAN PARLIAMENT AND OF THE COUNCIL of 12 March 2001 on the deliberate release into the environment of genetically modified organisms and repealing Council Directive 90/220/EEC.

Dissemination of rAAV.hCNGA3 would most likely only occur between human beings, since it is derived from AAV2/8. However no replication is expected in normal cells of treated individuals exposed to the replication-deficient virus, or from exposure of

uninfected people to treated individuals.

Safety countermeasures will be taken for the clinical phase I trial with regard to the results mentioned above. Until day 7 post-injection, any materials and tissues coming into contact with the patient's eyes, lachrymal fluid and nasal secretions will be subject to disinfection or autoclaving respectively. Study patients will be instructed to not manipulate the eye after surgery to facilitate rapid closure of the self-sealing sclerotomies. Study patients will also be instructed to avoid intimate physical contact to other subjects until day 7 post-injection (See also section 1.3.3.).

8. Examinations

All study visits described in the flowchart, page 14, and in section 8.1. may be performed on two consecutive days; screening and close-out visit on three days if deemed necessary. The reason behind this is to reduce the workload due to concentration during test procedures and to improve the reliability of the test results.

8.1. Screening Examinations

(O) = optional, if control deemed necessary by investigator

Visit 1
Screening (Day -30 to -2)

- Medical/surgical history
- Ocular/surgical history
- BMI
- Vital Signs
- Urine pregnancy test
- Hematology/basic chemistry/urine analysis
- CRP, IgG, IgM
- Immunopathology
- PCR of rAAV8 genome
- Best corrected visual acuity
- Basic ophthalmological exam (miosis)
- Contrast sensitivity (PR Charts)
- Flicker Fusion Frequency
- Colour constancy (chromatic adaption)
- Anomaloscopy
- Cambridge Colour Test
- IR Video Pupillography
- MP-1 Microperimetry (20°)
- Funduscopy (mydriasis)
- Dark adaption test
- ffERG (scotopic and photopic)
- sd-OCT

- Fundus autofluorescence
- Fundus photography
- Angiography
- Systemic steroids
- Topical steroids and antibiotics
- VFQ25
- Psychiatric examination
- Brief Symptom Inventory (BSI)
- Concomitant medication

8.2. Trial Examinations

Visit 2

D0

- Vital signs
- Urine pregnancy test
- Hematology/ basic chemistry (O)
- Basic ophthalmological exam (miosis)
- Funduscopy
- sdOCT (O)
- Systemic steroids
- Topical steroids and antibiotics
- Subretinal injection of rAAV8 vector
- Psychological counselling
- Adverse event recording
- Concomitant medication

Visit 3

D1

- Vital signs (O)
- Hematology/ basic chemistry (O)
- Basic ophthalmological exam (miosis)
- Funduscopy (mydriasis)
- sdOCT (O)
- Systemic steroids
- Topical steroids and antibiotics
- Psychological counselling
- Adverse event recording
- Concomitant medication

Visit 4

D2

- Vital signs (O)
- Hematology/ basic chemistry (O)

- CRP, IgG, IgM
- Basic ophthalmological exam
- Funduscopy (mydriasis)
- sdOCT (O)
- Systemic steroids
- Topical steroids and antibiotics
- Psychological counselling
- Adverse event recording
- Concomitant medication

Visit 5
D3 (± 1 Day)

- Vital signs
- Hematology/ basic chemistry
- CRP, IgG, IgM
- PCR of rAAV8 genome
- Basic ophthalmological exam (miosis)
- Funduscopy (mydriasis)
- Contrast sensitivity (PR charts)
- sdOCT (O)
- Systemic steroids
- Topical steroids and antibiotics
- Psychological counselling
- Adverse event recording
- Concomitant medication

Visit 6
D14 (± 3 days)

- Vital signs (O)
- Urine pregnancy test
- Hematology/ basic chemistry
- CRP, IgG, IgM
- PCR of rAAV8 genome
- Basic ophthalmological exam (miosis)
- Visual Acuity, BCVA (ETDRS)
- Contrast sensitivity (PR charts)
- Anomaloscopy
- Cambridge Colour Test
- Funduscopy
- sdOCT
- FAF
- FF

- Angiography (O)
- Anomaloscopy
- Roth 28-hue test (sat. and de-sat.)
- IR-Video-Pupillography
- MP-1 Microperimetry (20°)
- Dark adaptation test (FST)
- Topical steroids and antibiotics
- Brief Symptom Inventory (BSI)
- Psychological counselling
- Study specific scale, A3-PRO
- Adverse event recording
- Concomitant medication

Visit 7
D30 (± 5 days)

- Vital signs (O)
- Urine pregnancy test
- Hematology/ basic chemistry (O)
- CRP, IgG, IgM
- Immunopathology
- PCR of rAAV8 genome (O)
- Visual Acuity, BCVA (ETDRS)
- Basic ophthalmological exam (miosis)
- Contrast sensitivity (PR charts)
- Flicker Fusion Frequency
- Colour Constancy (chromatic adaption) (O)
- Anomaloscopy
- Cambridge Colour Test
- IR Video Pupillography
- MP-1 Microperimetry (20°)
- Funduscopy (mydriasis)
- Dark adaptation test
- ff-ERG (scotopic and photopic)
- sdOCT
- FAF
- FF
- Angiography (O)
- Topical steroids and antibiotics
- VFQ25
- Brief Symptom Inventory (BSI)
- Psychological counselling
- Study specific scale, A3-PRO
- Adverse event recording
- Concomitant Medication

Visit 8
D90 (± 7 days)

- Vital signs (O)
- Urine pregnancy test
- Hematology/ basic chemistry (O)
- CRP, IgG, IgM (O)
- Immunopathology
- PCR of rAAV8 genome (O)
- Visual Acuity, BCVA (ETDRS)
- Basic ophthalmological exam
- Contrast sensitivity (PR charts)
- Flicker Fusion Frequency
- Colour Constancy (chromatic adaption) (O)
- Anomaloscopy
- Cambridge Colour Test
- IR Video Pupillography
- MP-1 Microperimetry (20°)
- Funduscopy (mydriasis)
- Dark adaptation test
- ff-ERG (scotopic and photopic) (O)
- sdOCT (O)
- FAF (O)
- FF
- Angiography
- Brief Symptom Inventory (BSI)
- Psychological counselling
- Study specific scale, A3-PRO
- Adverse event recording
- Concomitant medication

Visit 9
D180 (± 7 days)

- Vital signs (O)
- Urine pregnancy test
- Hematology/ basic chemistry
- CRP, IgG, IgM (O)
- Immunopathology (O)
- PCR of rAAV8 genome (O)
- Visual Acuity, BCVA (ETDRS)
- Basic ophthalmological exam
- Contrast sensitivity (PR charts)
- Flicker Fusion Frequency
- Colour Constancy (chromatic adaption)

- Anomaloscopy
- Cambridge Colour Test
- IR Video Pupillography
- MP-1 Microperimetry (20°)
- Funduscopy (mydriasis)
- Dark adaptation test
- ff-ERG (scotopic and photopic) (O)
- sdOCT (O)
- FAF (O)
- FF (O)
- Angiography (O)
- VFQ25
- Brief Symptom Inventory (BSI)
- Psychological counselling
- Study specific scale, A3-PRO
- Adverse event recording
- Concomitant medication

Visit 10
D365, Close out (± 14 days)

- BMI
- Vital signs
- Urine pregnancy test
- Hematology/ basic chemistry
- CRP, IgG, IgM (O)
- Immunopathology (O)
- PCR of rAAV8 genome (O)
- Visual Acuity, BCVA (ETDRS)
- Basic ophthalmological exam
- Contrast sensitivity (PR charts)
- Flicker Fusion Frequency
- Colour Constancy (chromatic adaption)
- Anomaloscopy
- Cambridge Colour Test
- IR Video Pupillography
- MP-1 Microperimetry (20°)
- Funduscopy (mydriasis)
- Dark adaptation test
- ff-ERG (scotopic and photopic)
- sdOCT
- FAF
- FF
- Angiography
- VFQ25

- Brief Symptom Inventory (BSI)
- Psychological counselling
- Study specific scale, A3-PRO
- Adverse event recording
- Concomitant medication

8.3. Follow-up Examinations

Visit 11

Fup 1 M24 (± 1 month)

- Vital signs (O)
- Hematology/ basic chemistry
- CRP, IgG, IgM (O)
- Immunopathology (O)
- PCR of rAAV8 genome (O)
- Visual Acuity, BCVA (ETDRS)
- Basic ophthalmological exam
- Contrast sensitivity (PR charts)
- Anomaloscopy (O)
- Cambridge Colour Test (O)
- IR-Video-Pupillography (O)
- MP-1 Microperimetry (O)
- Funduscopy (mydriasis)
- Dark adaption test (O)
- ff-ERG (scotopic and photopic) (O)
- sdOCT
- FAF
- FF
- Angiography (O)
- VFQ25 (O)
- Study specific scale, A3-PRO
- Adverse event recording
- Concomitant medication

Visit 12

Fup 2 M36 (± 1 month)

- Vital signs (O)
- Hematology/ basic chemistry
- CRP, IgG, IgM (O)
- Immunopathology (O)
- PCR of rAAV8 genome (O)
- Visual Acuity, BCVA (ETDRS)
- Basic ophthalmological exam
- Contrast sensitivity (PR charts)

- Anomaloscopy (O)
- Cambridge Colour Test (O)
- IR-Video-Pupillography (O)
- MP-1 Microperimetry (O)
- Funduscopy (mydriasis)
- Dark adaption test (O)
- ff-ERG (scotopic and photopic) (O)
- sdOCT
- FAF
- FF
- Angiography (O)
- VFQ25 (O)
- Study specific scale, A3-PRO
- Adverse event recording
- Concomitant medication

Visit 13
Fup 3 M48 (± 1 month)

- Vital signs (O)
- Hematology/ basic chemistry
- CRP, IgG, IgM (O)
- Immunopathology (O)
- PCR of rAAV8 genome (O)
- Visual Acuity, BCVA (ETDRS)
- Basic ophthalmological exam
- Contrast sensitivity (PR charts)
- Anomaloscopy (O)
- Cambridge Colour Test (O)
- IR-Video-Pupillography (O)
- MP-1 Microperimetry (O)
- Funduscopy (mydriasis)
- Dark adaption test (O)
- ff-ERG (scotopic and photopic) (O)
- sdOCT
- FAF
- FF
- Angiography (O)
- VFQ25 (O)
- Study specific scale, A3-PRO
- Adverse event recording
- Concomitant medication

Visit 14
Fup 4 M60 (± 1 month)

- Vital signs (O)

- Hematology/ basic chemistry
- CRP, IgG, IgM (O)
- Immunopathology (O)
- PCR of rAAV8 genome (O)
- Visual Acuity, BCVA (ETDRS)
- Basic ophthalmological exam
- Contrast sensitivity (PR charts)
- Anomaloscopy (O)
- Cambridge Colour Test (O)
- IR-Video-Pupillography (O)
- MP-1 Microperimetry (O)
- Funduscopy (mydriasis)
- Dark adaption test (O)
- ff-ERG (scotopic and photopic) (O)
- sdOCT
- FAF
- FF
- Angiography (O)
- VFQ25 (O)
- Study specific scale, A3-PRO
- Adverse event recording
- Concomitant medication

8.4. Non-ophthalmological and ophthalmological Procedures

All procedures should be performed in the sequence given by the flowchart, whenever possible. If feasible, the same investigators should perform the examinations for the study duration. Advanced test procedures, such as, but not limited to BCVA, microperimetry and pupillography, will be performed by study personnel after method-specific training.
If not specified differently, all tests described here will be performed according to the Flowchart of Visits and Procedures in *both* eyes.
All ophthalmological procedures will be performed in a fixed sequence, testing the study eye first and the fellow eye afterwards.

More details of all examination procedures can be found in the Procedure Manual in its current version as approved by the Principal Investigator.

8.4.1. Medical / surgical history

Essential events have to be documented in the patient file / worksheet with dates of onset and underlying pathology.

8.4.2. Ocular / surgical history

Any ophthalmological disease or surgery in either eye has to be documented with date of onset.

8.4.3. Body Mass Index

Deriving from height and weight of the patient, the BMI (kg/m^2) will be calculated.

8.4.4. Vital signs

After 10 minutes resting in a sitting position blood pressure, heart rate and body temperature will be measured.

8.4.5. Urine pregnancy test

In female subjects with childbearing potential a commercial urine pregnancy test will be performed by the study nurse / study coordinator at all marked visits.

8.4.6. Hematology, basic chemistry and urine analysis

The following parameters will be tested at those visits where blood and urine tests are foreseen:

- Sedimentation rate
- Differential Blood count
- Hemoglobin
- Hematocrit
- Platelet count
- Glucose
- Sodium
- Potassium
- Uric acid
- Creatinine
- Creatinine kinase
- LDH
- Total bilirubin
- Direct bilirubin
- AST (SGOT)
- ALT (SGPT)
- γGT
- Alkaline phosphatase
- Proteine electrophoresis
- Coagulation (INR, PTT, aPTT)
- Urine analysis

8.4.7. CRP, Immunoglobulines

CRP, IgG and IgM will be determined at all marked study visits.

8.4.8. Immunopathology

In a specialized local lab (Prof. Specht) anti-AAV8 capsid ELISA will be determined.

8.4.9. PCR of rAAV8 genome

A search for potential systemic rAAV8 virus genome will be performed at early study visits and later on if a control is deemed necessary by the investigator.

Ophthalmological procedures

All tests described will be performed in a fixed order. The treated eye will be examined first, followed by the untreated eye.

8.4.10. Best corrected visual acuity

Best corrected visual acuity will be quantified using the ETDRS visual acuity protocol as described in the Procedure Manual.

8.4.11. Basic ophthalmological examination

After determination of visual acuity, outer eye segments will be inspected and the anterior segments of the eye will be examined using a slit-lamp.

8.4.12. Contrast sensitivity (Pelli Robson Charts)

This test for contrast sensitivity will be performed in both eyes at standardized illumination conditions, according to the Procedure Manual of the Pelli Robson (PR) chart. In contrast to the user instruction of the PR chart, the test distance will be 3m (instead of 1m), as own data have shown, that the standard distance of 1m is inferior in sensitivity.

8.4.13. Flicker Fusion Frequency

The Flicker Fusion Frequency is used to test cone function independent of selective amblyopia problems eventually present. The subject increases flicker frequency until disappearance of flicker sensation.

8.4.14. Colour constancy

Patients are asked to judge the chromatic appearance of a hexagonal test patch within a multi-coloured display at photopic levels. More details are described in the Procedure Manual.

8.4.15. Anomaloscopy

The Nagel anomaloscope can differentiate between six pathological findings i.e. colour deficiencies and will be used in the trial to investigate baseline and possible restitution of colour discrimination. The Rayleigh-equation quantitatively evaluates results.

8.4.16. Cambridge Colour Test

Chromatic discrimination thresholds will be determined using the low-vision version of the Cambridge Colour Test as described in more detail in the Procedure Manual. The stimulus array consists of discrete dots of variable random size and luminance. The target is a subset of dots, which delineate a large 5-degree square varying in saturation. Patients indicate the location of the coloured square in an alternative forced choice procedure.

8.4.17. Infrared-Video-Pupillography

Pupillography is used for the objective efficacy assessment after intervention. Chromatic Pupillography will be performed at various time-points during the study as outlined in the flowchart of visits. Standardized adaptation, background illuminance, stimulus wavelength and duration need to be considered according to the Procedure Manual. Consensual measurement is performed, i.e. study eye is always stimulated while pupillary reactions of the fellow eye are recorded monocularly.

8.4.18. Microperimetry

Microperimetry tests retinal sensitivity by projecting defined light stimuli to specific locations within the macula under mesopic conditions. The subjects are asked to press a button when a stimulus is perceived. Involuntary eye movements are corrected in real-time and fixation stability is quantified. This test will be performed according to the Procedure Manual.

Note: All following tests will be performed in pharmacologically induced mydriasis (2.5% phenylephrin, 0.5% tropicamid eye drops) only.

8.4.19. Fundoscopy

Stereoscopic biomicroscopy of the full fundus is performed using a 90 Dpt lens (Volk) or equivalent to check for signs of inflammation and retinal detachment.

8.4.20. Dark adaptation (DA)

After 3 minutes of bleaching with bright white light (pupil dilation), a staircase procedure is used to estimate detection thresholds for red and green. The whole measurement lasts for approx. 40 minutes.

8.4.21. Ganzfeld electroretinogram (ERG)

The Ganzfeld electroretinogram (ERG) is the record of diffuse electrical response to light stimuli, generated by neural and non-neural cells in the retina. Depending on recording conditions (i.e. scotopic or photopic), stimulus intensity, wavelength, frequency or stimulus duration, the function of various retinal cells can be isolated. Further details of the test procedures are described in the Procedure Manual.

8.4.22. Spectral domain optical coherence tomography (SD-OCT)

Patients will be examined in mydriasis using a Spectralis HRT+OCT system (Heidelberg Engineering, Heidelberg, Germany). Briefly, perpendicular line and volume scans are recorded to quantify micro-anatomical changes in the macula before (baseline) and after (follow up) treatment. The patient is asked to look at a visual cue while eye-tracking software will correct for involuntary eye movement. Baseline recordings will be identified to allow precise follow-up recordings based on the same anatomical landmarks used for eye-tracking. The untreated eye will be recorded first, followed by the treated one.

8.4.23. Fundus autofluorescence (FAF)

The Spectralis HRT+OCT system described above will be used in the same session to record FAF, a measure of retinal pigment epithelial metabolism and viability (Schmitz-Valckenberg et al., 2008). Central 55° recordings will be made with optional extensions towards the outer limits of the treated area, if they are not covered by the central recordings. The untreated eye will be recorded first, followed by the treated one.

8.4.24. Fundus photography (FP)

Fundus of all patients will be recorded in mydriasis for documentation of posterior segment changes. This includes 9 separate wide-angle recordings (central, superior,

superior-nasal, nasal, nasal-inferior, inferior, temporal-inferior, temporal, temporal-superior). The untreated eye will be recorded first, followed by the treated one.

8.4.25. Angiography

ICG Angiography

Indocyanine Green is a dye, which fluoresces in the infra-red light. The infra-red waves have the ability to penetrate the retinal layers making the circulation in deeper layers visible when photographed with an infra-red sensitive camera. The dye is applied by intravenous injection and flows through the body to reach the choroidal and retinal circulation. Due to its nature stays in the retinal and choroidal vessels, this allows to see and identify the distinct outlines of the vessels of the choroid.

Fluorescein Angiography (FA)

Perfusion characteristics will be assessed in mydriasis using a wide-angle objective after intravenous bolus administration of 10% fluorescein sodium (500mg in 5ml; Novartis Pharma AG, Bern, Switzerland).

The central fundus of the treated eye will be recorded with sequential photographs in the first 45 seconds after injection. At 1 and 5 minutes after injection the same 9-view recordings will be performed in both eyes.

8.4.26. Topical steroids and antibiotics

Moxifloxacin eye drops 0.5% are administered 4 times a day and dexamethasone gel 0.5% is administered 4 times a day for 21 days, starting at day -1.

8.4.27. Systemic steroids

Systemic steroids will be given orally at 1.0mg/kg for approximately 3 weeks starting at day -1 and then tapered off after day 19 at the discretion of the investigator (e.g 40/30/20/15/10mg).

8.4.28. Subretinal injection of rAAV8.hCNGA3 vector

The study treatment is described in section 6 briefly and in detail in the Procedure Manual.

8.4.29. VFQ25

The NEI-VFQ-25 is a 25-item vision-specific questionnaire that collects information about how the patient’s vision affects his/her life in 3 areas (General Health and Vision, Difficulty with Activities, Responses to Vision Problems). The NEI-VFQ-25 assesses the influence of visual impairment on functioning and specific aspects of

health-related quality of life. The interviewer-administered version of the NEIVFQ- 25 will be used for all patients and will be interview-administered by the study site.

8.4.30. Psychiatric examination

A basic psychiatric examination is performed at screening to ensure that the patient does not suffer from a psychiatric disorder and is not at risk for recurrence of such a disorder. Patients should not be under high pressure due to achromatopsia and/or have high and unrealistic expectations regarding the treatment outcome.

8.4.31. Brief symptom inventory (BSI)

The brief symptom inventory assesses psychological stress and is administered as a questionnaire in interview version. It will be performed by a psychologist or a psychiatrist.

8.4.32. Psychological counselling

The patient has the possibility of psychological counselling with a specialist who does not belong to the study team.

8.4.33. Study specific scale, A3-PRO

In addition to the widely used VFQ25 (see above), which may not be sensitive for the treatment effects in the study population, a study-specific scale was developed in an interview version to be used at selected study visits. A3-PRO assesses the subjectively perceived effects of the treatment.

8.4.34. Adverse event (AE) recording

Open questions regarding changes in health status since last visit are asked by the study physician at the beginning of each study visit.

8.4.35. Concomitant medication

At each visit changes in dosing / medication are recorded by the study staff.

9. Documentation of Trial Data

All original study data from all screened and enrolled patients will be entered into the electronical study database. Original hardcopy documents will need to be signed and dated. GCP-conform monitoring is performed by the CRO of the study as agreed in the monitor manual with the principal investigator on behalf of the sponsor. Any

correction on study documents needs to be initialized and dated. All handwriting must be readable. Incorrect entries must still be readable but striked out.

10. Biometrical Planning and Analysis

10.1. Trial Design

This is an open, mono-center trial with fellow-eye comparison.
Due to the characteristics of a phase I trial a dose-escalation design was chosen and due to the fact that there is no effective, approved therapy and this is a safety trial no control condition is foreseen. As a consequence of this, there will be no blinding / randomization procedure. The development of visual function and morphology will be tested against the fellow-eye. Taking this in account gross asymmetry of the disease between both eyes is an exclusion criterion.
The small sample size, as well as the particularity and rareness of the disease CNGA3-related achromatopsia, led to a monocentric study concept.

10.2. Sample Size Issues

No statistical sample size estimation was performed as no data are available regarding the safety of rAAV.hCNGA3 (first dose in man) or any other effect in the eye for such a calculation. As three vector dosages are planned in a dose-escalating schedule we decided for three patients in each group. Drop-outs will only be replaced up to a number of two patients in case that other reasons than vector- or procedure-related adverse events are the underlying reasons for the drop-out.

10.3. Definition for Study Groups

Study populations are defined in the following subsections. All populations will be identified and finalized in the Statistical Analysis Plan.

10.3.1. Safety Population

The Safety Population is defined as all subjects who receive at least one dose of investigational product or corticosteroids and have at least one post-therapy safety assessment.

10.3.2. Intent to Treat Population

The Intent to Treat (ITT) population is defined as all subjects who are enrolled and received at least one dose of investigational product or corticosteroids, and for whom at least one post-baseline assessment is available.

10.3.3. Per Protocol Population

The Per Protocol (PP) Population is a subset of the ITT Population and consists of subjects for whom no major protocol violations have been reported. The criteria for defining the PP set will be fully defined in the Statistical Analysis Plan.

A final decision for the allocation to the different populations of the subjects will be made before analysis of trial results.

10.4. Statistical Analysis

AEs and SAEs will be documented using line listings and tabulations (MetraCad code will be applied). Descriptive analysis will include line charts for individual patients. Standard errors and means will be displayed also using line charts. This will be done for the raw measurements and for the difference between treated and untreated control eye within each patient.
Descriptive parameters will be given for the full cohort (n=9) but not for the sub-cohorts (n=3).
Additionally correlation analysis will be performed in a purely descriptive manner for the comparison of subjective and objective measurements. For each subject and measurement, the delta of the value for the interventional eye minus the mean of control eye values over time will be determined. In scatter plots for pairs of variables (measurements pooled over subjects) the association between these deltas will be displayed.

10.5. Interim Analyses

Beyond continuous Safety Monitoring by the DMC no interim analysis in a closer sense is foreseen.

11. Anticonception Rules and Pregnancy

Experiences regarding transfection to the germline are available from application of similar AAV vectors.
Preclinical Data. Systemic application - predominantly in rodents - showed no transfection of the germ line (Alemany and Curiel, 2001; Peng et al., 2001; Peters et al., 2001; Gonin and Gaillard, 2004). Also for systemic application, Arruda et al. (Arruda et al., 2001) detected dose-related AAV vector sequences in DNA extracted

from rabbit in testes, but not in semen. In addition, in vitro attempts to directly infect isolated murine spermatogonia with a rAAV vector failed (Arruda et al., 2001). Schuettrumpf et al. (Schuettrumpf et al., 2006) - investigating effects in the rabbit to doses of $1x10^{11}$ – 10^{13} vg/kg i.v. – found transient vector dissemination to semen until day 4 post application, while long-term follow-up did not reveal any transduction of the spermatogenesis. Favaro et al. (Favaro et al., 2009) used doses of $1x10^{12}$ – 10^{13} vg/kg and detected virus genome until week 10 post injection (rabbit). In analyses in rabbits after intravenous rAAV doses ranging from 10e11 to 10e13 vector genomes/kg (doses similar to those used in a human clinical trial), a dose-dependent increase in PCR positive vector sequences was seen in semen samples; however, at no timepoint infectious AAV particles could be recovered from the semen samples (Ariuda et al., 2003).

When exposing the germline of rodents to AAV vectors directly several investigators did not find transfection of germ cells nor of foetuses in the case of test-tube fertilization (Gordon, 2001; Couto et al., 2004; Kojima et al., 2008). Laurema et al. (Laurema et al., 2003) exposed pregnant rabbits to AAV vectors (arteria uterina, $1x10^{10}$ vgp) and found that premature egg cells (without zona pellucida) were transfected 3 respective 8 days after gene therapy.

Clinical Data. Experiences in humans are available for non-ocular applications of AAV vectors. After i.v. injection with a twenty times higher viral load compared to our planned trial, only non-infectious particles were found in semen until 16 weeks after treatment. If vectors had been applied intramuscular, no AAV virus genome was present in semen two months and four months respectively after injection (Kay et al., 2000; Manno et al., 2006). The trials cited above show that in all administration types, except intravenous delivery, no AAV genome could be found in semen samples after Day 7 after administration. None of the available ongoing clinical trials (see introduction) with ocular gene therapy have investigated rAAV shedding in the semen. No data has been published regarding germline transfection from other ocular gene therapy studies (neither preclinical nor clinical). In a personal communication with Jean Bennett, Philadelphia, working with gene therapy in LCA patients our group was informed that there was no evidence of exposure to the reproductive track after subretinal application of a rAAV2 vector.

We therefore deem the need for contraception by female or male study participants with fertile partners for four months after the injection to be sufficient. Beyond the consideration of a desirable safety margin on one hand and avoidance of unnecessary constraints for the individual patient’s life on the other, this time span is also in line with other ophthalmological gene therapy trials, such as in Leber’s congenital amaurosis caused by RPE65 mutation, approved by the FDA.

13. Conditions for Amendments

In case of necessary changes to the flowchart of visits and procedures or other aspects of the trial, an amendment will be submitted to PEI and EC. Those changes will not be implemented unless approved by the PEI and EC. Exempted are changes to the protocol preventing immediate hazard for the patient. In this case

implementation may occur before approval. Any amendment will result in a revised version of the patient information form, which needs to be signed by all patients.
A recommendation of the DMC may also result in an amendment to the trial protocol.

14. Ethical and Regulatory Aspects

The Investigator will be responsible for the overall conduct of the clinical trial and will be responsible for ensuring the trial is conducted according to the protocol and all regulatory requirements and regulations.

The protocol and informed consent form for this study must be approved by an appropriately constituted EC as defined by local requirements. The list of the EC voting members, their titles or occupation, and their institutional affiliations and/or the EC general assurance number, if applicable, must be provided with the approval.
The EC will also be notified of completion of the study and a final report must be submitted to the EC and the PEI in accordance with local requirements. The Investigator will maintain an accurate and complete record of all communications, reports and submissions to the PEI and EC.

This trial is subject to German AMG in its current version and will be submitted, performed and monitored according to ICH-GCP standards. Any study procedure will only be performed after the patient has given written informed consent. Informed consent will be documented in the electronic patient file of the Centre for Ophthalmology (AIS) giving the date of consent, study name and the name of the physician. Each subject's signed informed consent must be kept on file by the Investigator.
German data protection regulations will be followed and patients will be informed in a standardized and detailed manner about pseudonomyzed health data and about those persons/institutions who will have access to personal data under special restrictions according to law.

The Sponsor of this trial is UKT (Universitätsklinikum Tübingen) represented by the Principal Investigator. Defined tasks have been delegated to the CRO of the trial, STZ eyetrial at the Centre for Ophthalmology, University of Tübingen such as, but not limited to, communication with regulatory authorities, submission of the trial to PEI and EC, monitoring of the trial, safety reports. UKT has contracted insurance for trial participation as well as travel insurance.

The contact information for **patient insurance** are:

[redacted]

[redacted]

Contact information for **travel insurance** are:

[redacted]

The amount covered for the patient insurance is 500 000€ per patient. The amount covered for the travel insurance is 100 000€ for invalidity und 50 000€ in the case of death.

The patient has the obligation to immediately inform the insurance companies mentioned above, if necessary with the support of the Investigator. The patient also has to inform the investigator

All patients will receive a copy of the insurance police, along with the copy of the patient informed consent.

The documents used for submission and conduct of this trial will be according to the SOP system of the local CRO, STZ *eyetrial* at the Centre for Ophthalmology, which is a certified member of the EVICR.net.

15. References

Aboshiha, J., A. M. Dubis, J. Cowing, R. T. Fahy, V. Sundaram, J. W. Bainbridge, R. R. Ali, A. Dubra, M. Nardini, A. R. Webster, A. T. Moore, G. Rubin, J. Carroll and M. Michaelides (2014). A prospective longitudinal study of retinal structure and function in achromatopsia. *Invest Ophthalmol Vis Sci*, 55: 5733-5743

Alemany, R. and D. T. Curiel (2001). CAR-binding ablation does not change biodistribution and toxicity of adenoviral vectors. *Gene Ther*, 8: 1347-1353

Aligianis, I. A., T. Forshew, S. Johnson, M. Michaelides, C. A. Johnson, R. C. Trembath, D. M. Hunt, A. T. Moore and E. R. Maher (2002). Mapping of a novel locus for achromatopsia (ACHM4) to 1p and identification of a germline mutation in the alpha subunit of cone transducin (GNAT2). *J Med Genet*, 39: 656-660

Allocca, M., C. Mussolino, M. Garcia-Hoyos, D. Sanges, C. Iodice, M. Petrillo, L. H. Vandenberghe, J. M. Wilson, V. Marigo, E. M. Surace and A. Auricchio (2007). Novel

adeno-associated virus serotypes efficiently transduce murine photoreceptors. *J Virol*, 81: 11372-11380

Ariuda, V. R., J. Schuettrumpf, L. JiangHua, K. Addya, D. Leonard, L. Couto, A. Chew, Z. Zhen, J. Sommer, R. W. Herzog, M. A. Kay, G. Bert, C. S. Manno and K. A. High (2003). Assessing the risk of inadvertent germline transmission of recombinant AAV-2 vector. . *Molecular Therapy*, 7: 161-162

Arruda, V. R., P. A. Fields, R. Milner, L. Wainwright, M. P. De Miguel, P. J. Donovan, R. W. Herzog, T. C. Nichols, J. A. Biegel, M. Razavi, M. Dake, D. Huff, A. W. Flake, L. Couto, M. A. Kay and K. A. High (2001). Lack of germline transmission of vector sequences following systemic administration of recombinant AAV-2 vector in males. *Mol Ther*, 4: 586-592

Asokan, A., D. V. Schaffer and R. J. Samulski (2012). The AAV vector toolkit: poised at the clinical crossroads. *Mol Ther*, 20: 699-708

Bainbridge, J. W., A. J. Smith, S. S. Barker, S. Robbie, R. Henderson, K. Balaggan, A. Viswanathan, G. E. Holder, A. Stockman, N. Tyler, S. Petersen-Jones, S. S. Bhattacharya, A. J. Thrasher, F. W. Fitzke, B. J. Carter, G. S. Rubin, A. T. Moore and R. R. Ali (2008). Effect of gene therapy on visual function in Leber's congenital amaurosis. *N Engl J Med*, 358: 2231-2239

Barnard, A. R., M. Groppe and R. E. MacLaren (2014). Gene Therapy for Choroideremia Using an Adeno-Associated Viral (AAV) Vector. *Cold Spring Harb Perspect Med*, 4:

Bennett, J., M. Ashtari, J. Wellman, K. A. Marshall, L. L. Cyckowski, D. C. Chung, S. McCague, E. A. Pierce, Y. Chen, J. L. Bennicelli, X. Zhu, G. S. Ying, J. Sun, J. F. Wright, A. Auricchio, F. Simonelli, K. S. Shindler, F. Mingozzi, K. A. High and A. M. Maguire (2012). AAV2 gene therapy readministration in three adults with congenital blindness. *Sci Transl Med*, 4: 120ra115

Bertelsen, M., H. Jensen, M. Larsen, B. Lorenz, M. N. Preising and T. Rosenberg (2013). Prevalence and diagnostic spectrum of generalized retinal dystrophy in Danish children. *Ophthalmic Epidemiol*, 20: 164-169

Black, A., V. Vasireddy, D. C. Chung, A. M. Maguire, R. Gaddameedi, T. Tolmachova, M. Seabra and J. Bennett (2014). Adeno-associated virus 8-mediated gene therapy for choroideremia: preclinical studies in in vitro and in vivo models. *J Gene Med*, 16: 122-130

Blackwell, H. R. and O. M. Blackwell (1961). Rod and cone receptor mechanisms in typical and atypical congenital achromatopsia. *Vision Research*, 1: 62-107

Burgueno-Montanes, C., M. Colunga Cueva and C. Costales Alvarez (2014). A novel mutation in the CNGA3 gene responsible for incomplete achromatopsia. *Arch Soc Esp Oftalmol*, 89: 107-109

Chang, B., T. Grau, S. Dangel, R. Hurd, B. Jurklies, E. C. Sener, S. Andreasson, H. Dollfus, B. Baumann, S. Bolz, N. Artemyev, S. Kohl, J. Heckenlively and B. Wissinger (2009). A homologous genetic basis of the murine cpfl1 mutant and human achromatopsia linked to mutations in the PDE6C gene. *Proc Natl Acad Sci U S A*, 106: 19581-19586

Cideciyan, A. V., S. G. Jacobson, W. A. Beltran, A. Sumaroka, M. Swider, S. Iwabe, A. J. Roman, M. B. Olivares, S. B. Schwartz, A. M. Komaromy, W. W. Hauswirth and G. D. Aguirre (2013). Human retinal gene therapy for Leber congenital amaurosis shows advancing retinal degeneration despite enduring visual improvement. *Proc Natl Acad Sci U S A*:

Couto, L., A. Parker and J. W. Gordon (2004). Direct exposure of mouse spermatozoa to very high concentrations of a serotype-2 adeno-associated virus gene therapy vector fails to lead to germ cell transduction. *Hum Gene Ther*, 15: 287-291

Curcio, C. A., K. R. Sloan, R. E. Kalina and A. E. Hendrickson (1990). Human photoreceptor topography. *J Comp Neurol*, 292: 497-523

Favaro, P., H. D. Downey, J. S. Zhou, J. F. Wright, B. Hauck, F. Mingozzi, K. A. High and V. R. Arruda (2009). Host and vector-dependent effects on the risk of germline transmission of AAV vectors. *Mol Ther*, 17: 1022-1030

Ferreira, V., H. Petry and F. Salmon (2014a). Immune Responses to AAV-Vectors, the Glybera Example from Bench to Bedside. *Front Immunol*, 5: 82

Ferreira, V., J. Twisk, K. Kwikkers, E. Aronica, D. Brisson, J. Methot, H. Petry and D. Gaudet (2014b). Immune responses to intramuscular administration of alipogene tiparvovec (AAV1-LPL(S447X)) in a phase II clinical trial of lipoprotein lipase deficiency gene therapy. *Hum Gene Ther*, 25: 180-188

Flanagan, N. M., A. J. Jackson and A. E. Hill (2003). Visual impairment in childhood: insights from a community-based survey. *Child Care Health Dev*, 29: 493-499

Gonin, P. and C. Gaillard (2004). Gene transfer vector biodistribution: pivotal safety studies in clinical gene therapy development. *Gene Ther*, 11 Suppl 1: S98-S108

Gordon, J. W. (2001). Direct exposure of mouse ovaries and oocytes to high doses of an adenovirus gene therapy vector fails to lead to germ cell transduction. *Mol Ther*, 3: 557-564

Greenberg, J. P., J. Sherman, S. A. Zweifel, R. W. Chen, T. Duncker, S. Kohl, B. Baumann, B. Wissinger, L. A. Yannuzzi and S. H. Tsang (2014). Spectral-Domain Optical Coherence Tomography Staging and Autofluorescence Imaging in Achromatopsia. *JAMA Ophthalmol*:

Hauswirth, W. W., T. S. Aleman, S. Kaushal, A. V. Cideciyan, S. B. Schwartz, L. Wang, T. J. Conlon, S. L. Boye, T. R. Flotte, B. J. Byrne and S. G. Jacobson (2008). Treatment of leber congenital amaurosis due to RPE65 mutations by ocular subretinal injection of adeno-associated virus gene vector: short-term results of a phase I trial. *Hum Gene Ther*, 19: 979-990

Huckfeldt, R. M. and J. Bennett (2014). Promising first steps in gene therapy for choroideremia. *Hum Gene Ther*, 25: 96-97

Jacobson, S. G., A. V. Cideciyan, R. Ratnakaram, E. Heon, S. B. Schwartz, A. J. Roman, M. C. Peden, T. S. Aleman, S. L. Boye, A. Sumaroka, T. J. Conlon, R. Calcedo, J. J. Pang, K. E. Erger, M. B. Olivares, C. L. Mullins, M. Swider, S. Kaushal, W. J. Feuer, A. Iannaccone, G. A. Fishman, E. M. Stone, B. J. Byrne and W. W. Hauswirth (2012). Gene therapy for leber congenital amaurosis caused by RPE65 mutations: safety and efficacy in 15 children and adults followed up to 3 years. *Arch Ophthalmol*, 130: 9-24

Jeon, C. J., E. Strettoi and R. H. Masland (1998). The major cell populations of the mouse retina. *J Neurosci*, 18: 8936-8946

Kay, M. A., C. S. Manno, M. V. Ragni, P. J. Larson, L. B. Couto, A. McClelland, B. Glader, A. J. Chew, S. J. Tai, R. W. Herzog, V. Arruda, F. Johnson, C. Scallan, E. Skarsgard, A. W. Flake and K. A. High (2000). Evidence for gene transfer and expression of factor IX in haemophilia B patients treated with an AAV vector. *Nat Genet*, 24: 257-261

Kohl, S., B. Baumann, M. Broghammer, H. Jagle, P. Sieving, U. Kellner, R. Spegal, M. Anastasi, E. Zrenner, L. T. Sharpe and B. Wissinger (2000). Mutations in the CNGB3 gene encoding the beta-subunit of the cone photoreceptor cGMP-gated channel are responsible for achromatopsia (ACHM3) linked to chromosome 8q21. *Hum Mol Genet*, 9: 2107-2116

Kohl, S., B. Baumann, T. Rosenberg, U. Kellner, B. Lorenz, M. Vadala, S. G. Jacobson and B. Wissinger (2002). Mutations in the cone photoreceptor G-protein alpha-subunit gene GNAT2 in patients with achromatopsia. *Am J Hum Genet*, 71: 422-425

Kohl, S., F. Coppieters, F. Meire, S. Schaich, S. Roosing, C. Brennenstuhl, S. Bolz, M. M. van Genderen, F. C. Riemslag, C. European Retinal Disease, R. Lukowski, A. I. den Hollander, F. P. Cremers, E. De Baere, C. B. Hoyng and B. Wissinger (2012). A nonsense mutation in PDE6H causes autosomal-recessive incomplete achromatopsia. *Am J Hum Genet*, 91: 527-532

Kohl, S., T. Marx, I. Giddings, H. Jagle, S. G. Jacobson, E. Apfelstedt-Sylla, E. Zrenner, L. T. Sharpe and B. Wissinger (1998). Total colourblindness is caused by mutations in the gene encoding the alpha-subunit of the cone photoreceptor cGMP-gated cation channel. *Nat Genet*, 19: 257-259

Kohl, S., B. Varsanyi, G. A. Antunes, B. Baumann, C. B. Hoyng, H. Jagle, T. Rosenberg, U. Kellner, B. Lorenz, R. Salati, B. Jurklies, A. Farkas, S. Andreasson, R. G. Weleber, S. G. Jacobson, G. Rudolph, C. Castellan, H. Dollfus, E. Legius, M. Anastasi, P. Bitoun, D. Lev, P. A. Sieving, F. L. Munier, E. Zrenner, L. T. Sharpe, F. P. Cremers and B. Wissinger (2005). CNGB3 mutations account for 50% of all cases with autosomal recessive achromatopsia. *Eur J Hum Genet*, 13: 302-308

Kojima, Y., Y. Hayashi, S. Kurokawa, K. Mizuno, S. Sasaki and K. Kohri (2008). No evidence of germ-line transmission by adenovirus-mediated gene transfer to mouse testes. *Fertil Steril*, 89: 1448-1454

Laurema, A., A. Heikkila, L. Keski-Nisula, T. Heikura, P. Lehtolainen, H. Manninen, T. T. Tuomisto, S. Heinonen and S. Yla-Herttuala (2003). Transfection of oocytes and other types of ovarian cells in rabbits after direct injection into uterine arteries of adenoviruses and plasmid/liposomes. *Gene Ther*, 10: 580-584

Lebherz, C., A. Maguire, W. Tang, J. Bennett and J. M. Wilson (2008). Novel AAV serotypes for improved ocular gene transfer. *J Gene Med*, 10: 375-382

Lee, G. W., S. J. Na, Y. H. Lee, S. Y. Jin and T. G. Lee (2012). Complication Incidence of Day Surgeries with 23 Gauge Vitrectomy. *J Korean Ophthalmol Soc*, 53: 1823-1827

Li, A., X. Zhu and C. M. Craft (2002). Retinoic acid upregulates cone arrestin expression in retinoblastoma cells through a Cis element in the distal promoter region. *Invest Ophthalmol Vis Sci*, 43: 1375-1383

Li, Q., R. Miller, P. Y. Han, J. Pang, A. Dinculescu, V. Chiodo and W. W. Hauswirth (2008). Intraocular route of AAV2 vector administration defines humoral immune response and therapeutic potential. *Mol Vis*, 14: 1760-1769

Lipinski, D. M., M. Thake and R. E. MacLaren (2013). Clinical applications of retinal gene therapy. *Prog Retin Eye Res*, 32: 22-47

Liu, H. S., M. S. Jan, C. K. Chou, P. H. Chen and N. J. Ke (1999). Is green fluorescent protein toxic to the living cells? *Biochem Biophys Res Commun*, 260: 712-717

Liu, M. M., J. Tuo and C. C. Chan (2011). Gene therapy for ocular diseases. *Br J Ophthalmol*, 95: 604-612

Maclaren, R. E., M. Groppe, A. R. Barnard, C. L. Cottriall, T. Tolmachova, L. Seymour, K. R. Clark, M. J. During, F. P. Cremers, G. C. Black, A. J. Lotery, S. M. Downes, A. R. Webster and M. C. Seabra (2014). Retinal gene therapy in patients with choroideremia: initial findings from a phase 1/2 clinical trial. *Lancet*:

Maguire, A. M., K. A. High, A. Auricchio, J. F. Wright, E. A. Pierce, F. Testa, F. Mingozzi, J. L. Bennicelli, G. S. Ying, S. Rossi, A. Fulton, K. A. Marshall, S. Banfi, D. C. Chung, J. I. Morgan, B. Hauck, O. Zelenaia, X. Zhu, L. Raffini, F. Coppieters, E. De Baere, K. S. Shindler, N. J. Volpe, E. M. Surace, C. Acerra, A. Lyubarsky, T. M. Redmond, E. Stone, J. Sun, J. W. McDonnell, B. P. Leroy, F. Simonelli and J. Bennett (2009). Age-dependent effects of RPE65 gene therapy for Leber's congenital amaurosis: a phase 1 dose-escalation trial. *Lancet*, 374: 1597-1605

Maguire, A. M., F. Simonelli, E. A. Pierce, E. N. Pugh, Jr., F. Mingozzi, J. Bennicelli, S. Banfi, K. A. Marshall, F. Testa, E. M. Surace, S. Rossi, A. Lyubarsky, V. R. Arruda, B. Konkle, E. Stone, J. Sun, J. Jacobs, L. Dell'Osso, R. Hertle, J. X. Ma, T. M. Redmond, X. Zhu, B. Hauck, O. Zelenaia, K. S. Shindler, M. G. Maguire, J. F. Wright, N. J. Volpe, J. W. McDonnell, A. Auricchio, K. A. High and J. Bennett (2008). Safety and efficacy of gene transfer for Leber's congenital amaurosis. *N Engl J Med*, 358: 2240-2248

Manno, C. S., G. F. Pierce, V. R. Arruda, B. Glader, M. Ragni, J. J. Rasko, M. C. Ozelo, K. Hoots, P. Blatt, B. Konkle, M. Dake, R. Kaye, M. Razavi, A. Zajko, J. Zehnder, P. K. Rustagi, H. Nakai, A. Chew, D. Leonard, J. F. Wright, R. R. Lessard, J. M. Sommer, M. Tigges, D. Sabatino, A. Luk, H. Jiang, F. Mingozzi, L. Couto, H. C. Ertl, K. A. High and M. A. Kay (2006). Successful transduction of liver in hemophilia by AAV-Factor IX and limitations imposed by the host immune response. *Nat Med*, 12: 342-347

Marangoni, D., Z. Wu, H. E. Wiley, C. J. Zeiss, C. Vijayasarathy, Y. Zeng, S. Hiriyanna, R. A. Bush, L. L. Wei, P. Colosi and P. A. Sieving (2014). Preclinical Safety Evaluation of a Recombinant AAV8 Vector for X-linked Retinoschisis after Intravitreal Administration in Rabbits. *Hum Gene Ther Clin Dev*:

McClements, M. E. and R. E. MacLaren (2013). Gene therapy for retinal disease. *Transl Res*, 161: 241-254

Michalakis, S., R. L. Muehlfriedel, N. Tanimoto, V. Krishnamoorthy, S. Koch, S. C. Beck, H. Buening, T. Gollisch, M. Biel and M. W. Seeliger (2011). Exploring Different Serotypes And Promoters In rAAV-mediated Gene Replacement Therapy Of Achromatopsia Type 2 (ACHM2). *ARVO Meeting Abstracts*, 52: 490

Michalakis, S., R. Muhlfriedel, N. Tanimoto, V. Krishnamoorthy, S. Koch, M. D. Fischer, E. Becirovic, L. Bai, G. Huber, S. C. Beck, E. Fahl, H. Buning, F. Paquet-Durand, X. Zong, T. Gollisch, M. Biel and M. W. Seeliger (2010). Restoration of cone vision in the CNGA3-/- mouse model of congenital complete lack of cone photoreceptor function. *Mol Ther*, 18: 2057-2063

Michalakis, S., R. Muhlfriedel, N. Tanimoto, V. Krishnamoorthy, S. Koch, M. D. Fischer, E. Becirovic, L. Bai, G. Huber, S. C. Beck, E. Fahl, H. Buning, J. Schmidt, X. Zong, T. Gollisch, M. Biel and M. W. Seeliger (2012). Gene therapy restores missing cone-mediated vision in the CNGA3-/- mouse model of achromatopsia. *Adv Exp Med Biol*, 723: 183-189

Michelfelder, S., K. Varadi, C. Raupp, A. Hunger, J. Korbelin, C. Pahrmann, S. Schrepfer, O. J. Muller, J. A. Kleinschmidt and M. Trepel (2011). Peptide ligands incorporated into the threefold spike capsid domain to re-direct gene transduction of AAV8 and AAV9 in vivo. *PLoS One*, 6: e23101

Mussolino, C., M. della Corte, S. Rossi, F. Viola, U. Di Vicino, E. Marrocco, S. Neglia, M. Doria, F. Testa, R. Giovannoni, M. Crasta, M. Giunti, E. Villani, M. Lavitrano, M. L. Bacci, R. Ratiglia, F. Simonelli, A. Auricchio and E. M. Surace (2011). AAV-mediated photoreceptor transduction of the pig cone-enriched retina. *Gene Ther*, 18: 637-645

Nathwani, A. C., E. G. Tuddenham, S. Rangarajan, C. Rosales, J. McIntosh, D. C. Linch, P. Chowdary, A. Riddell, A. J. Pie, C. Harrington, J. O'Beirne, K. Smith, J. Pasi, B. Glader, P. Rustagi, C. Y. Ng, M. A. Kay, J. Zhou, Y. Spence, C. L. Morton, J. Allay, J. Coleman, S. Sleep, J. M. Cunningham, D. Srivastava, E. Basner-Tschakarjan, F. Mingozzi, K. A. High, J. T. Gray, U. M. Reiss, A. W. Nienhuis and A. M. Davidoff (2011). Adenovirus-associated virus vector-mediated gene transfer in hemophilia B. *N Engl J Med*, 365: 2357-2365

Pang, J. J., X. Dai, S. E. Boye, I. Barone, S. L. Boye, S. Mao, D. Everhart, A. Dinculescu, L. Liu, Y. Umino, B. Lei, B. Chang, R. Barlow, E. Strettoi and W. W. Hauswirth (2011). Long-term retinal function and structure rescue using capsid mutant AAV8 vector in the rd10 mouse, a model of recessive retinitis pigmentosa. *Mol Ther*, 19: 234-242

Park, T. K., Z. Wu, S. Kjellstrom, Y. Zeng, R. A. Bush, P. A. Sieving and P. Colosi (2009). Intravitreal delivery of AAV8 retinoschisin results in cell type-specific gene expression and retinal rescue in the Rs1-KO mouse. *Gene Ther*, 16: 916-926

Penaud-Budloo, M., C. Le Guiner, A. Nowrouzi, A. Toromanoff, Y. Cherel, P. Chenuaud, M. Schmidt, C. von Kalle, F. Rolling, P. Moullier and R. O. Snyder (2008). Adeno-associated virus vector genomes persist as episomal chromatin in primate muscle. *J Virol*, 82: 7875-7885

Peng, K. W., L. Pham, H. Ye, R. Zufferey, D. Trono, F. L. Cosset and S. J. Russell (2001). Organ distribution of gene expression after intravenous infusion of targeted and untargeted lentiviral vectors. *Gene Ther*, 8: 1456-1463

Peters, A. H., J. Drumm, C. Ferrell, D. A. Roth, D. M. Roth, M. McCaman, P. L. Novak, J. Friedman, R. Engler and R. E. Braun (2001). Absence of germline infection in male mice following intraventricular injection of adenovirus. *Mol Ther*, 4: 603-613

Pokorny, J., V. C. Smith, A. J. Pinckers and M. Cozijnsen (1982). Classification of complete and incomplete autosomal recessive achromatopsia. *Graefes Archives for Clinical and Experimental Ophthalmology*, 219: 121-130

Prokofyeva, E., R. Wilke, G. Lotz, E. Troeger, T. Strasser and E. Zrenner (2009). An epidemiological approach for the estimation of disease onset in Central Europe in central and peripheral monogenic retinal dystrophies. *Graefes Arch Clin Exp Ophthalmol*, 247: 885-894

Puech, B., B. Kostrubiec, J. C. Hache and P. François (1961). Epidemiology and prevalence of hereditary retinal dystrophies in the Northern France. *J Fr Ophtalmol* 14: 153-164

Roosing, S., A. A. Thiadens, C. B. Hoyng, C. C. Klaver, A. I. den Hollander and F. P. Cremers (2014). Causes and consequences of inherited cone disorders. *Prog Retin Eye Res*, 42: 1-26

Samulski, R. J., L. S. Chang and T. Shenk (1987). A recombinant plasmid from which an infectious adeno-associated virus genome can be excised in vitro and its use to study viral replication. *J Virol*, 61: 3096-3101

Schmitz-Valckenberg, S., F. G. Holz, A. C. Bird and R. F. Spaide (2008). Fundus autofluorescence imaging: review and perspectives. *Retina*, 28: 385-409

Schnepp, B. C., K. R. Clark, D. L. Klemanski, C. A. Pacak and P. R. Johnson (2003). Genetic fate of recombinant adeno-associated virus vector genomes in muscle. *J Virol*, 77: 3495-3504

Schuettrumpf, J., J. H. Liu, L. B. Couto, K. Addya, D. G. Leonard, Z. Zhen, J. Sommer and V. R. Arruda (2006). Inadvertent germline transmission of AAV2 vector: findings in a rabbit model correlate with those in a human clinical trial. *Mol Ther*, 13: 1064-1073

Simonelli, F., A. M. Maguire, F. Testa, E. A. Pierce, F. Mingozzi, J. L. Bennicelli, S. Rossi, K. Marshall, S. Banfi, E. M. Surace, J. Sun, T. M. Redmond, X. Zhu, K. S. Shindler, G. S. Ying, C. Ziviello, C. Acerra, J. F. Wright, J. W. McDonnell, K. A. High, J. Bennett and A. Auricchio (2010). Gene therapy for Leber's congenital amaurosis is safe and effective through 1.5 years after vector administration. *Mol Ther*, 18: 643-650

Smith, A. J., J. W. Bainbridge and R. R. Ali (2012). Gene supplementation therapy for recessive forms of inherited retinal dystrophies. *Gene Ther*, 19: 154-161

Streilein, J. W. (2003). Ocular immune privilege: therapeutic opportunities from an experiment of nature. *Nat Rev Immunol*, 3: 879-889

Sundaram, V., C. Wilde, J. Aboshiha, J. Cowing, C. Han, C. S. Langlo, R. Chana, A. E. Davidson, P. I. Sergouniotis, J. W. Bainbridge, R. R. Ali, A. Dubra, G. Rubin, A. R. Webster, A. T. Moore, M. Nardini, J. Carroll and M. Michaelides (2014). Retinal structure and function in achromatopsia: implications for gene therapy. *Ophthalmology*, 121: 234-245

Sundin, O. H., J. M. Yang, Y. Li, D. Zhu, J. N. Hurd, T. N. Mitchell, E. D. Silva and I. H. Maumenee (2000). Genetic basis of total colourblindness among the Pingelapese islanders. *Nat Genet*, 25: 289-293

Tan, M. H., A. J. Smith, B. Pawlyk, X. Xu, X. Liu, J. B. Bainbridge, M. Basche, J. McIntosh, H. V. Tran, A. Nathwani, T. Li and R. R. Ali (2009). Gene therapy for retinitis pigmentosa and Leber congenital amaurosis caused by defects in AIPL1: effective rescue of mouse models

of partial and complete Aipl1 deficiency using AAV2/2 and AAV2/8 vectors. *Hum Mol Genet*, 18: 2099-2114

Testa, F., A. M. Maguire, S. Rossi, E. A. Pierce, P. Melillo, K. Marshall, S. Banfi, E. M. Surace, J. Sun, C. Acerra, J. F. Wright, J. Wellman, K. A. High, A. Auricchio, J. Bennett and F. Simonelli (2013). Three-Year Follow-up after Unilateral Subretinal Delivery of Adeno-Associated Virus in Patients with Leber Congenital Amaurosis Type 2. *Ophthalmology*, 120: 1283-1291

Thiadens, A. A., A. I. den Hollander, S. Roosing, S. B. Nabuurs, R. C. Zekveld-Vroon, R. W. Collin, E. De Baere, R. K. Koenekoop, M. J. van Schooneveld, T. M. Strom, J. J. van Lith-Verhoeven, A. J. Lotery, N. van Moll-Ramirez, B. P. Leroy, L. I. van den Born, C. B. Hoyng, F. P. Cremers and C. C. Klaver (2009). Homozygosity mapping reveals PDE6C mutations in patients with early-onset cone photoreceptor disorders. *Am J Hum Genet*, 85: 240-247

Thiadens, A. A., V. Somervuo, L. I. van den Born, S. Roosing, M. J. van Schooneveld, R. W. Kuijpers, N. van Moll-Ramirez, F. P. Cremers, C. B. Hoyng and C. C. Klaver (2010). Progressive loss of cones in achromatopsia: an imaging study using spectral-domain optical coherence tomography. *Invest Ophthalmol Vis Sci*, 51: 5952-5957

Vandenberghe, L. H., P. Bell, A. M. Maguire, C. N. Cearley, R. Xiao, R. Calcedo, L. Wang, M. J. Castle, A. C. Maguire, R. Grant, J. H. Wolfe, J. M. Wilson and J. Bennett (2011). Dosage thresholds for AAV2 and AAV8 photoreceptor gene therapy in monkey. *Sci Transl Med*, 3: 131-139

Wilkinson, J. T., A. B. Richards, D. Choi, J. E. Robertson, Jr. and C. J. Flaxel (2013). Incidence of retinal detachment after fellow-performed primary pars plana vitrectomy. *ISRN Ophthalmol*, 2013: 353209

Willett, K. and J. Bennett (2013). Immunology of AAV-Mediated Gene Transfer in the Eye. *Front Immunol*, 4: 261

Wissinger, B., D. Gamer, H. Jagle, R. Giorda, T. Marx, S. Mayer, S. Tippmann, M. Broghammer, B. Jurklies, T. Rosenberg, S. G. Jacobson, E. C. Sener, S. Tatlipinar, C. B. Hoyng, C. Castellan, P. Bitoun, S. Andreasson, G. Rudolph, U. Kellner, B. Lorenz, G. Wolff, C. Verellen-Dumoulin, M. Schwartz, F. P. Cremers, E. Apfelstedt-Sylla, E. Zrenner, R. Salati, L. T. Sharpe and S. Kohl (2001). CNGA3 mutations in hereditary cone photoreceptor disorders. *Am J Hum Genet*, 69: 722-737

Wissinger, B., F. Muller, I. Weyand, S. Schuffenhauer, S. Thanos, U. B. Kaupp and E. Zrenner (1997). Cloning, chromosomal localization and functional expression of the gene encoding the alpha-subunit of the cGMP-gated channel in human cone photoreceptors. *Eur J Neurosci*, 9: 2512-2521

Wykoff, C. C., M. B. Parrott, H. W. Flynn, Jr., W. Shi, D. Miller and E. C. Alfonso (2010). Nosocomial acute-onset postoperative endophthalmitis at a university teaching hospital (2002-2009). *Am J Ophthalmol*, 150: 392-398 e392

Yang, P., K. V. Michaels, R. J. Courtney, Y. Wen, D. A. Greninger, L. Reznick, D. J. Karr, L. B. Wilson, R. G. Weleber and M. E. Pennesi (2014). Retinal Morphology of Patients With Achromatopsia During Early Childhood: Implications for Gene Therapy. *JAMA Ophthalmol*:

Zanta-Boussif, M. A., S. Charrier, A. Brice-Ouzet, S. Martin, P. Opolon, A. J. Thrasher, T. J. Hope and A. Galy (2009). Validation of a mutated PRE sequence allowing high and sustained transgene expression while abrogating WHV-X protein synthesis: application to the gene therapy of WAS. *Gene Ther*, 16: 605-619

16. Appendices

Appendix 1

Rationale for vector & dosing

1. Vector

1.1 Safety

Three landmark clinical trials (NCT00516477, NCT00481546 and NCT00643747) have first shown safety and evidence of efficacy using recombinant adeno-associated viral (rAAV) vectored gene therapy in the eye (Bainbridge et al., 2008; Hauswirth et al., 2008; Maguire et al., 2008; Jacobson et al., 2012). In these three studies, a different form of retinal degeneration caused by mutations in the gene encoding RPE65 was treated by subretinal injections of rAAV2/2 vector solutions without a single serious adverse event (SAE) being reported A subset of patients even demonstrated some degree of improved visual function in the untreated eyes.

A phase 1 trial with 12 LCA patients confirms the safety and efficacy of the method, also after re-administration to the second eye (Maguire et al., 2009; Simonelli et al., 2010; Bennett et al., 2012). A follow up in 5 patients after 3 years additionally shows positive results (Testa et al., 2013).

A fourth trial (NCT01461213) is currently ongoing aiming to transduce retinal pigment epithelial and photoreceptor cells by subretinal delivery of rAAV2/2 vector solution in patients with choroideremia, caused by mutations in REP1. Again, **no SAE has been reported** and accumulating evidence suggests a **dose dependent therapeutic effect** in the treated vs. the untreated eye (Barnard et al., 2014; Huckfeldt and Bennett, 2014; Maclaren et al., 2014).

More detailed information is available from preclinical studies. The use of rAAV2/8 has been shown to be without safety concerns in the mouse to treat RP and choroideremia (Pang et al., 2011; Black et al., 2014), in the mouse and rabbit to treat X-linked retinoschisis (Park et al., 2009; Marangoni et al., 2014). Vandenberghe et al. investigated effects of subretinal delivery of rAAV2/8 expressing eGFP under the control of the ubiquitous CMV promoter in cynomolgus macaques (Vandenberghe

et al., 2011). Four different dosing regimens (10e8, 10e9, 10e10, and 10e11 vgp) were tested regarding local toxic effects (inflammation, degeneration), biodistribution of reporter gene, and systemic immune response. The following subheadings refer to this study, where not stated otherwise.

1.1.1 Immune response

Pre-clinical and clinical investigations have assessed the humoral and cellular immune responses to AAV administration to be safe and effective over a time period many years (see e. g. Willett and Bennett, 2013).

With respect to the **humoral immune response**, neutralizing antibodies (NABs) directed at the AAV capsid showed an upward trend with higher dosing in both intraocular fluid and serum. NABs are antibodies directed against a specific epitope (such as AAV capsid proteins), which aim to neutralize the biologic action of the antigen without triggering the cellular immune response. Interestingly, titres of NABs did not show an obvious correlation with total transgene expression (whole mount fluorescence), relative intensity and/or percentage of transduced cells within area exposed to virus particles. This is in line with the notion that the subretinal space is relatively protected from immunresponse and supported by earlier research (Streilein, 2003; Li et al., 2008). Li et al. demonstrated that pre-existing NABs against AAV do not have an impact on the transduction efficiency of subretinal AAV particles. This has independently been confirmed in other laboratories (Ali R., personal communication).

In Vandenberghe's study, **T-cell response** directed against eGFP was detected in one out of twenty eyes (1 out of 5 in highest dose group). The same animal and one other animal (with no discernible systemic cellular/humoral immune response) from the highest dose group showed signs of retinal inflammation and thinning in the eye treated with the highest vector dose. It is currently unknown whether this local effect is primarily due to the high virus load, contaminants in the research grade vector solution (not GMP grade), or due to toxic effects of high eGFP expression levels. While the lead author suspects residual components as the main culprit (Vandenberghe, personal communication), other studies have given evidence for negative effects of eGFP on photoreceptor viability (Liu et al., 1999).

Ferreira et al. (Ferreira et al., 2014a; Ferreira et al., 2014b) report about the immune response from a phase II clinical trial leading to the market approval of the first gene therapy product (Glybera). Both cellular and humoral immune responses were evident against AAV1 capsid proteins despite continuous systemic immunosuppression with Cyclosporine A (3mg/kg/D) and Mycophenolat-Mofetil (2g/d) over 12 weeks and a single intravenous injection of Methylprednisolon (1mg/kg) just prior intramuscular administration of therapeutic AAV1 vector solution. Interestingly, they could not detect any immune response against the transgene (lipoprotein lipase), only against the capsid proteins. But even the humoral and cellular immune response did not lead to cytotoxicity through mechanisms not fully understood. The authors speculate that this tolerance might be caused by known immunological mechanisms of ignorance, anergy or clonal deletion

1.1.2 Biodistribution

The biodistribution of AAV particles injected into the subretinal space is very limited. Several independent clinical trials using AAV for subretinal gene delivery have excluded AAV dissemination to peripheral blood (Hauswirth et al., 2008; Maguire et al., 2008; Jacobson et al., 2012) .Only in one case in one study, was AAV genome detected in a tear sample on day 1 after surgery, with none detected at later time points (Maguire et al., 2008).

Other clinical trials using essentially the same vector as proposed in this study (rAAV2/8 (Nathwani et al., 2011) administered ca. 1000 fold higher numbers of virus (2×10e12vgp/kg) via peripheral-vein infusion directly into the systemic circulation of hemophilia patients. Interestingly, vector genome was detectable in the plasma, saliva, semen and stools within 72 hours of vector infusion and up to but not after day 15 in all participants, with the exception of participant 1 whose semen remained clear of proviral DNA at all-time points assessed. Vector sequences were not detected in the urine of any of the participants at any time point after administration of vector in this study.

In the preclinical setting, Vandenberghe et al. (2011) used qPCR to detect reporter

gene (eGFP) biodistribution in non-human primates after subretinal delivery of AAV8 and showed dose dependent expression of eGFP mainly in retinal pigment epithelium (RPE) cells and photoreceptors (PRs). At a titre of 10e9 AAV8 transduced ca. 25% of PRs in the exposed area. At 10e10 already ca. 70±20% of PRs and RPE cells were positive for the reporter transgene. AAV8 showed ca. one log unit higher transduction efficiency when compared to AAV2. Given the ubiquitous activity of the CMV promoter, there was also extraocular eGFP expression found e.g. in the optic nerve (in 1/5 eyes at 10e10vgp; in 5/5 eyes at 10e11vgp) and in the optic chiasm (in 5/5 eyes at 10e11vgp). The authors described a correlation between the dosage (vgp) and extent of extraocular eGFP expression with animals in the high dose group having the most extensive extraocular involvement. However, the pattern of eGFP distribution could not fully be explained, as it did not match the pathway of retrograde transport in the visual pathway. E.g. the highest eGFP signal came from layers within the lateral geniculate nucleus (LGN) known to be dominated by the magnocellular system, which is associated with retinal ganglion cells (RGCs) from more peripheral retinal areas. These are also known to travel within the more peripheral parts of the optic nerve (ON), which might make them more accessible for primary transduction e.g. at the optic nerve head, where those axonal fibers are in direct contact with the subretinal space. On the other hand, AAV particles could have traversed the entire retina to reach the RGCs directly. However, this appears unlikely as no intermediate cells (e.g. bipolar cells) were eGFP positive even though the promoter would be active ubiquitously.

Taken together, it is not entirely sure, whether the extraocular transgene expression is really due to retrograde transfection at higher dose ranges, or whether subretinal delivery of AAV potentially exposes peripheral axonal fibers to transduction. This can be elegantly addressed by using a promoter with target cell specific activity in the therapeutic vector such as used in this study.

1.2 Efficiency

The vector of choice, rAAV2/8, has been studied in detail regarding its efficiency to transduce photoreceptor cells – the target cells for our trial. Vandenberghe et al.

(2011) injected rAAV2/8 expressing eGFP under the control of the CMV promoter and a woodchuck hepatitis virus post-transcriptional regulatory element into the subretinal space of cynomolgus macaques. The vector efficiently transduced RPE and photoreceptor cells across a dose range (10e8, 10e9, 10e10, and 10e11vgp).

2. Dosing

2.1 Overview

In the proposed clinical trial, patients will have one of three dosages injected into a preformed subretinal space with a maximal total volume of 1ml of balanced salt solution (BSS):

- Low dose: ≤ 1x10e10 vector genome particles (vgp)
- Intermediate dose: ≤ 5x10e10vgp
- High dose: ≤ 1x10e11vgp

2.2 Volume

The mouse eye has a vitreous volume of approximately 7µl. In non-human primates the vitreous volume ranges from circa 1.5 to 3ml, in humans from 4 to 8ml, depending on axial length. This indicates a three log unit difference from mouse to men. A 1µl subretinal injection in mice would therefore be broadly equivalent in scale to a volume of 1 ml in humans.
Based on our own studies, we know that an injection volume of 1-1.5µl will detach circa one third to one half of the retinal area in a mouse. In non-human primates, Vandenberghe et al. (2011) used 150µl to create a subretinal space covering ca. 20% of the retinal area in cynomolgus macaques. In humans, volumes of ca. 0.5-1ml would be expected to create an equivalent subretinal space. Volumes of 1ml in the subretinal space are quickly resorbed (within 24h) mainly through the trans-cellular pump mechanisms of the retinal pigment epithelium (RPE). However, the absolute volume might be considered less important than the absolute number of vector genome particles placed in the subretinal space.

2.3 Viral particle number

The relevant measure is the number of vector genome containing particles (vgp) and the number of target cells, which are brought in physical contact with the vector solution. Previous studies targeting the RPE have applied 1x10e10 to 1.5x10e11 rAAV2/2vgp into the subretinal space. The only published trial using the same vector design of the proposed study (rAAV2/8) used significant higher doses (1-2x10e14vgp) to target hepatocytes, and vgp were injected into systemic circulation rather than the confined subretinal space in the eye. Yet, no SARs were recorded in this trial.

Vandenberghe et al. (2011) showed local toxic effects (degeneration and inflammation) in non-human primates after subretinal injection of 1x10e11 vgp AAV2/8. However, it is currently not clear whether these adverse effects can primarily be attributed to virus load or are indeed secondary effects due to high eGFP expression.

Michalakis et al. (2010) showed successful gene therapy in the relevant mouse model (CNGA3-ko) by injecting 6-9x10e9 rAAV2/5 into the subretinal space detaching ca. 1/3 of the retina. This would be predicted to bring ca. 60,000 cone photoreceptors in direct contact with the vector solution (Jeon et al., 1998), resulting in a ratio of 100,000 – 150,000vgp/cone photoreceptor. As the proposed study foresees to detach the full macula, ca. 9% of all cone photoreceptors (200,000-300,000) will be in direct contact with the vector solution (Curcio et al., 1990). The equivalent dose would therefore be ca. 2-4.5x10e10vgp per injection.

At the same time, a significant number of vgp can be expected to transduce other, more prevalent cell types such as RPE cells and rod photoreceptors and therefore become unavailable for cone photoreceptor target cells (in contrast to transduction, however, transgene expression is limited due the use of a cone specific promoter sequence).

For the proposed gene therapy trial dosing groups of ≤1x10e10, ≤5x10e10 and ≤1x10e11vgp were chosen as the most promising range and toxic effects in non human primates will be tested using two doses: 1x10e11 and 1x10e12vgp. These concentrations are ten times and one hundred times higher, respectively, to the dose proposed in the first cohort of three patients (≤1x10e10) to be treated.

Safety and efficacy of a single subretinal injection of rAAV.hCNGA3 in patients with CNGA3-linked achromatopsia investigated in an exploratory, dose-escalation trial

Version 5.0

Status: Final

Protocol No: RDC-CNGA3-01
EudraCT No: 2014-001874-32
Reference No EC Tübingen: 096/2015 AMG1

Date: 16. February 2017

Table of Contents

I Amendment history ... 74
II Abbreviations ... 75
III Roles in the study ... 78
IV Synopsis ... 79
V Sponsor and Investigator Agreement ... **Error! Bookmark not defined.**
VI Synopsis in German / Deutsche Prüfplan- zusammenfassung ... 83
VII Flowchart of Visits and Procedures ... 85
1. Introduction ... 87
1.1.1. Clinical characteristics and genetics of achromatopsia ... 87
1.2. Rationale of the Trial ... 89
1.3. The rAAV.hCNGA3 Vector for the use in the human trial ... 91
1.3.1. Vector Production ... 92
1.3.2. Preclinical testing in rodents ... 93
1.3.3. Preclinical testing in non-human primates ... 93
2. Aim of the Trial ... 94
2.1. Primary Aim ... 94
2.2. Secondary Aims ... 95
3. Study Plan ... 95
3.1. Study Design ... 95
3.2. Study Duration ... 97
3.3. Participating Sites ... 97
3.4. Number of enrolled patients ... 97
4. Study Population ... 97
4.1. Characterization of Patient Population ... 97
4.2. Inclusion Criteria (Study Eye) ... 98
4.3. Exclusion Criteria ... 98
5. Patient Inclusion ... 99
5.1. Time plan for inclusion ... 99
5.2. Mode of assignment of patients to treatment ... 99
6. Intervention ... 100
6.1. Treatment ... 100
6.2. Concomitant medication ... 100
7. Endpoints for Safety and Efficacy of a single subretinal injection of rAAV.hCNGA3 genome ... 101
7.1. Efficacy ... 101
7.2. Safety ... 101
7.2.1. Ocular and systemic safety ... 101
7.2.2. Data Monitoring Committee ... 102
7.2.3. Definitions ... 102
7.3. Reporting Procedures for all Adverse Events ... 103
7.3.1. Reporting Procedures for Serious Adverse Events ... 104
7.4. Rules for discontinuation of the trial ... 104
7.5. Risk-Benefit Considerations ... 105

7.6. Risks for the Environment 108
8. Examinations 108
8.1. Screening Examinations 108
8.2. Trial Examinations 109
8.3. Follow-up Examinations 114
8.4. Non-ophthalmological and ophthalmological Procedures 116
8.4.1. Medical / surgical history 117
8.4.2. Ocular / surgical history 117
8.4.3. Body Mass Index 117
8.4.4. VIIs 117
8.4.5. Urine pregnancy test 117
8.4.6. Hematology, basic chemistry and urine analysis 117
8.4.7. CRP, Immunoglobulines 118
8.4.8. Immunopathology 118
8.4.9. PCR of rAAV8 genome 118
8.4.10. Best corrected visual acuity 118
8.4.11. Basic ophthalmological examination 118
8.4.12. Contrast sensitivity (Pelli Robson Charts) 118
8.4.13. Flicker Fusion Frequency 119
8.4.14. Colour constancy 119
8.4.15. Anomaloscopy 119
8.4.16. Cambridge Colour Test / Farnsworth D15 119
8.4.17. Infrared-Video-Pupillography 119
8.4.18. Microperimetry 120
8.4.19. Fundoscopy 120
8.4.20. Dark adaptation (DA) 120
8.4.21. Ganzfeld electroretinogram (ERG) 120
8.4.22. Spectral domain optical coherence tomography (SD-OCT) 120
8.4.23. Fundus autofluorescence (FAF) 121
8.4.24. Fundus photography (FP) 121
8.4.25. Angiography 121
8.4.26. Topical steroids and antibiotics 121
8.4.27. Systemic steroids 121
8.4.28. Subretinal injection of rAAV8.hCNGA3 vector 122
8.4.29. VFQ25 122
8.4.30. Psychiatric examination 122
8.4.31. Brief symptom inventory (BSI) 122
8.4.32. Psychological counselling 122
8.4.33. Study specific scale, A3-PRO 122
8.4.34. Adverse event (AE) recording 122
8.4.35. Concomitant medication 123
8.4.36. HIV Test 123
9. Documentation of Trial Data 123
10. Biometrical Planning and Analysis 123
10.1. Trial Design 123

10.2. Sample Size Issues 123
10.3. Definition for Study Groups 123
10.4. Statistical Analysis 124
10.5. Interim Analyses 125
11. Anticonception Rules and Pregnancy 125
13. Conditions for Amendments 126
14. Ethical and Regulatory Aspects 126
15. References 128
16. Appendices 135

I Amendment history

Number	Protocol Version	Date	Summary of changes
1	3.0	31.08.2015	Version 3.0 (page 13)<br>Study duration (Section 3.2, Page 28)<br>Risk-Benefit Considerations (Section 7.5, page 38/39)<br><br>Risks for the Environment (Section 7.6, page 40)
2	4.0	14.12.2015	Section VII (Flowchart) and 8.4.16 Farnsworth D15 was added<br>Section VII (Flowchart) HIV Test was added<br><br>Section 8.1: Screening examinations were adapted to the flowchart<br><br>Section IV, VII, 7.2.1: ELISPOT was deleted
3	5.0	16.02.2017	Section 10.5. An interim analysis was added and the reason explained.

II Abbreviations

µm	micrometer
A3-PRO	intervention-specific scale assessing patient reported outcome
AAV	adeno-associated viral
AAV-5	virus capsid
AAV5-S	virus capsid
AAV8	virus capsid serotype for expression of transgenes in photoreceptors
AAV8-hCNGA3	AAV8-pseudotyped hCNGA3 viral particles
ACHM	congenital achromatopsia
AE	adverse event
AIS	electronic patient file of the Centre for Ophthalmology
ANSM	Agence nationale de sécurité du médicament et des produits de santé
AO-SLO	applied optics scanning laser ophthalmoscopy
AR	adverse reaction
ARR3	promoter
BCA	bicinchoninic acid
BCVA	best corrected visual acuity
BGHpA	bovine growth hormone polyadenylation site
bp	base pair
BSI	Brief Symptom Inventory
c19q13.3	gene locus
cGMP	cyclic guanosine monophosphate
cGMP-PDE	guanosine monophosphate-gene
CNG	cyclic nucleotide gated
CNGA3	cyclic nucleotide gated channel alpha 3 gene
$CNGA3^{-/-}$	CNGA3 knockout
CRF	case report form
CRO	clinical research organization
CRP	c-reactive protein
CsCl	cesium chloride
D	day
de-sat.	de-saturated
DMC	data monitoring committee
DMEM	Dulbecco's Modified Eagle's Medium
E. coli	Escherichia coli
e. g.	for example
E1A	protein
EC	Ethics Committee
EFS	Etablissement Français du Sang
ELISA	enzyme-linked immunosorbent assay
EMA	European Medicines Agency

ERG	electroretinography
ESR	erythrocyte sedimentation rate
ETDRS	Early Treatment Diabetic Retinopathy Study
FAF	fundus autofluorescence
FDA	Food and Drug Administration
FBS	fetal bovine serum
FP	fundus fotography
ff-ERG	full field electroretinography
FST	dark adaptation test
G	gauge
GCP	good clinical practice
GMP	guanosine monophosphate
GMP	good manufacturing practice
GNAT2	gene
hArr3	human cone arrestin promoter
hCNGA3	gene involved in rod monochromacy
HEK293	human embryonic kidney cell line
HRD	inherited retinal dystrophies
ICA	infectious center assay
	indocyanine green
IgG	antibody
IgM	antibody
INL	inner nuclear layer
ip/ml	infectious particles/milliliter
IR	infrared
ITRs	inverted terminal repeats
ITT	intent to treat
kg	kilogram
LCA	Lebers congenital amaurosis
m	meter
M	month
ml	milliliter
MP	microperimetry
NCT00481546	clinical trial
NCT00516477	clinical trial
NCT00643747	clinical trial
NCT01461213	clinical trial
NHP	non-humane primate
OCT	optical coherence tomography
ONL	outer nuclear layer
OPN1MW opsin	promoter
OPN1SW opsin	promoter
OS	outer segments
PCR	polymerase chain reaction
PDE6C	gene

PDE6H	gene
pDP8	gene
pDP8-KanR	AAV8 *trans* plasmid
PEG	polyethylene glycol
PEI	Paul Ehrlich Institute (Regulatory Authority)
PI	principle investigator
PNA	peanut agglutinin
PP	polypropylene
PP	per protocol
PR charts	Pelli Robson charts
PRO	patient reported outcome
pSub-hArr3-hCNGA3-WPREm-KanR	AAV *cis* plasmid
qPCR	quantitative polymerase chain reaction
rAAV	recombinant adeno-associated viral
rAAV.hCNGA3	vector
REP1	gene
RPE65	gene
SAE	serious adverse event
SAR	serious adverse reaction
Sat.	saturated
sdOCT	spectral domaine optical coherence tomography
SDS-PAGE	sodium dodecylsulphate polyacrylamide gelelectrophoresis
SSSOI	Balanced Sterile Saline Solution for Intraocular Irrigation
SUSAR	suspected unexpected serious adverse reaction
TC	telephone conference
TCID50	tissue culture infection dose
TE	treated eye
TFF	tangential flow filtration
UE	untreated eye
UKT	Universitaetsklinikum Tuebingen
USP	U.S. Pharmacopeial Convention
VA	visual acuity
VFQ	visual function questionnaire
vg	vector genome
vgp	vector genome particles
vol/vol	volume percent concentration
vp/ml	virus particle/milliliter
WPRE	woodchuck hepatitis virus post-transcript. regulatory element
wt	wild type
y	year

III Roles in the study

Sponsor

Universitätsklinikum Tübingen
Geissweg 3
72076 Tübingen

Principal Investigator

Prof. Dr. Dr. med. M. Dominik Fischer
Centre for Ophthalmology, University Tuebingen
Elfriede-Aulhorn-Straße 7
D-72076 Tuebingen
Phone +49 7071 29 87894
Fax +49 7071/29 5021
Email: dominik.fischer@med.uni-tuebingen.de

Deputy Principal Investigator

Prof. Dr. med. Karl Ulrich Bartz-Schmidt
Elfriede-Aulhorn-Straße 7
D-72076 Tuebingen
Phone +49 7071 29 84001
Fax +49 7071 29 5215
Email: karl-ulrich.bartz-schmidt@med.uni-tuebingen.de

Responsible Ethics Committee

Ethics Committee of the Medical Faculty of the University of Tuebingen
Gartenstr. 47
D-72074 Tuebingen
Tel.: +49 70 71/ 29 77661
Fax: +49 70 71/ 29 5965
ethik.kommission@med.uni-tuebingen.de

CRO

STZ *eyetrial* at the Centre for Ophthalmology, University Tuebingen
Elfriede-Aulhorn-Straße 7
D-72076 Tübingen
Phone +49 7071/29 84898 (Prof. Wilhelm); +49 7071/29 84894 (Dr. Peters)
Fax +49 7071/29 5021
Email: barbara.wilhelm@stz-eyetrial.de; tobias.peters@stz-eyetrial.de

IV Synopsis

Title: Safety and efficacy of a single subretinal injection of rAAV.hCNGA3 in patients with CNGA3-linked achromatopsia investigated in an exploratory, dose-escalation trial

Phase: I/II

Indication: CNGA3-linked achromatopsia

Aim: To proof the safety and efficacy of rAAV.hCNGA3 in patients with achromatopsia

Study design: open, mono-center trial with fellow-eye comparison

Study Population:

Inclusion Criteria (Study Eye)

- clinical diagnosis of achromatopsia
- ≥ 18 years of age
- confirmed mutation in CNGA3
- BCVA ≥ 20/400
- a minimal outer nuclear layer thickness of 10µm at 3° eccentricity in the study eye (normal = 38±6µm)
- ability to understand and willingness to consent to study protocol
- no infection with Human Immundeficiency Virus (HIV)
- negative pregnancy test in women with childbearing potential (a woman who is two years post-menopausal or surgically sterile is not considered to be of childbearing potential)

Exclusion Criteria

- additional interfering eye conditions (e.g. uveitis, advanced cataract) in the study eye
- systemic conditions (e.g. coronary heart disease, autoimmune disorders) which may affect study participation or outcome measures
- current or recent participation in other study/or administration of biologic agent within the last three months
- recent (6 months) ocular surgery, intravitreal or subretinal implantation of a medical device
- known sensitivity to any compound used in the study
- contraindications to systemic immunosuppression
- subject/partner of childbearing potential unwilling to use adequate contraception for four months

- nursing or pregnant women
- any other cause that, in the investigator‘s opinion, renders potential subjects not suitable for the study
- mutations in another achromatopsia gene
- contraindications in view of the planned surgery (e.g. anaemia Hb<8g/dl, severe coagulopathy, severe blood pressure fluctuations)
- ocular opacity and mature cataract
- ocular infection with herpes simplex virus in medical history
- history of ocular malignancies
- disorders of the internal retina (e.g. retinal detachment in the patients history)
- glaucoma defined as damage of the optic nerve
- vascular retinal occlusion
- diabetic patients suffering from retinopathy and/or macula edema
- patients treated with oral corticoids within 14 days prior inclusion
- systemic illness or medically significant abnormal laboratory values in blood analysis including renal and hepatic functions at inclusion
- absence of vision on the other contralateratel eye

Patient Number: Nine patients will be assigned to three cohorts of three patients respectively.

Treatment:
Each cohort will receive a different, increasing dosage of viral vector genome particles. Three cohorts of patients are planned with low, medium and high dose administration levels up to 1x10e11 of rAAV8 incorporating the therapeutic expression cassette.
After standard three-port 23G pars plana vitrectomy, balanced salt solution will be used to induce a localized primary retinal detachment in a controlled fashion. Vector solution will be applied using disposable 41G extendible subretinal injection needles within a standard 23G body to fit the port system. Administration will be unilateral (worse eye) in each patient.

Primary Endpoint: Safety as the primary endpoint will be assessed by clinical examination of ocular inflammation (slit lamp, fundus biomicroscopy, angiography, perimetry or electrophysiology). Systemic safety will be assessed by vital signs, routine clinical chemistry testing (including CRP, ESR) and full/differential blood counts. Immunopathology essays will include specific enzyme-linked immunosorbent assays for humoral antibodies against rAAV8 capsid protein. Biodistribution will be monitored by polymerase chain reaction studies on rAAV8 genome in blood, urine, saliva and lachrymal fluid.

Statistical Methods: AEs and SAEs will be documented using line listings and tabulations (MetraCad code will be applied). Descriptive analysis will include line charts for individual patients. Standard errors and means will also be displayed using line charts. This will be done for the raw measurements and for the difference between treated and untreated control eye within each patient.
Descriptive parameters will be given for the full cohort (n=9) but not for the sub-cohorts (n=3). Additionally correlation analysis will be performed in a purely descriptive manner for the comparison of subjective and objective measurements. For each subject and measurement, the delta of the value for the interventional eye minus the mean of control eye values over time will be determined. In scatter plots for pairs of variables (measurements pooled over subjects) the association between these deltas will be displayed.

Time Schedule: Start of trial November 2015, end of recruitment November 2016, end of trial November 2017, duration of trial per patient: one year with four years of follow-up.

V Sponsor and Investigator Agreement

Sponsor of this clinical trial is the University Hospital Tübingen, Geissweg 3, 72076 Tübingen.

The sponsor is represented by the Principal Investigator or his deputy and confirms that this protocol entitled "Safety and efficacy of a single subretinal injection of rAAV.hCNGA3 in patients with CNGA3-linked achromatopsia investigated in an exploratory, dose-escalation trial" Version 5.0 (EudraCT 2014-001874-32) has been carefully read and fully understood, and there is agreement to comply with the conduct and terms of the study specified herein in compliance with Good Clinical Practice and all other relevant regulatory requirements.

Principal Investigator (PI) D. FISCHER

Date 16/02/2017

Signature

Deputy Principal Investigator BARTZ-SCHMIDT

Date 16.2.17

Signature

VI Synopsis in German / Deutsche Prüfplan-zusammenfassung

Titel: Sicherheit und Wirksamkeit einer einzelnen subretinalen Injektion von rAAV.hCNGA3 bei Patienten mit CNGA3-chromosomaler Achromatopsie, untersucht in einer explorativen Studie mit ansteigender Dosierung.

Phase: I/II

Indikation: CNGA3-chromosomale Achromatopsie

Ziel: Nachweis der Sicherheit und Wirksamkeit von rAAV.hCNGA3 in Patienten mit Achromatopsie

Studiendesign: Offene monozentrische Studie mit Vergleich zum Partnerauge

Studienpopulation:
Einschlusskriterien (Studienauge)

- Klinisch diagnostizierte Achromatopsie
- Alter ≥18 Jahre
- Bestätigte Mutation in CNGA3
- bestkorrigierte Sehschärfe ≥ 20/400
- minimale äußere nukleäre Schichtdicke von 10 µm bei 3° Exzentrizität im Studienauge (normal = 38±6µm)
- Fähigkeit das Studienprotokoll zu verstehen und in die Teilnahme einzuwilligen
- Keine Infektion mit dem Humanen Immundefizienz-Virus (HIV)
- Negativer Schwangerschaftstest bei gebärfähigen Frauen (Frauen 2 Jahre nach Beginn der Menopause oder operativ sterilisierte Frauen zählen als nicht gebärfähig).

Ausschlusskriterien

- Zusätzliche Augenkrankheiten (z.B. Uveitis, fortgeschrittener Katarakt) im Studienauge
- systemische Begleiterkrankungen (z.B. Koronare Herzkrankheiten, Autoimmun-erkrankungen), die für die Studienteilnahme als wesentlich erachtet werden
- Aktuelle oder kürzliche Teilnahme an einer anderen Klinischen Studie und/oder Einnahme biologischer Agenzien innerhalb der letzten 3 Monate
- Kürzliche (6 Monate) Augenoperation, intravitreale oder subretinale Implantation eines Medizinproduktes
- Bekannte Unverträglichkeit gegenüber einer in der Studie eingesetzten Substanz
- Kontraindikationen zur systemischen Immunsuppression

- Gebärfähige Patienten oder Partner, die nicht zur Empfängnisverhütung für vier Monate bereit sind
- Stillende oder schwangere Frauen
- Andere Gründe, die nach Meinung des Prüfers gegen die Eignung eines Probanden sprechen
- Mutation in einem anderen Achromatopsie-Gen
- Kontraindikationen bezogen auf die geplante Operation (z.B. Anämie, Hb<8g/dl, schwere Koagulopathie, starke Blutdruckschwankungen)
- Trübung der optischen Medien und fortgeschrittene Katarakt
- Okuläre Herpes Simplex Infektion in der Krankengeschichte
- Okuläre Tumoren in der Krankengeschichte
- Erkrankungen der inneren Netzhaut (z.B. Netzhautablösung in der Krankengeschichte)
- Glaukom mit Schädigung des Sehnerven
- Gefäßverschluss in der Netzhaut
- Diabetiker, die unter Retinopathie und/oder Makulaödem leiden
- Patienten, die innerhalb von 14 Tage vor Studieneinschluss mit oralen Kortikoiden behandelt wurden
- Systemische Erkrankung oder klinisch relevant abweichende Laborwerte einschließlich Nieren- und Leberwerten bei Studieneinschluss
- fehlende Sehfunktion des Partnerauges

Anzahl der Patienten: Neun Patienten werden 3 Kohorten mit je 3 Patienten zugeordnet.

Behandlung:

Jede Kohorte wird eine andere Dosierung des viralen Vektor-genom-Partikels erhalten. Drei Patientenkohorten sind geplant mit niedriger, mittlerer und hoher Dosierung bis zu 1x10e11vgp des rAAV8.
Nach Standard 3-Port 23G pars-plana-Vitrektomie wird Salzlösung eingesetzt, um eine kontrollierte, lokale, primäre Netzhautablösung zu induzieren. Die Vektorlösung wird mittels 41G erweiterbarer subretinaler Einwegs-Injektionsnadel mit einem Standard 23G Korpus, passend zum Port-System, eingebracht. Die Gabe wird bei jedem Patient unilateral vorgenommen (schlechteres Auge).

Primärer Endpunkt:

Die Sicherheit als primärer Endpunkt wird durch klinische Untersuchung der okulären Entzündung (Spaltlampe, Funduskopie, Angiographie, Perimetrie oder Elektrophysiologie) beurteilt. Systemische Sicherheit wird durch Vitalparameter, routinemäßige klinische Chemie (einschließlich CRP, BSG) und Voll-/Differentialblutbild beurteilt. Als immunpathologisches Nachweisverfahren dient das spezifische antikörpergekoppelte Nachweisverfahren ELISA (enzyme-linked immunosorbent assay) für humorale Antikörper gegen das rAAV8 Capsid-Protein, um die zelluläre Immunantwort gegen das rAAV8 Capsid-Protein zu überwachen. Die Biodistribution

wird durch Polymerase-Kettenreaktion (PCR)-Untersuchungen über das rAAV8 Genom in Blut und Urin überwacht werden.

Statistische Methoden:
Deskriptive Statistik ist geplant.

Zeitplan: Studienstart November 2015, Ende der Rekrutierung November 2016, Studienende November 2017, Dauer der Studie pro Patient: ein Jahr mit anschließend vier Jahren Nachbeobachtungsphase.

VII Flowchart of Visits and Procedures

See next page

Flowchart of Visits and Procedures CNGA3 Trial

	Visit (no)	1	2	3	4	5	6	7	8	9	10	Fup 1	Fup2	Fup3	Fup 4
	Date	Screening	D 0	D1	D2	D3	D14	D30	D90	D180	D365, Close out / ET	M24	M36	M48	M60
	Time window	day -30 to -2				± 1 day	± 3 days	± 5 days	± 7 days	± 7 days	± 14 days	± 1 month	± 1 month	± 1 month	± 1 month
	**Procedure**														
1.	Medical /surgical/ history	x													
2.	Ocular / surgical/ history	x													
3.	BMI	x									x				
4.	Vital signs	x	x	x	x	x	o	o	o	o	x	o	o	o	o
5.	Urine pregnancy test	x	x				x	x	x	x	x				
6.	Hematology/basic chemistry/urine analysis	x	o	o	o	x	x	o	o	x	x	x	x	x	x
7.	CRP, IgG, IgM	x			x	x	x	x	o	o	o	o	o	o	o
8.	Immunopathology[a]	x						x	x	o	o	o	o	o	o
9.	PCR of rAAV8 genome	x				x	x	o	o	o	o	o	o	o	o
10.	Best corrected visual acuity	x					x	x	x	x	x	x	x	x	x
11.	Basic ophthalmological exam (miosis)	x	x	x	x	x	x	x	x	x	x	x	x	x	x
12.	Contrast sensitivity (PR charts)	x					x	x	x	x	x	x	x	x	x
13.	Flicker fusion frequency	x						x	x	x	x				
14.	Colour constancy (chromatic adaptation)[e]	x						o	o	x	x	o	o	o	o
15.	Anomaloscopy[e]	x					x	x	x	x	x	o	o	o	o
16.	Cambridge Colour Test (CCT)[e] / Panel D15[f]	x					x	x	x	x	x	o	o	o	o
17.	IR-Video-Pupillography	x						x	x	x	x	o	o	o	o
18.	MP-1 Microperimetry (20°)	x						x	x	x	x	x	x	x	x
19.	Fundoscopy (mydriasis)	x	x	x	x	x	x	x	x	x	x	x	x	x	x
20.	Dark adaptation test	x						x	x	x	x	o	o	o	o
21.	ff-ERG (scotopic and photopic)	x						x		o	x	o	o	o	o
22.	sdOCT	x	o	o	o	o	x	x	o	o	x	x	x	x	x
23.	Fundus autofluorescence	x					x	x	o	o	x	x	x	x	x
24.	Fundus photography	x					x	x	x	o	x	x	x	x	x
25.	ICG (Indocyanin green) angiography	x					o	o	x	o	x	o	o	o	o
26.	Systemic steroids[b]	x (Day -1)	x	x	x	x									
27.	Topical steroids and antibiotics[c]	x (Day -1)	x	x	x	x	x	x							
28.	Subretinal injection of rAAV8 vector		x												
29.	VFQ25	x						x		x	x	o	o	o	o
30.	Psychiatric examination	x													
31.	Brief Symptom Inventory (BSI)	x					x	x	x	x	x				
32.	Psychological counselling[d]		x	x	x	x	x	x	x	x	x				
33.	study specific scale, A3-PRO						x	x	x	x	x	x	x	x	x
34.	Adverse event recording		x	x	x	x	x	x	x	x	x	x	x	x	x
35.	Concomitant medication	x	x	x	x	x	x	x	x	x	x	x	x	x	x
36.	HIV Test	x													

o: optional, if control deemed necessary by investigator

[a] including but not limited to: Anti-AAV8 capsid ELISA

[b] Systemic steroids will be given orally 1.0mg/kg for appr. three weeks starting at day -1 and then tapered off after day 19 as deemed appropriate by the investigator (e.g. 40/30/20/15/10mg).

[c] Moxifloxacin eye drops 0.5% 4x/d & 0.1% dexamethasone gel 4x/d for 21d starting at day -1

[d] at patient's need and discretion

[e] if the previous tests have shown improved results

[f] if CCT is not feasible

2. Introduction

1.1.Background

This is an investigator-initiated trial fully funded by the Tistou and Charlotte Kerstan Stiftung, Germany. The trial is part of the RD-Cure project, a joint project of the Universities Tübingen and Munich, Germany, addressing the development of the first ophthalmological gene therapy approaches in CNGA3-linked achromatopsia and – at a later stage – PDE6A-linked retinitis pigmentosa in Germany. The RD-Cure project including its clinical trials is under the continuous supervision of an international scientific Reviewer and Advisory Board (RAB).
Sponsor of the Trial presented here are the University Hospitals Tübingen, Germany.

1.1.1. Clinical characteristics and genetics of achromatopsia

Congenital achromatopsia (ACHM) or rod monochromatism is an autosomal recessively inherited congenital defect. Individuals can have a complete form of the disease, with a total lack of function of all three types of cones in the retina, or an incomplete form, in which one or more cone types may be partially functioning (Blackwell and Blackwell, 1961; Pokorny et al., 1982)
ACHM belongs to the group of inherited retinal dystrophies (HRD), a highly heterogeneous group of rare ocular diseases, with impaired light sensitivity and/or signal transmission within the neuroretina. Retinal dystrophies in general are a major cause of visual disability and legal blindness in the working population: The prevalence of general HRD in Danish children is 1.3 per 10,000 (Bertelsen et al., 2013), in Northern France the prevalence is 6.7 per 10,000 (Puech et al., 1961). Patients with HRD are more likely to be registered as blind at a much younger age, and have a longer duration of blindness and visual impairment, which significantly decreases their quality of life (Prokofyeva et al., 2009). HRD thus have considerable socio-economic impact (Flanagan et al., 2003).
Clinical Symptoms of ACHM are characterized by:

- Achromatopsia: A lack of the perception of color.
- Amblyopia: A neural condition causing reduced visual acuity without any morphological cause.
- Hemeralopia: Reduced visual capacity in bright light causing debilitating glare.
- Nystagmus: A pathological condition involving an uncontrolled oscillatory movement of the eyes.
- Photophobia: The avoidance of bright light by those suffering from hemeralopia.

ACHM is characterized by lack of cone photoreceptor function. Due to the complete unresponsiveness of cones (in contrast to common forms of color blindness, in which changes in expression of opsin genes merely affect spectral sensitivity but not the physiology of photoreceptors), ACHM is considered a severe ocular disease with serious consequences for vision as high-acuity central vision mediated by densely packed cones in the fovea is missing.

The disease was initially thought to be non-progressive because cone function appeared to be absent from birth, but recent morphological in vivo studies established ACHM to be a slowly progressing degenerative retinal disease, and foveolar cone photoreceptor loss can be observed in most adult ACHM patients (Thiadens et al., 2010; Aboshiha et al., 2014; Greenberg et al., 2014; Sundaram et al., 2014; Yang et al., 2014)

The key characteristics of ACHM are therefore very poor visual acuity ($\pm$ 0.1), severe photophobia at normal daylight conditions and complete color blindness that is accompanied by a pendular nystagmus. In addition there is a significant incidence of hyperopia in ACHM patients. Clinical testing by functional and morphological methods can reveal the complete lack of central cones.

Mutations in five genes are implicated in ACHM (see Table 1), which explains around 93% of cases (Roosing et al., 2014). The most common are mutations in one of the two genes encoding the alpha and beta subunit of the cone cyclic nucleotide-gated (CNG) channel subunits, CNGA3 (found in about 25-28% of cases: (Kohl et al., 1998; Kohl et al., 2000) and CNGB3 (50% of cases, (Sundin et al., 2000; Kohl et al., 2005). CNG channels are involved in transmitting information about vision and smell from sensory cells to the brain. Less common mutations are the GNAT2 gene (1-2% of cases (Aligianis et al., 2002; Kohl et al., 2002), which provides instructions for making the alpha subunit of transducin, that plays an essential role in transmitting visual signals from cones to the brain, and the PDE6C gene (1-2% of cases, (Chang et al., 2009; Thiadens et al., 2009; Thiadens et al., 2010; Sundaram et al., 2014), which encodes the alpha-prime subunit of cyclic guanosine monophosphate (cGMP) phosphodiesterase. Extremely rare (0.5% of cases) is a mutation in the PDE6H gene that participates in processes of transmission and amplification of the visual signal (Chang et al., 2009; Thiadens et al., 2009; Kohl et al., 2012). cGMP-PDEs are the effector molecules in G-protein-mediated phototransduction in vertebrate rods and cones

Table 1. Genetic Variants of Achromatopsia

Gene	Populations	Location	Incidence
CNGB3	Pingelapese and Irish	8q21	50%
CNGA3	Danish, Jews of Iraq, Iran and Morocco	2q11	25%
GNAT2	Europeans of Italy and Denmark	1q13	2%
PDE6C	Not determined	10q24	2%
PDE6H	Europeans of the Netherlands and Belgium	12q13	0.5%

Currently there is no effective and approved treatment available for ACHM.

1.1.2. Current experience with gene therapeutic treatment of hereditary degenerative retinal diseases

Treatments for other hereditary retinal degenerative diseases (Lebers congenital amaurosis, LCA; chorioideremia) are currently administered in clinical trials with

positive results. Three landmark clinical trials (NCT00516477, NCT00481546 and NCT00643747) have first shown safety and evidence of efficacy using recombinant adeno-associated viral (rAAV) vectored gene therapy in the eye (Bainbridge et al., 2008; Hauswirth et al., 2008; Maguire et al., 2008; Jacobson et al., 2012). In these three studies, nine patients with a different form of retinal degeneration caused by mutations in the gene encoding RPE65 were treated by subretinal injections of rAAV2/2 vector solutions without a single serious adverse event (SAE). A subset of these patients even demonstrated some degree of improved visual function in the untreated eyes. A phase 1 trial with 12 LCA patients additionally confirms the safety and efficacy of the method, also after re-administration to the second eye (Maguire et al., 2009; Simonelli et al., 2010; Bennett et al., 2012). A follow up in 5 patients after 3 years additionally shows positive results (Testa et al., 2013). However, although improved vision is reported, the underlying retinal degeneration may continue (Cideciyan et al., 2013).

A fourth trial (NCT01461213) is currently on-going aiming to transduce retinal pigment epithelial and photoreceptor cells by subretinal delivery of rAAV2/2 vector solution in patients with choroideremia, caused by mutations in REP1. No SAE has been reported so far and accumulating evidence suggests a dose dependent therapeutic effect in the treated vs. untreated eye (Barnard et al., 2014; Huckfeldt and Bennett, 2014; Maclaren et al., 2014). Further trials are planned.

The knowledge and experience gained in these trials mentioned above have been strongly encouraging for the planned novel intervention in ACHM.

1.2. Rationale of the Trial

The goal of the CNGA3 study is to develop, produce and investigate a recombinant adeno-associated viral (AAV) gene transfer vector for the curative therapy of CNGA3-linked inherited retinal dystrophies in patients, in order to counteract their progression and impairment of visual function leading to disability.

CNGA3 encodes the alpha subunit of the cone photoreceptor cyclic nucleotide-gated cation channel and mutations in this gene have been associated with complete and incomplete achromatopsia (Kohl et al., 1998; Wissinger et al., 2001; Burgueno-Montanes et al., 2014; Yang et al., 2014).

Recently, the curative potential of gene therapy in the CNGA3-deficient animal model has been demonstrated (Michalakis et al., 2010). The primary therapeutic effect was confirmed on the level of retinal morphology and histology as well as on the functional level. Regarding function a variety of methods were applied, including ERG and ganglion cell recordings, and tests of vision-guided behavior. The treatment in the animal model was exceptionally robust regarding the choice of specific promoters (OPN1SW opsin, OPN1MW opsin, ARR3) and virus capsides (AAV-5, AAV-8) and worked in all combinations. It was also robust despite the technically challenging subretinal injection procedure in the small mouse eye; 9 out of 10 mice showed substantial functional rescue. Finally, it was found that because trafficking to cone outer segments is limited to heterotetrameric CNG channels with correct stoichiometry, the genetically unaffected amount of endogenous CNGB3 subunits

determines the amount of channels in the active compartment of the cell, the outer segment, ensuring the correct dosage.

The principal proof-of-concept for restoration of cone-mediated vision by AAV5-mediated retinal gene replacement therapy in CNGA3$^{-/-}$ mice (Figure 1) has been described (Michalakis et al., 2010) and is illustrated in the following.

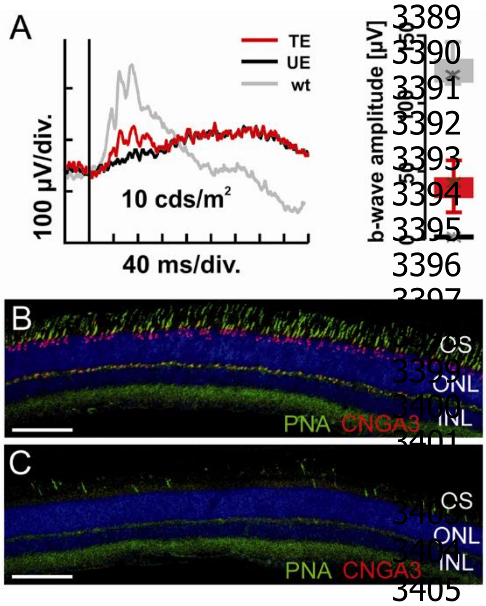

Figure 1. *Restoration of cone-mediated ERG and delay of cone degeneration in treated CNGA3$^{-/-}$ cones. (A) Single flash photopic ERG shows substantial restoration of cone system function (traces left, corresponding box plot bottom right). Treated eye (TE), untreated eye (UE), and wildtype eye (wt). (B and C) Retinal slices of age-matched treated (B) and untreated (C) CNGA3$^{-/-}$ mice, stained with the cone marker peanut agglutinin (PNA, green) and anti-CNGA3 (red) reveals the preservation of a high number of cones after treatment (B). Scale bars mark 100 µm. Nuclei are stained with Hoechst dye (blue). Abbrevations: INL, inner nuclear layer, ONL, outer nuclear layer, OS, (photoreceptor) outer segments.*

Different AAV serotypes with distinct preferential tropism for certain retinal cell types are available, and together with cell-type specific promoters provide exceptionally versatile and effective tools for gene therapy approaches targeting photoreceptors (see e.g. (Liu et al., 2011; Asokan et al., 2012; McClements and MacLaren, 2013)). In recent years AAV5 and especially AAV8 have evolved as the preferred serotypes for photoreceptor-specific gene expression in several animal models (Allocca et al., 2007; Lebherz et al., 2008; Tan et al., 2009; Mussolino et al., 2011; Vandenberghe et al., 2011; Smith et al., 2012). In subsequent studies Michalakis et al. confirmed a high efficacy for AAV8-mediated retinal gene replacement therapy in CNGA3$^{-/-}$ mice (Michelfelder et al., 2011).

In the past our research consortium performed a study in the Cnga3 knockout mouse model of achromatopsia comparing the efficacy of serotype 5 (rAAV5.mCnga3) and serotype 8 (rAAV8.mCnga3) AAV vectors (Michalakis et al., 2011). In total more than 50 Cnga3 knockout mice were injected with either rAAV5.mCnga3 or rAAV8.mCnga3 and a success rate (e.g. percentage of treated mice with positive biological activity assay (BAA) and positive TEA) of 67.6% and 63.0%, respectively was found. In all mice with positive BAA, TEA was also positive. There was no significant difference in the success rate between AAV5 and AAV8 serotyped vectors. However, the onset time for a positive BAA and TEA signal was faster with AAV8 vectors (4 weeks post injection for AAV8 versus 8 weeks post injection for AAV5). This finding and the data of other groups (Surace and Auricchio, 2008; Vandenberghe et al., 2011) prompted to continue with serotype 8.

In addition, it was found that AAV8-based vectors resulted in a faster and slightly more robust restoration of visual function compared to AAV5 (Figure 2). This further argues for AAV8 as the most effective serotype for expression of transgenes in photoreceptors. The ocular delivery of AAV8 is novel in this trial, but the same vector type has been applied systemically. A study that aimed at the correction of Hemophilia B tested the systemic delivery of very high AAV8 titers and no serious adverse effects have been reported (Nathwani et al., 2011)

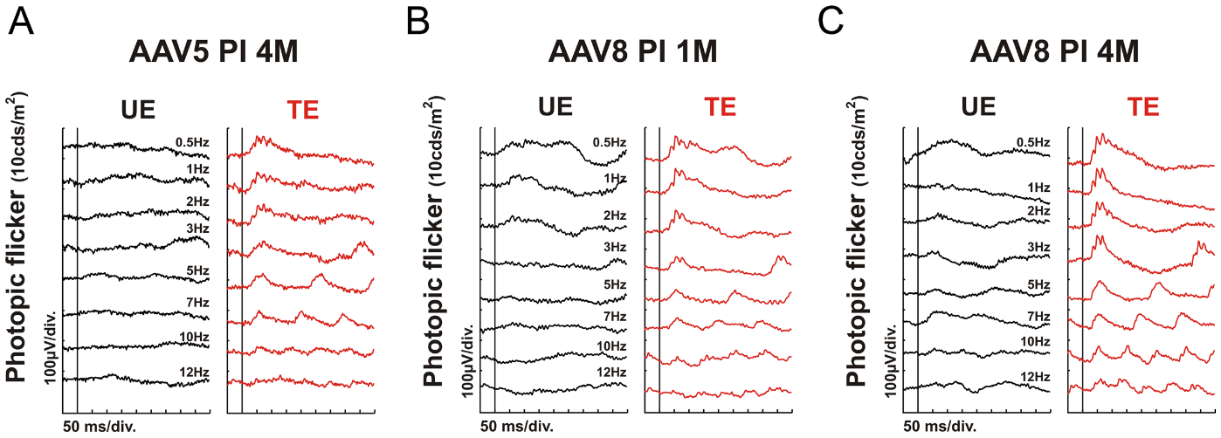

D

	4 weeks PI	6 weeks PI	8 weeks PI	3M PI	4M PI
AAV5-S opsin-CNGA3	X	X	✓	✓	✓
AAV8-S opsin-CNGA3	✓	✓	✓	✓	✓

Figure 2. Effect of AAV serotype on therapy onset. Representative photopic flicker series to compare the treatment efficacy of AAV5- and AAV8- mediated CNGA3 gene therapy in CNGA3$^{-/-}$ mice. Restoration of cone-specific light responses (A) by AAV5-S opsin-CNGA3 at 4 months (M) post injection (PI), (B) by AAV8-S opsin-CNGA3 at PI 1M and (C) by AAV8-S opsin-CNGA3 at PI 4M. UE, untreated eye. TE, treated eye. (D) Summary on the therapy onset data for AAV5 and AAV8 (Michalakis et al., 2011).

Based on these results the RD-Cure consortium decided for the AAV8 serotype for the CNGA3-linked achromatopsia intervention.

1.3. The rAAV.hCNGA3 Vector for the use in the human trial

The AAV gene therapy vector (AAV *cis* plasmid: pSub-hArr3-hCNGA3-WPREm-KanR) is based on the pSub201 *cis* plasmid backbone (Samulski et al., 1987) containing a kanamycin resistance gene. In this plasmid, the entire expression cassette is incorporated between two wild-type AAV2 inverted terminal repeats (ITRs). The expression cassette contains the 405 bp cone photoreceptor-specific human cone arrestin (hArr3) promoter (Li et al., 2002) and the full-length (2085 bp) human CNGA3 cDNA (Wissinger et al., 1997). The vector further contains a 543 bp woodchuck hepatitis virus post-transcriptional regulatory element (WPRE) with mutated WXF-open reading frame (Zanta-Boussif et al., 2009) and a 207 bp bovine growth hormone polyadenylation site (BGHpA). The AAV8 *trans* plasmid (pDP8-KanR)

was obtained from the pDP8 plasmid described in Penaud-Budloo et al. (Penaud-Budloo et al., 2008) by exchange of the ampicillin by a kanamycin resistance gene.

1.3.1. Vector Production

The GMP grade AAV *cis* and *trans* plasmid DNA was manufactured by Aldevron (Fargo, ND, United States of America). The production was carried out using an *E.coli* master cell bank procedure according to USP and EMA guidelines. These plasmids were used for the GMP production of AAV8-pseudotyped hCNGA3 viral particles (AAV8-hCNGA3) at Atlantic BioGMP (Nantes, France). Atlantic BioGMP is a fully GMP compliant pharmaceutical site for the production of AAVs authorized by ANSM and operated by the French National Blood Institute (Etablissement Français du Sang – EFS).
The AAV8-hCNGA3 production process involves calcium phosphate transfection of cis (pSub-hArr3-hCNGA3-WPREm-KanR) and trans (pDP8-KanR) plasmids in HEK293 cells. Cells were cultured in Dulbecco's Modified Eagle's Medium (DMEM) with 10 % fetal bovine serum (FBS; conforming to the "TSE-Note for Guidance EMEA 410/01 rev 03" of the European Directorate for the Quality of Medicines and to the monograph „Bovine Serum, European Pharmacopoeia 01/2008:2262, 7th Ed. Ph. Eur."). The medium did not contain any antibiotics or phenol red. AAV particles were harvested from cells and supernatant and purified by two consecutive CsCl-gradient centrifugation steps followed by tangential flow filtration (TFF) for buffer exchange and concentration, and finally sterile filtration (0.2µm) and filling. The final product is in Balanced Sterile Saline Solution for Intraocular Irrigation (SSSOI) (Industria Farceutica Galenica Senese, Ref: DD0412004, Supplier: Beaver-Visitec Intl Ref: 581732) with Kolliphor P188 Micro (= GMP grade PluronicF-68; BASF Ref: 50259816) (0.001% vol/vol). The product was filled into 1.2ml conical threaded polypropylene (PP) cryovials with threaded PP cap and a silicone washer (all USP Class VI, Corning Life Science Ref: 430658) at 0.13ml/vial and 5x10e12vg/ml (target genomic titer), finally stored at -70 °C ± 10°C. The production process and the final product conform to the "General chapter 5.14. of the 7th Ed. of the European Pharmacopoeia on gene transfer medicinal products for human use". Vector genome (vg) titer assayed by quantitative PCR (qPCR), infectious titer (infectious center assay, ICA) have been determined after each purification step. The harvest cells and supernatant were additionally tested for sterility (EP 2.6.1), mycoplasma (EP 2.6.7) and adventitious agents (EP 2.6.16). For the formulated final product, we will also determine the infectious titer using TCID50 (ip/ml) and vector particles using ELISA test (vp/ml). Finally, we will test for transgene expression (immunohistochemistry after treatment of CNGA3 KO mice), biological activity (electroretinography after treatment of CNGA3 KO mice), genomic identity (sequencing), protein purity (silver staining, Coomassie blue staining on SDS-PAGE), residual cell (qPCR for albumin and E1A) and plasmid (qPCR Kanamycin resistance gene) DNA, proteins (BCA, ELISAs), residual PEG, residual CsCl, residual benzonase (ELISA), endotoxins (EP2.6.14), pH,

osmolarity (EP 2.2.35), aggregates and appearance. The final product will also be tested for replication competent AAVs.

1.3.2. Preclinical testing in rodents

ERG amplitude was used as a biomarker for cone function and measure for vector efficacy. The efficacy of the murine vector pAAV2.1-mBP-CNGA3 was proven in CNGA3-/- knock-out mouse model (Michalakis et al., 2012).
The GMP-produced human vector to be used in our planned human trial was applied in the same knock-out mouse model. The *in vivo* biological activity – although less pronounced in comparison to the murine vector – could be demonstrated 8 weeks after treatment (short-term effect) with the engineering lot used ACHM2-ENG05 which was also used for the toxicology and biodistribution in NHPs. Regarding long-term efficacy, there is clear evidence that efficacy of the human vector is stable until at least 12 months post treatment in the mouse model with the process developmental lot ACHM2-DEV01.

1.3.3. Preclinical testing in non-human primates

Short term (n=12, 28 day) toxicological and long term (n=22, 13 week) toxicology and biodistribution studies were performed in male and female cynomolgus monkeys. The surgical subretinal application procedure proved to be feasible and safe. No ophthalmological adverse events occurred and no serological or clinical adverse events were observed. Animal activity and behavior, ECG and weight were normal up to 13 weeks after subretinal vector administration.
Shedding from the NHP BD studies analyzed in lachrymal and nasal swaps and urine showed detectable DNA in single animals up today 5 (1/6 animals, lachrymal swap), day 7 (nasal swap, 1/6 animals) and day 2 (urine, 1/6 animals) in the low dose subretinal group. Vector DNA could also be identified in single animals up to day 7 (lachrymal swaps, 2/6 animals; nasal swap, 3/6 animals) and day 5 (urine, 2/6 animals) in the high dose subretinal cohort, up to day 7 (lachrymal swap, 1/4 animals; urine, 1/4 animals; nasal swap, 2/4 animals) in the via falsa (intravitreal) high dose cohort. For the majority of animals, at these time-points the number of copies per µg was below or close to the level of significance defined by the FDA (i.e. 100 copies/µg).
None of the animals (which received subretinal injections (either low n=6 or high dose n=6) showed detectable DNA levels in the blood 72h post-injection. In the high dose via falsa group, vector DNA could be identified in the blood of all 4 animals up to four weeks post-injection.
The surgery and the perioperative care in NHPs deviates considerably from the situation in the clinical trial. The instruments available for such a surgery are optimized for human use. Due to the anatomical differences of the eye (e.g. size, location in orbit), some of the safety measures routinely performed in humans were not applicable in the NHPs. For example, displacement of conjunctiva over sclerotomies and introduction of trocars at an oblique angle were not possible. Likewise, NHPs could not be instructed to avoid any manipulation of the eye after

surgery to allow rapid healing of the self-sealing 23G sclerotomies. The investigators performing the NHP surgeries judged that stitching the sclerotomies would have introduced more irritation and cause for manipulation, which would have additionally facilitated potential leakage of fluid from the vitreous cavity into the tear film, lachrymal and nasal system. This is in stark contrast to the planned surgery in human patients, which is performed to minimize leakage and patients are instructed not to manipulate the eye after surgery to facilitate rapid closure of the self-sealing sclerotomies.

At the same time, the low dose in the NHP sample corresponds to the maximum dose planned to be applied in the clinical trial. In particular, regarding the shedding the limited replication-competence needs to be considered.

rAAV.hCNGA3 is unable to replicate independently, even in the presence of a helper virus, since it lacks the rep and cap genes required for rescue/packaging. Homologous recombination between rAAV.hCNGA3 and a wild type AAV could occur if both were present in the same cell. However, such recombination could only result in the exchange of the hCNGA3 expression cassette with the rep and cap genes of the wild type virus. It is not possible for the AAV genome to contain both rep/cap genes and the transgene, as this is beyond the packaging limit of the virion. Therefore the only mechanism by which the transgene could be mobilised is through a triple infection of the same cell by rAAV.hCNGA3 (containing the transgene), wild type AAV (providing the rep and cap functions) and a helper virus. This scenario is expected to be an extremely unlikely event, especially since the vector target cells (cone photoreceptors) are not the natural target cells of helper viruses and are shielded from viral infection by the blood-retina barrier. If it did occur, it would only result in the production of more wild type AAV and more rAAV.hCNGA3 vector particles (which would still lack rep and cap genes and consequently could not be self-sustaining).

Nevertheless, safety countermeasures will be taken for the clinical phase I trial with regard to the results mentioned above. Until day 7 post-injection, any materials and tissues coming into contact with the patient’s eyes, lachrymal fluid and nasal secretions will be subject to disinfection or autoclaving respectively. Study patients will be instructed to avoid intimate physical contact to other subjects until day 7 post-injection.

2. Aim of the Trial

The aim of this phase I trial is to proof the safety and efficacy of rAAV.hCNGA3 at three dosages in patients with achromatopsia.

2.1. Primary Aim

The primary aim of the trial is the investigation of the safety of rAAV.hCNGA3 after subretinal injection in patients with CNGA3-linked achromatopsia. Both the ocular and the systemic safety of the intervention will be investigated. Details regarding methods and parameters are described in chapters 7 and 8 of this trial protocol.

2.2. Secondary Aims

The investigation of treatment effects as reflected by patient reported outcomes and the efficacy of the intervention on visual function, as well as the evaluation of retinal imaging (safety) are secondary aims of the trial.

3. Study Plan

3.1. Study Design

This is an open mono-center trial with fellow-eye comparison. Three patients will be assigned to three consecutive, increasing dosage groups (see figure 3):

4) low dose: ≤ 1x10e10 vgp (n=3)

Between each injection (between patient 1 and 2 and patient 2 and 3) a safety interval of 4 weeks will be kept.
After collection of data on study visit Day 30 of all three patients of the dose cohort and before the scheduled start of the next injections of the consecutive dosage group the DMC will meet and evaluate safety data. If no safety concerns are reported the next dosage group of patients will be treated as scheduled. DMC advice may also result in a dose reduction for the next dosage cohort.

5) intermediate dose: ≤ 5x10e10 vgp (n=3)

Between each injection (between patient 4 and 5 and patient 5 and 6) a safety interval of 4 weeks will be kept.
After collection of data on study visit Day 30 and before the scheduled start of the next injections of the consecutive dosage group the DMC will meet and evaluate safety data of groups 1 and 2. If no safety concerns are reported the next dosage group of patients will be treated as scheduled. DMC advice may also result in a dose reduction for the next dosage cohort. Within the high dose cohort, the interval between injections / individual patients will be four weeks.

6) high dose: ≤ 1x10e11 vgp (n=3)

Between each injection (between patient 7 and 8 and patient 8 and 9) a safety interval of 4 weeks will be kept.
The following figure (Fig. 3) explains the schedule and timelines of dose groups and the points of decisions of the data monitoring committee.

Dosage/Dose Regimen for rAAV.hCNGA3 Phase I Trial

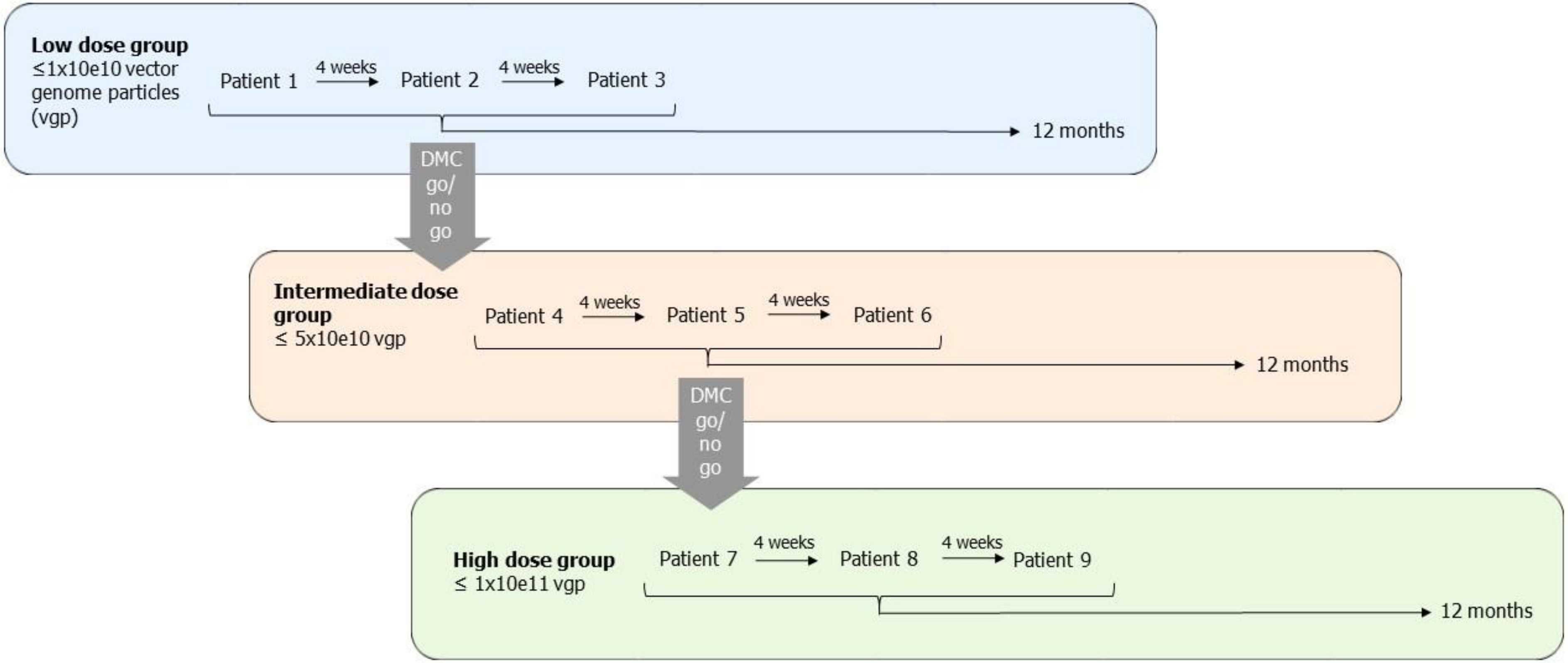

Figure 3: Schedule of dosing groups and intervals

3.2. Study Duration

- First patient first visit: November 2015
- Last patient first visit: November 2016
- Last patient last visit: November 2017
- Duration of trial participation per patient: 1 year plus 4-year follow-up period

After five years a clinical routine visit once per year and/or questionnaire will be performed (see also section 7.5) for long-term follow-up.

3.3. Participating Sites

The trial will be performed at the Centre for Ophthalmology, University of Tübingen. The site has a database of clinically and genetically characterized patients. In addition specialized retinal surgeons of the institution are familiar with subretinal interventions and have been trained in gene-therapeutic injections in an ongoing collaboration of the Centre for Ophthalmology with Oxford Eye Hospital, UK.

3.4. Number of enrolled patients

Nine patients will be enrolled in the trial. Drop-outs will only be replaced in case of non-ocular, non-treatment related reasons for the drop-out up to a number of two patients.

4. Study Population

4.1. Characterization of Patient Population

For this trial, adult patients of both genders with CNGA3-linked achromatopsia will be eligible (natural course and clinical symptoms see section 1.1.1.) with a BCVA ≥ 20/400 and discernable residual outer nuclear layer thickness in OCT scans of posterior pole. The precondition is the confirmed presence of mutation in the CNGA3 gene.
A) Patients will be pre-identified via the patient database of the Centre for Ophthalmology, University of Tübingen, they will be informed about the trial and invited for trial screening.
B) In addition, new patients to the clinic will be identified, informed about the trial and invited for trial screening. C) Patients from other hospitals, asking for trial participation will also be invited for screening.
Principally an intervention as early as possible in the course of the disease would be preferable, especially as the disease was initially thought to be non-progressive

because cone function was thought to be absent from birth. Yet, recent morphological in vivo studies established ACHM to be a slowly progressing degenerative retinal disease, and foveolar cone photoreceptor loss can be observed in most adult ACHM patients (Thiadens et al., 2010; Aboshiha et al., 2014; Greenberg et al., 2014; Sundaram et al., 2014; Yang et al., 2014). Therefore, it is deemed ethical to treat adult patients in this first interventional trial with the vector.

4.2. Inclusion Criteria (Study Eye)

- clinical diagnosis of achromatopsia
- ≥ 18 years of age
- confirmed mutation in CNGA3
- BCVA ≥ 20/400
- a minimal outer nuclear layer thickness of 10µm at 3° eccentricity in the study eye (normal = 38±6µm)
- ability to understand and willingness to consent to study protocol
- no infection with Human Immundeficiency Virus (HIV)
- negative pregnancy test in women with childbearing potential (a woman who is two years post-menopausal or surgically sterile is not considered to be of childbearing potential)

4.3. Exclusion Criteria

- additional interfering eye conditions (e.g. uveitis, advanced cataract) in the study eye
- systemic conditions (e.g. coronary heart disease, autoimmune disorders) which may affect study participation or outcome measures
- current or recent participation in other study/or administration of biologic agent within the last three months
- recent (6 months) ocular surgery, intravitreal or subretinal implantation of a medical device
- known sensitivity to any compound used in the study
- contraindications to systemic immunosuppression
- subject/partner of childbearing potential unwilling to use adequate contraception for four months
- nursing or pregnant women
- any other cause that, in the investigator‘s opinion, renders potential subjects not suitable for the study
- mutations in another achromatopsia gene

- contraindications in view of the planned surgery (e.g. anaemia Hb<8g/dl, severe coagulopathy, severe blood pressure fluctuations)
- ocular opacity and mature cataract
- ocular infection with herpes simplex virus in medical history
- history of ocular malignancies
- disorders of the internal retina (e.g. retinal detachment in the patients history)
- glaucoma defined as damage of the optic nerve
- vascular retinal occlusion
- diabetic patients suffering from retinopathy and/or macula edema
- patients treated with oral corticoids within 14 days prior inclusion
- systemic illness or medically significant abnormal laboratory values in blood analysis including renal and hepatic functions at inclusion
- absence of vision on the other contralateratel eye

5. Patient Inclusion

5.1. Time plan for inclusion

Patients will be screened for their eligibility after informed consent. Screening examinations may be distributed over three working days and should be performed in a time window of 30 to 2 days before treatment. There are no restrictions or recommendations with regard to the time after first diagnosis or the stage of the disease beyond the inclusion and exclusion criteria.

5.2. Mode of assignment of patients to treatment

Suitable patients in the patient data base of the Centre for Ophthalmology have already been identified and invited to an exploratory non-invasive trial, which has been running since December 2012 ("Detaillierte Genotyp-Phänotyp Analyse bei Patienten mit erblichen, genetisch gesicherten Netzhauterkrankungen" "Detailed genotype-phenotype analysis of patients with inherited genetically ensured retinal-diseases"). The patients out of this sample who qualify and are interested in participation in the rAAV.hCNGA3 trial will be invited for informed consent and screening visit.

6. Intervention

6.1. Treatment

See Appendix 1 for the rationale for vector and dosing for more details of the procedure. For reconstitution see detailed description in the Investigator Brochure.

1st: Standard three-port 23G pars plana vitrectomy (removal of vitreous humor and posterior hyaloid membrane) with balanced salt solution used to replace the vitreous humor and stabilize the eye during the procedure.

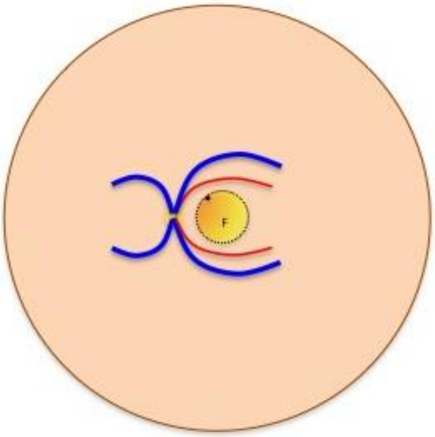

Figure 4: Scheme of injection

2nd: Balanced salt solution will be used to induce a shallow, localized primary retinal detachment involving the fovea (marked 'F' in Fig. 4) in an area of ca. 2mm diameter. The retinotomy (black dot) will be placed just central of the superior temporal branch of the central retinal artery (see Figure 4). This has given the most reproducible results in the non-human primate study with regard to localization of bleb formation.

3rd: Once the subretinal space has been pre-formed, 100 µl vector solution will be applied using a disposable 41G subretinal injection needle within a standard 23G body to fit the port system.

4th: An indentation search is performed gently to avoid vortexing of fluid inside the eye, which might induce a Venturi suction effect over the self-sealing retinotomy. Laser is applied to any peripheral breaks after relief of vitreous traction. Gas will be avoided where possible as it may induce a macular fold or displace subretinal vector to non-therapeutic regions. All vitrectomy ports will be sutured watertight at the end of surgery (e.g. using 8-0 vicryl sutures).

6.2. Concomitant medication

There are no forbidden medications during trial participation beyond the avoidance of anticoagulants around the surgical intervention with time lags depending on the half-life of the relevant drugs. Any concomitant medication will be documented during the trial.

7. Endpoints for Safety and Efficacy of a single subretinal injection of rAAV.hCNGA3 genome

7.1. Efficacy

Efficacy data (improvement in visual function) and patient reported outcomes will be investigated exploratively as well as retinal imaging. They are all secondary endpoints in this trial.

15. BCVA assessed using the ETDRS visual acuity protocol
16. Contrast sensitivity
17. Anomaloscopy
18. Cambridge Colour test
19. Flicker Fusion Test
20. Colour Constancy
21. MP-1 Microperimetry (20°)
22. Dark adaptation test (FST)
23. IR-Video-Pupillography
24. OCT, AO-SLO, IR, FAF and angiography recordings
25. FST (red/blue) and Ganzfeld-ERG
26. VFQ25
27. Brief Symptom Inventory (BSI)
28. A3-PRO (intervention-specific scale assessing patient reported outcome)

7.2. Safety

7.2.1. Ocular and systemic safety

Safety as primary endpoint will be assessed by clinical examination of

Ocular safety

3. loss of ≥ 15 letters visual acuity at 1m
4. severe, vector-induced, intraocular inflammation unresponsive to treatment

Systemic safety

5. vital signs
6. routine clinical chemistry testing (including CRP, IgG, IgM and full/differential blood counts
7. Immunopathology essays will include specific enzyme-linked immunosorbent assays for humoral antibodies against rAAV8 capsid protein
8. Biodistribution will be monitored by polymerase chain reaction studies on rAAV2/8 genome in blood, urine, saliva and lachrymal fluid.

7.2.2. Data Monitoring Committee

A Data Monitoring Committee (DMC) will monitor patients' safety at regular meetings. At crucial steps of the trial (see figure 3) such as after day 30, when safety data of previous patient cohort is available, the committee will provide a go/no go decision for the treatment of the next dose cohort. The DMC consists of Prof. Eberhart Zrenner, Tübingen, Prof. Robert MacLaren, Oxford, and Prof. Christian Hamel, Montpellier. The members of the DMC are independent of the clinical trial.

7.2.3. Definitions

Adverse Event (AE)
An AE or adverse experience is any untoward medical occurrence in a patient or clinical investigation participant administered a medicinal product, which does not necessarily have to have a causal relationship with the study treatment.
An AE can therefore be any unfavourable and unintended sign (including an abnormal laboratory finding), symptom or disease temporarily associated with the use of the study medication, whether or not considered related to the study medication.

Adverse Reaction (AR)
An AR or adverse reaction is an untoward and unintended reaction to a medicinal product related to any dose. The phrase "reaction to a medicinal product" means that a causal relationship between a study medication and an AE is at least a reasonable possibility, i.e., the relationship cannot be ruled out. All cases judged by either the reporting medically qualified professional or the sponsor as having a reasonable suspected causal relationship to the study medication qualify as adverse reactions.

Serious Adverse Event (SAE)
A serious adverse event is any untoward medical occurrence that at any dose:

- Results in death,
- Is life-threatening,
- Requires inpatient hospitalization or prolongation of existing hospitalization,
- Results in persistent or significant disability/incapacity, or
- Is a congenital anomaly/birth defect.

It is important to consider that the term "life-threatening" in the definition of "serious" refers to an event in which the participant was at risk of death at the time of the event; it does not refer to an event, which hypothetically might have caused death if it were more severe.

Serious Adverse Reaction (SAR)
A SAR, a serious adverse reaction, is an adverse event (expected or unexpected) that is both serious and, in the opinion of the reporting investigator, believed with reasonable probability to be due to one of the study treatments, based on the information provided.

Expected Serious Adverse Events/Reactions

Expected serious adverse events/reactions (ESAE/ESAR) are most likely to be related to (1) complications of retinal surgery, (2) immune reactions to the vector capsid or (3) immune reactions and/or toxic effects on retinal function caused by expression of human CNGA3 protein.

(1) Complications of retinal surgery

There are known complications of three port pars plana vitrectomy and subretinal surgery. These include retinal detachment, traumatic cataract, suprachoroidal and/or subretinal hemorrhage and endophthalmitis.

(2) Immune reactions to vector capsid

These are not expected, given the safety history with much larger doses of this vector in non-ophthalmological clinical trials (Nathwani et al., 2011), but cannot be ruled out completely. Therefore, presence of immune reactions will be investigated during the follow-up clinical examinations. Severe immune reactions triggered by the vector are likely to be evident within the first day of administration and might include non-infectious severe vitritis, chorioiditis or endophthalmitis. There may be additional systemic reactions such as headache and fever. Hence the patient will be monitored overnight and undergo a check up on the day after surgery.

(3) Immune reactions and/or toxic effects on retinal function caused by AAV8-CNGA3

Due to the time required for transgene expression following subretinal delivery of AAV8, it is unlikely that any toxic effects of the transgene product would be evident prior to at least two weeks after surgery at the earliest. These might include similar immune reactions as described in (2) above. Also any toxic effects of AAV8-CNGA3 on visual function might be manifest by a worsening of visual acuity after the initial period of post-operative recovery. Potential toxic effects on retinal function are likely to be manifest as a significant drop in visual acuity (defined by 15 letters or more).

Suspected Unexpected Serious Adverse Reaction (SUSAR)

A suspected unexpected serious adverse reaction is a reaction, the nature or severity of which is not consistent with the applicable product information as documented in the Investigator's Brochure and which at the same time is regarded as potentially related to the administration of the investigational product.

7.3. Reporting Procedures for all Adverse Events

All AEs occurring during the study observed by the investigator or reported by the participant, whether or not attributed to study medication, will be recorded on the CRF. The following information will be recorded: description, date of onset and end date, severity, assessment of relatedness to study medication, action taken and the outcome of AEs.

AEs considered related to the study medication as judged by a medically qualified investigator or the sponsor will be followed until resolution or the event is considered stable and/or until the end of the follow-up period (4 years).

The severity of events will be assessed on the following scale: 1 = mild, 2 = moderate, 3 = severe. The relationship of AEs to the study medication will be assessed by a medically qualified investigator and discussed with the PI.

Any *pregnancy* occurring during the clinical study and the outcome of the pregnancy of female patients or fathered by trial participants, should be recorded and followed up for congenital abnormality or birth defect until delivery. Pregnancy itself is documented as AE, in case of hospitalization as SAE.

7.3.1. Reporting Procedures for Serious Adverse Events

The Data Monitoring Committee (DMC) will review all SAEs for the study at certain time-points of the trial. The DMC is detailed in Appendix B and may hold personal /TC/electronic meetings. The DMC will meet at regular intervals and consider:

- Occurrence and nature of adverse events
- Whether additional information on adverse events is required
- Consider taking appropriate action where necessary to halt the trial (see below)
- Act / advise on incidents occurring between meetings that require rapid assessment (e.g. SUSARs)

Especially before the second and third trial cohort, when the Day 30 results for each patient are available, the DMC must meet and make a go/no-go decision for the treatment of the next dose cohort, which needs to be reported to the PI as soon as possible.
All SAEs will be reported to the DMC within one day of discovery or notification of the event. All SAE information will be recorded on an SAE form, which will be sent electronically to members of the DMC. Additional information received for a case (follow-up or corrections to the original case) will be detailed on a new SAE form.
The CRO will – on behalf of the Sponsor - report all AEs, SAEs and SUSARs to the Ethics Committee and the Regulatory Authority (PEI) in an annual safety report. Fatal or life-threatening SUSARs will be reported within 7 days and all other SUSARs within 15 days. The PI will also inform all members of the study group on all adverse events that might affect the safety of participants.

7.4. Rules for discontinuation of the trial

Any of the following events will result in discontinuation of the trial:

5. Severe loss of vision (more than 15 letters ETDRS acuity) occurring deemed to be a result of vector administration.
6. Severe ocular, vector-induced inflammation, unresponsive to treatment (endophthalmitis).
7. Any suspected unexpected serious adverse reaction (SUSAR).
8. Safety concerns other than those mentioned under 1-3 and consecutive request for discontinuation by the Data Monitoring Committee.

If an Adverse Event was the reason for discontinuation of the trial either by the PI or the participant, the participant must undergo an early termination visit (see flowchart). The patient must be given appropriate care under medical supervision until symptoms cease or the condition becomes stable. Maximal follow-up duration will be 1 year after trial termination

7.5. Risk-Benefit Considerations

The **risks** of the subretinal vector injection are related to 1) the surgical procedure three port pars plana vitrectomy and subretinal surgery as well as 2) the ophthalmic or systemic effects of the rAAV.hCNGA3 vector.
Regarding 1) there may be a potential loss of visual function due to complications of the surgical procedure such as bacterial infection, retinal detachment, suprachoroidal and/or subretinal hemorrhage and cataract as a consequence of the surgical trauma. These risks are treatable by standard ophthalmological care but may result in lack of partial or complete restoration of the visual function loss.
Table 2 gives an overview of known risks by pars plana vitrectomy based on the literature and experiences from NHP trials with the vector of this study and estimates the rates to be expected in the current trial. In case of the occurrence of any AE/complication listed in table 2, these will be treated according to standard of ophthalmological care.

**Surgical complications during 23g PPV**	Overall rate with mixed indications [1-3]	Incidence in NHP study	Predicted risk in gene therapy trial
retinal detachment / breaks	1-10 %	0%	1%
endophthalmitis	0.01-1 %	0%	0.01-1 %
wound leakage / transient hypotony	5-20 %	100%	5-20 %
choroidal hemorrhage / detachment	< 1 %	0%	< 1 %
suprachoroidal perfusion	< 1 %	0%	< 1 %
vitreal hemorrhage	1-5%	0%	< 1 %
cataract formation	1-5%	9%	10-20%
transient intraocular hypertension	5%	0%	5%
corneal erosion	5-20 %	5-20 %	5-20 %

Table 2: *Risks of complications during 23g PPV referring to literature, previous NHP studies and expected rates for the planned trial. [1] Wilkinson et al., 2013 [2]* Lee et al., 2012 *[3] Wykoff et al., 2010.*

Regarding 2) there is a risk for a loss of vision due to ophthalmic immune reactions to the vector (indirect) or due to direct effects of the vector (however, pre-clinical

studies showed no toxic effects with up to 10 fold higher concentrations). If any immune reaction should be triggered by the vector, this might include non-infectious inflammation of the pigment epithelium (epitheliitis), retina (retinitis), vitreous cavity (vitritis) or uveal tissue (uveitis). None of these have been reported in any previous clinical trial involving AAV in ocular gene transfer.

Such effects may result in a significant deterioration of visual function (decrease in VA of ≥ 15 letters) and might also occur after initial improvement. A vulnerable period for direct and/or indirect (immunogenic) effects due to the viral packaging protein of the vector would be the first two weeks (time of concomitant steroid treatment). No clinical trial has as of yet reported immune-reaction to the transgene (even in systemic gene transfer). It therefore seems unlikely that a direct or indirect effect due to transgene expression would occur after ocular gene transfer in the immune-privileged subretinal space.

Previous clinical trials with the same viral vector build have demonstrated good safety profiles (Nathwani et al., 2011). Other trials have shown good safety profiles after subretinal application of similar viral vector constructs (rAAV2) (Bainbridge et al., 2008; Hauswirth et al., 2008; Maguire et al., 2008; Maguire et al., 2009; Bennett et al., 2012; Jacobson et al., 2012). However, systemic risks cannot be completely ruled out and could stem from - previously undetected - **immune reactions**. Both the virus capsid and transgenic protein could potentially activate the immune system. The immune-privilege of the eye severely limits the likelihood of antigen presentation to leukocytes. The compartmentalization of the eye limits the biodistribution of viral vector. Much lower doses are required in the eye compared to previous studies targeting the liver (Nathwani et al., 2011). The transgenic protein is not secreted but expressed intracellularly and only in target cells (cone photoreceptors) due to a cone photoreceptor specific promoter. In light of these arguments and supported by results from previous clinical trials, it seems unlikely that a significant immune reaction is staged after intervention. Nevertheless, as immune reactions cannot be ruled out completely, patients will be screened accordingly during the follow-up clinical examinations. Any systemic reactions may appear during the first days as headache and fever. For safety reasons, patients will be hospitalized for three days including monitoring over the first night. Blood pressure, pulse and body temperature will be measured every 4 hours on day 1 post-op. These parameters will be measured twice at day 2 post-op. For hygiene procedure see 7.6.

The potential of **malignancy** due to the AAV vector is also very unlikely. In contrast to e.g. lentiviral vectors, rAAV are considered non-integrating and result in episome formation in the transduced cells rather than integrating the transgene cassette in the host genome. Wild-type AAVs carry the complete virus genome including re *Rep* genes, which orchestrate the integration of vector DNA (preferentially at the AAVS1 locus (c19q13.3)). Deletion of AAV *Rep* in the recombinant vector reduces these integration events by more than 99.5% (Schnepp et al., 2003). So far, there has been no report on insertional mutagenesis of recombinant AAV (Lipinski et al., 2013).

Nevertheless, all patients in this trial will be screened regularly even after study close out.

Subjects will be encouraged to monitor themselves and to assist in reporting adverse events; they will be provided with laminated wallet-sized cards with investigator contact information. Additionally, health care professionals treating the study patients, who are not otherwise associated with the clinical trial, will be notified to provide prompt reports of adverse events to the investigators. Investigators will maintain in the case history records of exposures to mutagenic agents and other medicinal products along with subjects' adverse event profiles. Clinical information will focus on information pertaining to new malignancies, new incidence or exacerbation of a pre-existing neurological disorder, new incidence or exacerbation of a prior rheumatologic or other autoimmune disorder, and new incidence of a hematologic disorder. For the subsequent years, subjects will be contacted at a minimum of once per year. A clinical questionnaire, administered by telephone call or at the eye hospital Tübingen or at the subject's home ophthalmological treatment centre, will focus on information pertaining to new malignancies, new incidence or exacerbation of a pre-existing neurological disorder, and new incidence or exacerbation of visual disturbances. It will ask subjects to describe any adverse events, including any unexpected illness and/or hospitalization, and provide a description of exposures to mutagenic agents and other medicinal products.

Dissemination of rAAV.hCNGA3 would most likely only occur between human beings, since it is derived from AAV2/8. However no replication is expected in normal cells of treated individuals exposed to the replication-deficient virus, or from exposure of uninfected people to treated individuals.

Germline transmission is theoretically possible but requires biodistribution far beyond what could be detected today combined with the extremely unlikely event of genomic integration.

Currently, there is no experience with the specific **teratogenity** of the rAAV.hCNGA3 vector, therefore the risk for an unborn child in case of an unforeseen conception, especially during the first half of the study participation, cannot be judged.

Generally, there is the potential of no subjective or objective benefit despite the risks of the procedure.

Patient **benefits** may consist in a deceleration or even halt of cone photoreceptor degeneration and in the initiation of cone photoreceptor function. This might include improvement of visual acuity, decreased glare, increased ability of color discrimination and decreased nystagmus. Especially the debilitating glare is a major impairment in achromatopsia patients' daily life. In addition, many patients are hampered by their very low vision. Beyond glare and visual acuity, novel colour perceptions may be an individual gain for the patients and their performance in daily

life. Improvements in visual functions are known to increase the vision-related quality of life and general well-being.

7.6. Risks for the Environment

These are dealt with in detail in the separate document *Information required concerning releases of genetically modified organisms* according to Annex IIIa of the DIRECTIVE 2001/18/EC OF THE EUROPEAN PARLIAMENT AND OF THE COUNCIL of 12 March 2001 on the deliberate release into the environment of genetically modified organisms and repealing Council Directive 90/220/EEC.

Dissemination of rAAV.hCNGA3 would most likely only occur between human beings, since it is derived from AAV2/8. However no replication is expected in normal cells of treated individuals exposed to the replication-deficient virus, or from exposure of uninfected people to treated individuals.

Safety countermeasures will be taken for the clinical phase I trial with regard to the results mentioned above. Until day 7 post-injection, any materials and tissues coming into contact with the patient's eyes, lachrymal fluid and nasal secretions will be collected in designated collecting boxes and will be subject to disinfection or autoclaving respectively (for more details see SOP waste treatment). After hospitalization patients will get collecting boxes to collect the materials mentioned above also in the hotel or at home. The boxes will be collected by the study team for destruction. Study patients will be instructed to not manipulate the eye after surgery to facilitate rapid closure of the self-sealing sclerotomies. Study patients will also be instructed to avoid intimate physical contact to other subjects until day 7 post-injection (See also section 1.3.3.).

8. Examinations

All study visits described in the flowchart, page 14, and in section 8.1. may be performed on two consecutive days; screening and close-out visit on three days if deemed necessary. The reason behind this is to reduce the workload due to concentration during test procedures and to improve the reliability of the test results.

8.1. Screening Examinations

(O) = optional, if control deemed necessary by investigator

Visit 1
Screening (Day -30 to -2)

- Medical/surgical history
- Ocular/surgical history
- BMI
- Vital Signs
- Urine pregnancy test

- Hematology/basic chemistry/urine analysis
- CRP, IgG, IgM
- Immunopathology
- PCR of rAAV8 genome
- Best corrected visual acuity
- Basic ophthalmological exam (miosis)
- Contrast sensitivity (PR Charts)
- Flicker Fusion Frequency
- Colour constancy (chromatic adaption)
- Anomaloscopy
- Cambridge Colour Test / Farnsworth D15
- IR Video Pupillography
- MP-1 Microperimetry (20°)
- Funduscopy (mydriasis)
- Dark adaption test
- ffERG (scotopic and photopic)
- sd-OCT
- Fundus autofluorescence
- Fundus photography
- Angiography
- Systemic steroids (Day -1)
- Topical steroids and antibiotics (Day -1)
- VFQ25
- Psychiatric examination
- Brief Symptom Inventory (BSI)
- Concomitant medication
- HIV Test

8.2. Trial Examinations

Visit 2

D0

- Vital signs
- Urine pregnancy test
- Hematology/ basic chemistry (O)
- Basic ophthalmological exam (miosis)
- Funduscopy
- sdOCT (O)
- Systemic steroids
- Topical steroids and antibiotics
- Subretinal injection of rAAV8 vector
- Psychological counselling
- Adverse event recording
- Concomitant medication

Visit 3
D1
- Vital signs (O)
- Hematology/ basic chemistry (O)
- Basic ophthalmological exam (miosis)
- Funduscopy (mydriasis)
- sdOCT (O)
- Systemic steroids
- Topical steroids and antibiotics
- Psychological counselling
- Adverse event recording
- Concomitant medication

Visit 4
D2
- Vital signs (O)
- Hematology/ basic chemistry (O)
- CRP, IgG, IgM
- Basic ophthalmological exam
- Funduscopy (mydriasis)
- sdOCT (O)
- Systemic steroids
- Topical steroids and antibiotics
- Psychological counselling
- Adverse event recording
- Concomitant medication

Visit 5
D3 (± 1 Day)
- Vital signs
- Hematology/ basic chemistry
- CRP, IgG, IgM
- PCR of rAAV8 genome
- Basic ophthalmological exam (miosis)
- Funduscopy (mydriasis)
- Contrast sensitivity (PR charts)
- sdOCT (O)
- Systemic steroids
- Topical steroids and antibiotics
- Psychological counselling
- Adverse event recording
- Concomitant medication

Visit 6
D14 (± 3 days)

- Vital signs (O)
- Urine pregnancy test
- Hematology/ basic chemistry
- CRP, IgG, IgM
- PCR of rAAV8 genome
- Basic ophthalmological exam (miosis)
- Visual Acuity, BCVA (ETDRS)
- Contrast sensitivity (PR charts)
- Anomaloscopy
- Cambridge Colour Test / Farnsworth D15
- Funduscopy
- sdOCT
- FAF
- FF
- Angiography (O)
- Anomaloscopy
- Roth 28-hue test (sat. and de-sat.)
- IR-Video-Pupillography
- MP-1 Microperimetry (20°)
- Dark adaptation test (FST)
- Topical steroids and antibiotics
- Brief Symptom Inventory (BSI)
- Psychological counselling
- Study specific scale, A3-PRO
- Adverse event recording
- Concomitant medication

Visit 7
D30 (± 5 days)

- Vital signs (O)
- Urine pregnancy test
- Hematology/ basic chemistry (O)
- CRP, IgG, IgM
- Immunopathology
- PCR of rAAV8 genome (O)
- Visual Acuity, BCVA (ETDRS)
- Basic ophthalmological exam (miosis)
- Contrast sensitivity (PR charts)
- Flicker Fusion Frequency
- Colour Constancy (chromatic adaption) (O)
- Anomaloscopy
- Cambridge Colour Test / Farnsworth D15
- IR Video Pupillography

- MP-1 Microperimetry (20°)
- Funduscopy (mydriasis)
- Dark adaptation test
- ff-ERG (scotopic and photopic)
- sdOCT
- FAF
- FF
- Angiography (O)
- Topical steroids and antibiotics
- VFQ25
- Brief Symptom Inventory (BSI)
- Psychological counselling
- Study specific scale, A3-PRO
- Adverse event recording
- Concomitant Medication

Visit 8
D90 (± 7 days)

- Vital signs (O)
- Urine pregnancy test
- Hematology/ basic chemistry (O)
- CRP, IgG, IgM (O)
- Immunopathology
- PCR of rAAV8 genome (O)
- Visual Acuity, BCVA (ETDRS)
- Basic ophthalmological exam
- Contrast sensitivity (PR charts)
- Flicker Fusion Frequency
- Colour Constancy (chromatic adaption) (O)
- Anomaloscopy
- Cambridge Colour Test / Farnsworth D15
- IR Video Pupillography
- MP-1 Microperimetry (20°)
- Funduscopy (mydriasis)
- Dark adaptation test
- ff-ERG (scotopic and photopic) (O)
- sdOCT (O)
- FAF (O)
- FF
- Angiography
- Brief Symptom Inventory (BSI)
- Psychological counselling
- Study specific scale, A3-PRO
- Adverse event recording
- Concomitant medication

Visit 9
D180 (± 7 days)

- Vital signs (O)
- Urine pregnancy test
- Hematology/ basic chemistry
- CRP, IgG, IgM (O)
- Immunopathology (O)
- PCR of rAAV8 genome (O)
- Visual Acuity, BCVA (ETDRS)
- Basic ophthalmological exam
- Contrast sensitivity (PR charts)
- Flicker Fusion Frequency
- Colour Constancy (chromatic adaption)
- Anomaloscopy
- Cambridge Colour Test / Farnsworth D15
- IR Video Pupillography
- MP-1 Microperimetry (20°)
- Funduscopy (mydriasis)
- Dark adaptation test
- ff-ERG (scotopic and photopic) (O)
- sdOCT (O)
- FAF (O)
- FF (O)
- Angiography (O)
- VFQ25
- Brief Symptom Inventory (BSI)
- Psychological counselling
- Study specific scale, A3-PRO
- Adverse event recording
- Concomitant medication

Visit 10
D365, Close out (± 14 days)

- BMI
- Vital signs
- Urine pregnancy test
- Hematology/ basic chemistry
- CRP, IgG, IgM (O)
- Immunopathology (O)
- PCR of rAAV8 genome (O)
- Visual Acuity, BCVA (ETDRS)
- Basic ophthalmological exam
- Contrast sensitivity (PR charts)
- Flicker Fusion Frequency
- Colour Constancy (chromatic adaption)

- Anomaloscopy
- Cambridge Colour Test / Farnsworth D15
- IR Video Pupillography
- MP-1 Microperimetry (20°)
- Funduscopy (mydriasis)
- Dark adaptation test
- ff-ERG (scotopic and photopic)
- sdOCT
- FAF
- FF
- Angiography
- VFQ25
- Brief Symptom Inventory (BSI)
- Psychological counselling
- Study specific scale, A3-PRO
- Adverse event recording
- Concomitant medication

8.3. Follow-up Examinations

Visit 11
Fup 1 M24 (± 1 month)

- Vital signs (O)
- Hematology/ basic chemistry
- CRP, IgG, IgM (O)
- Immunopathology (O)
- PCR of rAAV8 genome (O)
- Visual Acuity, BCVA (ETDRS)
- Basic ophthalmological exam
- Contrast sensitivity (PR charts)
- Anomaloscopy (O)
- Cambridge Colour Test / Farnsworth D15 (O)
- IR-Video-Pupillography (O)
- MP-1 Microperimetry (O)
- Funduscopy (mydriasis)
- Dark adaption test (O)
- ff-ERG (scotopic and photopic) (O)
- sdOCT
- FAF
- FF
- Angiography (O)
- VFQ25 (O)
- Study specific scale, A3-PRO
- Adverse event recording
- Concomitant medication

Visit 12
Fup 2 M36 (± 1 month)

- Vital signs (O)
- Hematology/ basic chemistry
- CRP, IgG, IgM (O)
- Immunopathology (O)
- PCR of rAAV8 genome (O)
- Visual Acuity, BCVA (ETDRS)
- Basic ophthalmological exam
- Contrast sensitivity (PR charts)
- Anomaloscopy (O)
- Cambridge Colour Test / Farnsworth D15 (O)
- IR-Video-Pupillography (O)
- MP-1 Microperimetry (O)
- Funduscopy (mydriasis)
- Dark adaption test (O)
- ff-ERG (scotopic and photopic) (O)
- sdOCT
- FAF
- FF
- Angiography (O)
- VFQ25 (O)
- Study specific scale, A3-PRO
- Adverse event recording
- Concomitant medication

Visit 13
Fup 3 M48 (± 1 month)

- Vital signs (O)
- Hematology/ basic chemistry
- CRP, IgG, IgM (O)
- Immunopathology (O)
- PCR of rAAV8 genome (O)
- Visual Acuity, BCVA (ETDRS)
- Basic ophthalmological exam
- Contrast sensitivity (PR charts)
- Anomaloscopy (O)
- Cambridge Colour Test / Farnsworth D15 (O)
- IR-Video-Pupillography (O)
- MP-1 Microperimetry (O)
- Funduscopy (mydriasis)
- Dark adaption test (O)
- ff-ERG (scotopic and photopic) (O)
- sdOCT
- FAF
- FF

- Angiography (O)
- VFQ25 (O)
- Study specific scale, A3-PRO
- Adverse event recording
- Concomitant medication

Visit 14
Fup 4 M60 (± 1 month)

- Vital signs (O)
- Hematology/ basic chemistry
- CRP, IgG, IgM (O)
- Immunopathology (O)
- PCR of rAAV8 genome (O)
- Visual Acuity, BCVA (ETDRS)
- Basic ophthalmological exam
- Contrast sensitivity (PR charts)
- Anomaloscopy (O)
- Cambridge Colour Test / Farnsworth D15 (O)
- IR-Video-Pupillography (O)
- MP-1 Microperimetry (O)
- Funduscopy (mydriasis)
- Dark adaption test (O)
- ff-ERG (scotopic and photopic) (O)
- sdOCT
- FAF
- FF
- Angiography (O)
- VFQ25 (O)
- Study specific scale, A3-PRO
- Adverse event recording
- Concomitant medication

8.4. Non-ophthalmological and ophthalmological Procedures

All procedures should be performed in the sequence given by the flowchart, whenever possible. If feasible, the same investigators should perform the examinations for the study duration. Advanced test procedures, such as, but not limited to BCVA, microperimetry and pupillography, will be performed by study personnel after method-specific training.
If not specified differently, all tests described here will be performed according to the Flowchart of Visits and Procedures in *both* eyes.
All ophthalmological procedures will be performed in a fixed sequence, testing the study eye first and the fellow eye afterwards.

More details of all examination procedures can be found in the Procedure Manual in its current version as approved by the Principal Investigator.

8.4.1. Medical / surgical history

Essential events have to be documented in the patient file / worksheet with dates of onset and underlying pathology.

8.4.2. Ocular / surgical history

Any ophthalmological disease or surgery in either eye has to be documented with date of onset.

8.4.3. Body Mass Index

Deriving from height and weight of the patient, the BMI (kg/m^2) will be calculated.

8.4.4. VIIs

After 10 minutes resting in a sitting position blood pressure, heart rate and body temperature will be measured.

8.4.5. Urine pregnancy test

In female subjects with childbearing potential a commercial urine pregnancy test will be performed by the study nurse / study coordinator at all marked visits.

8.4.6. Hematology, basic chemistry and urine analysis

The following parameters will be tested at those visits where blood and urine tests are foreseen:

- Sedimentation rate
- Differential Blood count
- Hemoglobin
- Hematocrit
- Platelet count
- Glucose
- Sodium
- Potassium
- Uric acid
- Creatinine
- Creatinine kinase
- LDH
- Total bilirubin
- Direct bilirubin
- AST (SGOT)

- ALT (SGPT)
- γGT
- Alkaline phosphatase
- Proteine electrophoresis
- Coagulation (INR, PTT, aPTT)
- Urine analysis

8.4.7. CRP, Immunoglobulines

CRP, IgG and IgM will be determined at all marked study visits.

8.4.8. Immunopathology

In a specialized local lab (Prof. Specht) anti-AAV8 capsid ELISA will be determined.

8.4.9. PCR of rAAV8 genome

A search for potential systemic rAAV8 virus genome will be performed at early study visits and later on if a control is deemed necessary by the investigator.

Ophthalmological procedures

All tests described will be performed in a fixed order. The treated eye will be examined first, followed by the untreated eye.

8.4.10. Best corrected visual acuity

Best corrected visual acuity will be quantified using the ETDRS visual acuity protocol as described in the Procedure Manual.

8.4.11. Basic ophthalmological examination

After determination of visual acuity, outer eye segments will be inspected and the anterior segments of the eye will be examined using a slit-lamp.

8.4.12. Contrast sensitivity (Pelli Robson Charts)

This test for contrast sensitivity will be performed in both eyes at standardized illumination conditions, according to the Procedure Manual of the Pelli Robson (PR) chart. In contrast to the user instruction of the PR chart, the test distance will be 3m (instead of 1m), as own data have shown, that the standard distance of 1m is inferior in sensitivity.

8.4.13. Flicker Fusion Frequency

The Flicker Fusion Frequency is used to test cone function independent of selective amblyopia problems eventually present. The subject increases flicker frequency until disappearance of flicker sensation.

8.4.14. Colour constancy

Patients are asked to judge the chromatic appearance of a hexagonal test patch within a multi-coloured display at photopic levels. More details are described in the Procedure Manual.

8.4.15. Anomaloscopy

The Nagel anomaloscope can differentiate between six pathological findings i.e. colour deficiencies and will be used in the trial to investigate baseline and possible restitution of colour discrimination. The Rayleigh-equation quantitatively evaluates results.

8.4.16. Cambridge Colour Test / Farnsworth D15

Chromatic discrimination thresholds will be determined using the low-vision version of the Cambridge Colour Test as described in more detail in the Procedure Manual. The stimulus array consists of discrete dots of variable random size and luminance. The target is a subset of dots, which delineate a large 5-degree square varying in saturation. Patients indicate the location of the coloured square in an alternative forced choice procedure.

The D15 set is a modification of the well-known Farnsworth-Munsell 100 Hue Test. The D15 test is intended for classification instead of more time-consuming in-depth study of color vision defects using the 100-Hue test. Each D15 set contains a reference disc and fifteen numbered discs, which make up an incomplete color circle. Following an attempt to sequentially arrange the discs by the patient, evaluation determines color perception or defects in deutan, protan or tritan axis discrimination. Sometimes there are indeterminate defects in the cases of retinal toxicity.

8.4.17. Infrared-Video-Pupillography

Pupillography is used for the objective efficacy assessment after intervention. Chromatic Pupillography will be performed at various time-points during the study as outlined in the flowchart of visits. Standardized adaptation, background illuminance, stimulus wavelength and duration need to be considered according to the Procedure Manual. Consensual measurement is performed, i.e. study eye is always stimulated while pupillary reactions of the fellow eye are recorded monocularly.

8.4.18. Microperimetry

Microperimetry tests retinal sensitivity by projecting defined light stimuli to specific locations within the macula under mesopic conditions. The subjects are asked to press a button when a stimulus is perceived. Involuntary eye movements are corrected in real-time and fixation stability is quantified. This test will be performed according to the Procedure Manual.

Note: All following tests will be performed in pharmacologically induced mydriasis (2.5% phenylephrin, 0.5% tropicamid eye drops) only.

8.4.19. Fundoscopy

Stereoscopic biomicroscopy of the full fundus is performed using a 90 Dpt lens (Volk) or equivalent to check for signs of inflammation and retinal detachment.

8.4.20. Dark adaptation (DA)

After 3 minutes of bleaching with bright white light (pupil dilation), a staircase procedure is used to estimate detection thresholds for red and green. The whole measurement lasts for approx. 40 minutes.

8.4.21. Ganzfeld electroretinogram (ERG)

The Ganzfeld electroretinogram (ERG) is the record of diffuse electrical response to light stimuli, generated by neural and non-neural cells in the retina. Depending on recording conditions (i.e. scotopic or photopic), stimulus intensity, wavelength, frequency or stimulus duration, the function of various retinal cells can be isolated. Further details of the test procedures are described in the Procedure Manual.

8.4.22. Spectral domain optical coherence tomography (SD-OCT)

Patients will be examined in mydriasis using a Spectralis HRT+OCT system (Heidelberg Engineering, Heidelberg, Germany). Briefly, perpendicular line and volume scans are recorded to quantify micro-anatomical changes in the macula before (baseline) and after (follow up) treatment. The patient is asked to look at a visual cue while eye-tracking software will correct for involuntary eye movement. Baseline recordings will be identified to allow precise follow-up recordings based on the same anatomical landmarks used for eye-tracking. The untreated eye will be recorded first, followed by the treated one.

8.4.23. Fundus autofluorescence (FAF)

The Spectralis HRT+OCT system described above will be used in the same session to record FAF, a measure of retinal pigment epithelial metabolism and viability (Schmitz-Valckenberg et al., 2008). Central 55° recordings will be made with optional extensions towards the outer limits of the treated area, if they are not covered by the central recordings. The untreated eye will be recorded first, followed by the treated one.

8.4.24. Fundus photography (FP)

Fundus of all patients will be recorded in mydriasis for documentation of posterior segment changes. This includes 9 separate wide-angle recordings (central, superior, superior-nasal, nasal, nasal-inferior, inferior, temporal-inferior, temporal, temporal-superior). The untreated eye will be recorded first, followed by the treated one.

8.4.25. Angiography

ICG Angiography

Indocyanine Green is a dye, which fluoresces in the infra-red light. The infra-red waves have the ability to penetrate the retinal layers making the circulation in deeper layers visible when photographed with an infra-red sensitive camera. The dye is applied by intravenous injection and flows through the body to reach the choroidal and retinal circulation. Due to its nature it stays in the retinal and choroidal vessels, this allows to see and identify the distinct outlines of the vessels of the choroid.

Fluorescein Angiography (FA)

Perfusion characteristics will be assessed in mydriasis using a wide-angle objective after intravenous bolus administration of 10% fluorescein sodium (500mg in 5ml; Novartis Pharma AG, Bern, Switzerland).

The central fundus of the treated eye will be recorded with sequential photographs in the first 45 seconds after injection. At 1 and 5 minutes after injection the same 9-view recordings will be performed in both eyes.

8.4.26. Topical steroids and antibiotics

Moxifloxacin eye drops 0.5% are administered 4 times a day and dexamethasone gel 0.5% is administered 4 times a day for 21 days, starting at day -1.

8.4.27. Systemic steroids

Systemic steroids will be given orally at 1.0mg/kg for approximately 3 weeks starting at day -1 and then tapered off after day 19 at the discretion of the investigator (e.g. 40/30/20/15/10mg).

8.4.28. Subretinal injection of rAAV8.hCNGA3 vector

The study treatment is described in section 6 briefly and in detail in the Procedure Manual.

8.4.29. VFQ25

The NEI-VFQ-25 is a 25-item vision-specific questionnaire that collects information about how the patient's vision affects his/her life in 3 areas (General Health and Vision, Difficulty with Activities, Responses to Vision Problems). The NEI-VFQ-25 assesses the influence of visual impairment on functioning and specific aspects of health-related quality of life. The interviewer-administered version of the NEIVFQ- 25 will be used for all patients and will be interview-administered by the study site.

8.4.30. Psychiatric examination

A basic psychiatric examination is performed at screening to ensure that the patient does not suffer from a psychiatric disorder and is not at risk for recurrence of such a disorder. Patients should not be under high pressure due to achromatopsia and/or have high and unrealistic expectations regarding the treatment outcome.

8.4.31. Brief symptom inventory (BSI)

The brief symptom inventory assesses psychological stress and is administered as a questionnaire in interview version. It will be performed by a psychologist or a psychiatrist.

8.4.32. Psychological counselling

The patient has the possibility of psychological counselling with a specialist who does not belong to the study team.

8.4.33. Study specific scale, A3-PRO

In addition to the widely used VFQ25 (see above), which may not be sensitive for the treatment effects in the study population, a study-specific scale was developed in an interview version to be used at selected study visits. A3-PRO assesses the subjectively perceived effects of the treatment.

8.4.34. Adverse event (AE) recording

Open questions regarding changes in health status since last visit are asked by the study physician at the beginning of each study visit.

8.4.35. Concomitant medication

At each visit changes in dosing / medication are recorded by the study staff.

8.4.36. HIV Test

Blood for a HIV test will be withdrawn

9. Documentation of Trial Data

All original study data from all screened and enrolled patients will be entered into the electronical study database. Original hardcopy documents will need to be signed and dated. GCP-conform monitoring is performed by the CRO of the study as agreed in the monitor manual with the principal investigator on behalf of the sponsor. Any correction on study documents needs to be initialized and dated. All handwriting must be readable. Incorrect entries must still be readable but striked out.

10. Biometrical Planning and Analysis

10.1. Trial Design

This is an open, mono-center trial with fellow-eye comparison.
Due to the characteristics of a phase I trial a dose-escalation design was chosen and due to the fact that there is no effective, approved therapy and this is a safety trial no control condition is foreseen. As a consequence of this, there will be no blinding / randomization procedure. The development of visual function and morphology will be tested against the fellow-eye.
The small sample size, as well as the particularity and rareness of the disease CNGA3-related achromatopsia, led to a monocentric study concept.

10.2. Sample Size Issues

No statistical sample size estimation was performed as no data are available regarding the safety of rAAV.hCNGA3 (first dose in man) or any other effect in the eye for such a calculation. As three vector dosages are planned in a dose-escalating schedule we decided for three patients in each group. Drop-outs will only be replaced up to a number of two patients in case that other reasons than vector- or procedure-related adverse events are the underlying reasons for the drop-out.

10.3. Definition for Study Groups

Study populations are defined in the following subsections. All populations will be identified and finalized in the Statistical Analysis Plan.

10.3.4. Safety Population

The Safety Population is defined as all subjects who receive at least one dose of investigational product or corticosteroids and have at least one post-therapy safety assessment.

10.3.5. Intent to Treat Population

The Intent to Treat (ITT) population is defined as all subjects who are enrolled and received at least one dose of investigational product or corticosteroids, and for whom at least one post-baseline assessment is available.

10.3.6. Per Protocol Population

The Per Protocol (PP) Population is a subset of the ITT Population and consists of subjects for whom no major protocol violations have been reported. The criteria for defining the PP set will be fully defined in the Statistical Analysis Plan.

A final decision for the allocation to the different populations of the subjects will be made before analysis of trial results.

10.4. Statistical Analysis

AEs and SAEs will be documented using line listings and tabulations (MetraCad code will be applied). Descriptive analysis will include line charts for individual patients. Standard errors and means will be displayed also using line charts. This will be done for the raw measurements and for the difference between treated and untreated control eye within each patient.
Descriptive parameters will be given for the full cohort (n=9) but not for the sub-cohorts (n=3).
Additionally correlation analysis will be performed in a purely descriptive manner for the comparison of subjective and objective measurements. For each subject and measurement, the delta of the value for the interventional eye minus the mean of control eye values over time will be determined. In scatter plots for pairs of variables (measurements pooled over subjects) the association between these deltas will be displayed.

10.5. Interim Analyses

An interim analysis is foreseen – beyond those of adverse events for the DMC and annual safety reports – in March/April 2017 in the process of negotiations with industrial sponsors. Those sponsors have an interest in joining the development of rAAV.hCNGA3 and bringing the treatment towards and into a phase III clinical trial.

11. Anticonception Rules and Pregnancy

Experiences regarding transfection to the germline are available from application of similar AAV vectors.
Preclinical Data. Systemic application - predominantly in rodents - showed no transfection of the germ line (Alemany and Curiel, 2001; Peng et al., 2001; Peters et al., 2001; Gonin and Gaillard, 2004). Also for systemic application, Arruda et al. (Arruda et al., 2001) detected dose-related AAV vector sequences in DNA extracted from rabbit in testes, but not in semen. In addition, in vitro attempts to directly infect isolated murine spermatogonia with a rAAV vector failed (Arruda et al., 2001).
Schuettrumpf et al. (Schuettrumpf et al., 2006) - investigating effects in the rabbit to doses of $1x10^{11}$ – 10^{13} vg/kg i.v. – found transient vector dissemination to semen until day 4 post application, while long-term follow-up did not reveal any transduction of the spermatogenesis. Favaro et al. (Favaro et al., 2009) used doses of $1x10^{12}$ – 10^{13} vg/kg and detected virus genome until week 10 post injection (rabbit). In analyses in rabbits after intravenous rAAV doses ranging from 10e11 to 10e13 vector genomes/kg (doses similar to those used in a human clinical trial), a dose-dependent increase in PCR positive vector sequences was seen in semen samples; however, at no time point infectious AAV particles could be recovered from the semen samples (Ariuda et al., 2003).
When exposing the germline of rodents to AAV vectors directly several investigators did not find transfection of germ cells nor of foetuses in the case of test-tube fertilization (Gordon, 2001; Couto et al., 2004; Kojima et al., 2008). Laurema et al. (Laurema et al., 2003) exposed pregnant rabbits to AAV vectors (arteria uterina, $1x10^{10}$ vgp) and found that premature egg cells (without zona pellucida) were transfected 3 respective 8 days after gene therapy.
Clinical Data. Experiences in humans are available for non-ocular applications of AAV vectors. After i.v. injection with a twenty times higher viral load compared to our planned trial, only non-infectious particles were found in semen until 16 weeks after treatment. If vectors had been applied intramuscular, no AAV virus genome was present in semen two months and four months respectively after injection (Kay et al., 2000; Manno et al., 2006). The trials cited above show that in all administration types, except intravenous delivery, no AAV genome could be found in semen samples after Day 7 after administration. None of the available ongoing clinical trials (see introduction) with ocular gene therapy have investigated rAAV shedding in the semen. No data has been published regarding germline transfection from other ocular gene therapy studies (neither preclinical nor clinical). In a personal communication with Jean Bennett, Philadelphia, working with gene therapy in LCA patients our group was informed that there was no evidence of exposure to the reproductive track after subretinal application of a rAAV2 vector.

We therefore deem the need for contraception by female or male study participants with fertile partners for four months after the injection to be sufficient. Beyond the consideration of a desirable safety margin on one hand and avoidance of unnecessary constraints for the individual patient's life on the other, this time span is also in line with other ophthalmological gene therapy trials, such as in Leber's congenital amaurosis caused by RPE65 mutation, approved by the FDA.

13. Conditions for Amendments

In case of necessary changes to the flowchart of visits and procedures or other aspects of the trial, an amendment will be submitted to PEI and EC. Those changes will not be implemented unless approved by the PEI and EC. Exempted are changes to the protocol preventing immediate hazard for the patient. In this case implementation may occur before approval. Any amendment will result in a revised version of the patient information form, which needs to be signed by all patients.
A recommendation of the DMC may also result in an amendment to the trial protocol.

14. Ethical and Regulatory Aspects

The Investigator will be responsible for the overall conduct of the clinical trial and will be responsible for ensuring the trial is conducted according to the protocol and all regulatory requirements and regulations.

The protocol and informed consent form for this study must be approved by an appropriately constituted EC as defined by local requirements. The list of the EC voting members, their titles or occupation, and their institutional affiliations and/or the EC general assurance number, if applicable, must be provided with the approval.
The EC will also be notified of completion of the study and a final report must be submitted to the EC and the PEI in accordance with local requirements. The Investigator will maintain an accurate and complete record of all communications, reports and submissions to the PEI and EC.

This trial is subject to German AMG in its current version and will be submitted, performed and monitored according to ICH-GCP standards. Any study procedure will only be performed after the patient has given written informed consent. Informed consent will be documented in the electronic patient file of the Centre for Ophthalmology (AIS) giving the date of consent, study name and the name of the physician. Each subject's signed informed consent must be kept on file by the Investigator.
German data protection regulations will be followed and patients will be informed in a standardized and detailed manner about pseudonomyzed health data and about those persons/institutions who will have access to personal data under special restrictions according to law.

The Sponsor of this trial is UKT (Universitätsklinikum Tübingen) represented by the Principal Investigator. Defined tasks have been delegated to the CRO of the trial, STZ eyetrial at the Centre for Ophthalmology, University of Tübingen such as, but not limited to, communication with regulatory authorities, submission of the trial to PEI and EC, monitoring of the trial, safety reports. UKT has contracted insurance for trial participation as well as travel insurance.

The contact information for **patient insurance** are:

[redacted]

Contact information for **travel insurance** are:

[redacted]

The amount covered for the patient insurance is 500 000€ per patient. The amount covered for the travel insurance is 100 000€ for invalidity und 50 000€ in the case of death.

The patient has the obligation to immediately inform the insurance companies mentioned above, if necessary with the support of the Investigator. The patient also has to inform the investigator

All patients will receive a copy of the insurance police, along with the copy of the patient informed consent.

The documents used for submission and conduct of this trial will be according to the SOP system of the local CRO, STZ *eyetrial* at the Centre for Ophthalmology, which is a certified member of the EVICR.net.

15. References

Aboshiha, J., A. M. Dubis, J. Cowing, R. T. Fahy, V. Sundaram, J. W. Bainbridge, R. R. Ali, A. Dubra, M. Nardini, A. R. Webster, A. T. Moore, G. Rubin, J. Carroll and M. Michaelides (2014). A prospective longitudinal study of retinal structure and function in achromatopsia. *Invest Ophthalmol Vis Sci*, 55: 5733-5743

Alemany, R. and D. T. Curiel (2001). CAR-binding ablation does not change biodistribution and toxicity of adenoviral vectors. *Gene Ther*, 8: 1347-1353

Aligianis, I. A., T. Forshew, S. Johnson, M. Michaelides, C. A. Johnson, R. C. Trembath, D. M. Hunt, A. T. Moore and E. R. Maher (2002). Mapping of a novel locus for achromatopsia (ACHM4) to 1p and identification of a germline mutation in the alpha subunit of cone transducin (GNAT2). *J Med Genet*, 39: 656-660

Allocca, M., C. Mussolino, M. Garcia-Hoyos, D. Sanges, C. Iodice, M. Petrillo, L. H. Vandenberghe, J. M. Wilson, V. Marigo, E. M. Surace and A. Auricchio (2007). Novel adeno-associated virus serotypes efficiently transduce murine photoreceptors. *J Virol*, 81: 11372-11380

Ariuda, V. R., J. Schuettrumpf, L. JiangHua, K. Addya, D. Leonard, L. Couto, A. Chew, Z. Zhen, J. Sommer, R. W. Herzog, M. A. Kay, G. Bert, C. S. Manno and K. A. High (2003). Assessing the risk of inadvertent germline transmission of recombinant AAV-2 vector. . *Molecular Therapy*, 7: 161-162

Arruda, V. R., P. A. Fields, R. Milner, L. Wainwright, M. P. De Miguel, P. J. Donovan, R. W. Herzog, T. C. Nichols, J. A. Biegel, M. Razavi, M. Dake, D. Huff, A. W. Flake, L. Couto, M. A. Kay and K. A. High (2001). Lack of germline transmission of vector sequences following systemic administration of recombinant AAV-2 vector in males. *Mol Ther*, 4: 586-592

Asokan, A., D. V. Schaffer and R. J. Samulski (2012). The AAV vector toolkit: poised at the clinical crossroads. *Mol Ther*, 20: 699-708

Bainbridge, J. W., A. J. Smith, S. S. Barker, S. Robbie, R. Henderson, K. Balaggan, A. Viswanathan, G. E. Holder, A. Stockman, N. Tyler, S. Petersen-Jones, S. S. Bhattacharya, A. J. Thrasher, F. W. Fitzke, B. J. Carter, G. S. Rubin, A. T. Moore and R. R. Ali (2008). Effect of gene therapy on visual function in Leber's congenital amaurosis. *N Engl J Med*, 358: 2231-2239

Barnard, A. R., M. Groppe and R. E. MacLaren (2014). Gene Therapy for Choroideremia Using an Adeno-Associated Viral (AAV) Vector. *Cold Spring Harb Perspect Med*, 4:

Bennett, J., M. Ashtari, J. Wellman, K. A. Marshall, L. L. Cyckowski, D. C. Chung, S. McCague, E. A. Pierce, Y. Chen, J. L. Bennicelli, X. Zhu, G. S. Ying, J. Sun, J. F. Wright, A. Auricchio, F. Simonelli, K. S. Shindler, F. Mingozzi, K. A. High and A. M. Maguire (2012). AAV2 gene therapy readministration in three adults with congenital blindness. *Sci Transl Med*, 4: 120ra115

Bertelsen, M., H. Jensen, M. Larsen, B. Lorenz, M. N. Preising and T. Rosenberg (2013). Prevalence and diagnostic spectrum of generalized retinal dystrophy in Danish children. *Ophthalmic Epidemiol*, 20: 164-169

Black, A., V. Vasireddy, D. C. Chung, A. M. Maguire, R. Gaddameedi, T. Tolmachova, M. Seabra and J. Bennett (2014). Adeno-associated virus 8-mediated gene therapy for choroideremia: preclinical studies in in vitro and in vivo models. *J Gene Med*, 16: 122-130

Blackwell, H. R. and O. M. Blackwell (1961). Rod and cone receptor mechanisms in typical and atypical congenital achromatopsia. *Vision Research*, 1: 62-107

Burgueno-Montanes, C., M. Colunga Cueva and C. Costales Alvarez (2014). A novel mutation in the CNGA3 gene responsible for incomplete achromatopsia. *Arch Soc Esp Oftalmol*, 89: 107-109

Chang, B., T. Grau, S. Dangel, R. Hurd, B. Jurklies, E. C. Sener, S. Andreasson, H. Dollfus, B. Baumann, S. Bolz, N. Artemyev, S. Kohl, J. Heckenlively and B. Wissinger (2009). A

homologous genetic basis of the murine cpfl1 mutant and human achromatopsia linked to mutations in the PDE6C gene. *Proc Natl Acad Sci U S A*, 106: 19581-19586

Cideciyan, A. V., S. G. Jacobson, W. A. Beltran, A. Sumaroka, M. Swider, S. Iwabe, A. J. Roman, M. B. Olivares, S. B. Schwartz, A. M. Komaromy, W. W. Hauswirth and G. D. Aguirre (2013). Human retinal gene therapy for Leber congenital amaurosis shows advancing retinal degeneration despite enduring visual improvement. *Proc Natl Acad Sci U S A*:

Couto, L., A. Parker and J. W. Gordon (2004). Direct exposure of mouse spermatozoa to very high concentrations of a serotype-2 adeno-associated virus gene therapy vector fails to lead to germ cell transduction. *Hum Gene Ther*, 15: 287-291

Curcio, C. A., K. R. Sloan, R. E. Kalina and A. E. Hendrickson (1990). Human photoreceptor topography. *J Comp Neurol*, 292: 497-523

Favaro, P., H. D. Downey, J. S. Zhou, J. F. Wright, B. Hauck, F. Mingozzi, K. A. High and V. R. Arruda (2009). Host and vector-dependent effects on the risk of germline transmission of AAV vectors. *Mol Ther*, 17: 1022-1030

Ferreira, V., H. Petry and F. Salmon (2014a). Immune Responses to AAV-Vectors, the Glybera Example from Bench to Bedside. *Front Immunol*, 5: 82

Ferreira, V., J. Twisk, K. Kwikkers, E. Aronica, D. Brisson, J. Methot, H. Petry and D. Gaudet (2014b). Immune responses to intramuscular administration of alipogene tiparvovec (AAV1-LPL(S447X)) in a phase II clinical trial of lipoprotein lipase deficiency gene therapy. *Hum Gene Ther*, 25: 180-188

Flanagan, N. M., A. J. Jackson and A. E. Hill (2003). Visual impairment in childhood: insights from a community-based survey. *Child Care Health Dev*, 29: 493-499

Gonin, P. and C. Gaillard (2004). Gene transfer vector biodistribution: pivotal safety studies in clinical gene therapy development. *Gene Ther*, 11 Suppl 1: S98-S108

Gordon, J. W. (2001). Direct exposure of mouse ovaries and oocytes to high doses of an adenovirus gene therapy vector fails to lead to germ cell transduction. *Mol Ther*, 3: 557-564

Greenberg, J. P., J. Sherman, S. A. Zweifel, R. W. Chen, T. Duncker, S. Kohl, B. Baumann, B. Wissinger, L. A. Yannuzzi and S. H. Tsang (2014). Spectral-Domain Optical Coherence Tomography Staging and Autofluorescence Imaging in Achromatopsia. *JAMA Ophthalmol*:

Hauswirth, W. W., T. S. Aleman, S. Kaushal, A. V. Cideciyan, S. B. Schwartz, L. Wang, T. J. Conlon, S. L. Boye, T. R. Flotte, B. J. Byrne and S. G. Jacobson (2008). Treatment of leber congenital amaurosis due to RPE65 mutations by ocular subretinal injection of adeno-associated virus gene vector: short-term results of a phase I trial. *Hum Gene Ther*, 19: 979-990

Huckfeldt, R. M. and J. Bennett (2014). Promising first steps in gene therapy for choroideremia. *Hum Gene Ther*, 25: 96-97

Jacobson, S. G., A. V. Cideciyan, R. Ratnakaram, E. Heon, S. B. Schwartz, A. J. Roman, M. C. Peden, T. S. Aleman, S. L. Boye, A. Sumaroka, T. J. Conlon, R. Calcedo, J. J. Pang, K. E. Erger, M. B. Olivares, C. L. Mullins, M. Swider, S. Kaushal, W. J. Feuer, A. Iannaccone, G. A. Fishman, E. M. Stone, B. J. Byrne and W. W. Hauswirth (2012). Gene therapy for leber congenital amaurosis caused by RPE65 mutations: safety and efficacy in 15 children and adults followed up to 3 years. *Arch Ophthalmol*, 130: 9-24

Jeon, C. J., E. Strettoi and R. H. Masland (1998). The major cell populations of the mouse retina. *J Neurosci*, 18: 8936-8946

Kay, M. A., C. S. Manno, M. V. Ragni, P. J. Larson, L. B. Couto, A. McClelland, B. Glader, A. J. Chew, S. J. Tai, R. W. Herzog, V. Arruda, F. Johnson, C. Scallan, E. Skarsgard, A. W. Flake and K. A. High (2000). Evidence for gene transfer and expression of factor IX in haemophilia B patients treated with an AAV vector. *Nat Genet*, 24: 257-261

Kohl, S., B. Baumann, M. Broghammer, H. Jagle, P. Sieving, U. Kellner, R. Spegal, M. Anastasi, E. Zrenner, L. T. Sharpe and B. Wissinger (2000). Mutations in the CNGB3 gene

encoding the beta-subunit of the cone photoreceptor cGMP-gated channel are responsible for achromatopsia (ACHM3) linked to chromosome 8q21. *Hum Mol Genet*, 9: 2107-2116

Kohl, S., B. Baumann, T. Rosenberg, U. Kellner, B. Lorenz, M. Vadala, S. G. Jacobson and B. Wissinger (2002). Mutations in the cone photoreceptor G-protein alpha-subunit gene GNAT2 in patients with achromatopsia. *Am J Hum Genet*, 71: 422-425

Kohl, S., F. Coppieters, F. Meire, S. Schaich, S. Roosing, C. Brennenstuhl, S. Bolz, M. M. van Genderen, F. C. Riemslag, C. European Retinal Disease, R. Lukowski, A. I. den Hollander, F. P. Cremers, E. De Baere, C. B. Hoyng and B. Wissinger (2012). A nonsense mutation in PDE6H causes autosomal-recessive incomplete achromatopsia. *Am J Hum Genet*, 91: 527-532

Kohl, S., T. Marx, I. Giddings, H. Jagle, S. G. Jacobson, E. Apfelstedt-Sylla, E. Zrenner, L. T. Sharpe and B. Wissinger (1998). Total colourblindness is caused by mutations in the gene encoding the alpha-subunit of the cone photoreceptor cGMP-gated cation channel. *Nat Genet*, 19: 257-259

Kohl, S., B. Varsanyi, G. A. Antunes, B. Baumann, C. B. Hoyng, H. Jagle, T. Rosenberg, U. Kellner, B. Lorenz, R. Salati, B. Jurklies, A. Farkas, S. Andreasson, R. G. Weleber, S. G. Jacobson, G. Rudolph, C. Castellan, H. Dollfus, E. Legius, M. Anastasi, P. Bitoun, D. Lev, P. A. Sieving, F. L. Munier, E. Zrenner, L. T. Sharpe, F. P. Cremers and B. Wissinger (2005). CNGB3 mutations account for 50% of all cases with autosomal recessive achromatopsia. *Eur J Hum Genet*, 13: 302-308

Kojima, Y., Y. Hayashi, S. Kurokawa, K. Mizuno, S. Sasaki and K. Kohri (2008). No evidence of germ-line transmission by adenovirus-mediated gene transfer to mouse testes. *Fertil Steril*, 89: 1448-1454

Laurema, A., A. Heikkila, L. Keski-Nisula, T. Heikura, P. Lehtolainen, H. Manninen, T. T. Tuomisto, S. Heinonen and S. Yla-Herttuala (2003). Transfection of oocytes and other types of ovarian cells in rabbits after direct injection into uterine arteries of adenoviruses and plasmid/liposomes. *Gene Ther*, 10: 580-584

Lebherz, C., A. Maguire, W. Tang, J. Bennett and J. M. Wilson (2008). Novel AAV serotypes for improved ocular gene transfer. *J Gene Med*, 10: 375-382

Lee, G. W., S. J. Na, Y. H. Lee, S. Y. Jin and T. G. Lee (2012). Complication Incidence of Day Surgeries with 23 Gauge Vitrectomy. *J Korean Ophthalmol Soc*, 53: 1823-1827

Li, A., X. Zhu and C. M. Craft (2002). Retinoic acid upregulates cone arrestin expression in retinoblastoma cells through a Cis element in the distal promoter region. *Invest Ophthalmol Vis Sci*, 43: 1375-1383

Li, Q., R. Miller, P. Y. Han, J. Pang, A. Dinculescu, V. Chiodo and W. W. Hauswirth (2008). Intraocular route of AAV2 vector administration defines humoral immune response and therapeutic potential. *Mol Vis*, 14: 1760-1769

Lipinski, D. M., M. Thake and R. E. MacLaren (2013). Clinical applications of retinal gene therapy. *Prog Retin Eye Res*, 32: 22-47

Liu, H. S., M. S. Jan, C. K. Chou, P. H. Chen and N. J. Ke (1999). Is green fluorescent protein toxic to the living cells? *Biochem Biophys Res Commun*, 260: 712-717

Liu, M. M., J. Tuo and C. C. Chan (2011). Gene therapy for ocular diseases. *Br J Ophthalmol*, 95: 604-612

Maclaren, R. E., M. Groppe, A. R. Barnard, C. L. Cottriall, T. Tolmachova, L. Seymour, K. R. Clark, M. J. During, F. P. Cremers, G. C. Black, A. J. Lotery, S. M. Downes, A. R. Webster and M. C. Seabra (2014). Retinal gene therapy in patients with choroideremia: initial findings from a phase 1/2 clinical trial. *Lancet*:

Maguire, A. M., K. A. High, A. Auricchio, J. F. Wright, E. A. Pierce, F. Testa, F. Mingozzi, J. L. Bennicelli, G. S. Ying, S. Rossi, A. Fulton, K. A. Marshall, S. Banfi, D. C. Chung, J. I. Morgan, B. Hauck, O. Zelenaia, X. Zhu, L. Raffini, F. Coppieters, E. De Baere, K. S. Shindler, N. J. Volpe, E. M. Surace, C. Acerra, A. Lyubarsky, T. M. Redmond, E. Stone, J. Sun, J. W. McDonnell, B. P. Leroy, F. Simonelli and J. Bennett (2009). Age-dependent

effects of RPE65 gene therapy for Leber's congenital amaurosis: a phase 1 dose-escalation trial. *Lancet*, 374: 1597-1605

Maguire, A. M., F. Simonelli, E. A. Pierce, E. N. Pugh, Jr., F. Mingozzi, J. Bennicelli, S. Banfi, K. A. Marshall, F. Testa, E. M. Surace, S. Rossi, A. Lyubarsky, V. R. Arruda, B. Konkle, E. Stone, J. Sun, J. Jacobs, L. Dell'Osso, R. Hertle, J. X. Ma, T. M. Redmond, X. Zhu, B. Hauck, O. Zelenaia, K. S. Shindler, M. G. Maguire, J. F. Wright, N. J. Volpe, J. W. McDonnell, A. Auricchio, K. A. High and J. Bennett (2008). Safety and efficacy of gene transfer for Leber's congenital amaurosis. *N Engl J Med*, 358: 2240-2248

Manno, C. S., G. F. Pierce, V. R. Arruda, B. Glader, M. Ragni, J. J. Rasko, M. C. Ozelo, K. Hoots, P. Blatt, B. Konkle, M. Dake, R. Kaye, M. Razavi, A. Zajko, J. Zehnder, P. K. Rustagi, H. Nakai, A. Chew, D. Leonard, J. F. Wright, R. R. Lessard, J. M. Sommer, M. Tigges, D. Sabatino, A. Luk, H. Jiang, F. Mingozzi, L. Couto, H. C. Ertl, K. A. High and M. A. Kay (2006). Successful transduction of liver in hemophilia by AAV-Factor IX and limitations imposed by the host immune response. *Nat Med*, 12: 342-347

Marangoni, D., Z. Wu, H. E. Wiley, C. J. Zeiss, C. Vijayasarathy, Y. Zeng, S. Hiriyanna, R. A. Bush, L. L. Wei, P. Colosi and P. A. Sieving (2014). Preclinical Safety Evaluation of a Recombinant AAV8 Vector for X-linked Retinoschisis after Intravitreal Administration in Rabbits. *Hum Gene Ther Clin Dev*:

McClements, M. E. and R. E. MacLaren (2013). Gene therapy for retinal disease. *Transl Res*, 161: 241-254

Menghini M, Lujan BJ, Zayit-Soudry S, Syed R, Porco TC, Bayabo K, Carroll J, Roorda A, Duncan JL (2015) Correlation of outer nuclear layer thickness with cone density values in patients with retinitis pigmentosa and healthy subjects. *Invest Ophthalmol Vis Sci* 56: 372-381 DOI 10.1167/iovs.14-15521

Michalakis, S., R. L. Muehlfriedel, N. Tanimoto, V. Krishnamoorthy, S. Koch, S. C. Beck, H. Buening, T. Gollisch, M. Biel and M. W. Seeliger (2011). Exploring Different Serotypes And Promoters In rAAV-mediated Gene Replacement Therapy Of Achromatopsia Type 2 (ACHM2). *ARVO Meeting Abstracts*, 52: 490

Michalakis, S., R. Muhlfriedel, N. Tanimoto, V. Krishnamoorthy, S. Koch, M. D. Fischer, E. Becirovic, L. Bai, G. Huber, S. C. Beck, E. Fahl, H. Buning, F. Paquet-Durand, X. Zong, T. Gollisch, M. Biel and M. W. Seeliger (2010). Restoration of cone vision in the CNGA3-/- mouse model of congenital complete lack of cone photoreceptor function. *Mol Ther*, 18: 2057-2063

Michalakis, S., R. Muhlfriedel, N. Tanimoto, V. Krishnamoorthy, S. Koch, M. D. Fischer, E. Becirovic, L. Bai, G. Huber, S. C. Beck, E. Fahl, H. Buning, J. Schmidt, X. Zong, T. Gollisch, M. Biel and M. W. Seeliger (2012). Gene therapy restores missing cone-mediated vision in the CNGA3-/- mouse model of achromatopsia. *Adv Exp Med Biol*, 723: 183-189

Michelfelder, S., K. Varadi, C. Raupp, A. Hunger, J. Korbelin, C. Pahrmann, S. Schrepfer, O. J. Muller, J. A. Kleinschmidt and M. Trepel (2011). Peptide ligands incorporated into the threefold spike capsid domain to re-direct gene transduction of AAV8 and AAV9 in vivo. *PLoS One*, 6: e23101

Mussolino, C., M. della Corte, S. Rossi, F. Viola, U. Di Vicino, E. Marrocco, S. Neglia, M. Doria, F. Testa, R. Giovannoni, M. Crasta, M. Giunti, E. Villani, M. Lavitrano, M. L. Bacci, R. Ratiglia, F. Simonelli, A. Auricchio and E. M. Surace (2011). AAV-mediated photoreceptor transduction of the pig cone-enriched retina. *Gene Ther*, 18: 637-645

Nathwani, A. C., E. G. Tuddenham, S. Rangarajan, C. Rosales, J. McIntosh, D. C. Linch, P. Chowdary, A. Riddell, A. J. Pie, C. Harrington, J. O'Beirne, K. Smith, J. Pasi, B. Glader, P. Rustagi, C. Y. Ng, M. A. Kay, J. Zhou, Y. Spence, C. L. Morton, J. Allay, J. Coleman, S. Sleep, J. M. Cunningham, D. Srivastava, E. Basner-Tschakarjan, F. Mingozzi, K. A. High, J. T. Gray, U. M. Reiss, A. W. Nienhuis and A. M. Davidoff (2011). Adenovirus-associated virus vector-mediated gene transfer in hemophilia B. *N Engl J Med*, 365: 2357-2365

Pang, J. J., X. Dai, S. E. Boye, I. Barone, S. L. Boye, S. Mao, D. Everhart, A. Dinculescu, L. Liu, Y. Umino, B. Lei, B. Chang, R. Barlow, E. Strettoi and W. W. Hauswirth (2011). Long-term retinal function and structure rescue using capsid mutant AAV8 vector in the rd10 mouse, a model of recessive retinitis pigmentosa. *Mol Ther*, 19: 234-242

Park, T. K., Z. Wu, S. Kjellstrom, Y. Zeng, R. A. Bush, P. A. Sieving and P. Colosi (2009). Intravitreal delivery of AAV8 retinoschisin results in cell type-specific gene expression and retinal rescue in the Rs1-KO mouse. *Gene Ther*, 16: 916-926

Penaud-Budloo, M., C. Le Guiner, A. Nowrouzi, A. Toromanoff, Y. Cherel, P. Chenuaud, M. Schmidt, C. von Kalle, F. Rolling, P. Moullier and R. O. Snyder (2008). Adeno-associated virus vector genomes persist as episomal chromatin in primate muscle. *J Virol*, 82: 7875-7885

Peng, K. W., L. Pham, H. Ye, R. Zufferey, D. Trono, F. L. Cosset and S. J. Russell (2001). Organ distribution of gene expression after intravenous infusion of targeted and untargeted lentiviral vectors. *Gene Ther*, 8: 1456-1463

Peters, A. H., J. Drumm, C. Ferrell, D. A. Roth, D. M. Roth, M. McCaman, P. L. Novak, J. Friedman, R. Engler and R. E. Braun (2001). Absence of germline infection in male mice following intraventricular injection of adenovirus. *Mol Ther*, 4: 603-613

Pokorny, J., V. C. Smith, A. J. Pinckers and M. Cozijnsen (1982). Classification of complete and incomplete autosomal recessive achromatopsia. *Graefes Archives for Clinical and Experimental Ophthalmology*, 219: 121-130

Prokofyeva, E., R. Wilke, G. Lotz, E. Troeger, T. Strasser and E. Zrenner (2009). An epidemiological approach for the estimation of disease onset in Central Europe in central and peripheral monogenic retinal dystrophies. *Graefes Arch Clin Exp Ophthalmol*, 247: 885-894

Puech, B., B. Kostrubiec, J. C. Hache and P. François (1961). Epidemiology and prevalence of hereditary retinal dystrophies in the Northern France. *J Fr Ophtalmol* 14: 153-164

Roosing, S., A. A. Thiadens, C. B. Hoyng, C. C. Klaver, A. I. den Hollander and F. P. Cremers (2014). Causes and consequences of inherited cone disorders. *Prog Retin Eye Res*, 42: 1-26

Samulski, R. J., L. S. Chang and T. Shenk (1987). A recombinant plasmid from which an infectious adeno-associated virus genome can be excised in vitro and its use to study viral replication. *J Virol*, 61: 3096-3101

Schmitz-Valckenberg, S., F. G. Holz, A. C. Bird and R. F. Spaide (2008). Fundus autofluorescence imaging: review and perspectives. *Retina*, 28: 385-409

Schnepp, B. C., K. R. Clark, D. L. Klemanski, C. A. Pacak and P. R. Johnson (2003). Genetic fate of recombinant adeno-associated virus vector genomes in muscle. *J Virol*, 77: 3495-3504

Schuettrumpf, J., J. H. Liu, L. B. Couto, K. Addya, D. G. Leonard, Z. Zhen, J. Sommer and V. R. Arruda (2006). Inadvertent germline transmission of AAV2 vector: findings in a rabbit model correlate with those in a human clinical trial. *Mol Ther*, 13: 1064-1073

Simonelli, F., A. M. Maguire, F. Testa, E. A. Pierce, F. Mingozzi, J. L. Bennicelli, S. Rossi, K. Marshall, S. Banfi, E. M. Surace, J. Sun, T. M. Redmond, X. Zhu, K. S. Shindler, G. S. Ying, C. Ziviello, C. Acerra, J. F. Wright, J. W. McDonnell, K. A. High, J. Bennett and A. Auricchio (2010). Gene therapy for Leber's congenital amaurosis is safe and effective through 1.5 years after vector administration. *Mol Ther*, 18: 643-650

Smith, A. J., J. W. Bainbridge and R. R. Ali (2012). Gene supplementation therapy for recessive forms of inherited retinal dystrophies. *Gene Ther*, 19: 154-161

Streilein, J. W. (2003). Ocular immune privilege: therapeutic opportunities from an experiment of nature. *Nat Rev Immunol*, 3: 879-889

Sundaram, V., C. Wilde, J. Aboshiha, J. Cowing, C. Han, C. S. Langlo, R. Chana, A. E. Davidson, P. I. Sergouniotis, J. W. Bainbridge, R. R. Ali, A. Dubra, G. Rubin, A. R. Webster, A. T. Moore, M. Nardini, J. Carroll and M. Michaelides (2014). Retinal structure

and function in achromatopsia: implications for gene therapy. *Ophthalmology*, 121: 234-245

Sundin, O. H., J. M. Yang, Y. Li, D. Zhu, J. N. Hurd, T. N. Mitchell, E. D. Silva and I. H. Maumenee (2000). Genetic basis of total colourblindness among the Pingelapese islanders. *Nat Genet*, 25: 289-293

Tan, M. H., A. J. Smith, B. Pawlyk, X. Xu, X. Liu, J. B. Bainbridge, M. Basche, J. McIntosh, H. V. Tran, A. Nathwani, T. Li and R. R. Ali (2009). Gene therapy for retinitis pigmentosa and Leber congenital amaurosis caused by defects in AIPL1: effective rescue of mouse models of partial and complete Aipl1 deficiency using AAV2/2 and AAV2/8 vectors. *Hum Mol Genet*, 18: 2099-2114

Testa, F., A. M. Maguire, S. Rossi, E. A. Pierce, P. Melillo, K. Marshall, S. Banfi, E. M. Surace, J. Sun, C. Acerra, J. F. Wright, J. Wellman, K. A. High, A. Auricchio, J. Bennett and F. Simonelli (2013). Three-Year Follow-up after Unilateral Subretinal Delivery of Adeno-Associated Virus in Patients with Leber Congenital Amaurosis Type 2. *Ophthalmology*, 120: 1283-1291

Thiadens, A. A., A. I. den Hollander, S. Roosing, S. B. Nabuurs, R. C. Zekveld-Vroon, R. W. Collin, E. De Baere, R. K. Koenekoop, M. J. van Schooneveld, T. M. Strom, J. J. van Lith-Verhoeven, A. J. Lotery, N. van Moll-Ramirez, B. P. Leroy, L. I. van den Born, C. B. Hoyng, F. P. Cremers and C. C. Klaver (2009). Homozygosity mapping reveals PDE6C mutations in patients with early-onset cone photoreceptor disorders. *Am J Hum Genet*, 85: 240-247

Thiadens, A. A., V. Somervuo, L. I. van den Born, S. Roosing, M. J. van Schooneveld, R. W. Kuijpers, N. van Moll-Ramirez, F. P. Cremers, C. B. Hoyng and C. C. Klaver (2010). Progressive loss of cones in achromatopsia: an imaging study using spectral-domain optical coherence tomography. *Invest Ophthalmol Vis Sci*, 51: 5952-5957

Vandenberghe, L. H., P. Bell, A. M. Maguire, C. N. Cearley, R. Xiao, R. Calcedo, L. Wang, M. J. Castle, A. C. Maguire, R. Grant, J. H. Wolfe, J. M. Wilson and J. Bennett (2011). Dosage thresholds for AAV2 and AAV8 photoreceptor gene therapy in monkey. *Sci Transl Med*, 3: 131-139

Wilkinson, J. T., A. B. Richards, D. Choi, J. E. Robertson, Jr. and C. J. Flaxel (2013). Incidence of retinal detachment after fellow-performed primary pars plana vitrectomy. *ISRN Ophthalmol*, 2013: 353209

Willett, K. and J. Bennett (2013). Immunology of AAV-Mediated Gene Transfer in the Eye. *Front Immunol*, 4: 261

Wissinger, B., D. Gamer, H. Jagle, R. Giorda, T. Marx, S. Mayer, S. Tippmann, M. Broghammer, B. Jurklies, T. Rosenberg, S. G. Jacobson, E. C. Sener, S. Tatlipinar, C. B. Hoyng, C. Castellan, P. Bitoun, S. Andreasson, G. Rudolph, U. Kellner, B. Lorenz, G. Wolff, C. Verellen-Dumoulin, M. Schwartz, F. P. Cremers, E. Apfelstedt-Sylla, E. Zrenner, R. Salati, L. T. Sharpe and S. Kohl (2001). CNGA3 mutations in hereditary cone photoreceptor disorders. *Am J Hum Genet*, 69: 722-737

Wissinger, B., F. Muller, I. Weyand, S. Schuffenhauer, S. Thanos, U. B. Kaupp and E. Zrenner (1997). Cloning, chromosomal localization and functional expression of the gene encoding the alpha-subunit of the cGMP-gated channel in human cone photoreceptors. *Eur J Neurosci*, 9: 2512-2521

Wykoff, C. C., M. B. Parrott, H. W. Flynn, Jr., W. Shi, D. Miller and E. C. Alfonso (2010). Nosocomial acute-onset postoperative endophthalmitis at a university teaching hospital (2002-2009). *Am J Ophthalmol*, 150: 392-398 e392

Yang, P., K. V. Michaels, R. J. Courtney, Y. Wen, D. A. Greninger, L. Reznick, D. J. Karr, L. B. Wilson, R. G. Weleber and M. E. Pennesi (2014). Retinal Morphology of Patients With Achromatopsia During Early Childhood: Implications for Gene Therapy. *JAMA Ophthalmol*:

Zanta-Boussif, M. A., S. Charrier, A. Brice-Ouzet, S. Martin, P. Opolon, A. J. Thrasher, T. J. Hope and A. Galy (2009). Validation of a mutated PRE sequence allowing high and

sustained transgene expression while abrogating WHV-X protein synthesis: application to the gene therapy of WAS. *Gene Ther*, 16: 605-619

16. Appendices

Appendix 1

Rationale for vector & dosing

1. Vector

1.1 Safety

Three landmark clinical trials (NCT00516477, NCT00481546 and NCT00643747) have first shown safety and evidence of efficacy using recombinant adeno-associated viral (rAAV) vectored gene therapy in the eye (Bainbridge et al., 2008; Hauswirth et al., 2008; Maguire et al., 2008; Jacobson et al., 2012). In these three studies, a different form of retinal degeneration caused by mutations in the gene encoding RPE65 was treated by subretinal injections of rAAV2/2 vector solutions without a single serious adverse event (SAE) being reported A subset of patients even demonstrated some degree of improved visual function in the untreated eyes.

A phase 1 trial with 12 LCA patients confirms the safety and efficacy of the method, also after re-administration to the second eye (Maguire et al., 2009; Simonelli et al., 2010; Bennett et al., 2012). A follow up in 5 patients after 3 years additionally shows positive results (Testa et al., 2013).

A fourth trial (NCT01461213) is currently ongoing aiming to transduce retinal pigment epithelial and photoreceptor cells by subretinal delivery of rAAV2/2 vector solution in patients with choroideremia, caused by mutations in REP1. Again, no SAE has been reported and accumulating evidence suggests a dose dependent therapeutic effect in the treated vs. the untreated eye (Barnard et al., 2014; Huckfeldt and Bennett, 2014; Maclaren et al., 2014).

More detailed information is available from preclinical studies. The use of rAAV2/8 has been shown to be without safety concerns in the mouse to treat RP and choroideremia (Pang et al., 2011; Black et al., 2014), in the mouse and rabbit to treat X-linked retinoschisis (Park et al., 2009; Marangoni et al., 2014). Vandenberghe et al. investigated effects of subretinal delivery of rAAV2/8 expressing eGFP under the control of the ubiquitous CMV promoter in cynomolgus macaques (Vandenberghe et al., 2011). Four different dosing regimens (10e8, 10e9, 10e10, and 10e11 vgp)

were tested regarding local toxic effects (inflammation, degeneration), biodistribution of reporter gene, and systemic immune response. The following subheadings refer to this study, where not stated otherwise.

In the past, our research consortium of the RD-Cure project performed a study in the Cnga3 knockout mouse model of achromatopsia comparing the efficacy of serotype 5 (rAAV5.mCnga3) and serotype 8 (rAAV8.mCnga3) AAV vectors (Michalakis et al., 2011). In total more than 50 Cnga3 knockout mice were injected with either rAAV5.mCnga3 or rAAV8.mCnga3 and a success rate (e.g. percentage of treated mice with positive biological activity assay (BAA) and positive TEA) of 67.6% and 63.0%, respectively was found. In all mice with positive BAA, TEA was also positive. There was no significant difference in the success rate between AAV5 and AAV8 serotyped vectors. However, the onset time for a positive BAA and TEA signal was faster with AAV8 vectors (4 weeks post injection for AAV8 versus 8 weeks post injection for AAV5). This finding and the data of other groups (Surace and Auricchio, 2008; Vandenberghe et al., 2011) prompted to continue with serotype 8.

1.1.1 Immune response

Pre-clinical and clinical investigations have assessed the humoral and cellular immune responses to AAV administration to be safe and effective over a time period many years (see e. g. Willett and Bennett, 2013).

With respect to the **humoral immune response**, neutralizing antibodies (NABs) directed at the AAV capsid showed an upward trend with higher dosing in both intraocular fluid and serum. NABs are antibodies directed against a specific epitope (such as AAV capsid proteins), which aim to neutralize the biologic action of the antigen without triggering the cellular immune response. Interestingly, titres of NABs did not show an obvious correlation with total transgene expression (whole mount fluorescence), relative intensity and/or percentage of transduced cells within area exposed to virus particles. This is in line with the notion that the subretinal space is relatively protected from immunresponse and supported by earlier research (Streilein, 2003; Li et al., 2008). Li et al. demonstrated that pre-existing NABs against AAV do not have an impact on the transduction efficiency of subretinal AAV particles. This has independently been confirmed in other laboratories (Ali R., personal

communication).

In Vandenberghe's study, **T-cell response** directed against eGFP was detected in one out of twenty eyes (1 out of 5 in highest dose group). The same animal and one other animal (with no discernible systemic cellular/humoral immune response) from the highest dose group showed signs of retinal inflammation and thinning in the eye treated with the highest vector dose. It is currently unknown whether this local effect is primarily due to the high virus load, contaminants in the research grade vector solution (not GMP grade), or due to toxic effects of high eGFP expression levels. While the lead author suspects residual components as the main culprit (Vandenberghe, personal communication), other studies have given evidence for negative effects of eGFP on photoreceptor viability (Liu et al., 1999).

Ferreira et al. (Ferreira et al., 2014a; Ferreira et al., 2014b) report about the immune response from a phase II clinical trial leading to the market approval of the first gene therapy product (Glybera). Both cellular and humoral immune responses were evident against AAV1 capsid proteins despite continuous systemic immunosuppression with Cyclosporine A (3mg/kg/D) and Mycophenolat-Mofetil (2g/d) over 12 weeks and a single intravenous injection of Methylprednisolon (1mg/kg) just prior intramuscular administration of therapeutic AAV1 vector solution. Interestingly, they could not detect any immune response against the transgene (lipoprotein lipase), only against the capsid proteins. But even the humoral and cellular immune response did not lead to cytotoxicity through mechanisms not fully understood. The authors speculate that this tolerance might be caused by known immunological mechanisms of ignorance, anergy or clonal deletion

1.1.2 Biodistribution

The biodistribution of AAV particles injected into the subretinal space is very limited. Several independent clinical trials using AAV for subretinal gene delivery have excluded AAV dissemination to peripheral blood (Hauswirth et al., 2008; Maguire et al., 2008; Jacobson et al., 2012) .Only in one case in one study, was AAV genome detected in a tear sample on day 1 after surgery, with none detected at later time points (Maguire et al., 2008).

Other clinical trials using essentially the same vector as proposed in this study

(rAAV2/8 (Nathwani et al., 2011) administered ca. 1000 fold higher numbers of virus (2×10e12vgp/kg) via peripheral-vein infusion directly into the systemic circulation of hemophilia patients. Interestingly, vector genome was detectable in the plasma, saliva, semen and stools within 72 hours of vector infusion and up to but not after day 15 in all participants, with the exception of participant 1 whose semen remained clear of proviral DNA at all-time points assessed. Vector sequences were not detected in the urine of any of the participants at any time point after administration of vector in this study.

In the preclinical setting, Vandenberghe et al. (2011) used qPCR to detect reporter gene (eGFP) biodistribution in non-human primates after subretinal delivery of AAV8 and showed dose dependent expression of eGFP mainly in retinal pigment epithelium (RPE) cells and photoreceptors (PRs). At a titre of 10e9 AAV8 transduced ca. 25% of PRs in the exposed area. At 10e10 already ca. 70±20% of PRs and RPE cells were positive for the reporter transgene. AAV8 showed ca. one log unit higher transduction efficiency when compared to AAV2. Given the ubiquitous activity of the CMV promoter, there was also extraocular eGFP expression found e.g. in the optic nerve (in 1/5 eyes at 10e10vgp; in 5/5 eyes at 10e11vgp) and in the optic chiasm (in 5/5 eyes at 10e11vgp). The authors described a correlation between the dosage (vgp) and extent of extraocular eGFP expression with animals in the high dose group having the most extensive extraocular involvement. However, the pattern of eGFP distribution could not fully be explained, as it did not match the pathway of retrograde transport in the visual pathway. E.g. the highest eGFP signal came from layers within the lateral geniculate nucleus (LGN) known to be dominated by the magnocellular system, which is associated with retinal ganglion cells (RGCs) from more peripheral retinal areas. These are also known to travel within the more peripheral parts of the optic nerve (ON), which might make them more accessible for primary transduction e.g. at the optic nerve head, where those axonal fibers are in direct contact with the subretinal space. On the other hand, AAV particles could have traversed the entire retina to reach the RGCs directly. However, this appears unlikely as no intermediate cells (e.g. bipolar cells) were eGFP positive even though the promoter would be active ubiquitously.

Taken together, it is not entirely sure, whether the extraocular transgene expression is really due to retrograde transfection at higher dose ranges, or whether subretinal delivery of AAV potentially exposes peripheral axonal fibers to transduction. This can be elegantly addressed by using a promoter with target cell specific activity in the therapeutic vector such as used in this study.

1.2 Efficiency

The vector of choice, rAAV2/8, has been studied in detail regarding its efficiency to transduce photoreceptor cells – the target cells for our trial. Vandenberghe et al. (2011) injected rAAV2/8 expressing eGFP under the control of the CMV promoter and a woodchuck hepatitis virus post-transcriptional regulatory element into the subretinal space of cynomolgus macaques. The vector efficiently transduced RPE and photoreceptor cells across a dose range (10e8, 10e9, 10e10, and 10e11vgp).

2. Dosing

In the proposed clinical trial, patients will have one of three dosages injected into a preformed subretinal space with a maximal total volume of 1ml of balanced salt solution (BSS):

- Low dose: ≤ $1x10^{10}$ vector genome particles (vgp)
- Intermediate dose: ≤ $5x10^{10}$ vgp
- High dose: ≤ $1x10^{11}$ vgp

The two important parameters are the number of vector genome containing particles (vgp) and the number of target cells, which are brought in physical contact with the vector solution. Previous studies targeting the RPE have applied $1x10^{10}$ to $1.5x10^{11}$ rAAV2/2 vgp into the subretinal space. The only published trial using the same vector design of the proposed study (rAAV2/8) used significant higher doses ($1\text{-}2x10^{14}$ vgp) to target hepatocytes, and were injected into systemic circulation rather than the confined subretinal space in the eye. Yet, no SARs were recorded in this trial.

Vandenberghe et al. showed local toxic effects (degeneration and inflammation) in non-human primates after subretinal injection of $1x10^{11}$ vgp AAV2/8. However, these AAV particles were not manufactured to GMP or even GLP guidelines and used undiluted in this cohort. Also, cytotoxic GFP was used as reporter-transgene under the control of a highly active CMV promoter likely contributing to the toxic effects observed.

Our toxicology study has shown no adverse reaction at the level of the highest dose planned in the clinical trial (≤ $1x10^{11}$ vgp) and only transient reactions were observed in 2/4 animals with a ten-fold higher dose ($1x10^{12}$ vgp) with no residual changes were observed 90d post dosing. We therefore conclude that it seems safe to apply a maximal dose of ≤ $1x10^{11}$ vgp in our patients. This dose was therefore chosen as the maximal dose.

This dosing range was chosen based on pre-clinical evidence in the CNGA3-ko mouse model. Michalakis et al. showed successful rescue by injecting $5x10^{9}$ rAAV2/5 into the subretinal space detaching ca. 1/3 of the retina [1]. This was again confirmed with the IMP vector (engineering lot) using a dose as low as $5x10^{9}$ vgp in a relevant mouse model (see Study report).

Detaching 1/3 of the mouse retina brings $5x10^{6}$ cells (rod & cone photoreceptors and retinal pigment epithelium) in direct contact with the vector solution resulting in a theoretical multiplicity of infection (MOI) of $1x10^{3}$ [2, 3]. As the proposed study foresees to raise a bleb of ca. 2mm radius, $1x10^{9}$ cells would be in direct contact with the vector solution [3, 4]. To achieve an MOI of $1x10^{3}$ an equivalent dose $1x10^{12}$ vgp would be needed.

As this is a first in man study, we felt it was prudent to start with a lower dose to test safety before stepwise increasing the absolute vg number to the equivalent dose from the pre-clinical trials with proven efficacy.

Summary of changes

Clinical trial protocol

Number	Protocol Version	Date	Summary of changes
1	1.1	27.02.2015	Roles in the study were adapted due to changes in academic titles (page 9)<br>Investigators Agreement (page 12)
2	2.0	14.08.2015	Inclusion/exclusion criteria were revised according to regulators suggestions (page 10/11)<br>Information on biodistribution methods (synopsis and section 7.2.1)<br>Correction regarding safety data in NHP trials (section 1.3.3)<br>Time schedule (synopsis and study plan, sections 3.1, 3.2 and Fig. 3<br>Flowchart of visits (clarification and addition)<br>Rationale for vector serotype decision (rationale for study section 1.2; Appendix)
3	3.0	31.08.2015	Study duration (Section 3.2, Page 28)<br>Risk-Benefit Considerations (Section 7.5, page 38/39)<br>Risks for the Environment (Section 7.6, page 40)
4	4.0	14.12.2015	Section VII (Flowchart) and 8.4.16 Farnsworth D15 was added<br>Section VII (Flowchart) HIV Test was added<br>Section 8.1: Screening examinations were adapted to the flowchart<br>Section IV, VII, 7.2.1: ELISPOT was deleted
5	5.0	16.02.2017	Section 10.5. An interim analysis was added, and the reason explained.

Statistical analysis plan

There were no changes in the statistical analysis plan except an interim analysis was included in version 5.0 (see above).
