# Supplementary material for: Safety and Vision Outcomes of Subretinal Gene Therapy Targeting Cone Photoreceptors in Achromatopsia: A Nonrandomized Controlled Trial
Source: JAMA Ophthalmol. 2020 Apr 30;138(6):1–9. doi: 10.1001/jamaophthalmol.2020.1032 (PMC7193523; doi:10.1001/jamaophthalmol.2020.1032)
Supplement: Supplement 2. — eAppendix. Supplementary Methods and Results eTable 1. Flowchart of Visits and Procedures eTable 2. Classification of Reported Adverse Events eTable 3. Findings in Blood Tests eTable 4. Reporting of Vector Genome Copies Detected Over Time eTable 5. Titers of anti-AAV8 Antibodies Over Time (ELISA) eTable 6. Reaction Profile of Immune Cells Against AAV8 Epitopes and Recall Antigens eTable 7. Significance Analyses in Functional Outcomes of the Control Eye eTable 8. Ophthalmological Outcomes - Tests of Significance eTable 9. Patient Reported Outcomes: Study Specific Scale A3 (PRO) Results eTable 10. Patient Reported Outcomes: VFQ-25 Results eTable 11. Patient Reported Outcomes: BSI Results eFigure 1. Color Photographs, Infrared Images and Virtual Cross Sections of Treated Eyes eFigure 2. Treatment Area and Effect on Foveal Anatomy of Treated Eyes eFigure 3. Visual Acuity, Contrast Sensitivity, Color Contrast Sensitivity, and Temporal Resolution of the Treated Eyes. eFigure 4. Visual Acuity, Contrast Sensitivity, Color Contrast Sensitivity, and Temporal Resolution of the Untreated Control Eyes. eFigure 5. Visual Acuity and Contrast Sensitivity Comparison Between the Treated Eyes and Untreated Control Eyes eFigure 6. Z Score Normalization of Efficacy Endpoints [file jamaophthalmol-138-643-s002.pdf]

Supplementary Online Content

Fischer MD, Michalakis S, Wilhelm B, et al. Safety and vision outcomes of subretinal gene therapy targeting cone photoreceptors in achromatopsia. *JAMA Ophthalmol.* Published online April 30, 2020. doi:10.1001/jamaophthalmol.2020.1032

eAppendix. Supplementary Methods and Results

eTable 1. Flowchart of Visits and Procedures

eTable 2. Classification of Reported Adverse Events

eTable 3. Findings in Blood Tests

eTable 4. Reporting of Vector Genome Copies Detected Over Time

eTable 5. Titers of anti-AAV8 Antibodies Over Time (ELISA)

eTable 6. Reaction Profile of Immune Cells Against AAV8 Epitopes and Recall Antigens

eTable 7. Significance Analyses in Functional Outcomes of the Control Eye

eTable 8. Ophthalmological Outcomes - Tests of Significance

eTable 9. Patient Reported Outcomes: Study Specific Scale A3 (PRO) Results

eTable 10. Patient Reported Outcomes: VFQ-25 Results

eTable 11. Patient Reported Outcomes: BSI Results

eFigure 1. Color Photographs, Infrared Images and Virtual Cross Sections of Treated Eyes

eFigure 2. Treatment Area and Effect on Foveal Anatomy of Treated Eyes

eFigure 3. Visual Acuity, Contrast Sensitivity, Color Contrast Sensitivity, and Temporal Resolution of the Treated Eyes.

eFigure 4. Visual Acuity, Contrast Sensitivity, Color Contrast Sensitivity, and Temporal Resolution of the Untreated Control Eyes.

eFigure 5. Visual Acuity and Contrast Sensitivity Comparison Between the Treated Eyes and Untreated Control Eyes

eFigure 6. Z Score Normalization of Efficacy Endpoints

This supplementary material has been provided by the authors to give readers additional information about their work.

© 2020 Fischer MD et al. *JAMA Ophthalmology.*

eAppendix. Supplementary Methods and Results

1. Patients and Methods

The study was designed and conducted by M. D. Fischer, S. Michalakis, B. Wilhelm, D. Zobor, S. Kohl, A. Werner, S. Tsang, T. Peters, K. U. Bartz-Schmidt, E. Zrenner M. Biel, and B. Wissinger. Data were gathered and analyzed by M. D. Fischer, P. Martus, and the remaining authors. The members of the RD-CURE decided to publish the paper and vouch for the data and the analysis. M. D. Fischer wrote the paper. S. Michalakis, B. Wilhelm, S. Kohl, V. Sothilingam, D. Dauletbekov, F. Paquet-Durand, S. Tsang, T. Peters, M. Ueffing, E. Zrenner, M. Biel, P. Martus, and B. Wissinger reviewed the paper. All authors read and approved the manuscript.

This was a first-in-man, dose escalation clinical trial registered with ClinicalTrials.gov (NCT02610582: 'Safety and Efficacy of a Single Subretinal Injection of rAAV.hCNGA3 in Patients With CNGA3-linked Achromatopsia'). It was conducted in accordance with the tenets set forth in the Declaration of Helsinki. All authors vouch for the fidelity of the study conduct to the protocol and accuracy and completeness of the data, analysis and reporting of adverse events. An independent data and safety committee monitored the integrity and safety of the study. All clinical data were reviewed by in independent data monitor and clinical data were analyzed through an eCRF database independently by the Institute for Clinical Epidemiology and Applied Biostatistics, University Hospital Tuebingen. A more in-depth report on the clinical trial protocol is currently under review (N. Kahle et al. *Human Gene Therapy Clinical Development*).

1.1 Patient Eligibility Criteria

Adult (>18 years and older) patients of either sex were eligible for the study if they met all of the following inclusion criteria and none of the exclusion criteria set out below:

Inclusion Criteria:

- clinical diagnosis of achromatopsia
- ≥ 18 years of age
- confirmed mutation in *CNGA3*
- BCVA ≥ 20/400
- a minimal outer nuclear layer thickness of 10µm at 3° eccentricity in the study eye (normal = 38±6µm)
- ability to understand and willingness to consent to study protocol
- no infection with Human Immunodeficiency Virus (HIV)

- negative pregnancy test in women with childbearing potential (a woman who is two years post-menopausal or surgically sterile is not considered to be of childbearing potential)

Exclusion Criteria:

- additional interfering eye conditions (e.g. uveitis, advanced cataract) in the study eye
- systemic conditions (e.g. coronary heart disease, autoimmune disorders) which may affect study participation or outcome measures
- current or recent participation in other study/or administration of biologic agent within the last three months
- recent (6 months) ocular surgery, intravitreal or subretinal implantation of a medical device
- known sensitivity to any compound used in the study
- contraindications to systemic immunosuppression
- subject/partner of childbearing potential unwilling to use adequate contraception for four months
- nursing or pregnant women
- any other cause that, in the investigator's opinion, renders potential subjects not suitable for the study
- mutations in another achromatopsia gene
- contraindications in view of the planned surgery (e.g. anaemia Hb<8g/dl, severe coagulopathy, severe blood pressure fluctuations)
- ocular opacity and mature cataract
- history of ocular malignancies
- disorders of the internal retina (e.g. retinal detachment in the patients history)
- glaucoma defined as damage of the optic nerve
- vascular retinal occlusion
- diabetic patients suffering from retinopathy and/or macula edema
- patients treated with oral corticoids within 14 days prior inclusion
- systemic illness or medically significant abnormal laboratory values in blood analysis including renal and hepatic functions at inclusion
- absence of vision on the other contralateral eye

1.2 Primary Outcome Measure

Safety (adverse events): Number of participants with abnormal laboratory values and/or adverse events that are related to treatment within one year. Safety as the primary endpoint was assessed by clinical examination of ocular inflammation. Systemic safety was assessed

© 2020 Fischer MD et al. *JAMA Ophthalmology.*

by vital signs, routine clinical chemistry testing and full/differential blood counts. Immunopathology assays included specific enzyme-linked immunosorbent assays for humoral antibodies against AAV8 capsid protein and lymphocyte transformation tests, flow cytometry and supernatant cytokine assays (LTT-SCA) to monitor cellular immune reactivity against AAV8 capsid protein as well as recall antigens to analyze the effect on general immune reactions[1,2] (also see section 8 for methodological details). Shedding and biodistribution was monitored by polymerase chain reaction for vector genome in blood, urine, saliva and tears.

1.3 Secondary Outcome Measures

Efficacy measures: Number of participants with improved visual function (best corrected visual acuity, contrast sensitivity, color vision, temporal resolution, retinal sensitivity, and pupillography) within one year. Patient reported outcomes were investigated exploratively (see **eTable 1**). A more in-depth report on the methods applied as per clinical trial protocol is currently under review (N. Kahle et al. *Human Gene Therapy Clinical Development*).

2. Reported adverse events and safety summary

From the beginning of the study in November 2015, 56 adverse events in 9 patients were reported. Seventeen adverse events were non-ocular, 39 were ocular. The two unresolved ocular adverse events were mild pigmentary changes. No SAEs occurred during the study. The vast majority of the non-ocular and ocular AEs were related to the surgical procedure and not to the adenovirus-associated vector. The only AEs, which may be related to the vector are a transient occurrence of hyper-reflective spots (in virtual cross sections of treated retina by optical coherence tomography) in patient 102 (low dose) and one self-limiting episode of iridocyclitis in patient 106 (intermediate dose). There were no ocular AEs outside the expected side effect profile of the study procedure and study drug.

2.1 List of reported non-ocular adverse events

Common cold	8
Headache	1
Sinusitis	1
Urinary tract infection	2
Twisted (right) ankle	1
Temporary leukocytosis	1
Temporary granulocytosis	1
Atopic dermatitis	1
Heartburn	1

© 2020 Fischer MD et al. *JAMA Ophthalmology.*

2.2 List of reported ocular adverse events

Neurosensory retinal detachment in target area	10
Conjunctival injection/redness	7
Chemosis of the conjunctiva	7
Pigmentary changes	5
Foreign body sensation	4
Corneal erosion	2
Iridocyclitis	1
Tyndall	1
Hyper-reflective spots in OCT of central macula	1
Minor retinal hemorrhage	1

3. Vital signs and blood test data

Vital signs included body mass index (BMI), systolic and diastolic blood pressure, heart rate and temperature. Except for BMI, all measurements were obtained at each visit. Data from two patients were missing at visit 5 (day 3). BMI was measured only at visit 1 and visit 10. Neither systolic nor diastolic blood pressure demonstrated a significant change over time (max. variation over 1 year: 12mmHg). Likewise, heart rate (variation < 10 min-1) and body temperature (variation < 1°) and BMI remained essentially stable within the observation period of one year.

An overview of blood test results is presented in eTable 3. Only two out of 1,296 hematological test results (8 time points, 9 patients, 18 parameters) were deemed clinically significant (temporary leukocytosis & granulocytosis under steroid treatment) but neither required change in clinical management and/or any form of treatment (see also 3.1. below). There was not a single clinically significant change in 1008 test results (8 time points, 9 patients, 14 parameters) concerning basic clinical chemistry and only one in 216 test results (8 time points, 9 patients, 3 parameters) on C-reactive protein, total IgM or IgG levels (elevated CRP associated with recurrent urinary tract infection).

3.1. Hematology: EDTA blood samples were used to count leukocytes (reference range [RR]: 3,800-10,300 μl^{-1} absolute), neutrophils (RR: 1,800-7,000 μl^{-1}, 40-80% of leukocytes), lymphocytes (RR: 1,100-3,200 μl^{-1}, 20-45% of leukocytes), monocytes (RR: 200-700 μl^{-1}, 2-8% of leukocytes), eosinophils (RR: 30-470 μl^{-1}, 1-4% of leukocytes), basophils (RR: 20-110 μl^{-1}, 0-2% of leukocytes), immature granulocytes (RR: 200-700 μl^{-1}, 0-0.6% of leukocytes), normoblasts (RR: 0-100 μl^{-1}, 0-1% of leukocytes), erythrocytes (RR: 4.2-6.2x10^6 μl^{-1}),

© 2020 Fischer MD et al. *JAMA Ophthalmology.*

hematocrit (RR: 42-52%), hemoglobin (RR: 14-18 g/dl), mean corpuscular volume (RR: 80-93 fl), mean corpuscular hemoglobin (RR: 27-34 pg), mean corpuscular hemoglobin concentration (RR: 32-36 g/dl), red cell distribution width (RR: 0-15%), platelet count (RR: 1.5-4.5x10^5 μl^{-1}), platelet distribution width (RR: 10-16 fl), and mean platelet volume (RR: 9-12 fl).

Of interest, seven out of nine patients showed a slight to moderate increase in total number of leukocytes with a peak at visit week 2 (mean = 12,931 μl^{-1}, range = 7,920 - 18,160 μl^{-1}). The maximal leukocytosis was 1.8 times the upper reference range limit (patient 102). The same patient also demonstrated a temporary granulocytosis (2 times the upper reference range limit) at the same time-point. Both parameters normalized by the next visit and none of the patients showed any related clinical symptoms at any time-point.

3.2. Basic clinical chemistry: Plasma samples collected in lithium heparin tubes were tested for electrolytes (sodium, potassium), creatinine, glomerular filtration rate (GFR), urea, direct and global bilirubin, creatinine kinase (CK), aspartate transaminase (AST) and alanine transaminase (ALT), alkalic phosphatase (AP), lactate dehydrogenase (LDH), gamma-glutamyl-transferase (GGT). No consistent and/or clinically significant changes were found. Some patients had pre-existing values outside the reference range.

3.3. Immune markers: C-reactive protein (CRP, reference range: 0-0.5 mg/dl) was tested in plasma samples collected in lithium heparin tubes and IgG and IgM values quantified from serum samples. No consistent and/or clinically significant changes were found. However, 2 out of 9 had elevated CRP values with a peak at month 1 (mean = 0.44 mg/dl, range = 0.01 – 1.95 mg/dl). The patient (103) with CRP of 1.95 mg/dl was diagnosed with urinary tract infection (UTI, see reported AEs) and treated accordingly.

3.4. Urine analysis: Color, clarity, specific weight, pH, protein and glucose content were analyzed. No consistent alteration in any of these could be seen during study course. Patient 103 had two episodes of UTIs during the course of the study, which was in line with her medical history or recurrent UTIs.

4. Virus shedding / biodistribution data

Samples of blood (5ml in sodium-citrate tubes), urine (>5ml in a sterile container), saliva and tears were collected before surgery, and at day 3, 14, 30 and 90 after surgery for quantitative qPCR analysis. Saliva and tears were collected with sterile rayon swabs, which were soaked with the respective fluid before placing it in 1ml sterile phosphate buffered saline (PBS) in a cryotube at room temperature. The stem of the rayon swab was cut and the cryotube vortexed.

© 2020 Fischer MD et al. *JAMA Ophthalmology.*

The swab stem was then pressed on the inner side of the tube and finally discarded. DNA was extracted from 200µl (blood and urine) or 400µl (saliva and tears) samples using the QIAamp DNA Mini Kit (Qiagen) according to the manufacturer's instructions and concentrated DNA samples (8µl) were used as template for qPCR analysis. To generate a standard curve, a fresh sample of the clinical vector AAV8.CNGA3 was incubated with DNAse for 1 hour and then heated to 95° for 10 min to inactivate the DNAse and denaturate the vector capsid to yield naked vector genomes (vg). This sample was then serially diluted to produce a standard range between $8x10^8$ to 10 vg/reaction. The limit of detection was defined according to the MIQE (minimum information for publication of quantitative real-time PCR experiments) guidelines[3,4]: The standard curve sample containing the lowest concentration of AAV8.CNGA3 genome, which was detected in 95% of reactions and with the standard deviation (in Ct values) of its replicates less than 1.3 was chosen as the limit of detection and was estimated to be at 800 vg/reaction (average Ct 35.99). The following pair of primers targeting the *CNGA3* sequence was used for the qPCR assay at a final concentration of 0.25µM:
forward 5'-GCTACAAAAACAGAGGACAAACAACA-3';
reverse 5'-AAAAGGAGCAACATAGTTAAGAATACCA-3'.
For each qPCR reaction, 10µl of SsoAdvanced™ Universal SYBR® Green Supermix (Bio-Rad) was mixed with the primers and 8µl of DNA eluate as a template. The CFX96 Real time system (Bio-Rad) was used for thermal cycling, and real-time fluorescence detection, utilizing 20µl reaction volumes. The 3-step qPCR protocol included a 2min enzyme activation step at 95°C, followed by 40 cycles of 10s at 95°C, 30s at 60°C and 30s at 72°C. **eTable 4** reports all results of vg numbers detected over time in blood, urine, saliva and tear samples over time. Not a single sample showed a positive result before or after treatment at any time-point tested.

5. Vector production

The AAV8.CNGA3 vector used in this study is a recombinant adeno-associated virus (AAV) vector expressing the full length (2085 bp) human cyclic nucleotide-gated cation channel alpha 3 gene (*CNGA3*) under the control of the 405 bp cone photoreceptor-specific human arrestin 3 (*ARR3*) promoter. The expression cassette is flanked by wild-type AAV serotype 2 inverted terminal repeats (ITRs)[5] and contains a 207 bp bovine growth hormone polyadenylation signal (BGHpA) and a 543 bp Woodchuck Hepatitis Virus Posttranscriptional Regulatory Element (WPRE) sequence with mutated WXF-open reading frame.[6]

GMP grade AAV *cis* (pSub-hArr3-hCNGA3-WPREm-KanR) and *trans* (pDP8-KanR)[7] plasmids were produced from fully characterized *E. Coli* Master Cell Bank (MCB) (DH10B strain) (Aldevron, Fargo, ND, United States of America). GMP-grade AAV8-pseudotyped viral particles (AAV8.CNGA3) were produced at Atlantic BioGMP (Nantes, France). GMP

© 2020 Fischer MD et al. *JAMA Ophthalmology.*

manufacturing of AAV8.CNGA3 involved calcium phosphate transfection of cis and trans plasmids in fully characterized HEK293 MCB. AAV particles were harvested from cells and supernatant and purified by two consecutive CsCl-gradient centrifugation steps followed by tangential flow filtration (TFF) for buffer exchange and concentration, and finally sterile filtration and filling and stored at -70°C until use. The production process and the final product conform to the European Pharmacopoeia. Vector genome (vg) titer assayed by quantitative PCR (qPCR), infectious titer (infectious center assay, ICA) were determined after each purification step. For the final vector, the infectious titer and vector particles titer was determined using TCID50 (ip/ml) and ELISA test (vp/ml), respectively. Quality control (IPQC) included assays for transgene expression, biological activity, genomic integrity, protein purity, residual cell and plasmid DNA, residual PEG, CsCl, benozonase, endotoxins, pH, osmolarity, aggregates and appearance. The final product was also tested for replication competent AAVs.

6. Biological activity assays / Transgene expression assay

The different GMP vector production batches were assessed for biological activity and transgene expression in *Cnga3*$^{-/-}$ mice according to previously published procedures[8]. Specifically, *Cnga3*$^{-/-}$ mice aged 2 weeks received a subretinal injection from each batch in the treated eye (TE), whereas the contralateral, untreated eye (UE) served as control. An ophthalmic examination using optical coherence tomography (OCT) was performed on each mouse at baseline (directly upon completion of the injection) to assure regular morphological conditions and exclude technical complications as an immediate post injection quality control. The vector efficacy for the batches was evaluated at 8 weeks and additional timepoints following the injection by means of electroretinography (ERG). *Cnga3*$^{-/-}$ mice lack any cone-mediated vision. Therefore, ERG protocols specifically testing for cone function are suitable as an indirect measure for CNGA3 function and for the assessment of biological activity (biological activity assay, BAA). After completion of the ERG measurements mice were euthanized, the eyes enucleated and processed for immunohistological analysis of transgene expression (transgene expression assay, TEA). For this, the tissue was fixed and cryoembedded. Vertical cryosections were stained with a rat monoclonal antibody (clone 7D8) directed against human CNGA3 protein[9]. The immunosignal was detected with a Cy3 tagged donkey anti-rat IgG secondary antibody (Jackson ImmunoResearch Europe). Confocal images from the immunostained cryosections were collected using a SP8 SMD confocal laser scanning microscope (Leica Microsystems). The 7D8 antibody also detects mouse Cnga3 protein and gives a specific signal in cone photoreceptor outer segments of wildtype mouse retina and no signal in *Cnga3*$^{-/-}$ retina. BAA and TEA confirmed biological activity and transgene expression for all tested AAV8.CNGA3 batches.

© 2020 Fischer MD et al. *JAMA Ophthalmology.*

7. Surgical procedure and concomitant pharmacological treatment

7.1. Subretinal surgery

Standard three-port 23G *pars plana* vitrectomy (removal of vitreous humor and posterior hyaloid membrane) with balanced salt solution was used to replace the vitreous and allow subsequent maneuvers: Balanced salt solution was used to induce a shallow, localized primary retinal detachment involving the cone rich fovea (marked ‘F’ in the **Illustration**) in an area of ca. 3mm diameter. The retinotomy (black dot) was placed just central of the superior temporal branch of the central retinal artery (see Fig. S1). Once the subretinal space had been pre-formed, 200µl vector solution was applied using a disposable 41G subretinal injection needle within a standard 23G body to fit the port system. Sclerotomies were sutured watertight at the end of surgery (7-0 vicryl sutures).

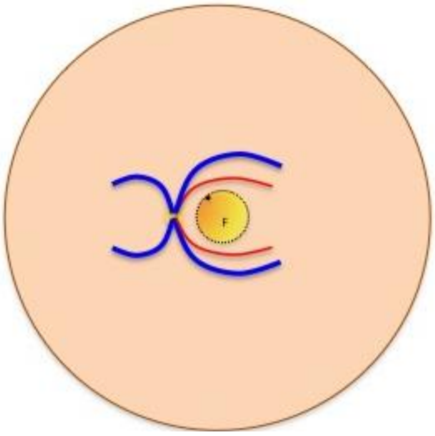

Illustration of subretinal injection in the central macula in a left eye. Venous (blue) and arterial (red) vessels originate in the optic disc and circumscribe the macula with foveal center (F). Black dot indicates a suitable place for retinotomy (penetration site of the needle through the retina) and the yellow area indicates the target area of the bleb filled with vector solution.

7.2. Standard concomitant medication

Any concomitant medication was documented during the trial. Pharmacological treatment followed the standard operating procedure of the hospital for vitrectomy: Moxifloxacin (5 mg/ml, Novartis Pharma) eyedrops *qid* and dexamethasone (0.1%, Bausch & Lomb) eyedrops *qid* for three weeks starting the day before surgery. In theater the eye is scrubbed with 10% and 1% povidone iodine solution, during surgery 2% Hydroxypropylmethylcellulose were applied as eye drops to maintain corneal clarity and 50mg Mezlocillin (Bayer) + 4mg dexamethasone (both in 1ml) were applied as subconjunctival injection. Mydriatic ointment (Atropin 2%, hospital pharmacy) combined with antibiotic/antiphlogistic ointment (Gentamicin 0.3% + 0.03% dexamethasone) was administered at the end of surgery to the operated eye with an overnight eye patch.

7.3. Study specific concomitant medication

Systemic steroids were given orally 1.0mg/kg for three weeks starting at day -1 and then tapered off after day 19 by 50% in a weekly interval until 5mg and then stopped (e.g. 80mg until day 21, 40mg until day 28, 20mg until day 35, 10mg until day 42 and 5mg for another week until day 49).

© 2020 Fischer MD et al. *JAMA Ophthalmology.*

8. Immunological assessment

8.1. Methods

ELISA

Anti-AAV8 antibodies were determined by ELISA according to a standard procedure using the Kit control AAV2/8 from the AAV8 titration ELISA obtained from Progen Biotechnik GmbH (Heidelberg, Germany) as antigen for coating 96 well plates. Patients' sera were diluted 1:500. Peroxidase conjugated anti-human IgG- ang IgM-antibodies (diluted 1:2,000 and 1:3,000, respectively) were used for visualization of bound antibodies. Positive and negative controls were applied. Normal values were calculated with sera from 10 healthy blood donors.

PBMC cultures

PBMC were isolated from 50ml heparinized blood drawn in the morning by centrifugation through Ficoll-Hypaque as described previously[1,2]. PBMC were adjusted to 1 million cells/ml in RPMI 1640 medium supplemented with gentamycin and 25% autologous serum, and incubated at 37°C in a humidified atmosphere containing 5% CO_2. Prior to the experiments kinetics had been performed to obtain the optimal time points for the assays. These resulted in incubation of PBMC with the different antigens for 24h for the determination of the early activation marker CD69 by flow cytometry, and 7 days for the analysis of the proliferation and release of cytokines into the supernatants[1,2].

Proliferation assay

Proliferation of PBMC was determined by thymidine-uptake according to standard procedures[1,2]. PBMC ($1x10^6$/well) were seeded into 96-well cell culture plates and incubated without (spontaneous proliferation; control), with the specific antigen AAV8.CNGA3, and with the recall antigens Bacillus Calmette-Guérin (BCG), PPD (purified protein derivative) and tetanus toxoid in different concentrations ranging from 5 to 0.005µg/ml. BCG activates predominantly macrophages/monocytes and T helper type 1 (TH1)-cells, PPD stimulates TH1-cells and tetanus toxoid TH2-cells. After pulsing with ^{3}H-thymidine (0.74MBq/ml, 20µl/well), incorporated radioactivity was measured by liquid scintillation spectroscopy using a β-counter, and given as counts per minute (cpm). All tests were performed in quadruplicates; mean values were calculated and used for statistical analysis.

Flow cytometry

The activation marker CD69 was determined on T-, B-, and natural killer (NK)-cells by flow cytometry using FastImmune™CD4/CD69/CD3,-CD8/CD69/CD3,-CD19/CD69/CD45, and -

© 2020 Fischer MD et al. *JAMA Ophthalmology.*

CD56/CD69/CD45 (Becton-Dickinson, San Jose, CA); an IgG-isotype antibody (BD Biosciences Pharmingen, San Diego, CA) was applied as control[1,2]. A minimum of 10,000 cells were counted. Quadrants were set based upon the isotype controls for each antibody. Results were expressed as the percentage of CD69 expressing cells of the respective cell types (CD4+/CD3+;CD8+/CD3+;CD19+/CD45+;CD56+/CD45+).

Cytokine assay

Cytokines (Interleukin [IL]-1,-5,-6,-10,-13, interferon[IFN]-γ, tumor necrosis factor[TNF]α and -β were determined in the PBMC supernatants as recently described using antibody pairs and recombinant cytokines as standards (Pharmingen,San Diego,CA,USA)[1,2]. Normal values were defined as 100pg/ml for TNFβ and IL-17, 150pg/ml for IL-10, 250pg/ml for IL-6, 300pg/ml for IL-13 and TNFα, 500pg/ml for IL-1 and IL-5, and 1,300pg/ml for IFNγ.

Cell viability

Viability and function of the PBMC was evaluated by incubating them in parallel with poke weed mitogen (PWM; 40µg/ml, Biochrom AG, Berlin, Germany)[1,2], activation of subpopulations was proven by incubation with the recall-antigens Bacillus Calmette-Guérin (BCG), purified protein derivative of Mycobacterium tuberculosis (PPD), and tetanus toxoid (TT)[1,2]. PBMC which were not activated by any of these mitogens/antigens were omitted from further analyses.

8.2. Results from immunological assessments

ELISA

Patients were not prescreened for anti-AAV8 antibodies. No induction of antibodies to AAV8 capsid epitopes was observed in any of the patients regardless of dose group and/or time-point tested (**eTable 5**).

PBMC cultures

PBMC were tested in samples from all patients at screening, 30±5, 90±7 and 180±7 days after surgery in order to test PBMC sub-fractions (CD4+ T-cells, CD8+ T-cells, CD19+ B-cells or CD56+ natural killer cells) on their activation/proliferation potential after exposure to AAV8, vehicle or unspecific stimulants (PPD, TT or BCG, **eTable 6**). Samples from the medium and high dose cohorts showed increased vector-induced proliferation at 90 to 180 days after gene therapy. Such a proliferation was not induced by the recall antigens.

Regarding the activation (CD69 expression) in different PBMC subclasses, CD4+ T-cells featured increased activation after exposure to AAV8, but also after exposure to PPD and TT

© 2020 Fischer MD et al. *JAMA Ophthalmology.*

antigens. CD8+ T-cells showed no reactivity after exposure to AAV8 or other recall antigens. CD19+ B-cells and CD56+ NK-cells presented an activation pattern similar to CD4+ T-cells with increased CD69 expression after stimulation with AAV8, PPD or TT antigen (especially at day 90 through day 180 and more so for samples from medium and high dose subjects).

The quantification of key cytokine levels showed elevated levels of GM-CSF, IFNγ and TNFβ after exposing the PBMCs to AAV8 at day 90 and 180 in samples from medium and high dose subjects. There was some induction of IL-1a at day 90 in medium and high dose samples when stimulating with AAV8, but also after stimulating with PPD.

In conclusion, there was no discernable humoral immune response to AAV8 following the subretinal vector delivery. On a cellular level, the vector seems to increase the reactivity of CD4+ TH1-cells following application of the medium or high dose 3 and 6 months after treatment. The vector also seems to activate B-cells. We observed only a marginal influence of the vaccination with the vector on general immune response towards recall antigens. Thus, during treatment an increase of AAV8.CNGA3-specific proliferation of PBMC was observed at day 90 which later again decreased or became negative. Moreover, there was an antigen-specific activation of CD19+ B cells as well as CD4+ and CD8+ T-cells and CD56+ NK cells, also beginning in most instances at day 90, verifying the induction of an immune response towards AAV8.CNGA3 during treatment. In one patient (patient 107) an antigen-specific response was observed already before treatment. Incubation of PBMC with AAV8.CNGA3 also induced the production of GM-CSF, the TH1-cytokines IFNγ and TNFβ, of IL-17, in one patient of the TH2-cytokine IL-5, as well as of the macrophage/monocyte-related cytokines IL-1α and IL-6. In contrast, the production of IL-10, IL-13, and TNFα was hardly affected. However, not only the antigen-specific but also the recall-antigen induced immune response increased during treatment resulting preferentially in proinflammatory reactions.

9. Exploration of secondary endpoints

9.1. Ophthalmological endpoints

In general, exploration of secondary efficacy endpoints was concordant with a functional gain due to the intervention. With some outcome measures, significant results were obtained despite the small sample size of nine patients. This was true for best corrected visual acuity, contrast sensitivity and the global (ellipse) and protan-specific color tests. Pupillography, which was performed consensually (i.e. one eye was stimulated and the other eye was measured), also showed significant changes observed both for diameter and relative constriction after red light stimulus, and for the diameter after both blue light stimuli. Overall 84 tests of significance

© 2020 Fischer MD et al. *JAMA Ophthalmology.*

were performed for the treatment eye and thus 4 or 5 seemingly significant results were expected by chance (**eTable 8**). In the analysis, however, 22 significant results were observed. This supports the notion of a treatment effect. However, only 5 significant results were observed for the difference between treatment and control eye and this was close to the number 4.2 expected by chance. It cannot be concluded whether this was due to the statistical noise introduced by the control eye or due to learning effects. Supplementary **eTable 8** summarizes the statistical comparisons of the ophthalmological outcomes. In the second column the tendency of differences is given (↑ = values increase, ↓ = values decrease). In columns 3 to 10 eight different statistical tests are presented: The first four tests refer to the treatment eye, the remaining four tests refer to the difference between treatment and control eye. Within these groups, the first two tests refer to the comparison of visit 9 (6 months after treatment) with visit 1 (screening) and the other two tests refer to the comparison of visit 10 (1 year after treatment) with visit 1. This was done as for visit 9 and visit 10 results were often quite different. Finally, a distinction is made between parametrical testing (two-sided, paired t-test) and non-parametrical testing (Wilcoxon signed rank test). Outcomes with at least one significant ($p < 0.05$) result are printed bold face as are the corresponding p-values in columns 3 to 8.

9.2. Patient reported outcome measures

Patient related outcome measures were assessed with three instruments: a study specific scale (A3-PRO), the National Eye Institute-Visual Function Questionnaire (VFQ-25), and the Brief Symptom Inventory (BSI)[10-12]. In the first instrument (A3-PRO), changes to baseline were documented (**eTable 9**), in the others absolute values with baseline measurements. Thus, tests of significance refer to the reference value “zero” (i.e. no change) for the A3-PRO and to comparisons vs. baseline for the VFQ 25 and the BSI (**eTables 10-11**). The item ‘general satisfaction’ was scaled on a 5-point Likert scale with values 1-2 indicating an unfavorable course, 3 being neutral, and 4-5 indicating a favorable course. Note that high values for the A3-PRO and the VFQ25 indicate an improvement, whereas low values indicate improvements in the BSI results. For the A3 (PRO) three significant improvements were observed (expected false-positives: one or two), for the VFQ 25, six significant improvements were observed (expected by chance: one or two), for the BSI, two were expected but 16 were observed. This is in line with the results of the ophthalmological outcomes and again supports the notion of a treatment effect. The positive results were obtained for the identification of colors (both instruments), the identification of letters and numbers, near activities, role difficulties, and peripheral vision (VFQ 25), and nine of thirteen scales of the BSI. eTables 9-11 summarize the results of each questionnaire.

© 2020 Fischer MD et al. *JAMA Ophthalmology.*

eTable 1. Flowchart of Visits and Procedures

**Visit**	**1**	**2**	**3**	**4**	**5**	**6**	**7**	**8**	**9**	**10**	**11-14**
	Screening	D0	D1	D2	D3	D14	D30	D90	D180	D365<br>Close out	FUP<br>M24, M36, M48, M60
**Time window**	day -30 to -2				± 1 day	± 3 days	± 5 days	± 7 days	± 7 days	± 14 days	± 1 month
**Procedure**											
Medical /surgical/ history	x										
Ocular / surgical/ history	x										
BMI	x									x	
Vital signs	x	x	x	x	x	o	o	o	o	x	o
Urine pregnancy test	x	x				x	x	x	x	x	
Hematology/basic chemistry/urine analysis	x	o	o	o	x	x	o	o	x	x	x
CRP, IgG, IgM	x			x	x	x	x	o	o	o	o
Immunopathology [a]	x						x	x	o	o	o
PCR of rAAV8 genome	x				x	x	o	o	o	o	o
Best corrected visual acuity	x					x	x	x	x	x	x
Basic ophthalmological exam (miosis)	x	x	x	x	x	x	x	x	x	x	x
Contrast sensitivity (PR charts)	x					x	x	x	x	x	x
Flicker fusion frequency	x						x	x	x	x	
Colour constancy (chromatic adaptation) [b]	x						o	o	x	x	o
Anomaloscopy [b]	x					x	x	x	x	x	o
Cambridge Colour Test [b] / Panel D15 [c]	x					x	x	x	x	x	o
IR-Video-Pupillography	x						x	x	x	x	o
Microperimetry (20°)	x						x	x	x	x	x
Fundoscopy (mydriasis)	x	x	x	x	x	x	x	x	x	x	x
Dark adaptation test	x						x	x	x	x	o
sdOCT	x	o	o	o	o	x	x	o	o	x	x

© 2020 Fischer MD et al. *JAMA Ophthalmology.*

Fundus autofluorescence	x					x	x	o	o	x	x
Fundus photography	x					x	x	x	o	x	x
ICG (Indocyanin green) angiography	x					o	o	x	o	x	o
Systemic steroids [d]	x (D -1)	x	x	x	x						
Topical steroids and antibiotics [d]	x (D -1)	x	x	x	x	x					
Subretinal injection of rAAV8 vector		x									
NEI VFQ-25	x						x		x	x	o
Psychiatric examination	x										
Brief Symptom Inventory (BSI)	x					x	x	x	x	x	
Psychological counselling [e]		x	x	x	x	x	x	x	x	x	
Study specific scale, A3-PRO						x	x	x	x	x	x
Adverse event recording		x	x	x	x	x	x	x	x	x	x
Concomitant medication	x	x	x	x	x	x	x	x	x	x	x
HIV Test	x										

eTable 1 x: Procedure/examination is scheduled, o: procedure optional (if control deemed necessary by investigator), FUP: Follow-up visit, [a] including but not limited to: Anti-AAV8 capsid ELISA, [b] if the previous tests have shown improved results, [c] if CCT is not feasible, [d] See section 7.2-7.3 on concomitant medication, [e] at patient's need and discretion.

© 2020 Fischer MD et al. *JAMA Ophthalmology.*

eTable 2: Classification of reported adverse events (number of reports)

Specification	Plausible Relationship to Study Drug or Study Procedure			
	**Not probable**	**Possible**	**Probable**	**Sure**
Related to Study Drug	n/a	0	0	0
Related to Study Procedure	n/a	2	1	32
Related to Study Drug and Procedure	n/a	1	1	0
No Specification	18	n/a	n/a	n/a

eTable 2 For 1 AE relationship was reported as not classifiable. N/a = not applicable

eTable 3: Findings in blood tests (number of reports)

Time point	*Screening*	*Day 2*	*Day 3*	*Week 2*	*Month 1*	*Month 3*	*Month 6*	*Year 1*
**hematology**								
results outside reference range	6/9	0/2	0/7	9/9	8/9	4/9	4/9	6/9
clinically significant abnormalities	0/9	0/2	0/7	1/9	0/9	0/9	0/9	0/9
**basic chemistry**								
results outside reference range	7/9	0/2	7/7	6/9	9/9	5/9	8/9	6/9
clinically significant abnormalities	0/9	0/2	0/7	0/9	0/9	0/9	0/9	0/9
**immune markers (CRP, IGG, IGM)**								
results outside reference range	4/9	2/9	1/7	2/9	3/9	2/4	3/4	2/3
clinically significant abnormalities	0/9	0/9	0/7	0/9	0/9	0/4	0/4	0/3
**urine analysis**								
results outside reference range	2/9	0/2	2/7	2/9	1/9	1/8	2/9	0/9
clinically significant abnormalities	0/9	0/2	0/7	0/9	0/9	0/8	0/9	0/9

eTable 3 First number: patients with findings / second number: patients tested

eTable 4. Reporting of vector genome copies detected over time

Patient		***101***	***102***	***103***	***104***	***105***	***106***	***107***	***108***	***109***
		low dose			intermediate dose			high dose		
**Blood**	Screening	–	–	–	–	–	–	–	–	–
	Day 3	–	–	–	–	–	–	–	–	–
	Day 14	–	–	–	–	–	–	–	–	–
	Day 30	–	–	–	–	–	–	–	–	–
	Day 90	–	–	–	–	–	–	–	–	–
**Saliva**	Screening	–	–	–	–	–	–	–	–	–
	Day 3	–	–	–	–	–	–	–	–	–
	Day 14	–	–	–	–	–	–	–	–	–
	Day 30	–	–	–	–	–	–	–	–	–
	Day 90	–	–	–	–	–	–	–	–	–
**Urine**	Screening	–	–	–	–	–	–	–	–	–

© 2020 Fischer MD et al. *JAMA Ophthalmology.*

	Day 3	–	–	–	–	–	–	–	–	–
	Day 14	–	–	–	–	–	–	–	–	–
	Day 30	–	–	–	–	–	–	–	–	–
	Day 90	–	–	–	–	–	–	–	–	–
**Tears**	Screening	–	–	–	–	–	–	–	–	–
	Day 3	–	–	–	–	–	–	–	–	–
	Day 14	–	–	–	–	–	–	–	–	–
	Day 30	–	–	–	–	–	–	–	–	–
	Day 90	–	–	–	–	–	–	–	–	–

eTable 4 Virus shedding. Samples were deemed negative (–) if data indicated levels below the limit of detection (800 vector genomes per sample).

eTable 5 Titers of anti-AAV8 antibodies over time (ELISA)

	patients								
	**101**	**102**	**103**	**104**	**105**	**106**	**107**	**108**	**109**
	low dose			intermediate dose			high dose		
	**IgG anti-AAV8 antibodies (ELISA; absorption x 1,000)**								
Screening	n.t.	122	64	48	70	47	63	85	68
Day 30	135	70	61	40	50	129	16	32	35
Day 90	37	138	50	51	61	54	37	36	36
Day 180	107	88	35	37	32	84	37	22	37
Day 365	97	64	46	44	45	36	78	55	18
	**IgM anti-AAV8 antibodies (ELISA; absorption x 1,000)**								
Screening	n.t.	40	67	38	38	29	39	23	29
Day 30	47	22	58	35	36	59	39	15	23
Day 90	31	45	56	57	39	31	12	22	14
Day 180	29	18	76	52	31	47	23	13	20
Day 365	45	33	59	50	46	18	20	30	16

eTable 5 Humoral immune response against AAV8. Serum samples were used to assess the titer of IgG and IgM antibodies against AAV8 capsid epitopes by ELISA. There was no clinically significant change observed in the titers against AAV8 epitopes in any of the samples tested. n.t.: not tested

eTable 6 Reaction profile of different immune cells against AAV8 capsid epitopes and recall antigens

**Antigens**		patients								
		**101**	**102**	**103**	**104**	**105**	**106**	**107**	**108**	**109**
		low dose			intermediate dose			high dose		
		**LTT: Proliferative response of PBMC (cpm)**								
**Control**										
no antigen	Screening	444	242	292	125	223	244	94	868	49
	Day 30	142	101	386	222	182	209	260	66	214
	Day 90	174	254	480	304	187	230	166	247	197
	Day 180	116	74	163	82	162	208	199	446	402
	Day 365	182	260	351	253	272	130	130	41	105
**Specific antigen:**										
AAV8.CNGA3	Screening	528	269	270	72	200	397	110	936	76
	Day 30	48	87	443	202	176	174	181	106	200
	Day 90	161	267	218	235	285	147	2,233	1,026	1,008
	Day 180	127	46	119	226	139	2,823	952	134	118
	Day 365	170	346	268	210	474	614	1,623	33	77
**Recall antigens**										
BCG	Screening	428	357	778	505	1,049	694	222	643	166
	Day 30	88	320	447	238	569	204	277	58	183
	Day 90	2,540	520	922	1,206	2,490	1,335	1,116	407	412

© 2020 Fischer MD et al. *JAMA Ophthalmology.*

	Day 180	2,145	350	270	354	1,695	1,029	707	182	239
	Day 365	660	646	338	357	1,955	604	2,331	504	646
PPD	Screening	257	683	n.t.	n.t.	n.t.	n.t.	71	1,126	266
	Day 30	79	n.t.	n.t.	n.t.	234	192	241	71	182
	Day 90	n.t.	n.t.	n.t.	344	977	227	385	348	251
	Day 180	n.t.	63	244	65	170	456	1,056	361	159
	Day 365	329	322	660	191	266	123	1,989	114	126
Tetanus toxoid	Screening	401	2,466	2,352	902	1,805	2,237	85	892	788
	Day 30	951	684	364	202	542	201	194	57	192
	Day 90	2,750	581	372	812	2,237	863	1,595	1,576	402
	Day 180	4,468	515	203	852	1,134	2,007	1,155	191	184
	Day 365	1,796	546	341	1,881	2,653	1,488	2,408	90	1,379
		**Flow cytometry: Activation of CD4+ T cells (percent CD4+CD69+ T cells)**								
**Control**										
no antigen	Screening	0.5	0.4	0.4	0.1	0.2	0.1	3.2	0.6	0.1
	Day 30	0.1	0.1	0.0	0.6	0.1	0.0	1.3	0.8	0.2
	Day 90	1.5	0.1	0.1	0.2	0.1	0.6	2.8	0.5	0.3
	Day 180	0.2	0.2	0.2	0.3	0.2	0.2	1.6	0.6	2.2
	Day 365	1.7	0.3	0.8	0.3	0.1	1.1	0.7	0.3	0.3
**Specific antigen:**										
AAV8.CNGA3	Screening	0.3	0.5	0.2	0.3	0.4	0.8	6.9	0.9	0.9
	Day 30	4.0	1.1	0.6	0.2	0.2	1.9	1.1	2.7	0.5
	Day 90	0.6	0.8	0.7	0.6	0.3	1.1	2.4	18.6	2.2
	Day 180	0.4	0.4	0.7	0.7	0.3	4.5	9.5	0.5	6.5
	Day 365	1.3	2.5	2.5	0.4	2.0	1.1	7.8	0.3	1.1
**Recall antigens**										
BCG	Screening	4.7	4.9	3.7	4.7	5.4	4.8	24.6	12.3	1.6
	Day 30	19.9	13.5	9.3	28.6	3.5	30.1	23.2	4.6	16.3
	Day 90	3.9	2.8	17.0	7.4	11.1	39.9	4.1	25.4	2.1
	Day 180	5.6	9.1	8.3	2.4	9.8	3.1	4.2	2.5	2.6
	Day 365	7.3	4.2	4.4	7.0	2.2	1.3	12.4	1.6	4.7
PPD	Screening	0.1	0.2	n.t.	n.t.	n.t.	n.t.	2.0	0.5	1.0
	Day 30	1.4	n.t.	n.t.	n.t.	n.t.	0.5	0.3	0.5	1.0
	Day 90	n.t.	n.t.	n.t.	0.8	0.3	1.7	1.3	7.7	3.2
	Day 180	n.t.	0.0	1.4	0.5	0.8	3.8	4.3	4.3	3.0
	Day 365	0.7	19.7	4.5	0.2	0.2	0.4	1.5	0.3	0.9
Tetanus toxoid	Screening	0.4	0.4	0.6	0.2	0.8	0.4	2.4	0.4	0.6
	Day 30	1.2	1.4	0.3	0.1	0.5	1.9	0.6	1.0	0.2
	Day 90	2.0	0.2	1.8	0.8	0.5	2.3	9.0	24.5	4.7
	Day 180	1.0	0.5	2.3	0.4	16.7	3.0	3.4	5.1	1.7
	Day 365	0.7	0.5	3.1	0.3	0.6	0.8	1.0	0.5	0.8
		**Flow cytometry: Activation of CD8+ T cells (percent CD8+CD69+ T cells)**								
**Control**										
no antigen	Screening	2.9	21.9	25.1	5.6	0.8	5.9	45.1	6.2	5.6
	Day 30	8.6	11.0	3.0	0.0	1.1	0.0	44.7	4.5	9.8
	Day 90	27.6	2.6	9.6	3.6	0.8	9.5	49.8	2.0	22.1
	Day 180	3.0	39.1	3.3	2.1	2.3	4.0	n.t.	11.4	35.6
	Day 365	8.0	11.1	0.5	15.5	0.9	21.6	57.9	8.1	30.0
**Specific antigen:**										
AAV8.CNGA3	Screening	1.9	23.6	62.7	10.6	1.7	6.2	39.8	4.5	11.4
	Day 30	31.4	31.3	2.6	3.6	1.3	1.7	60.3	3.6	6.8
	Day 90	22.3	6.4	12.1	2.4	1.5	7.7	42.1	10.2	24.4
	Day 180	4.6	30.6	5.2	2.7	1.7	21.7	n.t.	5.9	26.9
	Day 365	10.3	20.7	9.6	13.1	4.0	39.0	44.3	4.0	8.5
**Recall antigens**										
BCG	Screening	10.8	43.7	46.1	16.7	18.0	41.8	53.6	9.2	12.5
	Day 30	29.5	71.3	13.2	45.4	7.1	22.9	64.7	5.7	16.1
	Day 90	22.7	20.3	34.9	20.9	6.4	20.6	56.0	9.7	24.3
	Day 180	10.4	100.0	10.7	6.2	9.0	29.0	n.t.	7.2	34.5
	Day 365	11.9	24.3	11.9	39.8	6.6	26.4	86.0	6.0	32.8
PPD	Screening	2.6	18.9	n.t.	n.t.	n.t.	n.t.	43.7	4.0	10.2
	Day 30	14.0	n.t.	n.t.	n.t.	n.t.	0.8	44.5	2.2	8.0
	Day 90	n.t.	n.t.	n.t.	2.8	2.0	7.3	57.8	7.6	32.0
	Day 180	n.t.	35.7	4.5	1.3	0.3	58.7	n.t.	12.4	21.8

© 2020 Fischer MD et al. *JAMA Ophthalmology.*

	Day 365	11.2	49.8	21.3	8.3	0.9	15.3	40.0	4.8	31.6
Tetanus toxoid	Screening	3.6	32.3	42.3	7.3	10.0	24.8	41.4	9.2	8.1
	Day 30	18.1	53.2	1.9	4.4	2.5	1.2	44.4	3.9	7.9
	Day 90	22.1	18.2	16.2	4.3	2.2	6.9	62.4	7.6	28.5
	Day 180	6.7	97.6	4.1	2.1	7.2	42.3	n.t.	10.1	26.8
	Day 365	11.5	25.3	12.7	13.7	2.7	24.4	55.2	12.5	35.6
		**Flow cytometry: Activation of CD19+ B cells (percent CD19+CD69+ B cells)**								
**Control**										
no antigen	Screening	3.7	0.1	4.0	6.8	0.6	4.1	12.8	6.2	3.2
	Day 30	5.4	5.0	5.1	0.8	2.4	1.1	4.9	7.6	0.0
	Day 90	3.7	1.2	6.7	1.2	1.8	0.9	17.8	3.7	3.0
	Day 180	3.8	0.0	7.6	3.4	4.2	8.5	25.7	5.6	33.8
	Day 365	9.6	0.0	0.0	4.2	5.4	4.4	7.1	0.0	12.3
**Specific antigen:**										
AAV8.CNGA3	Screening	4.0	8.8	10.3	4.2	2.0	5.4	56.6	4.5	6.5
	Day 30	14.9	7.8	5.0	2.4	3.8	4.8	7.0	23.9	4.5
	Day 90	8.3	2.3	14.2	10.3	6.0	5.6	52.1	76.7	48.9
	Day 180	5.3	4.0	9.2	3.5	4.6	39.1	60.9	21.4	68.0
	Day 365	11.9	48.5	50.2	0.0	27.5	18.8	18.1	7.7	26.1
**Recall antigens**										
BCG	Screening	31.5	51.6	44.5	16.7	28.2	62.7	63.5	9.2	41.1
	Day 30	68.8	64.2	30.0	45.4	23.9	47.2	39.4	44.1	54.7
	Day 90	21.1	33.7	63.9	27.1	44.2	67.1	24.2	62.1	48.2
	Day 180	55.1	55.5	37.2	25.3	44.3	52.1	39.1	59.1	57.3
	Day 365	13.0	58.6	64.3	45.5	33.4	59.9	32.2	50.9	69.1
PPD	Screening	3.8	6.0	n.t.	n.t.	n.t.	n.t.	31.3	4.0	18.8
	Day 30	5.7	n.t.	n.t.	n.t.	n.t.	2.3	4.6	1.0	5.9
	Day 90	n.t.	n.t.	n.t.	4.0	5.0	4.6	13.5	37.3	55.2
	Day 180	8.0	0.0	5.8	1.5	2.6	24.4	38.5	42.7	34.5
	Day 365	n.t.	75.9	68.3	0.0	4.9	2.6	7.9	8.6	18.8
Tetanus toxoid	Screening	2.4	3.4	25.1	7.3	10.0	8.7	11.0	9.2	4.6
	Day 30	9.5	13.1	5.4	4.4	2.5	3.4	3.9	4.3	54.7
	Day 90	6.5	3.5	17.8	1.7	9.1	11.1	33.9	75.9	50.9
	Day 180	8.1	5.5	6.7	4.1	56.5	31.4	21.8	59.9	27.2
	Day 365	11.2	15.3	51.9	5.6	9.5	27.0	7.2	0.0	20.7
		**Flow cytometry: Activation of CD56+ NK cells (percent CD56+CD69+ NK cells)**								
**Control**										
no antigen	Screening	0.9	9.4	8.2	4.7	0.6	4.5	29.2	2.9	4.6
	Day 30	2.4	11.1	2.7	1.6	2.4	1.6	31.3	1.8	10.0
	Day 90	5.5	4.6	1.9	2.7	7.2	3.5	60.6	6.2	8.2
	Day 180	4.8	4.0	7.4	5.1	9.0	3.6	54.9	5.1	15.7
	Day 365	7.3	12.2	3.5	8.3	8.0	7.0	50.6	3.0	12.4
**Specific antigen:**										
AAV8.CNGA3	Screening	1.1	11.5	13.0	17.8	4.2	5.7	27.1	29.8	6.4
	Day 30	10.9	21.2	5.8	2.7	8.1	4.8	33.6	3.7	8.7
	Day 90	4.6	10.4	5.0	3.4	5.4	5.2	50.9	36.8	22.5
	Day 180	6.0	6.5	7.3	8.2	14.5	58.5	47.6	7.4	34.9
	Day 365	5.3	29.8	28.3	8.9	27.5	22.4	38.0	1.9	9.3
**Recall antigens**										
BCG	Screening	31.5	53.3	44.9	64.6	46.7	31.9	56.8	27.2	19.6
	Day 30	71.1	80.0	26.5	34.2	54.7	51.1	46.2	30.0	31.4
	Day 90	51.1	44.2	70.6	24.5	38.6	49.5	59.9	50.7	27.5
	Day 180	26.5	27.6	28.3	43.1	53.5	21.4	61.9	25.8	34.1
	Day 365	21.5	41.3	51.0	26.2	30.1	40.5	47.7	11.4	32.4
PPD	Screening	11.5	12.4	n.t.	n.t.	n.t.	n.t.	29,2	3,5	15,5
	Day 30	5.8	n.t.	n.t.	n.t.	n.t.	2.0	26,6	9,8	7,9
	Day 90	n.t.	n.t.	n.t.	2.8	4.4	5.1	48,3	17,5	35,8
	Day 180	n.t.	3.5	6.5	5.8	1.9	33.9	59.3	22.6	11.9
	Day 365	3.8	55.2	25.5	6.2	8.5	5.9	37.7	2.4	9.5
Tetanus toxoid	Screening	2.0	25.1	21.2	10.6	21.5	19.7	30.1	6.2	10.1
	Day 30	13.0	65.7	3.7	3.3	13.2	4.7	29.2	8.1	10.7
	Day 90	18.8	26.5	8.0	8.0	13.9	7.5	52.7	47.9	37.6
	Day 180	11.4	18.5	7.9	7.8	46.8	55.5	57.0	32.1	17.0
	Day 365	10.7	22.6	26.7	15.7	17.1	39.9	49.1	1.8	2.9

© 2020 Fischer MD et al. *JAMA Ophthalmology.*

		**Cytokine- ELISA: GM-CSF (pg/ml) pos > 300**								
**Control**										
no antigen	Screening	0	0	708	0	513	0	808	0	0
	Day 30	0	0	0	0	356	0	1,274	0	0
	Day 90	0	0	160	0	683	0	1,411	0	0
	Day 180	0	0	465	0	850	0	1,146	0	0
	Day 365	0	0	436	0	320	0	486	0	0
**Specific antigen:**										
AAV8.CNGA3	Screening	460	0	843	0	850	0	984	0	0
	Day 30	0	0	0	0	494	0	1,172	1,026	0
	Day 90	0	0	0	0	841	325	1,852	1,516	2,068
	Day 180	0	0	1,424	0	1,228	3,318	3,985	0	1,481
	Day 365	460	1,464	2,410	0	582	443	1,120	0	0
**Recall antigens**										
BCG	Screening	1,814	1,939	2,997	2,114	4,050	2,257	1,556	3,167	3,967
	Day 30	177	1,104	4,161	598	1,220	196	1,640	1,503	520
	Day 90	3,925	1,173	1,564	2,645	3,040	2,257	2,641	4,405	4,473
	Day 180	3,964	3,297	1,974	1,786	2,842	2,943	4,001	2,767	2,397
	Day 365	4,724	4,743	1,415	1,404	1,184	1,109	1,414	1,723	1,546
PPD	Screening	0	612	n.t.	n.t.	n.t.	n.t.	883	186	10,000
	Day 30	0	n.t.	n.t.	n.t.	n.t.	0	941	0	0
	Day 90	n.t.	n.t.	n.t.	0	934	0	1,262	815	2,758
	Day 180	n.t.	426	540	0	763	0	2,728	679	0
	Day 365	5,847	1,661	1,485	0	n.t.	0	432	0	0
Tetanus toxoid	Screening	871	5,367	2,724	2,190	5,902	1,958	761	631	1,660
	Day 30	0	313	0	0	2,587	0	1,198	0	0
	Day 90	10,000	879	692	1,695	3,664	478	2,016	1,224	992
	Day 180	9,245	8,614	425	211	1,836	2,185	1,362	0	635
	Day 365	10,000	2,346	730	943	1,184	497	1,077	0	1,059
		**Cytokine- ELISA: IFNγ (pg/ml) (pos > 1.250)**								
**Control**										
no antigen	Screening	0	0	194	175	1,116	0	0	0	0
	Day 30	0	0	0	202	1,062	0	0	0	0
	Day 90	0	0	0	293	1,688	0	0	0	0
	Day 180	0	0	0	6,583	1,457	0	0	0	0
	Day 365	0	0	0	0	512	0	0	0	0
**Specific antigen:**										
AAV8.CNGA3	Screening	0	0	174	215	1,457	0	0	0	0
	Day 30	0	318	418	409	1,089	0	14,894	0	0
	Day 90	0	382	109	1,033	2,173	0	14,594	2,494	2,144
	Day 180	0	181	347	434	2,395	40,000	7,107	0	0
	Day 365	0	2,485	0	0	356	5,557	4,822	0	0
**Recall antigens**										
BCG	Screening	1,460	2,262	2,624	1,120	14,151	1,730	0	2,064	0
	Day 30	0	130	310	241	4,690	0	0	0	0
	Day 90	16,872	2,294	863	4,474	19,930	3,216	7,618	1,497	1,074
	Day 180	8,878	1,309	1,336	1,406	8,012	4,533	7,473	0	945
	Day 365	9,925	2,863	0	0	1,238	1,226	5,953	2,611	0
PPD	Screening	0	205	n.t.	n.t.	n.t.	n.t.	0	0	6,232
	Day 30	0	n.t.	n.t.	n.t.	n.t.	0	0	0	0
	Day 90	n.t.	n.t.	n.t.	358	1,767	0	0	4,941	3,446
	Day 180	n.t.	581	0	280	2,331	13,444	8,402	1.117	0
	Day 365	5,384	6,121	0	0	0	0	0	0	0
Tetanus toxoid	Screening	0	340	1,588	934	3,279	1,271	0	0	0
	Day 30	0	0	253	294	1,553	0	0	0	0
	Day 90	10,760	713	153	1,369	2,395	0	7,589	2,677	2,310
	Day 180	5,896	732	194	396	3,339	24,029	0	0	0
	Day 365	4,336	0	0	0	497	8,648	0	0	0
		**Cytokine- ELISA: IL-10 (pg/ml) (pos > 150)**								
**Control**										
no antigen	Screening	0	0	0	0	291	0	0	0	0
	Day 30	0	0	0	0	457	0	0	0	0
	Day 90	0	0	0	0	611	0	0	0	0
	Day 180	0	0	0	0	433	0	0	0	0

© 2020 Fischer MD et al. *JAMA Ophthalmology.*

	Day 365	0	0	0	0	705	0	135	0	79
**Specific antigen:**										
AAV8.CNGA3	Screening	177	0	0	0	421	0	0	0	0
	Day 30	0	0	0	0	363	0	165	0	0
	Day 90	0	0	82	0	623	0	0	0	0
	Day 180	89	0	95	435	539	0	0	0	0
	Day 365	0	0	201	0	705	0	0	0	273
**Recall antigens**										
BCG	Screening	524	0	0	0	350	0	0	0	490
	Day 30	0	0	0	0	421	0	201	0	0
	Day 90	0	0	0	257	1,543	0	347	307	280
	Day 180	81	0	0	0	708	829	357	0	223
	Day 365	240	0	690	467	892	193	0	203	235
PPD	Screening	0	0	n.t.	n.t.	n.t.	n.t.	0	0	0
	Day 30	0	n.t.	n.t.	n.t.	n.t.	0	0	0	0
	Day 90	n.t.	n.t.	n.t.	0	327	0	0	0	0
	Day 180	n.t.	0	0	0	339	0	0	0	0
	Day 365	0	743	424	0	736	0	0	0	0
Tetanus toxoid	Screening	297	562	0	0	386	86	0	0	0
	Day 30	0	95	0	0	1,100	0	0	0	0
	Day 90	89	0	0	1,673	846	0	0	0	0
	Day 180	451	2,548	343	188	386	0	297	0	0
	Day 365	211	224	334	774	970	0	221	0	153
		**Cytokine- ELISA: IL-13 (pg/ml) (pos > 300)**								
**Control**										
no antigen	Screening	0	0	3,744	0	420	2,255	9,829	322	0
	Day 30	0	0	2,357	0	391	2,537	4,040	310	0
	Day 90	1,838	0	2,163	0	656	3,254	10,000	631	0
	Day 180	0	0	3,098	0	672	2,883	10,000	0	0
	Day 365	0	0	10,000	0	4,000	2,308	10,000	0	0
**Specific antigen:**										
AAV8.CNGA3	Screening	1,101	0	4,209	0	522	2,411	10,000	444	0
	Day 30	0	0	2,593	0	397	2,956	10,000	334	0
	Day 90	0	426	2,211	202	738	3,377	10,000	690	320
	Day 180	0	0	3,933	0	788	3,788	10,000	0	0
	Day 365	1,333	467	10,000	0	4,207	3,252	10,000	674	0
**Recall antigens**										
BCG	Screening	0	0	3,423	0	589	2,231	8,927	657	0
	Day 30	0	0	2,061	0	338	2,762	7,701	0	0
	Day 90	1,549	0	2,292	403	837	3,010	10,000	707	1,743
	Day 180	385	0	3,402	169	892	3,224	10,000	444	279
	Day 365	1,697	1,193	10,000	0	4,242	2,639	10,000	1,850	0
PPD	Screening	0	0	n.t.	n.t.	n.t.	n.t.	10,000	811	526
	Day 30	0	n.t.	n.t.	n.t.	n.t.	2,753	10,000	0	0
	Day 90	n.t.	n.t.	n.t.	0	639	3,199	10,000	1,475	406
	Day 180	n.t.	0	3.227	0	639	3,244	10,000	406	0
	Day 365	2,462	558	10,000	0	3,966	2,639	10,000	0	0
Tetanus toxoid	Screening	0	4,595	4,957	2,391	7,357	4,058	8,986	992	3,280
	Day 30	0	0	2,308	0	1,206	2,782	8,394	0	0
	Day 90	10,000	753	2,155	2,204	4,572	3,305	10,000	780	704
	Day 180	10,000	10,000	3,755	0	1,304	3,991	10,000	704	2,915
	Day 365	10,000	10,000	10,000	4,093	10,000	10,000	10,000	1,158	5,271
		**Cytokine- ELISA: IL-17 (pg/ml) (pos > 30)**								
**Control**										
no antigen	Screening	0	0	0	0	0	0	0	39	0
	Day 30	0	0	0	0	100	0	0	39	0
	Day 90	0	0	0	0	100	0	0	0	0
	Day 180	0	0	0	0	143	0	0	0	0
	Day 365	0	0	0	0	0	0	0	0	0
**Specific antigen:**										
AAV8.CNGA3	Screening	0	0	49	0	134	71	0	47	0
	Day 30	0	0	0	0	503	0	18	58	0
	Day 90	0	0	157	0	503	0	94	97	0
	Day 180	0	0	0	0	152	144	224	23	0

© 2020 Fischer MD et al. *JAMA Ophthalmology.*

	Day 365	0	146	0	0	0	0	33	0	0
**Recall antigens**										
BCG	Screening	0	260	183	262	1,000	142	0	118	33
	Day 30	0	260	0	0	1,000	0	0	67	0
	Day 90	0	0	170	966	1,000	168	180	81	68
	Day 180	0	417	99	151	1,000	172	909	58	96
	Day 365	390	297	0	105	102	87	178	35	40
PPD	Screening	0	0	n.t.	n.t.	n.t.	n.t.	0	196	0
	Day 30	0	0	n.t.	n.t.	0	0	18	0	0
	Day 90	n.t.	n.t.	n.t.	0	0	0	0	252	0
	Day 180	265	n.t.	0	0	0	0	151	0	0
	Day 365	275	214	114	0	0	0	0	0	0
Tetanus toxoid	Screening	0	0	504	0	208	212	0	709	35
	Day 30	0	0	0	0	100	0	0	34	0
	Day 90	0	0	0	96	100	0	120	150	0
	Day 180	0	0	73	0	0	277	67	42	0
	Day 365	36	0	0	22	0	22	0	17	0
		**Cytokine- ELISA: IL-1α (pg/ml) (pos > 500)**								
**Control**										
no antigen	Screening	546	0	0	0	0	0	0	0	0
	Day 30	0	0	0	0	0	0	0	0	0
	Day 90	873	0	0	0	0	0	0	0	0
	Day 180	0	0	0	0	0	540	0	0	0
	Day 365	456	0	0	0	3,103	0	0	0	0
**Specific antigen:**										
AAV8.CNGA3	Screening	1,120	0	0	0	0	0	0	0	2,501
	Day 30	0	0	0	0	0	0	2,132	2,132	0
	Day 90	0	0	0	0	0	0	1,405	1,405	1,503
	Day 180	297	0	0	0	0	11,586	4,540	0	2,501
	Day 365	0	6,313	10,168	0	3,547	1,527	5,225	0	0
**Recall antigens**										
BCG	Screening	2,118	9,548	8,022	8,758	6,245	2,431	3,206	8,736	16,000
	Day 30	1,953	16,000	3,390	3,159	7,395	2,431	4,240	4,240	4,711
	Day 90	6,151	14,572	8,334	16,000	8,542	4,905	7,592	7,592	14,294
	Day 180	10,151	16,000	7,436	7,111	11,581	16,000	13,461	4,220	16,000
	Day 365	16,000	16,000	16,000	16,000	16,000	9,988	16,000	16,000	16,000
PPD	Screening	1,530	0	n.t.	n.t.	n.t.	0	0	0	0
	Day 30	0	n.t.	n.t.	n.t.	n.t.	0	0	0	0
	Day 90	n.t.	n.t.	n.t.	0	0	0	290	290	2,538
	Day 180	n.t.	0	0	0	0	0	2,876	739	0
	Day 365	328	10,897	7,435	0	2,225	0	0	0	0
Tetanus toxoid	Screening	2,069	0	2,058	0	1,086	0	0	0	0
	Day 30	0	0	0	0	0	0	0	0	0
	Day 90	0	0	0	0	0	0	1,420	1,420	1,363
	Day 180	739	799	0	0	1,004	3,019	0	683	0
	Day 365	378	0	6,335	10,316	3,000	1,173	0	0	0
		**Cytokine- ELISA: IL-5 (pg/ml) (pos > 500)**								
**Control**										
no antigen	Screening	0	0	0	0	529	0	0	0	0
	Day 30	0	0	0	0	460	0	0	0	0
	Day 90	0	0	0	0	723	0	0	0	0
	Day 180	0	0	0	0	820	0	0	0	0
	Day 365	0	0	0	0	349	0	0	0	0
**Specific antigen:**										
AAV8.CNGA3	Screening	0	0	0	0	578	0	0	0	0
	Day 30	0	300	0	0	549	0	0	1,537	0
	Day 90	0	0	0	536	861	0	0	2,514	0
	Day 180	0	0	0	0	890	0	0	0	0
	Day 365	0	0	0	0	243	0	0	0	0
**Recall antigens**										
BCG	Screening	1,484	0	0	0	505	0	0	0	0
	Day 30	0	0	0	0	505	0	0	0	0
	Day 90	311	0	0	0	939	0	0	0	0
	Day 180	0	0	0	0	841	0	0	0	0

© 2020 Fischer MD et al. *JAMA Ophthalmology.*

	Day 365	0	266	0	0	392	0	0	0	0
PPD	Screening	0	0	n.t.	n.t.	n.t.	n.t.	0	0	0
	Day 30	0	n.t.	n.t.	n.t.	n.t.	0	0	0	0
	Day 90	n.t.	n.t.	0	0	878	0	0	329	0
	Day 180	n.t.	0	0	0	841	0	0	0	0
	Day 365	0	0	0	0	0	0	0	0	0
Tetanus toxoid	Screening	0	15,000	659	3,517	2,959	1,572	0	0	1,702
	Day 30	0	271	0	0	2,959	0	0	0	0
	Day 90	15,000	1,593	0	3,282	10,521	0	0	0	0
	Day 180	15,000	15,000	0	0	1,937	0	0	0	1,172
	Day 365	15,000	4,808	772	2,215	2,764	0	1,230	0	1,350
		**Cytokine- ELISA: IL-6 (pg/ml) (pos > 250)**								
**Control**										
no antigen	Screening	0	0	910	0	346	0	0	0	0
	Day 30	170	0	0	0	371	0	0	0	0
	Day 90	1.765	0	0	94	612	0	0	0	0
	Day 180	0	0	0	0	645	0	0	0	0
	Day 365	0	0	0	0	1.105	0	0	0	0
**Specific antigen:**										
AAV8.CNGA3	Screening	677	0	468	6.624	681	0	0	3.525	1.412
	Day 30	0	76	139	613	360	373	0	8.000	0
	Day 90	0	121	16	1.235	1.329	1.073	2.998	8.000	8.000
	Day 180	0	40	122	809	726	8.000	8.000	0	8.000
	Day 365	502	8.000	8.000	0	1.640	1.248	2.388	0	0
**Recall antigens**										
BCG	Screening	8.000	8.000	8.000	8.000	8.000	8.000	8.000	8.000	8.000
	Day 30	8.000	8.000	8.000	8.000	8.000	6.076	8.000	8.000	8.000
	Day 90	8.000	8.000	8.000	8.000	8.000	8.000	8.000	8.000	8.000
	Day 180	8.000	8.000	8.000	8.000	8.000	8.000	8.000	8.000	8.000
	Day 365	8.000	8.000	8.000	8.000	8.000	8.000	8.000	8.000	8.000
PPD	Screening	0	3.036	n.t.	n.t.	n.t.	n.t.	0	8.000	8.000
	Day 30	0	0	n.t.	n.t.	n.t.	0	379	0	6.924
	Day 90	0	n.t.	n.t.	18	789	2.075	0	6.924	8.000
	Day 180	n.t.	165	222	0	584	1.983	8.000	1.983	0
	Day 365	8.000	8.000	8.000	0	984	0	0	0	0
Tetanus toxoid	Screening	159	600	8.000	110	8.000	5.948	0	2.649	8.000
	Day 30	0	844	97	222	443	0	0	0	5.715
	Day 90	159	1.972	1.503	621	1.127	6.925	2.277	5.715	3.918
	Day 180	1.945	8.000	1.106	628	1.263	1.018	0	1.018	0
	Day 365	1.673	2.344	8.000	7.656	1.366	1.509	0	0	910
		**Cytokine- ELISA: TNFα (pg/ml) (pos > 300)**								
**Control**										
no antigen	Screening	0	44	0	83	54	0	0	0	0
	Day 30	0	0	0	0	46	0	0	0	0
	Day 90	0	5	0	4	69	0	0	0	0
	Day 180	0	46	0	0	69	0	0	0	0
	Day 365	0	0	0	0	0	0	0	0	0
**Specific antigen:**										
AAV8.CNGA3	Screening	0	59	0	0	50	0	0	0	0
	Day 30	0	81	0	0	108	0	0	0	0
	Day 90	0	15	0	0	178	0	0	0	0
	Day 180	0	8	0	11	92	976	0	0	0
	Day 365	0	0	0	0	0	0	0	0	0
**Recall antigens**										
BCG	Screening	0	133	116	146	203	0	0	247	640
	Day 30	0	292	70	53	213	0	0	0	0
	Day 90	0	287	130	208	167	0	242	231	167
	Day 180	0	296	141	208	176	0	0	0	0
	Day 365	0	606	393	1.192	0	0	580	621	229
PPD	Screening	0	20	n.t.	n.t.	n.t.	n.t.	0	0	1.352
	Day 30	0	n.t.	n.t.	n.t.	n.t.	0	0	0	0
	Day 90	n.t.	n.t.	n.t.	0	60	0	0	0	209
	Day 180	n.t.	10	46	0	84	0	0	0	0
	Day 365	244	0	0	0	0	0	0	0	0

© 2020 Fischer MD et al. *JAMA Ophthalmology.*

Tetanus toxoid	Screening	0	28	10	6	64	0	0	0	0
	Day 30	0	7	0	56	87	0	0	0	0
	Day 90	0	20	31	0	113	0	0	0	0
	Day 180	0	19	3	4	110	320	0	0	0
	Day 365	0	0	0	0	0	0	0	0	0
		**Cytokine- ELISA: TNFβ (pg/ml) (pos > 80)**								
**Control**										
no antigen	Screening	0	0	414	0	116	255	562	0	0
	Day 30	0	0	221	0	135	311	665	0	0
	Day 90	0	0	198	0	230	475	989	0	0
	Day 180	0	0	332	0	247	417	753	0	0
	Day 365	0	0	312	0	166	113	675	0	0
**Specific antigen:**										
AAV8.CNGA3	Screening	0	0	406	0	224	274	608	0	0
	Day 30	0	0	235	0	90	384	718	0	0
	Day 90	0	0	231	105	234	524	1.155	97	247
	Day 180	0	0	343	0	232	820	1.023	0	0
	Day 365	0	72	460	0	238	199	878	0	0
**Recall antigens**										
BCG	Screening	77	183	395	74	175	234	516	42	0
	Day 30	0	0	196	0	133	326	564	0	0
	Day 90	64	175	231	0	400	451	963	82	81
	Day 180	86	93	353	0	257	498	1.023	0	0
	Day 365	0	0	272	0	255	175	802	0	166
PPD	Screening	49	234	n.t.	n.t.	n.t.	n.t.	560	281	376
	Day 30	0	n.t.	n.t.	n.t.	n.t.	350	636	0	0
	Day 90	n.t.	242	n.t.	0	259	424	977	0	272
	Day 180	549	242	343	0	232	538	817	59	0
	Day 365	549	66	325	0	255	95	794	261	0
Tetanus toxoid	Screening	51	447	405	168	196	280	508	0	132
	Day 30	0	0	206	347	162	279	593	0	0
	Day 90	2.500	342	233	0	595	478	950	91	238
	Day 180	2.500	85	311	0	362	592	726	0	41
	Day 365	2.500	191	309	0	911	236	757	0	120

eTable 6 Proliferation response: samples from the medium and high dose cohorts show increased vector-induced proliferation at 90 to 180 days after gene therapy. Such a proliferation was not induced by recall antigens (purified protein derivatives of Bacillus Calmette-Guérin [BCG], Mycobacterium tuberculosis [PPD], or tetanus toxoid [TT] antigen). Activation (expression of CD69) profile: CD4+ T-cells feature increased activation (CD69 expression) after exposure to AAV8.CNGA3, but also after exposure to Mycobacterium tuberculosis [PPD] and tetanus toxoid [TT] antigen. CD8+ T-cells show no reactivity at all. CD19+ B-cells and CD56+ NK-cells show an activation pattern similar to CD4+ T-cells with increased CD69 expression after stimulation with AAV8.CNGA3, PPD or TT antigen (especially at day 90 through day 180 and more so for medium and high dose samples). Cytokine response assays: quantification of key cytokine levels by ELISA for GM-CSF, IFNδ, TNFβ, IL-17, IL-5, IL-13, IL-10, IL-6, TNFα, and IL-1α was done following exposure to serial dilutions of vector, BCG, PPD and TT. Elevated levels of GM-CSF, IFNδ and TNFβ could be measured after exposing the PBMCs to AAV8.CNGA3 at day 90 and 180 in samples from medium and high dose subjects. There was some induction of IL-1a at day 90 in medium and high dose samples when stimulating with AAV8.CNGA3, but also after stimulating with PPD. n.t.: not tested

© 2020 Fischer MD et al. *JAMA Ophthalmology.*

eTable 7: Significance analyses in functional outcomes of the control eye.

**Outcome parameter**	**Significance test control eye baseline vs. 0.5 yr**	**Significance test control eye baseline vs. 1 yr**
	parametric / non-parametric	*parametric / non-parametric*
BCVA**‡**	0.62 / 0.62	0.08 / 0.08
Contrast sensitivity	0.17 / 0.20	**0.043** / **0.028**
Flicker fusion frequency	0.59 / 0.51	0.79 / 0.78
Mean retinal sensitivity	0.53 / 0.86	0.96 / 0.94
Fixation stability	0.42 / 0.48	0.81 / 0.73
CCT**†** ellipse area	0.13 / 0.075	0.13 / 0.13
Full stimulus threshold	0.31 / 0.27	0.60 / 0.93

eTable 7: **‡**BCVA best-corrected visual acuity, **†**CCT Cambridge colour test, + functional improvement, parametric: two-sided, paired t-test, non-parametric: Wilcoxon signed-rank test.

eTable 8: Ophthalmological outcomes - tests of significance

	Tendency of change	treated eye				delta (treated – control eye)			
		6m vs. screening		1y vs. screening		6m vs. screening.		1y vs. screening	
		par	npar	par	npar	par	npar	par	npar
**Best corrected visual acuity (ETDRS)** [1]	↑[2]	**0.005**	**0.018**	**0.010**	**0.020**	0.11	0.14	0.49	0.44
**Contrast sensitivity (3 m)**	↑	**0.023**	**0.028**	**0.003**	**0.008**	0.28	0.23	0.33	0.192
**Contrast sensitivity (1 m)**	↑	**0.002**	**0.007**	0.36	0.154	0.24	0.23	0.45	0.48
Flicker fusion frequency	↑	0.124	0.173	0.53	0.67	0.158	0.109	0.62	0.37
**Cambridge colour test - protan axis**	↓	0.139	0.176	**0.011**	**0.028**	0.064	0.063	0.98	0.75
Cambridge colour test - deutan axis	↓	0.23	0.173	0.171	0.173	0.89	0.61	0.82	0.75
Cambridge colour test - tritan axis	↓	0.105	0.128	0.46	0.35	0.77	0.45	0.104	1.00
**Cambridge colour test - ellipse area**	↓	**0.04**	**0.046**	0.11	0.12	**0.049**	0.075	0.13	0.12
Microperimetry - mean sensitivity		0.21	0.18	0.53	0.53	0.24	0.40	0.71	0.60
Microperimetry - fixation stability 2°	---	0.28	0.27	0.49	0.24	0.194	0.24	0.80	0.61
Microperimetry - fixation stability 4°	---	0.33	0.40	0.50	0.31	0.29	0.29	0.88	0.92
Full (red) stimulus threshold	---	0.58	0.77	0.37	0.62	0.34	0.40	0.47	0.83
Full (blue) stimulus threshold	---	0.25	0.28	0.47	0.73	0.34	0.50	0.35	0.50

© 2020 Fischer MD et al. *JAMA Ophthalmology.*

Foveal thickness	---	0.47	0.67	0.52	0.62	0.88	0.91	0.126	0.097
Pupil baseline diameter red light	↓	**0.01**	**0.05**	0.14	0.11	**0.005**	**0.011**	1.0	0.95
Pupil relative constriction red light	↓	0.11	0.14	**0.001**	**0.008**	0.91	0.95	0.96	0.86
Pupil diameter blue light long	↓	**0.011**	0.051	0.14	0.11	**0.005**	**0.011**	1.0	0.95
Pupil constriction blue light long		0.59	0.52	0.24	0.37	0.78	0.95	0.35	0.52
Pupil diameter blue light short	↓	**0.006**	**0.021**	**0.034**	0.066	0.91	0.95	0.28	0.37
Pupil constriction blue light short		0.22	0.21	0.36	0.44	0.33	0.47	0.44	0.52

eTable 8 [1]Boldface: significant results (not corrected for multiplicity), [2]arrows: tendencies regarding treatment eye, at least one p-value below 0.05, par: parametric testing (two-sided Student's t-test), npar: non-parametric testing (Wilcoxon Signed Rank test).

eTable 9: Study specific scale A3 (PRO) results

	Visit									
	Number of subjects					Test against zero				
Visit	2w	1m	3m	6m	1y	2w	1m	3m	6m	1y
Glare	9	9	9	9	9	0.45	0.26	0.069	0.084	0.084
Identification of people	9	9	9	9	9	0.35	0.35	0.169	0.169	0.111
**Identification of letters and numbers**	9	9	9	9	9	0.195	0.45	0.35	0.195	**0.050**
**Identification of colors**	9	9	9	9	9	0.169	**0.035**	0.35	**0.035**	0.095
Fixation	9	9	9	9	9	0.122	0.59	0.35	0.169	0.68
General satisfaction[1]	4	5	6	8	9	1/0/3	0/1/4	1/2/3	3/1/4	3/2/4

eTable 9 [1]Entries: no/neutral/yes, all other answers: not sure, number of subjects: excluding not sure, boldface: significant results (not corrected for multiplicity).

© 2020 Fischer MD et al. *JAMA Ophthalmology.*

eTable 10: VFQ-25 results

		Visit						
	Tendency	Number of subjects				Test against screening (p-values)		
		Scr	1m	6m	1y	1m	6m	1y
General health	---	9	9	9	9	0.73	0.169	0.195
General vision	---	9	9	9	9	0.59	0.35	0.81
Ocular pain	---	9	9	9	9	0.056	0.051	0.59
**Near activities**	↑	9	9	9	9	0.30	**0.05**	0.084
Distance activities	---	9	9	9	9	0.72	0.44	0.59
Social function	↑	9	9	9	9	0.22	0.081	0.141
Mental health	↑	9	9	9	9	0.51	0.122	0.67
**Role limitations**	↑	9	9	9	9	**0.040**	**0.028**	0.68
Dependency	---	9	9	9	9	0.38	0.50	0.62
**Color vision**	↑	9	9	9	9	**0.002**	0.081	**0.021**
**Peripheral vision**	↑	9	9	9	9	**0.035**	0.169	1.00

eTable 10 Scr: screening visit, boldface: significant results (not corrected for multiplicity).

eTable 11: BSI results

		Visit						
	Tendency	Number of subjects				Test against screening (p-values)		
		Scr	1m	6m	1y	1m	6m	1y
Aggression	↓	9	9	9	9	0.064	**0.012**	**0.010**
Fear	↓	9	9	9	9	**0.020**	0.108	**0.042**
Depression	↓	9	9	9	9	**0.046**	0.129	0.098
Paranoia		9	9	9	9	0.093	0.60	0.087
Phobia	↓	9	9	9	9	0.065	**0.018**	0.088
Psychoticism	---	9	9	9	9	0.107	0.77	0.18
Somatization	---	9	9	9	9	0.73	0.110	0.84
Insecurity	↓	9	9	9	9	0.11	**0.036**	0.12
Compulsiveness	↓	9	9	9	9	0.23	0.17	**0.050**
Additional items		9	9	9	9	0.80	0.59	0.40
tGSI	↓	9	9	9	9	**0.031**	**0.004**	**0.023**
tPSDI	↓	9	9	9	9	0.290	**0.015**	**0.047**
tPST	↓	9	9	9	9	**0.049**	**0.006**	**0.018**

eTable 11 Scr: screening visit, boldface: significant results (not corrected for multiplicity). tGSI: t-transformed global severity index, tPSDI: t-transformed positive symptom distress index, tPST: t-transformed positive symptom total.

© 2020 Fischer MD et al. *JAMA Ophthalmology.*

REFERENCES

1. Barth H, Berg PA, Klein R. Methods for the in vitro determination of an individual disposition towards TH1- or TH2-reactivity by the application of appropriate stimulatory antigens. Clin Exp Immunol 2003;134:78-85.
2. Thaher F, Plankenhorn S, Klein R. Differential effects of the tumor necrosis factor alpha-blocker infliximab and etanercept on immunocompetent cells in vitro. Int Immunopharmacol 2011;11:1724-31.
3. Bustin SA, Benes V, Garson JA, et al. The MIQE guidelines: minimum information for publication of quantitative real-time PCR experiments. Clin Chem 2009;55:611-22.
4. Armbruster DA, Pry T. Limit of blank, limit of detection and limit of quantitation. Clin Biochem Rev 2008;29 Suppl 1:S49-52.
5. Samulski RJ, Chang LS, Shenk T. A recombinant plasmid from which an infectious adeno-associated virus genome can be excised in vitro and its use to study viral replication. J Virol 1987;61:3096-101.
6. Zanta-Boussif MA, Charrier S, Brice-Ouzet A, et al. Validation of a mutated PRE sequence allowing high and sustained transgene expression while abrogating WHV-X protein synthesis: application to the gene therapy of WAS. Gene Ther 2009;16:605-19.
7. Ayuso E, Blouin V, Lock M, et al. Manufacturing and characterization of a recombinant adeno-associated virus type 8 reference standard material. Hum Gene Ther 2014;25:977-87.
8. Michalakis S, Muhlfriedel R, Tanimoto N, et al. Restoration of cone vision in the CNGA3-/- mouse model of congenital complete lack of cone photoreceptor function. Mol Ther 2010;18:2057-63.
9. Meyer MR, Angele A, Kremmer E, Kaupp UB, Muller F. A cGMP-signaling pathway in a subset of olfactory sensory neurons. Proc Natl Acad Sci U S A 2000;97:10595-600.
10. Wilhelm B, Koegel A, Kahle N, et al. How do patients rate their subjective symptoms after CNGA3 gene therapy: First application of the instrument A3-PRO Invest Ophthalmol Vis Sci 2017;58:4678.
11. Marella M, Pesudovs K, Keeffe JE, O'Connor PM, Rees G, Lamoureux EL. The psychometric validity of the NEI VFQ-25 for use in a low-vision population. Invest Ophthalmol Vis Sci 2010;51:2878-84.
12. Crameri A, Schuetz C, Andreae A, et al. The Brief Symptom Inventory and the Outcome Questionnaire-45 in the Assessment of the Outcome Quality of Mental Health Interventions. Psychiatry J 2016;2016:7830785.

© 2020 Fischer MD et al. *JAMA Ophthalmology.*

eFigure 1

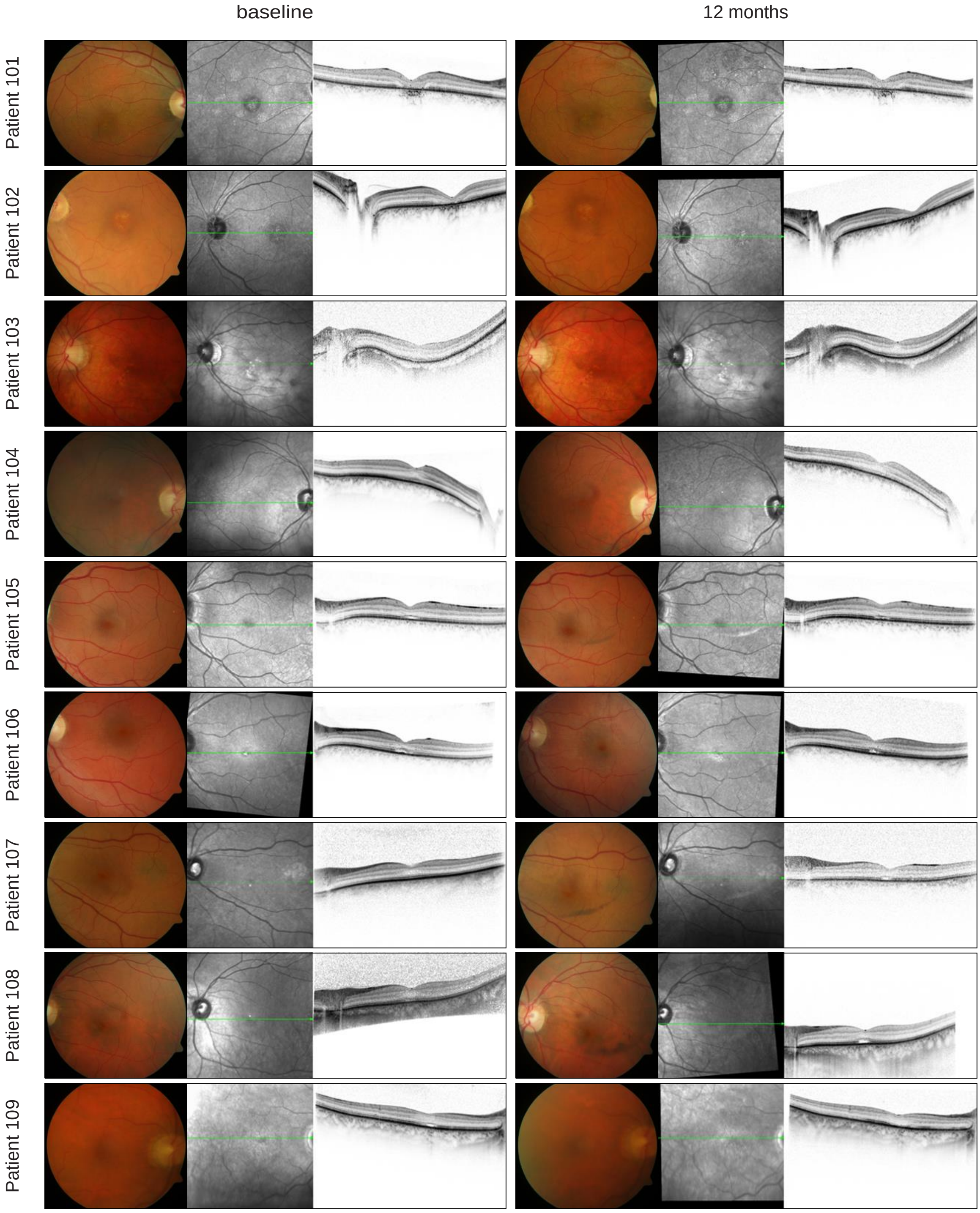

eFigure 1. Color photographs, infrared images and virtual cross sections of treated eyes. Color photographs and infrared images of the fundus were recorded before and 12 months after gene therapy in all patients. Optical coherence tomography was performed to gain virtual cross sections through the treated area, demonstrating no adverse effect on the retinal anatomy.

© 2020 Fischer MD et al. *JAMA Ophthalmology.*

eFigure 2

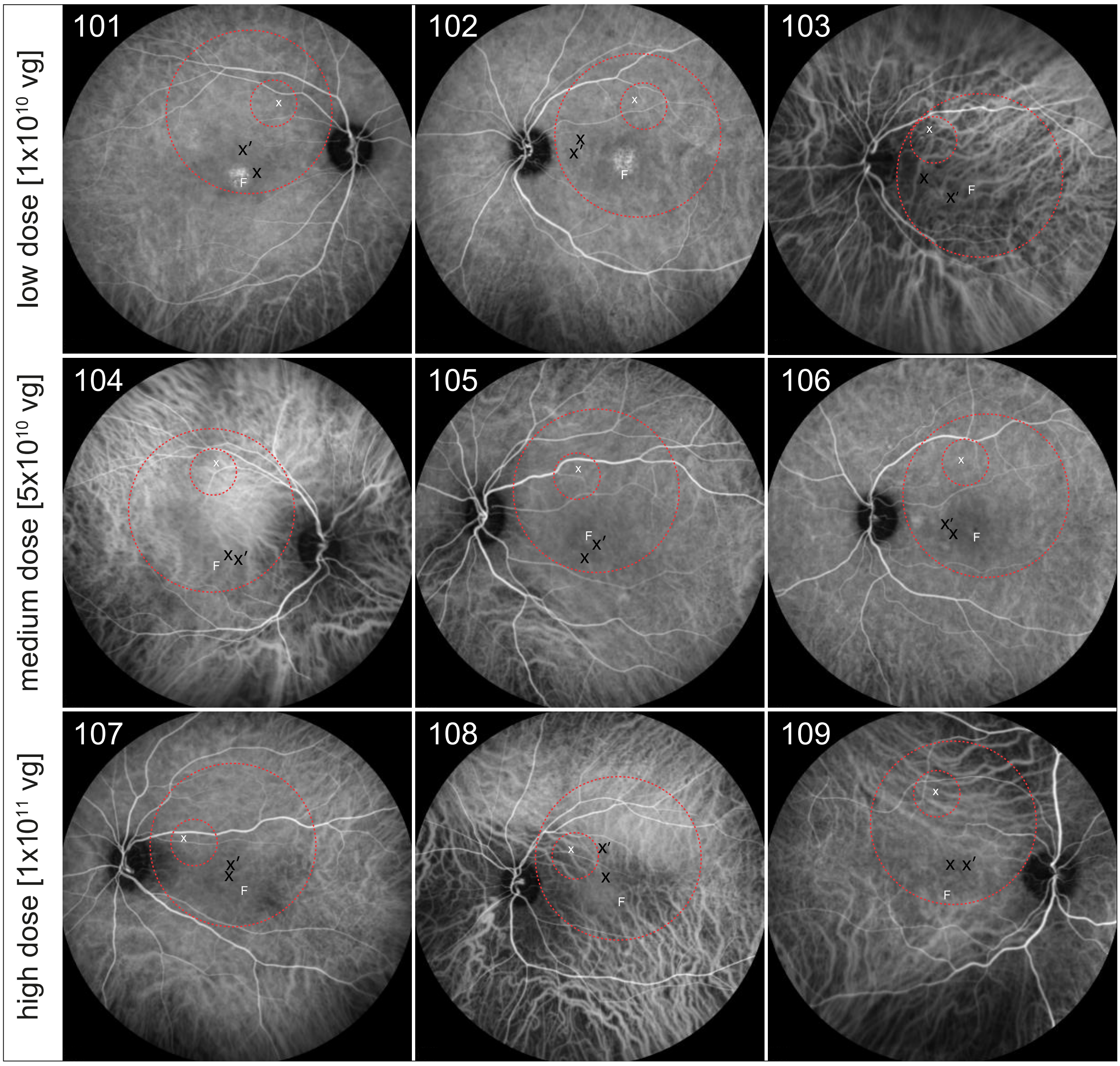

eFigure 2. Treatment area and effect on foveal anatomy of treated eyes. Indocyanine green angiography images showed no change in retinal and choroidal perfusion. Target area included the cone photoreceptor rich macula in all cases (small dashed circles: pre-bleb, larger circles: extent of full bleb, white cross: retinotomy, black cross: preferred retinal locus [PRL = fixation] at baseline, black cross with apostrophe: PRL 1 year after treatment).

© 2020 Fischer MD et al. *JAMA Ophthalmology.*

eFigure 3

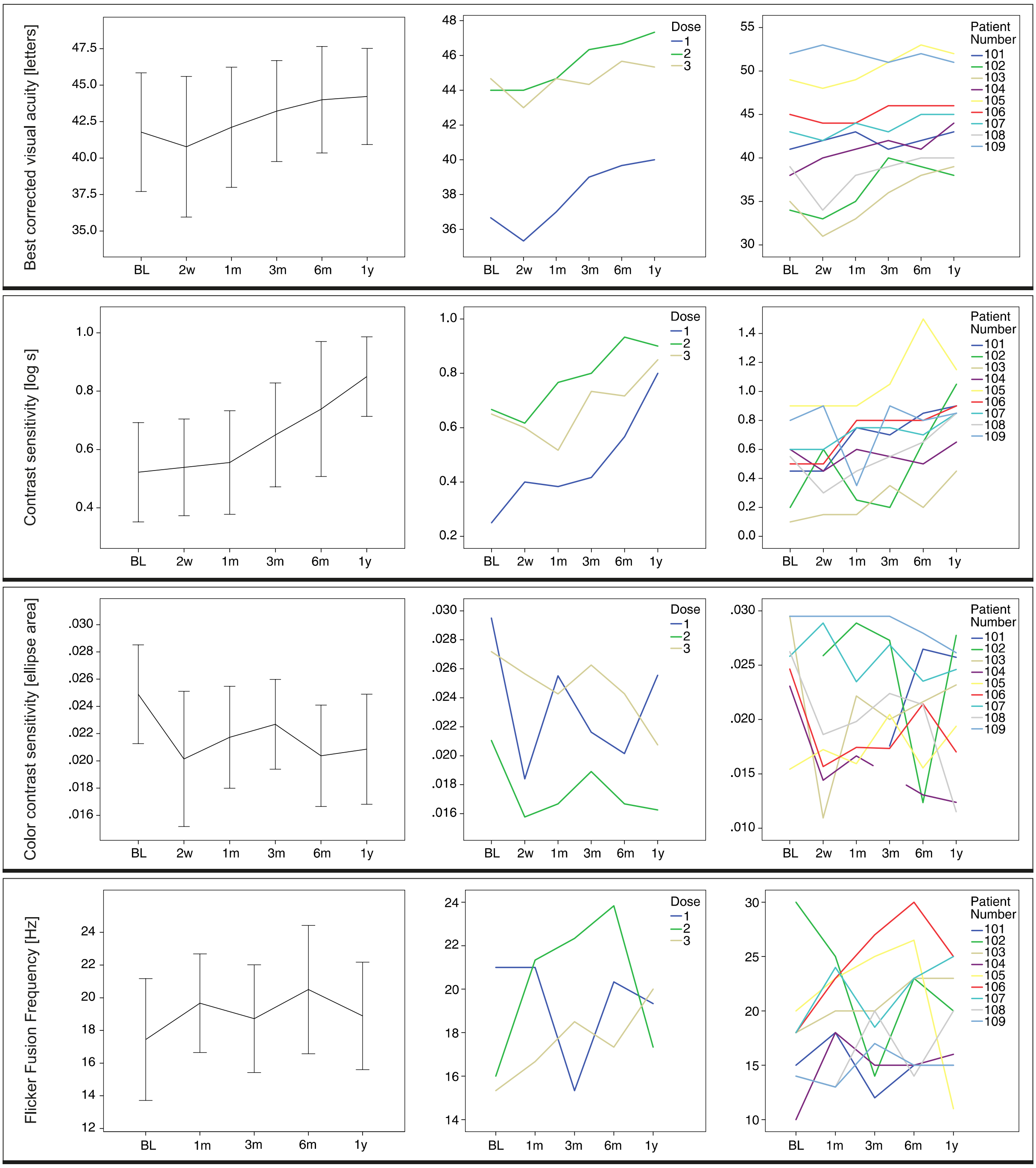

eFigure 3. Visual acuity, contrast sensitivity, color contrast sensitivity, and temporal resolution of the treated eyes. Top panel shows the best corrected visual acuity (BCVA) as sum score of identified letters on a standardized chart over time. Second panel shows the contrast sensitivity in patients from baseline (BL) over time. The third panel displays the threshold to discriminate color contrasts (ellipse area) over time. The bottom panel shows the patient's ability to discriminate light stimuli as individual signals at higher frequency. All data are presented as mean±2*standard error of all nine patients (left), mean of n=3 by dose group (middle) and as individual traces (right).

© 2020 Fischer MD et al. *JAMA Ophthalmology.*

eFigure 4

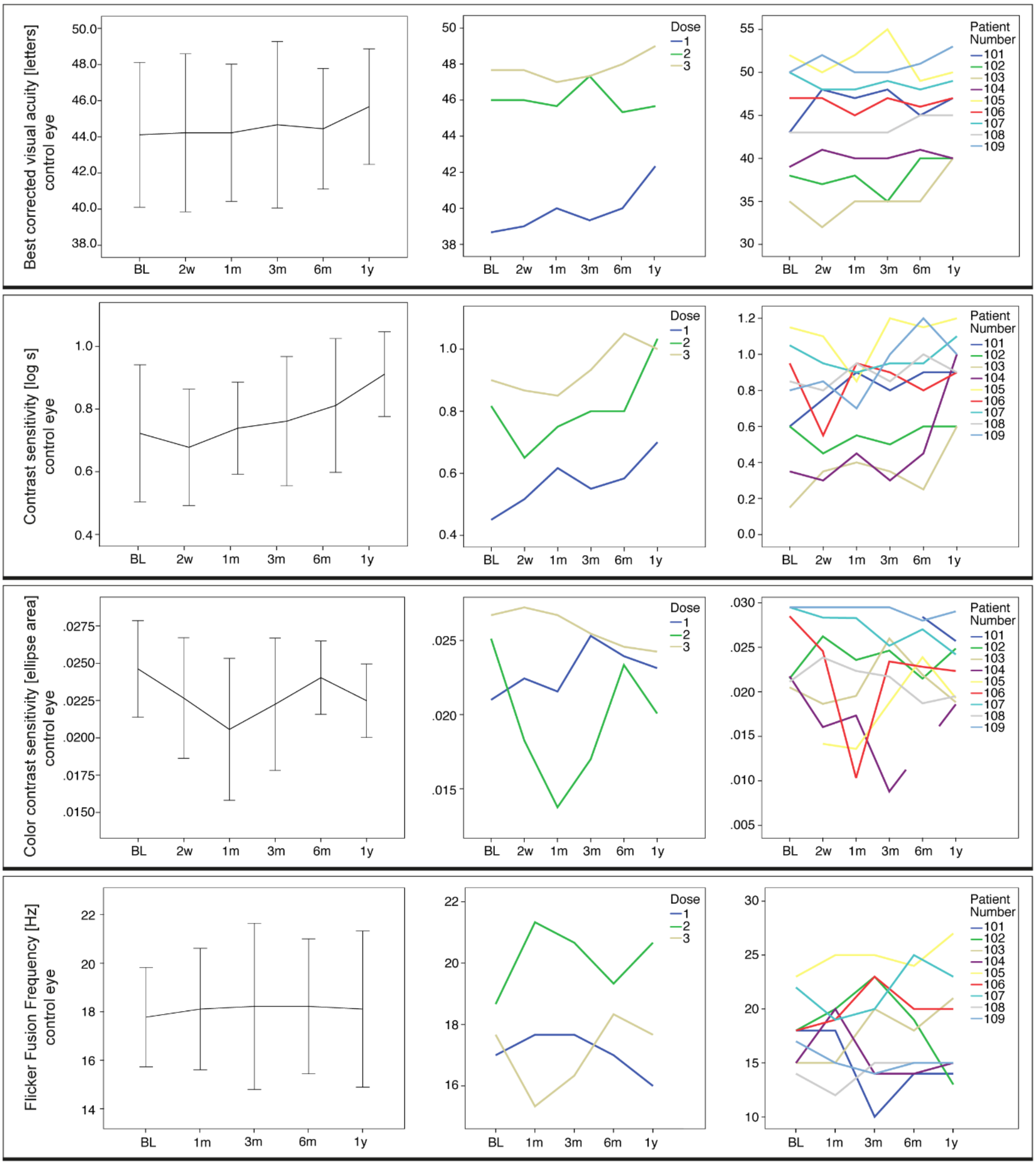

eFigure 4. Visual acuity, contrast sensitivity, color contrast sensitivity, and temporal resolution of the untreated control eyes. Top panel shows the best corrected visual acuity (BCVA) as sum score of identified letters on a standardized chart over time. Second panel shows the contrast sensitivity in patients from baseline (BL) over time. The third panel displays the threshold to discriminate color contrasts (ellipse area) over time. The bottom panel shows the patient's ability to discriminate light stimuli as individual signals at higher frequency. All data are presented as mean±2*standard error of all nine patients (left), mean of n=3 by dose group (middle) and as individual traces (right).

eFigure 5

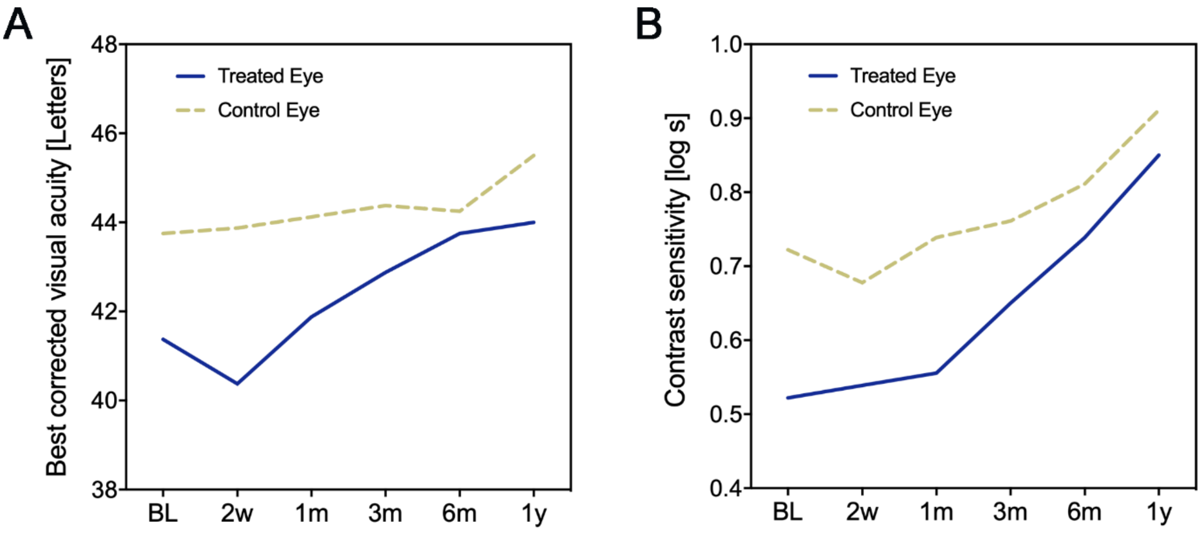

eFigure 5. Visual acuity and contrast sensitivity comparison between the treated eyes and untreated control eyes. Summary graphs illustrating the development of the mean best corrected visual acuity (A) and the contrast sensitivity (B) among treated eyes and untreated control eyes over the 1-year duration of the study. In order to facilitate the comparison the mean values are plotted without standard deviation. Given that the worse eye was chosen as the study eye for treatment, the mean values for the treated eyes at baseline were lower than the corresponding mean values of the control eyes. Over the duration of the study the mean values of the treated eyes reached close to the corresponding values of control eyes.

© 2020 Fischer MD et al. *JAMA Ophthalmology.*

eFigure 6

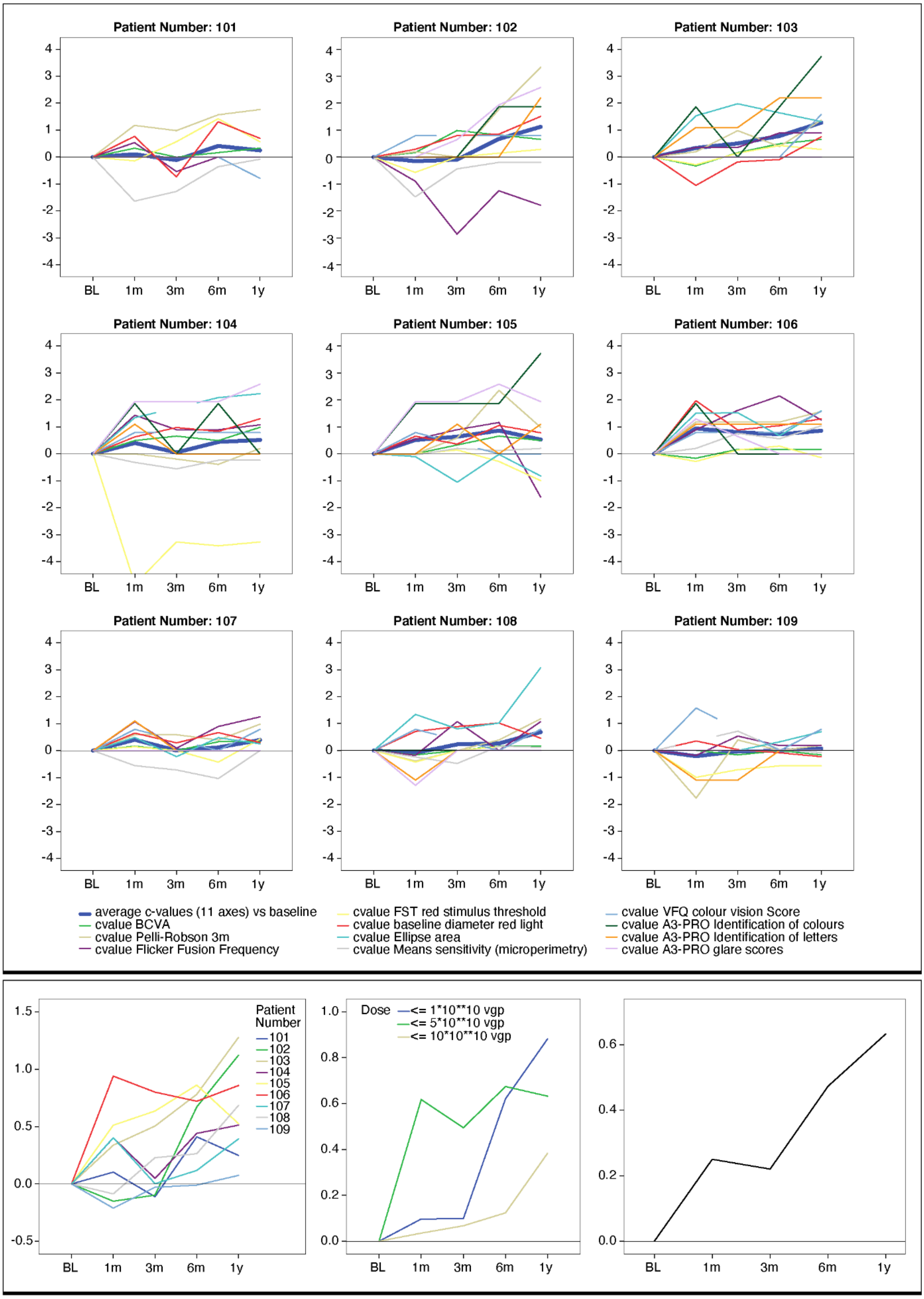

© 2020 Fischer MD et al. *JAMA Ophthalmology.*

eFigure 6. Z-score normalization of efficacy endpoints. Eleven surrogate markers for cone photoreceptor function were combined in a z-score normalization approach to test efficacy across endpoints. Top panel shows the change of all 11 markers (plus the mean = bold blue line) in each individual patient over time from baseline (y-axis indicates change in standard deviations from baseline). Bottom panel shows all mean scores by patient (left), mean of mean scores by dose group (middle) and mean of all (right).

© 2020 Fischer MD et al. *JAMA Ophthalmology.*
